# Structure-Based Rational Design of Small α-Helical Peptides with Broad-Spectrum Activity against Multidrug-Resistant Pathogens

**DOI:** 10.1021/acs.jmedchem.2c01708

**Published:** 2022-12-27

**Authors:** Sandeep Lohan, Anastasia G. Konshina, Roman G. Efremov, Innokentiy Maslennikov, Keykavous Parang

**Affiliations:** † Center for Targeted Drug Delivery, Department of Biomedical and Pharmaceutical Sciences, Chapman University School of Pharmacy, Harry and Diane Rinker Health Science Campus, 94 01 Jeronimo Road, Irvine, California92618, United States; ‡ AJK Biopharmaceutical, 5270 California Avenue, Irvine, California92617, United States; § Structural Biology Research Center, Department of Biomedical and Pharmaceutical Sciences, Chapman University School of Pharmacy, Harry and Diane Rinker Health Science Campus, 9401 Jeronimo Road, Irvine, California92618, United States; ∥ M.M. Shemyakin & Yu.A. Ovchinnikov Institute of Bioorganic Chemistry, Russian Academy of Sciences, Miklukho-Maklaya Street, 16/10, Moscow117997, Russia; ⊥ National Research University Higher School of Economics, Myasnitskaya ul. 20, Moscow101000, Russia; # Moscow Institute of Physics and Technology (State University), Dolgoprudny, Moscow Oblast141701, Russia

## Abstract

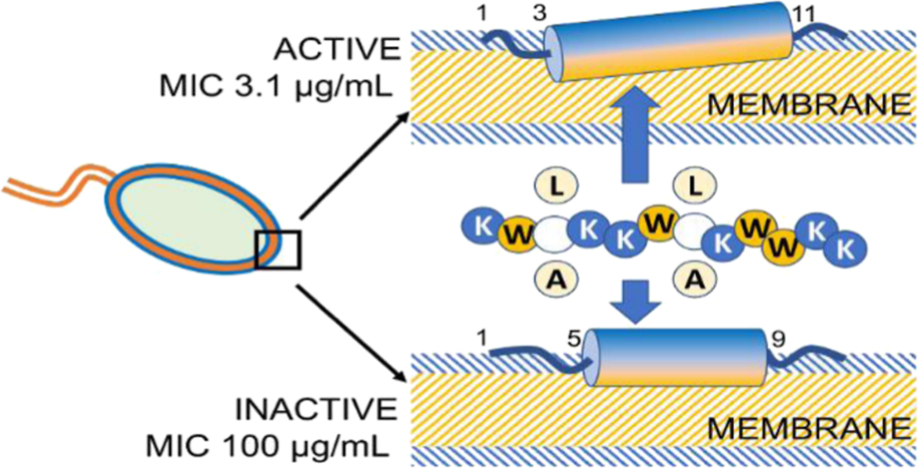

A series of small (7–12 mer) amphipathic cationic peptides were designed and synthesized to create short helical peptides with broad-range bactericidal activity and selectivity toward the bacterial cells. The analysis identified a lead 12-mer peptide **8b** with broad-spectrum activity against Gram-positive (MIC = 3.1–6.2 μg/mL) and Gram-negative (MIC = 6.2–12.5 μg/mL) bacteria and selectivity toward prokaryotic versus eukaryotic cells (HC_50_ = 280 μg/mL, >75% cell viability at 150 μg/mL). The rapid membranolytic action of **8b** was demonstrated by a calcein dye leakage assay and confirmed using scanning electron microscopy. According to circular dichroism and NMR spectroscopy, the peptides have an irregular spatial structure in water. A lipid bilayer induced an amphipathic helix only in 12-mer peptides, including **8b**. Molecular dynamics simulations provided detailed information about the interaction of **8b** and its closest analogues with bacterial and mammalian membranes and revealed the roles of particular amino acids in the activity and selectivity of peptides.

## 1 Introduction

Accelerating growth and global expansion of microbial resistance has resulted in a major threat to public health, restoring infectious diseases to the list of the leading cause of mortality worldwide.^1^ Earlier, drug-resistant pathogens, such as methicillin-resistant *Staphylococcus aureus* (MRSA) and vancomycin-resistant *enterococci*, as potential causative of various life-threatening infections were mainly confined to nosocomial environments only. However, the extraordinary ability of these pathogens to develop resistance resulted in high incidences of community-acquired infections.^2^ As per the World Health Organization, *Acinetobacter baumannii* is at the top of the list of the microbes that pose the greatest threat to human health and for which new antibiotics are urgently needed.^3^ Over the past 2 decades, while antibiotic-resistant microbes have been emerging exponentially, the development of new anti-infectives has sharply declined.^4^ Thus, developing new antibiotics to cure infectious diseases has become an urgent need and attracted the attention of biomedical researchers worldwide.

Tremendous efforts have been made to develop antibiotics inspired by antimicrobial peptides (AMPs).^5,6^ Natural AMPs have been isolated from animals, plants, and bacteria as the key components of their innate immunity.^7^ AMPs display broad spectra of activity against bacteria (Gram-positive and Gram-negative), fungi (including yeasts), parasites (including planaria and nematodes), and viruses (such as HIV and herpes simplex).^8,9^ Unlike most traditional antibiotics that kill or inhibit the growth of bacteria by targeting various key biosynthetic processes, AMPs are known to interact with the bacterial membrane and either destabilize the physical integrity of the latter or translocate through it and interact with intracellular macromolecules associated with vital metabolic processes of the cell.^10^

The attractive therapeutic features of AMPs, such as the broad-spectrum activity against antibiotic-resistant pathogens, unique mode of action, and the ability to kill target bacteria rapidly,^11^ leave minimal scope for pathogens to develop resistance.^12^ These unique characteristics of AMPs make them an ideal class of molecules to be developed as next-generation antibiotics. Despite attractive therapeutic features, a few drawbacks, such as the comparatively large molecular size and low metabolic stability, curtail the clinical applications of AMPs.^13^ Most of the native AMPs are amphiphilic molecules with size ranging from 12 to 50 residues.^11^ Previous studies on structure–activity relationships of natural and synthetic AMPs have identified that the net charge/hydrophobic bulk ratio and amphipathicity, defined by a secondary structure, are essential for peptide biological activity.^9,14^ Moreover, numerous studies have demonstrated a direct correlation between AMP hydrophobic content and toxicity.^15^

While several mechanisms have been proposed to describe the peptide–lipid interactions, the precise mechanism of the lytic activity of AMPs is a matter of debate.^14^ According to a prevailed model, a net positive charge of AMPs in physiological conditions is crucial for the initial electrostatic interaction with anionic components of the bacterial membrane. Following such recognition and keeping their hydrophilic surface toward the phospholipid headgroups, AMPs reorient the hydrophobic surface toward the lipid alkyl chains and incorporate into the membrane, ultimately destabilizing the membrane, resulting in a loss of membrane fluidity and causing cell death.^11,16^ While numerous studies describe the AMPs’ action against bacterial membranes on a cellular level, there is still little understanding of the process with atomistic details. Also, the prevailing model^17^ presumes a structural rearrangement of AMPs upon interaction with the membrane. However, the impact of spatial structure and structural flexibility (stability) on the peptide membranolytic activity is still not clear.

Thus, with an ultimate goal to circumvent the clinical drawbacks of AMPs, and the primary objective to understand the role of specific amino acids, spatial structure, and structural stability in peptides’ activity and selectivity, we designed a library of short, 7–12 mer AMPs possessing the key features of a net cationic charge, hydrophobicity, and spatial amphipathicity. The shortest 7-mer peptides were composed of four Arg and three Trp to provide positive charge and hydrophobic bulk, respectively. The cationic (Arg or Lys) and hydrophobic (Leu, Ile, or Ala) residues were added at the specific positions in the sequences to create continuous cationic and hydrophobic surfaces if a peptide adopts the helical conformation. The activity screening against Gram-positive and Gram-negative nonresistant and resistant bacterial strains and in vitro cytotoxicity assessment using normal human cells, including red blood cells, revealed the lead peptides with high potency and selectivity. Further studies showed the lead peptides’ fast and efficient bactericidal kinetics and indicated the membranolytic action as their primary bactericidal mechanism. The membrane perturbation effect of the lead peptides was further confirmed by scanning electron microscopy (SEM) analysis. NMR spectroscopy and molecular dynamics simulations in water and in the presence of the lipid bilayer mimicking bacterial and mammalian membrane, conducted for the lead peptides and closest analogues, revealed structural details of their interactions with a model membrane, including membrane-induced changes of the secondary structure. The entirety of the experimental and computational results allowed us to identify the roles of specific amino acids in providing and maintaining conformational features, such as spatial structure, structure stability, and membrane-induced structural changes, and correlate the features with the activity and toxicity of the peptides. Amino acids with long hydrophobic side chains (Leu and Ile) were essential for the stability of the amphipathic helix on the bilayer surface, interaction, and deep penetration of the peptides into the hydrophobic core of the cell membrane, thus establishing the basis for rapid membranolytic action of the peptides. In turn, among two cationic amino acids, Arg, but not Lys, induces strong interaction of the peptides with the mammalian membrane. As a result, the peptides containing Lys demonstrated much lower toxicity toward mammalian cells than those containing Arg.

## 2 Results

### 2.1 SAR-Based Design and Synthesis

**Figure 1:**
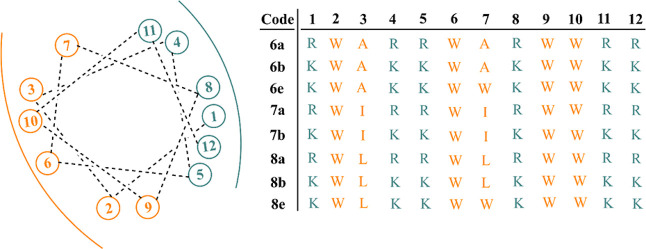
Helical wheel projection of the 12-mer peptides. Cationic and hydrophobic residues are depicted in blue and red, respectively.

All peptides were designed to attain amphipathicity upon adopting helical structures with the hydrophobic residues on one side and the cationic residues on the opposite side of the helical wheel projection [Figures 1 and S1 (Supporting Information)]. With this structural arrangement in mind, we designed several sets of 7–12 amino acid-long peptides composed of cationic (Arg or Lys) and hydrophobic (Trp, and either Leu, Ile, or Ala) amino acids (Table 1). All peptides were synthesized by using the standard Fmoc/*t*Bu solid-phase peptide synthesis protocol and purified as described in the Experimental Section. The purity of all synthesized peptides was found to be >95%, as determined by analytical RP-HPLC. The chemical identity of the peptides was verified by high-resolution mass spectrometry (HR-MS) and NMR spectroscopy. The purity and HR-MS data of all the synthesized peptides are provided in the Supporting Information.

**Table 1:** Antibacterial and Hemolytic Activity Results of the Peptides (**1a–8d**)^a^

		MIC^c^, μg/mL (μM)					
code	peptide sequence^b^	*S. aureus* (ATCC 29213)	MRSA (ATCC BAA-1556)	*E. coli* (ATCC 25922)	*P. aeruginosa* (ATCC 27883)	*K. pneumoniae* (ATCCBAA-1705)	HC_50_^d^, μg/mL (μM)
**1a**	NH_2_-R-W-R-R-W-W-R-CONH_2_	100 (83.30)	100 (83.30)	>100 (>83.30)	>100 (>83.30)	>100 (>83.30)	>500 (>416.52)
**1b**	NH_2_-R-R-W-W-R-R-W-CONH_2_	50 (41.65)	100 (83.30)	>100 (>83.30)	>100 (>83.30)	>100 (>83.30)	>500 (>416.52)
**1c**	NH_2_-W-R-R-W-W-R-R-CONH_2_	100 (83.30)	100 (83.30)	>100 (>83.30)	>100 (>83.30)	>100 (>83.30)	>500 (>416.52)
**1d**	NH_2_-R-R-W-R-R-W-W-CONH_2_	100 (83.30)	100 (83.30)	>100 (>83.30)	>100 (>83.30)	>100 (>83.30)	>500 (>416.52)
**1e**	NH_2_-R-W-W-R-R-W-R-CONH_2_	100 (83.30)	100 (83.30)	>100 (>83.30)	>100 (>83.30)	>100 (>83.30)	>500 (>416.52)
**2a**	NH_2_-R-W-W-R-R-W-A-R-CONH_2_	>100 (>78.65)	>100 (>78.65)	>100 (>78.65)	>100 (>78.65)	>100 (>78.65)	>500 (>393.23)
**2b**	NH_2_-R-W-W-R-R-A-W-R-CONH_2_	>100 (>78.65)	>100 (>78.65)	>100 (>78.65)	>100 (>78.65)	>100 (>78.65)	>500 (>393.23)
**2c**	NH_2_-R-W-A-R-R-W-W-R-CONH_2_	>100 (>78.65)	>100 (>78.65)	>100 (>78.65)	>100 (>78.65)	>100 (>78.65)	>500 (>393.23)
**2d**	NH_2_-W-R-R-W-A-R-R-W-CONH_2_	>100 (>78.65)	>100 (>78.65)	>100 (>78.65)	>100 (>78.65)	>100 (>78.65)	>500(>393.23)
**2e**	NH_2_-A-R-R-W-W-R-R-W-CONH_2_	100 (78.65)	100 (>78.65)	>100 (>78.65)	>100 (>78.65)	>100 (>78.65)	>500(>393.23)
**3a**	NH_2_-R-W-W-R-R-A-W-R-A-CONH_2_	>100 (>74.48)	>100 (>74.48)	>100 (>74.48)	>100 (>74.48)	>100 (>74.48)	>500 (>372.42)
**3b**	NH_2_-R-A-W-R-R-W-W-R-A-CONH_2_	>100 (>74.48)	>100 (>74.48)	>100 (>74.48)	>100 (>74.48)	>100 (>74.48)	>500(>372.42)
**3c**	NH_2_-R-W-A-R-R-W-W-R-A-CONH_2_	>100 (>74.48)	>100 (>74.48)	>100 (>74.48)	>100 (>74.48)	>100 (>74.48)	>500(>372.42)
**3d**	NH_2_-R-W-W-R-A-A-W-R-R-CONH_2_	>100 (>74.48)	>100 (>74.48)	>100 (>74.48)	>100 (>74.48)	>100 (>74.48)	>500(>372.42)
**3e**	NH_2_-R-W-W-R-R-A-W-A-R-CONH_2_	>100 (>74.48)	>100 (>74.48)	>100 (>74.48)	>100 (>74.48)	>100 (>74.48)	>500(>372.42)
**3f**	NH_2_-A-W-W-R-R-A-W-R-R-CONH_2_	>100 (>74.48)	>100 (>74.48)	>100 (>74.48)	>100 (>74.48)	>100 (>74.48)	>500(>372.42)
**4a**	NH_2_-R-A-W-R-R-A-W-R-A-W-CONH_2_	>100 (>70.74)	>100 (>70.74)	>100 (>70.74)	>100 (>70.74)	>100 (>70.74)	>500 (>353.69)
**4b**	NH_2_-R-A-W-R-R-W-W-R-A-A-CONH_2_	>100 (>70.74)	>100 (>70.74)	>100 (>70.74)	>100 (>70.74)	>100 (>70.74)	>500 (>353.69)
**4c**	NH_2_-R-W-W-R-R-A-W-R-A-A-CONH_2_	>100 (>70.74)	>100 (>70.74)	>100 (>70.74)	>100 (>70.74)	>100 (>70.74)	>500(>353.69)
**4d**	NH_2_-R-A-W-R-R-W-A-R-A-W-CONH_2_	>100 (>70.74)	>100 (>70.74)	>100 (>70.74)	>100 (>70.74)	>100 (>70.74)	>500(>353.69)
**4e**	NH_2_-R-W-A-R-R-A-W-R-A-W-CONH_2_	>100 (>70.74)	>100 (>70.74)	>100 (>70.74)	>100 (>70.74)	>100 (>70.74)	>500(>353.69)
**4f**	NH_2_-R-A-W-R-A-W-W-R-R-A-CONH_2_	>100 (>70.74)	>100 (>70.74)	>100 (>70.74)	>100 (>70.74)	>100 (>70.74)	>500(>353.69)
**4g**	NH_2_-R-A-W-R-R-W-W-A-R-A-CONH_2_	>100 (>70.74)	>100 (>70.74)	>100 (>70.74)	>100 (>70.74)	>100 (>70.74)	>500(>353.69)
**4h**	NH_2_-A-A-W-R-R-W-W-R-R-A-CONH_2_	>100 (>70.74)	>100 (>70.74)	>100 (>70.74)	>100 (>70.74)	>100 (>70.74)	>500(>353.69)
**5a**	NH_2_-R-A-A-R-R-W-A-R-W-W-R-CONH_2_	100 (63.70)	100 (63.70)	>100 (>63.70)	>100 (>63.70)	>100 (>63.70)	>500 (>318.50)
**5b**	NH_2_-R-W-A-R-R-W-A-R-W-W-R-CONH_2_	50 (29.67)	100 (59.35)	100 (59.35)	100 (59.35)	>100 (>59.35)	>500 (>296.74)
**5c**	NH_2_-R-W-I-R-R-W-I-R-W-W-R-CONH_2_	50 (28.26)	50 (28.26)	100 (56.52)	50 (28.26)	100 (56.52)	>500 (>282.62)
**5d**	NH_2_-R-W-L-R-R-W-L-R-W-W-R-CONH_2_	25 (14.13)	25 (14.13)	50 (28.26)	50 (28.26)	50 (28.26)	130 (73.48)
**5e**	Ac-R-W-I-R-R-W-I-R-W-W-R-CONH_2_	50 (27.61)	50 (27.61)	100 (55.21)	50 (27.61)	100 (55.21)	60 (33.13)
**5f**	Ac-R-W-L-R-R-W-L-R-W-W-R-CONH_2_	12.5 (6.90)	12.5 (6.90)	50 (27.61)	25 (13.80)	50 (27.61)	65 (35.89)
**6a**	NH_2_-R-W-A-R-R-W-A-R-W-W-R-R-CONH_2_	50 (27.16)	50 (27.16)	>100 (>54.31)	100 (54.31)	>100 (>54.31)	230 (124.92)
**6b**	NH_2_-K-W-A-K-K-W-A-K-W-W-K-K-CONH_2_	100 (59.77)	100 (59.77)	>100 (>59.77)	>100 (>59.77)	>100 (>59.77)	>500 (>298.85)
**6c**	Ac-R-W-A-R-R-W-A-R-W-W-R-R-CONH_2_	50 (26.55)	50 (26.55)	100 (53.10)	100 (53.10)	>100 (>53.10)	110 (58.41)
**6d**	Ac-K-W-A-K-K-W-A-K-W-W-K-K-CONH_2_	50 (29.15)	100 (58.30)	100 (58.30)	100 (58.30)	100 (58.30)	345 (201.15)
**6e**	NH_2_-K-W-A-K-K-W-W-K-W-W-K-K-CONH_2_	12.5 (6.99)	12.5 (6.99)	25 (13.98)	25 (13.98)	50 (27.96)	360 (201.32)
**7a**	NH_2_-R-W-I-R-R-W-I-R-W-W-R-R-CONH_2_	6.2 (3.22)	6.2 (3.22)	6.2 (3.22)	6.2 (3.22)	12.5 (6.49)	90 (46.74)
**7b**	NH_2_-K-W-I-K-K-W-I-K-W-W-K-K-CONH_2_	6.2 (3.53)	6.2 (3.53)	6.2 (3.53)	6.2 (3.53)	25 (14.23)	460 (261.77)
**7c**	Ac-R-W-I-R-R-W-I-R-W-W-R-R-CONH_2_	6.2 (3.15)	6.2 (3.15)	12.5 (6.35)	6.2 (3.15)	25 (12.70)	40 (20.33)
**7d**	Ac-K-W-I-K-K-W-I-K-W-W-K-K-CONH_2_	6.2 (3.45)	6.2 (3.45)	12.5 (6.95)	12.5 (6.95)	25 (13.89)	160 (88.92)
**8a**	NH_2_-R-W-L-R-R-W-L-R-W-W-R-R-CONH_2_	6.2 (3.22)	6.2 (3.22)	12.5 (6.49)	12.5 (6.49)	12.5 (6.49)	45 (23.37)
**8b**	NH_2_-K-W-L-K-K-W-L-K-W-W-K-K-CONH_2_	3.1 (1.76)	3.1 (1.76)	6.2 (3.53)	6.2 (3.53)	6.2 (3.53)	280 (159.34)
**8c**	Ac-R-W-L-R-R-W-L-R-W-W-R-R-CONH_2_	6.2 (3.15)	6.2 (3.15)	12.5 (6.35)	12.5 (6.35)	12.5 (6.35)	30 (15.25)
**8d**	Ac-K-W-L-K-K-W-L-K-W-W-K-K-CONH_2_	3.1 (1.72)	6.2 (3.45)	6.2 (3.45)	6.2 (3.45)	12.5 (6.95)	145 (80.59)
**8e**	NH_2_-K-W-L-K-K-W-W-K-W-W-K-K-CONH_2_	12.5 (6.83)	12.5 (6.83)	12.5 (6.83)	12.5 (6.83)	25 (13.66)	240 (131.13)
	daptomycin	0.7 (0.43)	1.5 (0.86)	ND^e^	ND^e^	ND^e^	ND^e^
	polymyxin B	ND^e^	ND^e^	0.7 (0.58)	1.5 (1.25)	0.7 (0.58)	ND^e^
	ciprofloxacin	1.5 (4.53)	3.1 (9.36)	0.7 (2.11)	0.7 (2.11)	1.5 (4.53)	ND^e^

a Results represent the highest MICs observed from the three independent experiments performed in triplicate.

b All the amino acid residues are presented in one-alphabet notation.

c Minimum inhibitory concentrations (MIC) were determined as the lowest concentration of the peptides that inhibited bacterial growth.

d HC_50_ is the concentration in μg/mL of peptides at which 50% hemolysis was observed.

e ND represents not determined. Values in parentheses represent the MICs and HC_50_ in μM.

**Figure 2:**
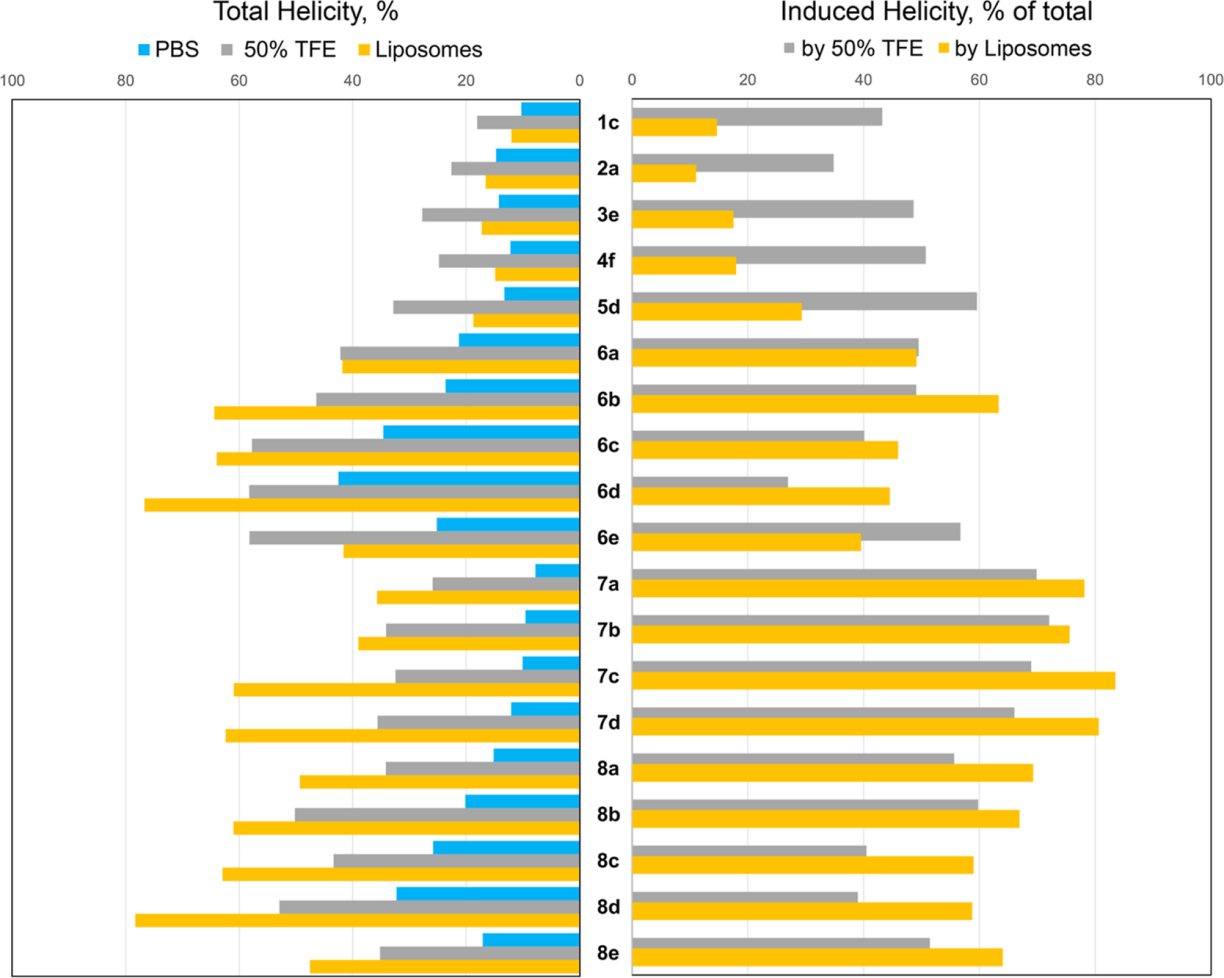
Assessment of the peptides’ helicity (in %) using CD spectroscopy data in different media. The total helicity (left panel) was calculated as described in ref^19^ using the CD data collected in PBS (blue), TFE (gray), and liposomes (orange). The induced helicity (right panel) was calculated as % of the total helicity of the peptide induced by TFE (gray) and liposomes (orange) relative to the helicity of the same peptide in PBS.

Many reports, including ours,^18^ previously identified that four cationic and three hydrophobic residues are required in a peptide for antimicrobial activity and selectivity toward the bacterial membrane. Accordingly, in the initial set of heptameric peptides **1a–e**, considering the pivotal role of net charge/hydrophobic ratio,^5^ we used four cationic (Arg) and three hydrophobic (Trp) residues (peptides **1a–e**, Table 1). However, 7-mer peptides **1a–e** did not show any antibacterial activity (MIC > 100 μg/mL, Table 1). Evaluation of the secondary structure by circular dichroism (CD) spectroscopy of a representative 7-mer peptide **1c** showed very low if any helicity in the presence of the liposomes [Figure 2 and Table S1 (Supporting Information)]. We hypothesized that the helical stabilizing ability of Ala^19^ may help the peptides to attain helical conformation and eventually demonstrate antibacterial activity. However, no improvement in antibacterial activity was observed for longer 8-mer (**2a–e**), 9-mer (**3a–f**), and 10-mer (**4a–h**) peptides having one, two, and three Ala residues, respectively (Table 1). CD spectroscopy analysis explains the lack of activity since, in the presence of liposomes, none of the 8-, 9-, and 10-mer peptides attained a helical conformation crucial for the formation of an amphipathic surface [Figure 2 and Table S1 (Supporting Information)]. These results are consistent with the general requirement of at least three turns (11 residues) for the formation of a stable helical conformation.^20^

Therefore, we designed 11-mer (**5a** and **5b**) and 12-mer (**6a**) peptides with an extra positive charge (one or two additional Arg) and hydrophobic bulk (replaced an Ala with Trp). The 11-mer peptide **5b** and the 12-mer peptide **6a** showed modest activity against *S. aureus* (MIC = 50 μg/mL; Table 1). At the same time, 12-mer peptide **6a** showed about 50% increase in helicity in the presence of liposomes, as compared to PBS [Figure 2 and Table S1 (Supporting Information)]. Thus, we obtained the template for 12-mer peptide that forms an amphipathic helix upon interaction with the liposome surface. In the next step, we tuned up the hydrophobic side of the helical surface that defines the peptides’ interaction with the hydrophobic core of the membrane. In additional sets of 11- and 12-mer peptides, we replaced all Ala with Ile (**5c** and **7a**) or Leu (**5d** and **8a**), expecting longer aliphatic side chains to improve the antimicrobial activity of helical peptides.^21^ Indeed, these substitutions resulted in a sharp increase in the antibacterial activity of 12-mer peptides with Ile (**7a**) or Leu (**8a**) against all the tested bacterial strains, with MICs dropping to the range of 3.1–25 μg/mL (Table 1).

Similar to the antibacterial activity, peptides **7a** and **8a** showed much higher hemolytic activity than peptide **6a** (Table 1). Taking into account that the bacterial membrane possesses a different net surface charge compared to the mammalian membrane,^14^ we tuned up the cationic surface of the peptides by replacing Arg with Lys (peptides **7b** and **8b**) to improve the peptides’ selectivity by making the peptide–membrane interaction more dependent on lipid composition. While these modifications did not change the peptides’ antibacterial potential, they resulted in an approximately 5-fold decrease in the hemolytic activity of peptides **7b** and **8b** (Table 1). In turn, the change in overall charge/hydrophobicity balance by *N*-terminal acetylation (peptides **6c**, **6d**, **7c**, **7d**, **8c**, and **8d**) resulted in an increase in the hemolytic activity for all peptides as compared to their free *N*-terminal amine analogues (Table 1).

### 2.2 Extended Antibacterial Activity and Cytotoxicity Screening

#### 2.2.1 Broad-Spectrum Antibacterial Activity

In the initial activity screening, the MICs of all synthesized peptides **1a–8d** were determined against five different bacterial strains—two Gram-positive strains (*S. aureus* and methicillin-resistant *S. aureus* (MRSA)) and three Gram-negative strains [*Escherichia coli*, *Pseudomonas aeruginosa*, and *Klebsiella pneumoniae* (Table 1)]. Based on the results of the initial activity screening, we selected peptides **7a**, **7b**, **8a**, and **8b** for tests against an extended spectrum of Gram-positive and Gram-negative bacteria using the ESKAPE panel (*Enterococcus faecium*, *S. aureus*, *K. pneumoniae*, *A. baumannii*, *P. aeruginosa*, and *Enterobacter* species). All selected peptides exhibited activity with MIC = 3.1–12.5 μg/mL (Table 2) against all tested strains of Gram-positive bacteria except *Staphylococcus pneumoniae*. For the nonresistant and MDR *S. pneumoniae*, the peptides showed moderate activity (MIC = 25 μg/mL for **7a**, **7b**, and **8a**; MIC = 12.5 μg/mL for **8b**). Notably, both **8a** and **8b** showed higher activity against vancomycin-resistant *Enterococci* (ATCC 70022 and 51575) than commercially available lipopeptide antibiotic daptomycin. Compared to **7a** and **7b**, peptides **8a** and **8b** demonstrated similar or better activity (MIC = 3.1–12.5 μg/mL) against all the tested strains of Gram-negative bacteria.

**Table 2:** Antibacterial Activity of the Selected Peptides against Drug-Resistant Gram-Positive and Gram-Negative Bacterial Strains

	MIC^g^ (μg/mL)							
bacterial strain	**7a**	**7b**	**8a**	**8b**	daptomycin	vancomycin	ciprofloxacin	polymyxin B
Gram-Positive								
*E. faecium* (ATCC 27270)	6.2	6.2	6.2	3.1	1.5	1.5	ND^h^	ND^h^
a*E. faecium* (ATCC 700221)	6.2	6.2	3.1	3.1	6.2	>50	ND^h^	ND^h^
*E. faecalis* (ATCC 29212)	12.5	12.5	12.5	6.2	6.2	0.7	ND^h^	ND^h^
a*E. faecalis* (ATCC 51575)	12.5	6.2	6.2	6.2	12.5	>50	ND^h^	ND^h^
*S. pneumoniae* (ATCC 49619)	25	25	25	12.5	12.5	3.1	ND^h^	ND^h^
b*S. pneumoniae* (ATCC 700677)	25	25	12.5	12.5	12.5	1.5	ND^h^	ND^h^
*Bacillus subtilis* (ATCC 6633)	6.2	3.1	3.1	3.1	0.7	0.7	ND^h^	ND^h^
*Bacillus cereus* (ATCC 13061)	12.5	6.2	6.2	3.1	1.5	0.7	ND^h^	ND^h^
Gram-Negative								
c*E. coli* (ATCC BAA-2452)	12.5	6.2	12.5	6.2	ND^h^	ND^h^	0.7	0.7
*Klebsiella pneumonia* (ATCC 13883)	25	25	12.5	12.5	ND^h^	ND^h^	1.5	6.2
d*K. pneumonia* (ATCC BAA-2470)	12.5	25	12.5	12.5	ND^h^	ND^h^	0.7	1.5
e*A. baumannii* (ATCC BAA1605)	12.5	12.5	6.2	3.1	ND^h^	ND^h^	0.7	0.7
*P. aeruginosa* (ATCC 10145)	12.5	6.2	6.2	6.2	ND^h^	ND^h^	0.7	0.7
a*P. aeruginosa* (ATCC BAA-1744)	12.5	12.5	12.5	6.2	ND^h^	ND^h^	0.7	0.7

a Vancomycin.

b Multi-drug resistant (penicillin, tetracycline, and erythromycin).

c NDM-1.

d Carbapenem.

e Ciprofloxacin.

^f^Imipenem-resistant bacterial strains.

g Minimum inhibitory concentrations (MIC) were determined as the lowest concentration of the peptides that inhibited bacterial growth.

h ND represents not determined. Results represent the highest MIC observed from three independent experiments performed in triplicate.

We also examined the effect of monovalent (Na^+^, K^+^, and NH_4_^+^) and divalent (Ca^2+^ and Mg^2+^) cations at physiologically relevant concentrations on the activity of the lead peptides (**7a**, **7b**, **8a**, and **8b**) against MRSA and *E. coli*. The results revealed that the peptides either maintained their potency or demonstrated a 2- to 4-fold increase in the MICs in the presence of cationic salts or serum [Table S2 (Supporting Information)].

#### 2.2.2 Hemolytic Activity and Cell Viability

The toxicity of all the synthesized peptides toward human red blood cells (hRBCs) was evaluated using the hemolytic assay by measuring peptide concentrations required for 50% hemolysis (HC_50_ values, Table 1). Overall, peptides having Arg as cationic residues demonstrated higher hemolytic activity than those with Lys. The N-terminal acetylated peptides, in most cases, were found to be more hemolytic than their free N-terminal amine analogues.

**Figure 3:**
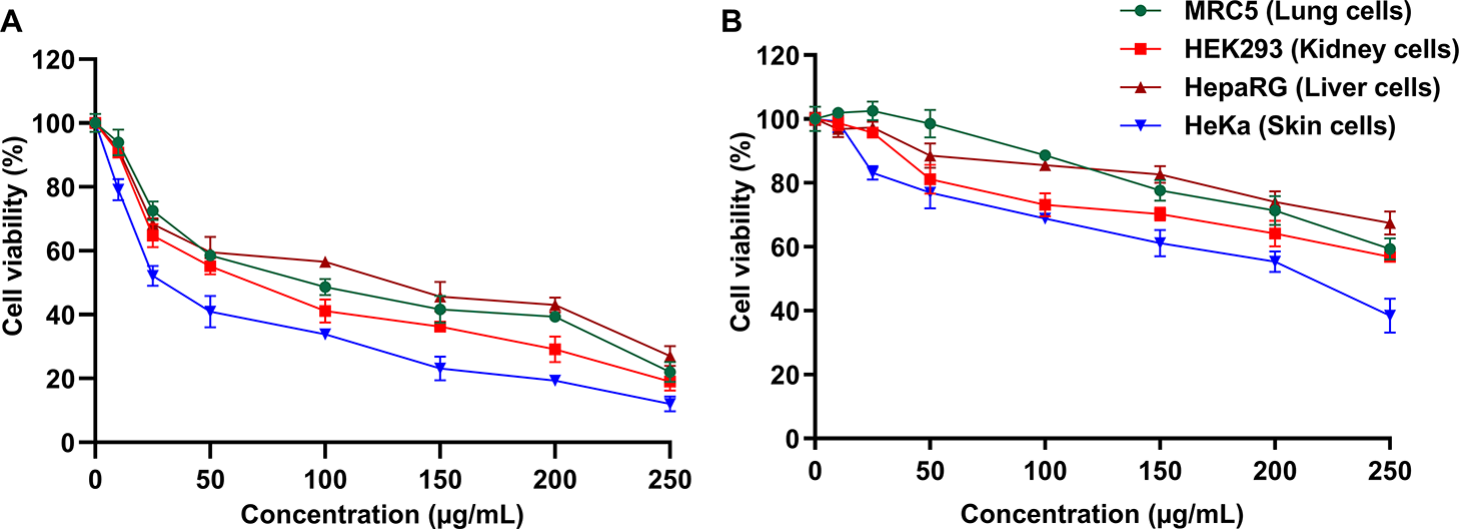
Cytotoxicity of peptides **8a** (A) and **8b** (B). The viability of human lung cells (MRC5, green), human embryonic kidney (HEK293, red), human liver cells (HepaRG, brown), and skin cells (HeKa, blue) is shown as a function of the peptide concentration. The results represent the data obtained from the experiments performed in triplicate (incubation for 24 h).

The cytotoxicity of the lead peptides **8a** and **8b** was further examined by conducting the 24 h cell viability assay using human lung fibroblast cells (MRC-5), human embryonic kidney cells (HEK-293), human hepatic cells (HepaRG), and human skin fibroblast cells (HeKa) (Figure 3). In good agreement with the hemolytic data (Table 1), peptide **8a** was more cytotoxic than peptide **8b**. The viability of all the tested cells dropped below 60% at 50 μg/mL and below 30% at 250 μg/mL of peptide **8a**, while it was above 75% at 50 μg/mL and, except for skin cells, above 60% at 250 μg/mL of peptide **8b**. The cytotoxicity of daptomycin against kidney and liver cells was used as a control. A negligible cytotoxicity was observed even at the highest experimental concentration of 250 μg/mL daptomycin (Figure S6, Supporting Information).

#### 2.2.3 Bactericidal Kinetic Assay

**Figure 4:**
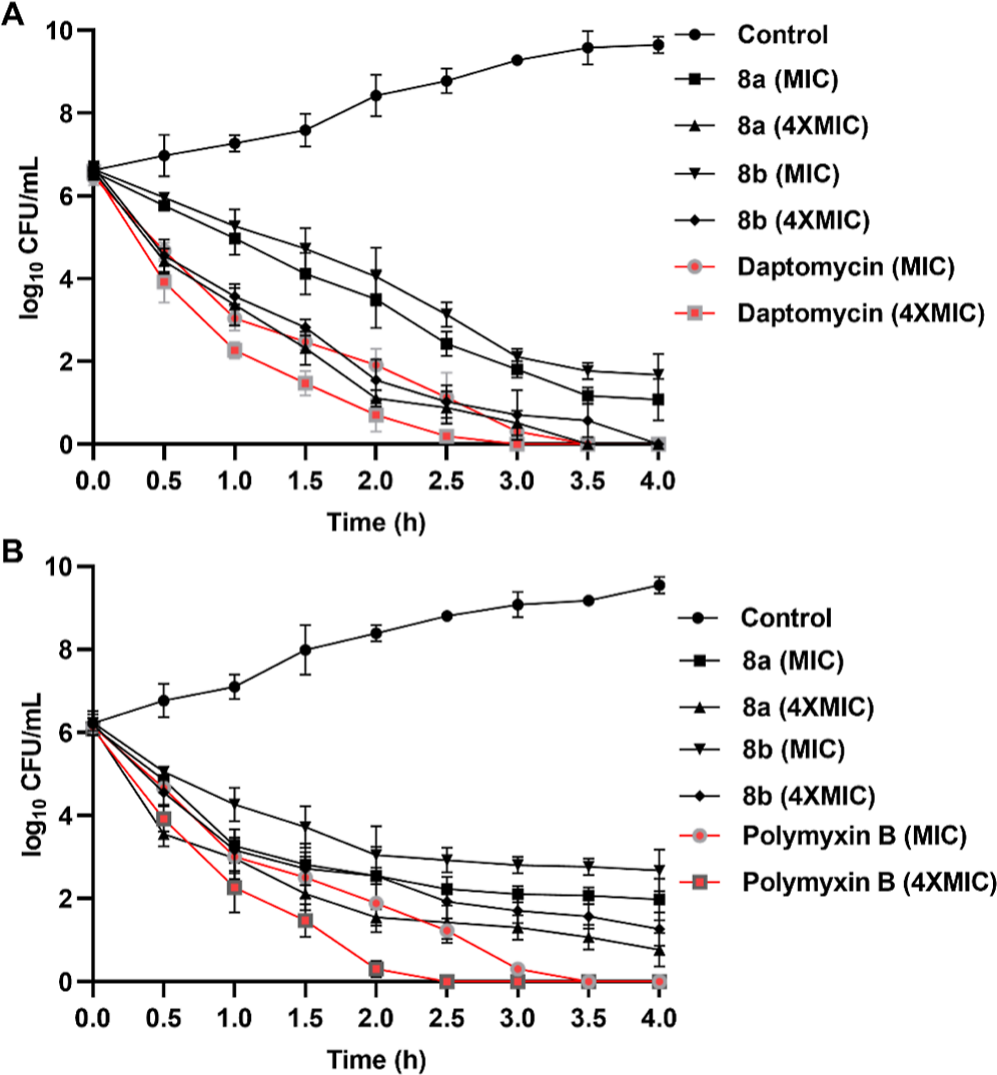
Bactericidal kinetics of test peptides (**8a** and **8b**) and standard antibiotics (daptomycin and polymyxin B) against MRSA (A) and *E. coli* (B) at MIC and 4 × MIC. The data obtained are from the experiments performed in triplicate.

The bactericidal kinetic assay showed time-dependent growth inhibition of both MRSA and *E. coli* by **8a** and **8b** (Figure 4). After 4 h of incubation, both **8a** and **8b** at MIC eradicated approximately 75–80% of the MRSA cells and completely eliminated MRSA cells at 4 × MIC. On the other hand, a comparatively milder action was observed against *E. coli*, as both **8a** and **8b** at 4 × MIC eradicated only about 70–75% of *E. coli* cells even after 4 h of incubation. Daptomycin at 4 × MIC exerted the complete killing of MRSA in 3 h. Similarly, polymyxin B at 4 × MIC completely eradicated *E. coli* cells in 2.5 h (Figure 4).

### 2.3 Mechanistic Studies of the Lead Peptides and Analogues (Experiments and Simulations)

#### 2.3.1 Calcein Dye Leakage Assay

**Figure 5:**
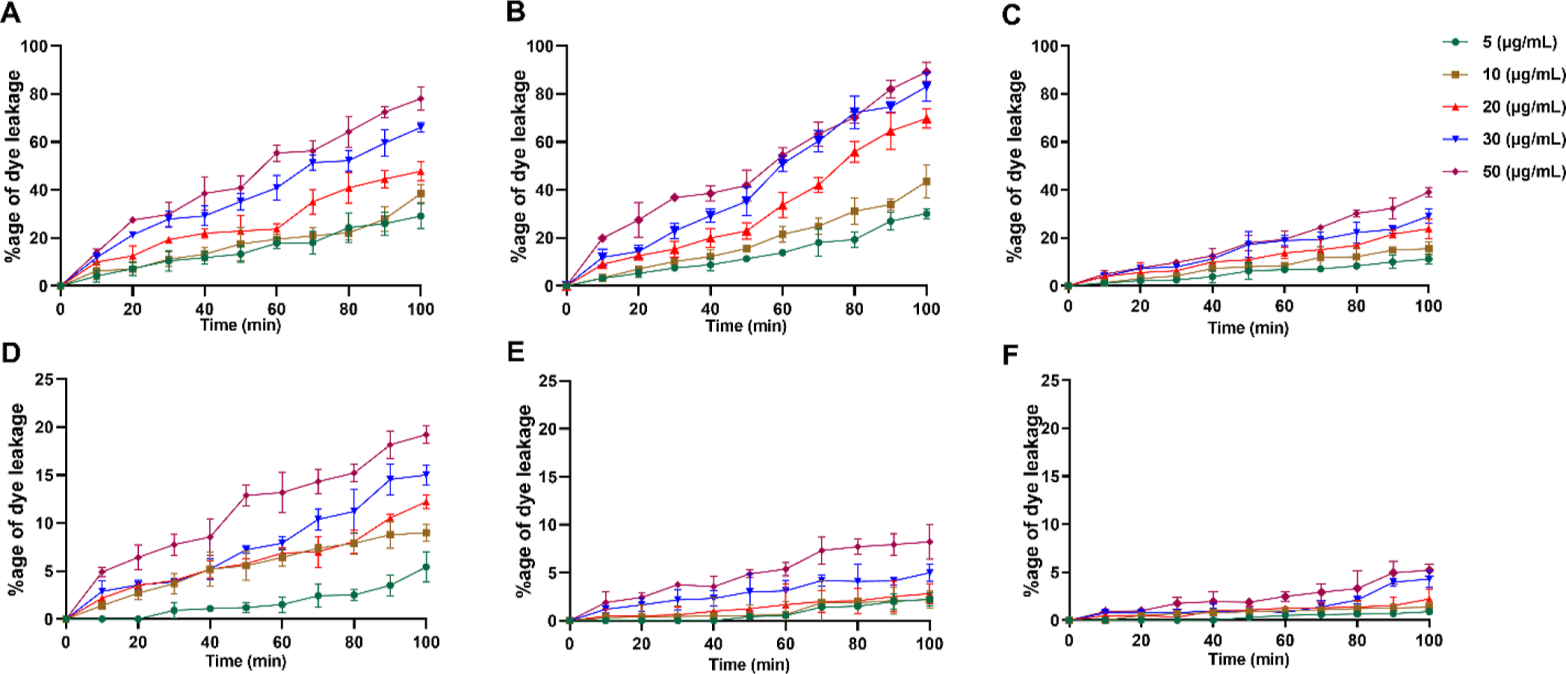
Concentration-dependent leakage of calcein dye from bacterial membrane mimicking [top panels (A–C)] and mammalian membrane mimicking [bottom panels (D–F)] liposomes in the presence of peptides **8a** (A,D), **8b** (B,E), and daptomycin (C,F). The data obtained are from the experiments performed in triplicate.

Upon incubation with the liposomes mimicking a bacterial membrane, both **8a** and **8b** induced a concentration-dependent dye leakage. After 100 min of incubation with **8a** and **8b**, approximately 30% and 80–90% leakage was observed at peptide concentrations 5 and 50 μg/mL, respectively (Figure 5). Daptomycin at 50 μg/mL induced only 42% dye leakage after 100 min of incubation with the same liposomes mimicking the bacterial membrane. In turn, liposomes mimicking the mammalian membrane after 100 min of incubation with 50 μg/mL peptides **8a** and **8b** showed a mild 19 and 10% leakage, respectively, while daptomycin at 50 μg/mL induced a negligible amount of dye leakage (around 7%). Overall, the outcomes of the calcein dye leakage experiments indicated that, like most of the native AMPs, peptides **8a** and **8b** exerted antibacterial action via destabilizing the target bacterial membrane.

#### 2.3.2 Fluorescence Microscopy

**Figure 6:**
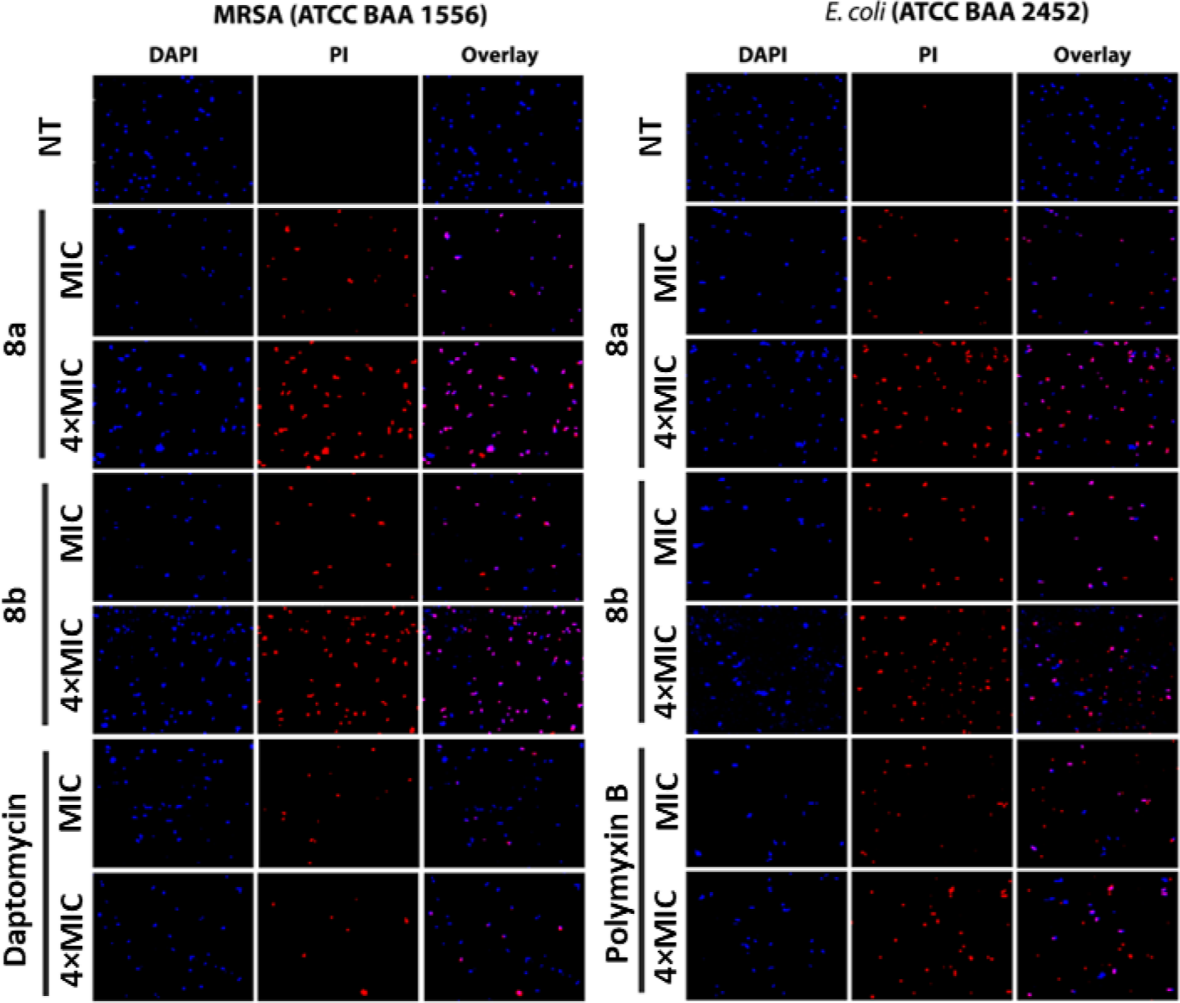
Fluorescence micrographs of DAPI- and PI-stained bacterial cells (MRSA and *E. coli*) treated with test peptides (**8a** and **8b**) and standard antibiotics (daptomycin and polymyxin B) at MIC and 4 × MIC.

To further investigate the membrane perturbation action, the ability of lead peptides **8a** and **8b** to cause membrane damage was assessed by fluorescence microscopy (Figure 6). The concentration-dependent effect of lead peptides on both Gram-positive (MRSA) and Gram-negative (*E. coli*) bacteria was examined by a double staining method using DAPI (4′,6-diamidino-2-phenylindole) and PI (propidium iodide). Following the treatment with test peptides (**8a** and **8b**) at MIC and 4 × MIC for 1 h, bacterial cells were stained with DAPI, which stains the DNA of all bacterial cells in blue irrespective of their viability and PI, which penetrates only injured or dead cells with compromised membranes. In control (without initial treatment with peptides, Figure 6, top row NT), both MRSA and *E. coli* showed blue fluorescence with DAPI. At the same time, a negligible number of cells had red fluorescence with PI. On the other hand, MRSA and *E. coli* cells treated with **8a** and **8b** showed strong red fluorescence with PI. Comparatively higher red fluorescence was observed at 4 × MIC of both peptides for both MRSA and *E. coli*, suggesting the concentration-dependent membrane disruption effect (Figure 6). Interestingly, MRSA and *E. coli* cells treated with daptomycin and polymyxin B, respectively, exhibited lower PI staining than those treated with **8a** and **8b**.

#### 2.3.3 FACS Analysis

**Figure 7:**
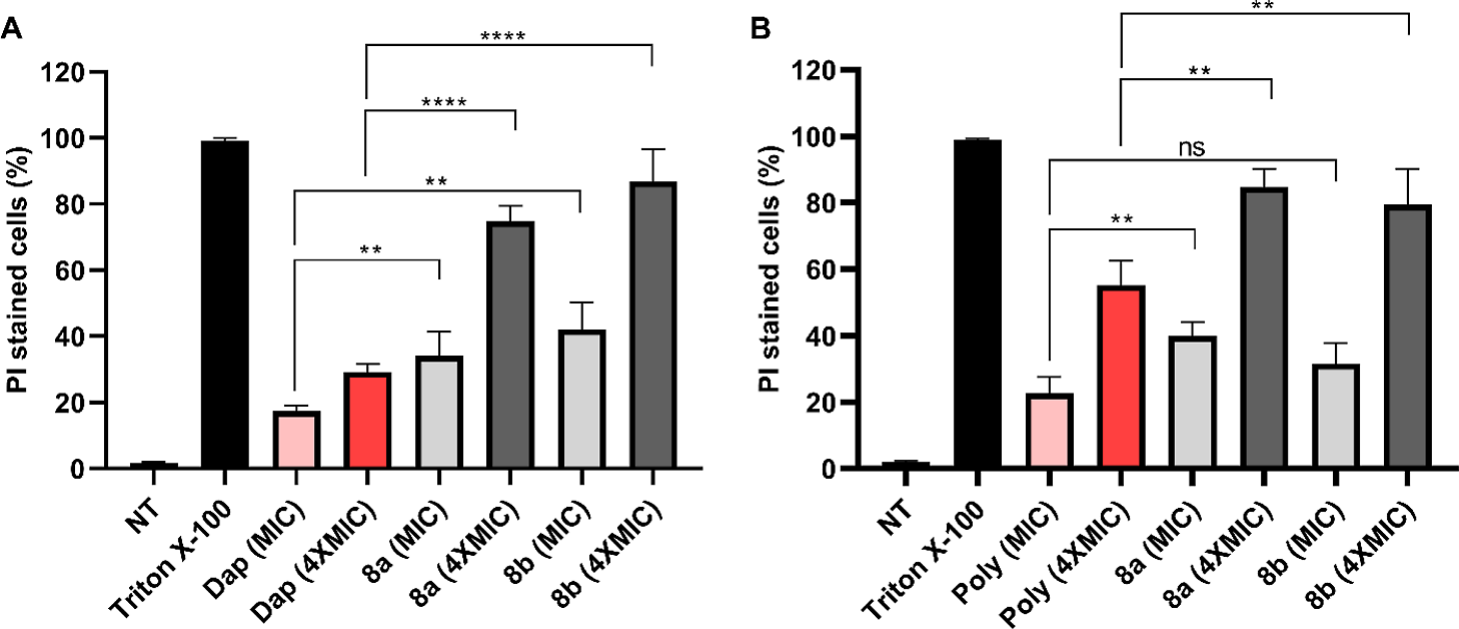
Flow cytometric analysis of MRSA (A) and *E. coli* (B) bacterial cells treated with peptides (**8a** and **8b**) and standard antibiotics [daptomycin (Dap) and polymyxin B (Poly)] at MIC and 4 × MIC. NT corresponds to the negative control—not treated cells in PBS; Triton X-100 represents positive control, cells treated with 10% (v/v) aqueous solution of Triton X-100. The data obtained are from the experiments performed in triplicate. The data were analyzed using a two-tailed unpaired Student’s *t*-test (**p < 0.01, ****p < 0.0001, ns—not significant).

Flow cytometric analysis was performed to quantify the PI-stained bacterial cells (MRSA and *E. coli*) upon treatment with the peptides (**8a** and **8b**) and standard antibiotics (daptomycin and polymyxin B). Bacterial cells treated with PBS or with 10% aqueous triton X-100 (v/v) were used as negative and positive controls, respectively. Around 99% of the bacterial cells treated with PBS demonstrated no PI fluorescence, suggesting that the bacterial cytoplasmic membranes were intact. In contrast, 98% of bacterial cells treated with triton X-100 exhibited PI fluorescence [Figures 7 and S7 (Supporting Information)]. A concentration-dependent increase in fluorescent intensity was observed for **8a** and **8b** (Figure 7). Treatment of MRSA cells with **8a** and **8b** at MIC resulted in a PI fluorescence of 34.2 and 42.1%, respectively. Upon treatment with **8a** and **8b** at a concentration level of 4 × MIC, a sharp increase in the PI-stained MRSA and *E. coli* cells (75–85%) was observed. On the other hand, both daptomycin and polymyxin B treatments did not significantly increase the PI-stained population of MRSA and *E. coli* cells, respectively. These results revealed the predominant membrane disruption capability of **8a** and **8b** compared with the standard antibiotics daptomycin and polymyxin B.

#### 2.3.4 Field-Emission Scanning Electron Microscopy (FE-SEM)

**Figure 8:**
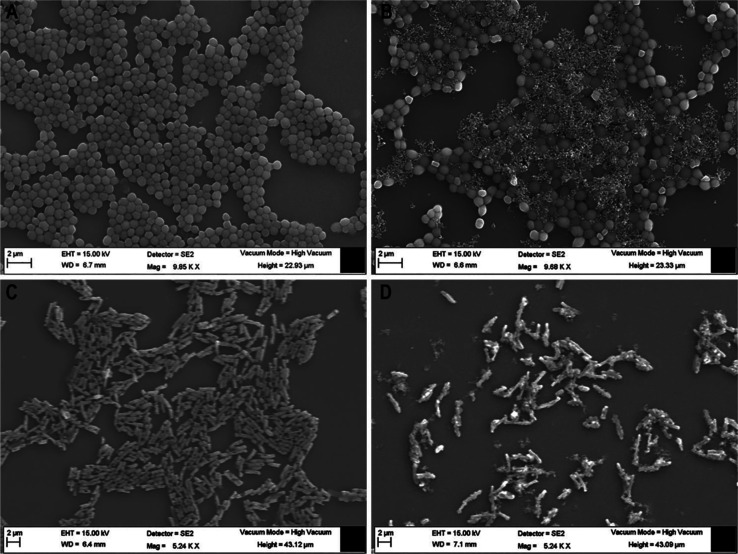
FE-SEM images of MRSA (A,B) and *E. coli* (C,D). Mid-logarithmic-phase bacterial cells were incubated with **8b** (B,D) at a final concentration of 4 × MIC for 1 h. The control images (A,C) were taken without the peptide.

To further investigate the membranolytic behavior of lead peptide **8b**, we visualized the untreated and treated MRSA and *E. coli* cells at an ultra-structural level using FE-SEM. Figure 8A,C illustrates that untreated bacterial cells exhibit regular size and shape with a bright and smooth surface. On the other hand, **8b** caused severe membrane damage to the bacterial cells of both types (Figure 8B,D). A visible disruption in the membrane, along with surface wrinkling, roughening, and cellular debris, can be seen in the case of treated MRSA cells (Figure 8B). Even more prominent morphological changes are evident in the case of *E. coli*, with surface blebs and cellular debris oozing out of the cells (Figure 8D). The results indicate the membrane disruption action of **8b** against both MRSA and *E. coli*.

#### 2.3.5 Role of Overall Polarity/Hydrophobicity on the Peptides’ Membrane Selectivity

**Figure 9:**
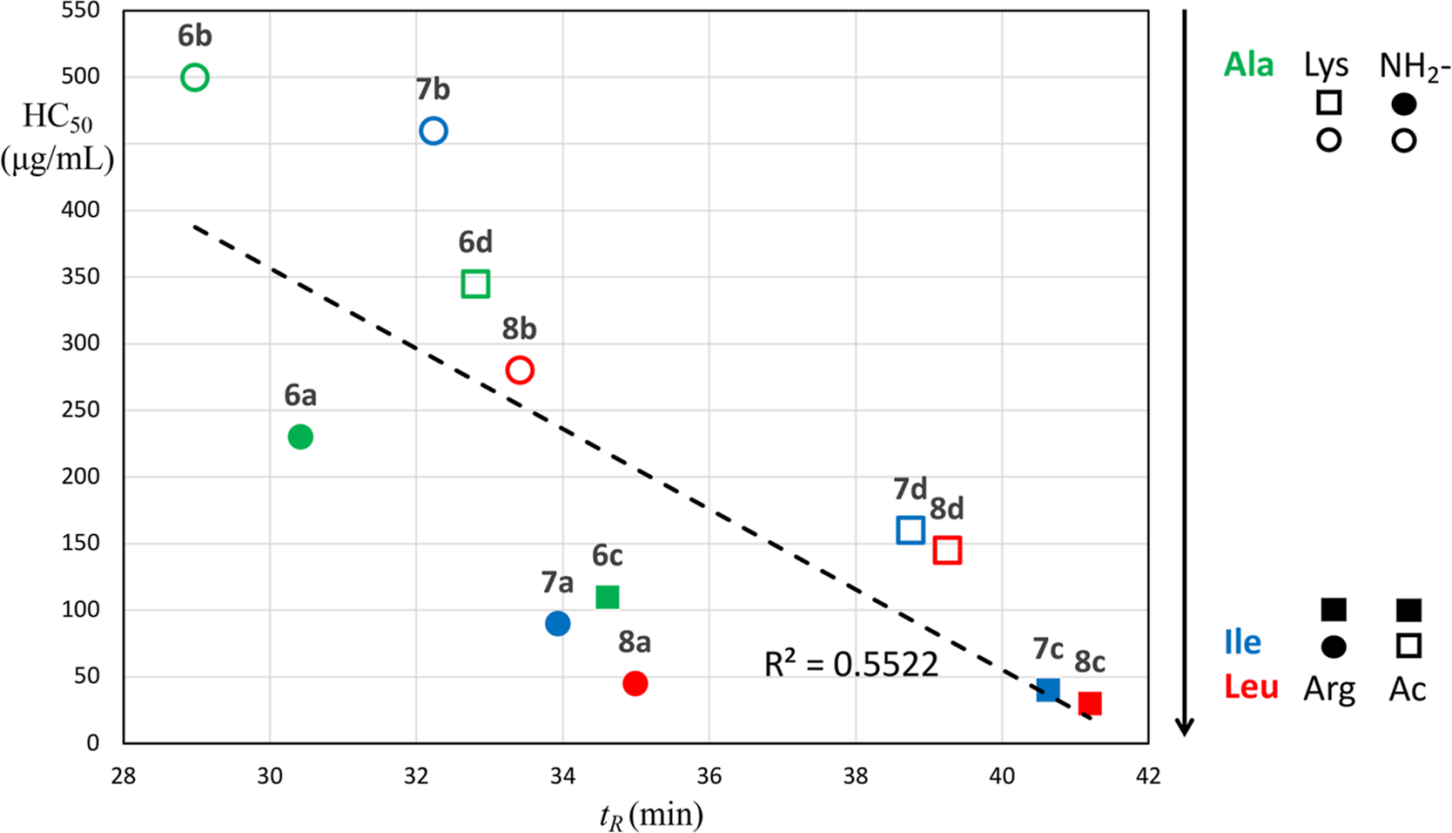
Correlation between hydrophobicity and hemolytic activity. Retention time (*t*_R_) as a factor of overall hydrophobicity of a peptide was determined using RP-HPLC [Table S1 (Supporting Information)]. Hemolytic activity (HC_50_) represents the concentration of the peptide required to exert hemolysis of 50% RBCs (Table 1). The peptides containing specific amino acids are marked as follows: Ala (green), Leu (red), Ile (blue), Arg (filled marks), and Lys (open marks). The N-terminus acetylated peptides are marked by squares, and the peptides with free N-terminus are marked by circles.

The net charge/hydrophobic bulk ratio of AMPs is well known to play a crucial role in driving them toward the bacterial membrane over the mammalian membrane. The RP-HPLC retention time can be considered as the overall measure of a molecule’s net charge–hydrophobicity balance. As expected, the retention times show that the amino acids contribute toward the overall hydrophobicity of the peptides in the following order: Leu > Ile > Ala and Arg > Lys. In addition, significantly higher retention times were observed for the N-terminal acetylated peptides (**6c**, **6d**, **7c**, **7d**, **8c**, and **8d**) as compared to their free N-terminal counterparts (**6a**, **6b**, **7a**, **7b**, **8a**, and **8b**) (Table S1). In agreement with the previous reports,^22,23^ longer retention time and higher hydrophobicity correlate with higher hemolytic activity (Figure 9).

#### 2.3.6 Circular Dichroism

The peptide secondary structure induced by the interaction with a cell membrane is one of the key features which defines the antibacterial activity of amphiphilic AMPs. The changes in the secondary structure of the peptides were evaluated by CD spectroscopy in PBS, 50% trifluoroethanol (TFE), and in the presence of liposomes mimicking a bacterial membrane. While the prediction of helicity from the CD spectra can be very inaccurate for short peptides,^24^ the changes in spectral intensity at 208 and 222 nm for the same peptide in different conditions could be used to monitor the conformational changes^25^ and particularly the changes in the peptides’ helicity. The CD spectra of 7-mer (**1c**) and 8-mer (**2a**) peptides did not show helicity in any of the used media. The spectra of 9-mer (**3e**), 10-mer (**4f**), and 11-mer (**5d**) peptides showed some helicity in 50% TFE, but no considerable helicity in PBS or in the presence of the liposomes [Figure S2 and Table S1 (Supporting Information)]. In turn, the CD analysis for all 12-mer peptides revealed spectral features characteristic of a helical conformation (two minima at 208 and 222 nm) in 50% TFE and with the liposomes, while in PBS, very low, if any, of these features were detected [Figures 2 and S3–S5 and Table S1 (Supporting Information)]. As shown in Figure 2 (right panel), the lipid environment induced a more pronounced shift toward helical structure than 50% TFE for all tested 12-mer peptides having high antibacterial activity (**7a–d** an **8a–e**).

#### 2.3.7 NMR Spectroscopy

For the selected lead peptide **8b** and its close analogues (**6b** and **8a**), we evaluated the secondary structure in water and in the presence of liposomes mimicking bacterial or mammalian membranes. We used a standard NOE-based approach to identify the conformation of the peptide backbone and localize elements of secondary structure (turns and helices), if any. In short, the evaluation was based on identified NOE contacts between the backbone (H^N^, H^α^) and side-chain (mostly H^β^) atoms. Thus, an extended backbone conformation is characterized by strong NOE contacts between H^N^ and preceding H^α^ atoms (Hi^N^/H_i-1_^α^), weak contacts between H^N^ and preceding H^β^ atoms (Hi^N^/Hi-1^β^), and missing continuous H^N^/H^N^ contacts. In turn, a continuous pattern of H^N^/H^N^ contacts, weaker H_i_^N^/H_i-1_^α^ and stronger H_i_^N^/H_i-1_^β^ contacts, testify for the regions with short turns or helices.^26^ Intermolecular NOE contacts were used to localize interactions between peptide and lipid groups.

**Figure 10:**
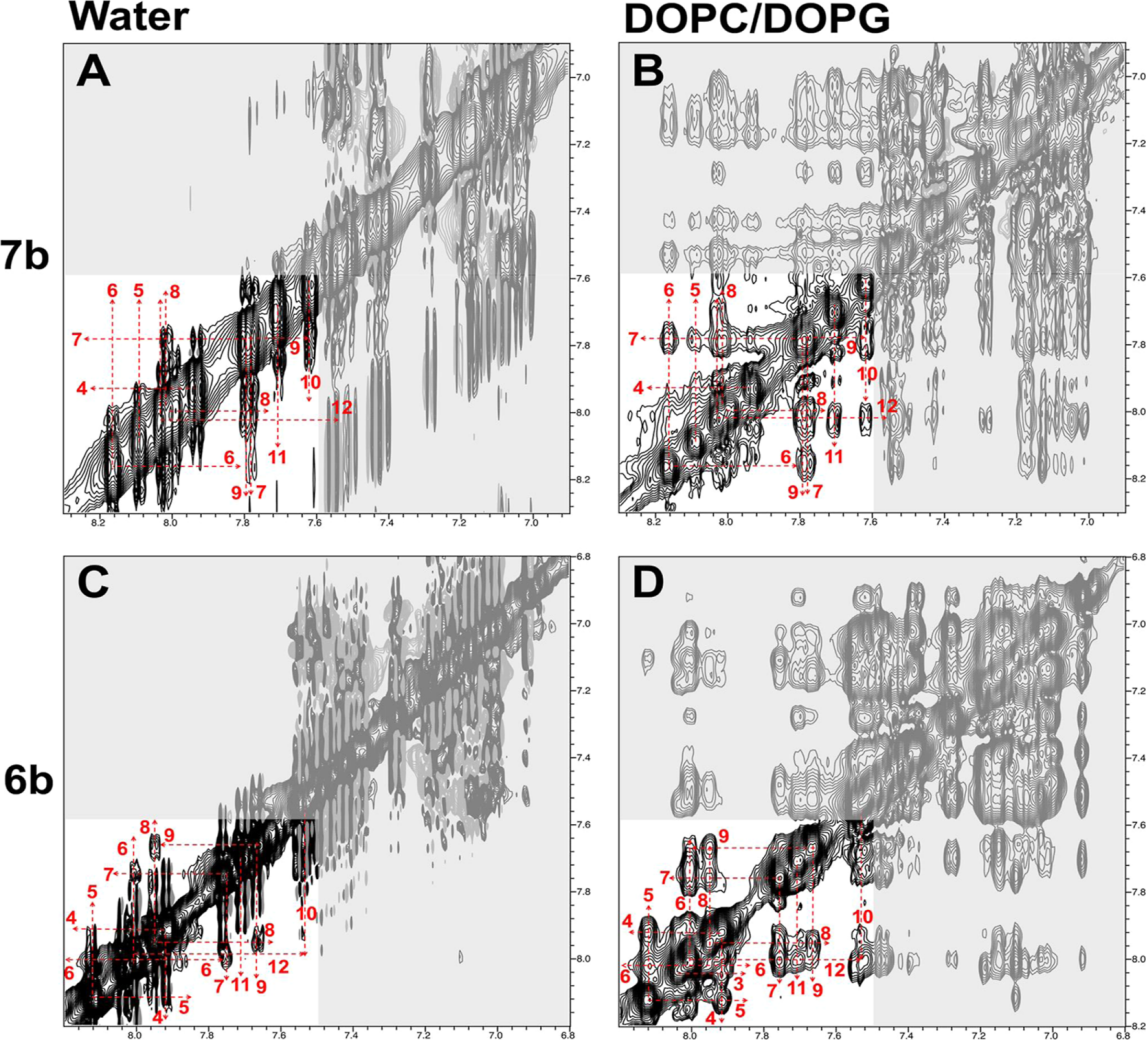
Amide regions of NOESY spectra of peptides **7b** [top row (A,B)] and **6b** [bottom row (C,D)] in water [left (A,C)] and mixed DOPC/DOPG liposomes [right (B,D)]. The assignment of the H^N^ resonances is shown with the dashed lines and the residue label. H^N^ signal for Trp2 is located outside of the shown area. The gray hatched areas contain signals from the Trp aromatic groups.

**Figure 11:**
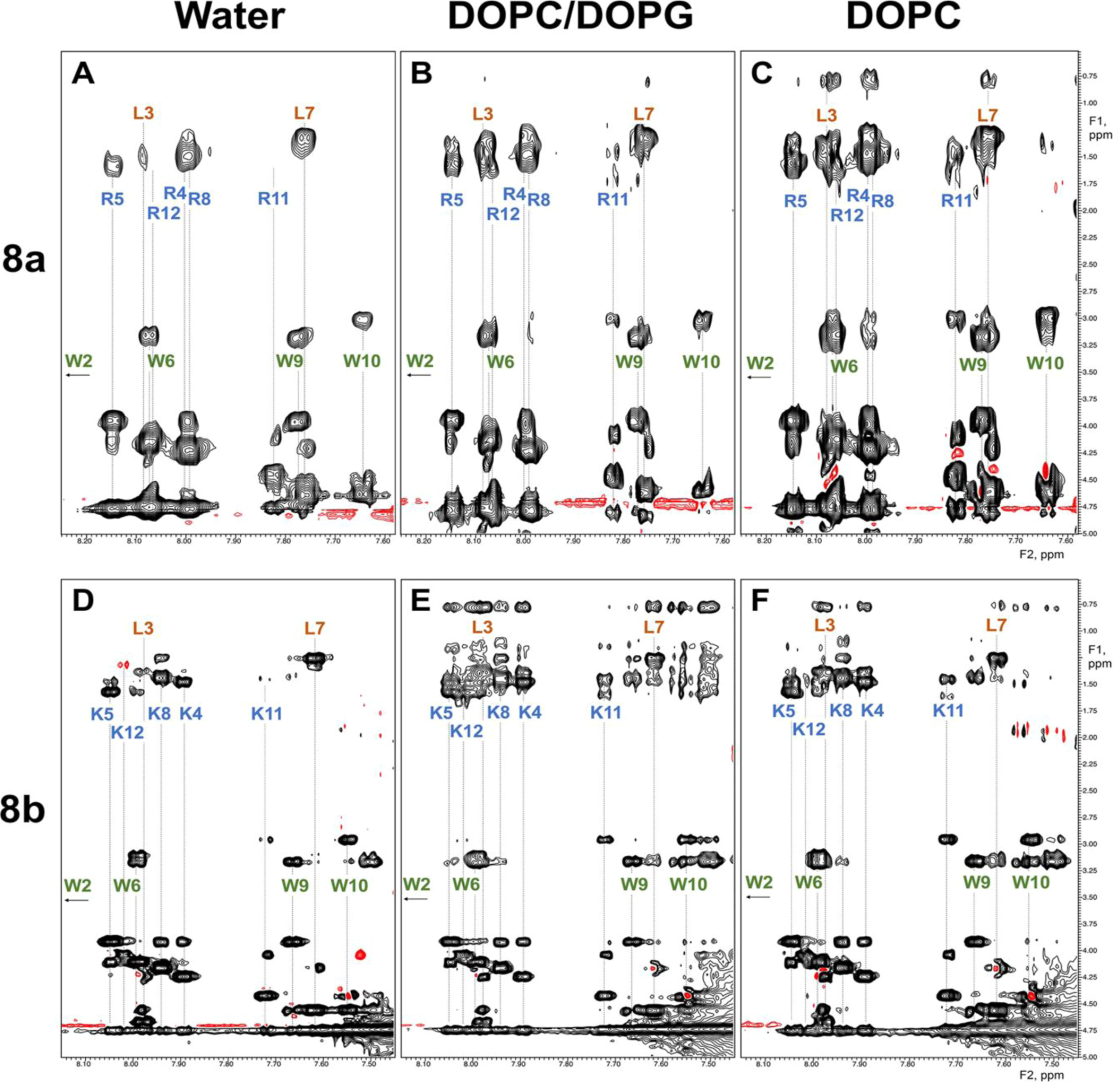
Amide/aliphatic regions of NOESY spectra of peptides **8a** [top row: panels (A–C)] and **8b** [bottom row: panels (D–F)] in water [left: panels (A,D)], mixed DOPC/DOPG liposomes [center: panels (B,E)], and DOPG liposomes [right: panels (C,F)]. The assignment of the H^N^ resonances is shown with the vertical dashed lines and residue label. H^N^ signal for Trp2 is located outside of the shown area.

In general, the NOE spectra for all tested 12-mer peptides in water demonstrate weak NOE, both positive and negative, in accordance with the molecular weight of these monomeric peptides (1.6–1.9 kDa). In agreement with the CD results, NMR data for the peptides containing Leu (**8a** and **8b**) or Ile (**7b**) showed that in water they have predominantly extended, unordered conformation with an indication of a short transient turn formed by residues 7–9 (Figures 10 and 11). In water, the peptide with Ala (**6b**) has a slightly longer full helical turn (residues 6–10, Figure 10C). The H^N^–H^α^ coupling constants for all tested peptides in water are within the range of 6.2–6.8 Hz representing the unordered conformation of the backbone that can be described as a mixture of different short-living backbone conformations, including helical turns (coupling constants below 5 Hz) and extended conformations (coupling constants above 8 Hz).

The addition of the liposomes’ mimicking bacterial membrane (DOPC/DOPG) resulted in substantial changes in the NOE spectra of all tested peptides. The intensity of all NOE cross-peaks increased substantially, reflecting a significant increase in the correlation time and mass of the peptide–lipid particles compared with the monomeric peptide in water, thus confirming the strong interaction of the tested peptides **6b**, **7b**, **8a**, and **8b** with lipids (Figures 10 and 11). However, the changes in NOE patterns were more peptide and lipid specific. Particularly, for the peptides **8b, 7b**, and **6b** in DOPC/DOPG mixture, continuous H^N^/H^N^ contacts (Figure 10) and a rise in intensities of H_i_^N^/H_i-1_^β^ contacts (Figure 11) demonstrate increased helicity for the residues 4–12. Additionally, multiple intra-residue NOEs were detected between the amide hydrogens and the side-chain methyl groups of Leu (**8b**, Figure 11E) and Ile (**7b**, data not shown) residues as well as between amide hydrogens and aromatic groups of Trp in all tested peptides (Figure 10, panels B and D show examples for **6b** and **7b**). The high-intensity NOE contacts along the side chains indicate restricted mobility of these bulky moieties that, in turn, could be the direct consequence of a stable helical structure and strong interactions with the lipid phase.

Interestingly, peptide **8b**, with Lys as cationic residues, has no similar network of backbone-methyl NOEs in the DOPC liposomes, mimicking the mammalian membrane. According to the overall NOE intensities, the peptides with Lys (**6b** and **8b**) interact with the DOPC liposomes much weaker than with the mixed DOPC/DOPG liposomes (Figure 11, panels E and F). At the same time, the NOE contacts for peptide **8a** show an opposite dependence on the lipid composition, with stronger NOEs in the presence of DOPC liposomes than in the presence of mixed DOPC/DOPG liposomes (Figure 11, panels B and C). Thus, in accordance with cytotoxicity and calcein dye leakage assay results, the NOE data revealed that the peptide containing Arg (**8a**) interacts with the liposomes mimicking the mammalian membrane’s composition much stronger than the peptide containing Lys (**8b**).

While the presence of lipids evidently affected the intensities and the whole pattern of NOE contacts, the effect of lipids on chemical shifts and spin–spin coupling of the peptide ^1^H-signals was minimal for all tested peptides. This fact can be explained by the dynamic averaging of NMR parameters between the lipid-bound and free peptide states. Because of the high molecular weight of the liposomes, tightly bound peptide molecules are “invisible” in our NMR experiments; however, long-lasting magnetization acquired by the peptides in the lipid-bound state and detected after dissociation in the free state provides a strong enhancement to the NOE effect and represents peptide conformation in the lipid-bound state.^27^ The high peptide/lipid molar ratio (1:1) used in NMR experiments was selected to minimize signal broadening and to make the free state of the peptides prevalent in all samples for easy detection of the transferred NOEs.

The conservative chemical shifts and spin–spin coupling provide another valuable observation from the NMR data related to the effect of the peptides on liposome stability in our model experiments. The data do not support the interaction of lipids as single molecules with the peptides or such interactions are negligible. While we cannot exclude the appearance of massive irregular lipid aggregates upon interaction with the peptides, these particles are very unstable in an aqueous solution unless they attract and tightly bind other amphiphilic molecules to shield the hydrophobic surface areas. Since we do not detect any decrease in NMR signal intensity for the peptides in the samples with the liposomes, such destabilization of the liposomes at a given peptide/lipid molar ratio in our model experiments seems very unlikely. More focused study with different peptide/lipid molar ratios would help to understand the details of peptide action on the membrane bilayer.

The strong overlap in the aliphatic region of the ^1^H NMR spectra of peptides **7b**, **8a**, and **8b** hampers the detection of the peptide/lipid contacts. While we carefully examined the less crowded methyl region of the spectra of peptide **6b**, we cannot identify any contacts between the peptide and aliphatic groups of the lipids. The spectra of peptide **7b** with mixed DOPC/DOPG liposomes show a good separation of the peptide signals with the methyl signals of aliphatic lipid chains. Using this opportunity, we were able to unambiguously identify a set of NOE contacts between the aromatic side chain of Trp9 (Hζ2, Hζ3, and Hη2 atoms) and aliphatic methyl groups of the lipids. Interestingly, these contacts are specific to Trp9 aromatic atoms only; no contacts to other Trp side chains were detected univocally. There are multiple similar contacts in the NOESY spectra of the peptides **8a** and **8b** in the presence of DOPC/DOPG liposomes; however, these contacts have possible intramolecular assignments. Other potential peptide/lipid contacts are also hidden due to the overlap of the signals from aliphatic lipid chains and long side chains of the peptides. Also, strong signals from the choline group of DOPC overlapped with the H_2_^β^ signals of Trp. The analysis of the NOESY spectra of peptides **8a** and **8b** with DOPC liposomes did not reveal unambiguous intermolecular peptide–DOPC contacts. An additional study utilizing hydrophobic paramagnetic probes or selectively deuterated and/or ^13^C-labeled peptides or lipids might be necessary to identify the intramolecular contacts unambiguously in the heavily overlapped regions.

#### 2.3.8 Molecular Dynamics Simulations of the Lead Peptides and Their Analogues with Distinct Antimicrobial and Hemolytic Activities

For the theoretical investigations, we chose a pair of peptides (**6b/8b**) with low/high antimicrobial activity as well as a homologous Arg-containing peptide **8a** which, along with antimicrobial activity, demonstrates high cytotoxicity. A number of all-atom MD simulations were performed to shed light on the details of peptide–membrane interactions that may be responsible for the different biological activities of the peptides. The bacterial or mammal cell membranes were modeled either by two-component DOPC/DOPG or by pure DOPC bilayers, respectively. These bilayers are similar in composition to the lipid vesicles and liposomes used in our NMR, CD, and dye leakage experiments.

**Figure 12:**
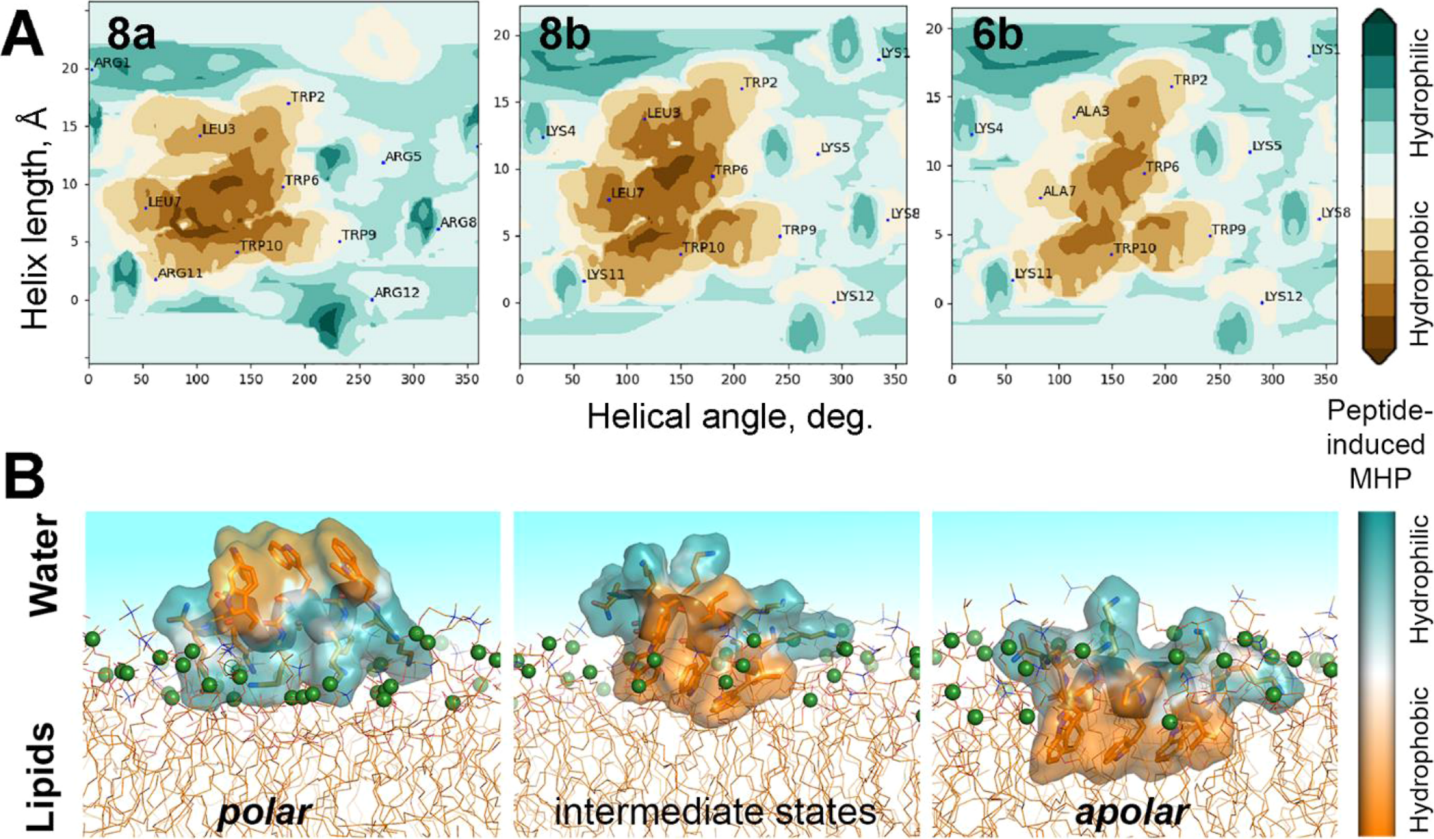
Molecular hydrophobicity potential (MHP) and principal binding modes of the lead peptides. (A) MHP maps for the peptides **8a** (left), **8b** (center), and **6b** (right) in α-helical conformation. The maps are plotted in cylindrical coordinates [rotation angle around the helix axis and *z*-distance—shift along the helix axis (*Z*)]. Peptide-induced MHP values on the peptide molecular surface are color-coded according to the scale on the right. Projections of centers of mass of side-chain atoms of residues are labeled. The maps demonstrate similar locations of charged and hydrophobic groups on the peptide surfaces and different amplitudes of the MHP for the hydrophobic pattern. (B) Two principal membrane binding modes were detected in a series of independent 200 ns MD simulations of the peptides **6b**, **8a**, and **8b** in DOPC/DOPG or DOPC bilayers. MD snapshots are given for peptide **8b** in the DOPC/DOPG bilayer. The peptide surface is color-coded according to the peptide MHP.^28^ The MHP shows the level of complementarity of the amphiphilic peptide surface and the water–lipid (polar/nonpolar) environment in different peptide insertion modes. The backbone and side-chain atoms of the peptide are shown in ribbon and stick representation, respectively. Phosphate groups of lipids are given as green spheres. Only part of the lipid bilayer nearest to the peptide is shown. Water molecules are omitted for clarity, and the water region is indicated with a blue background.

Based on the NMR and CD data on the helical structure of peptides **6b** and **8b** in the lipid environment, the initial conformations of all modeled peptides were constructed to be α-helices with distinct amphiphilic surfaces. As shown in Figure 12A, the polar pattern is formed by positively charged Lys (**6b** and **8b**) or Arg (**8a**) residues. The core part of the apolar surface is composed of aromatic Trp residues in positions 2, 6, 9, and 10. The presence of Leu in **8b** and **8a** instead of Ala in **6b** at positions 3 and 7 makes the corresponding hydrophobic clusters on the molecular surface of peptides **8b**/**8a** much more pronounced and larger in size.

**Figure 13:**
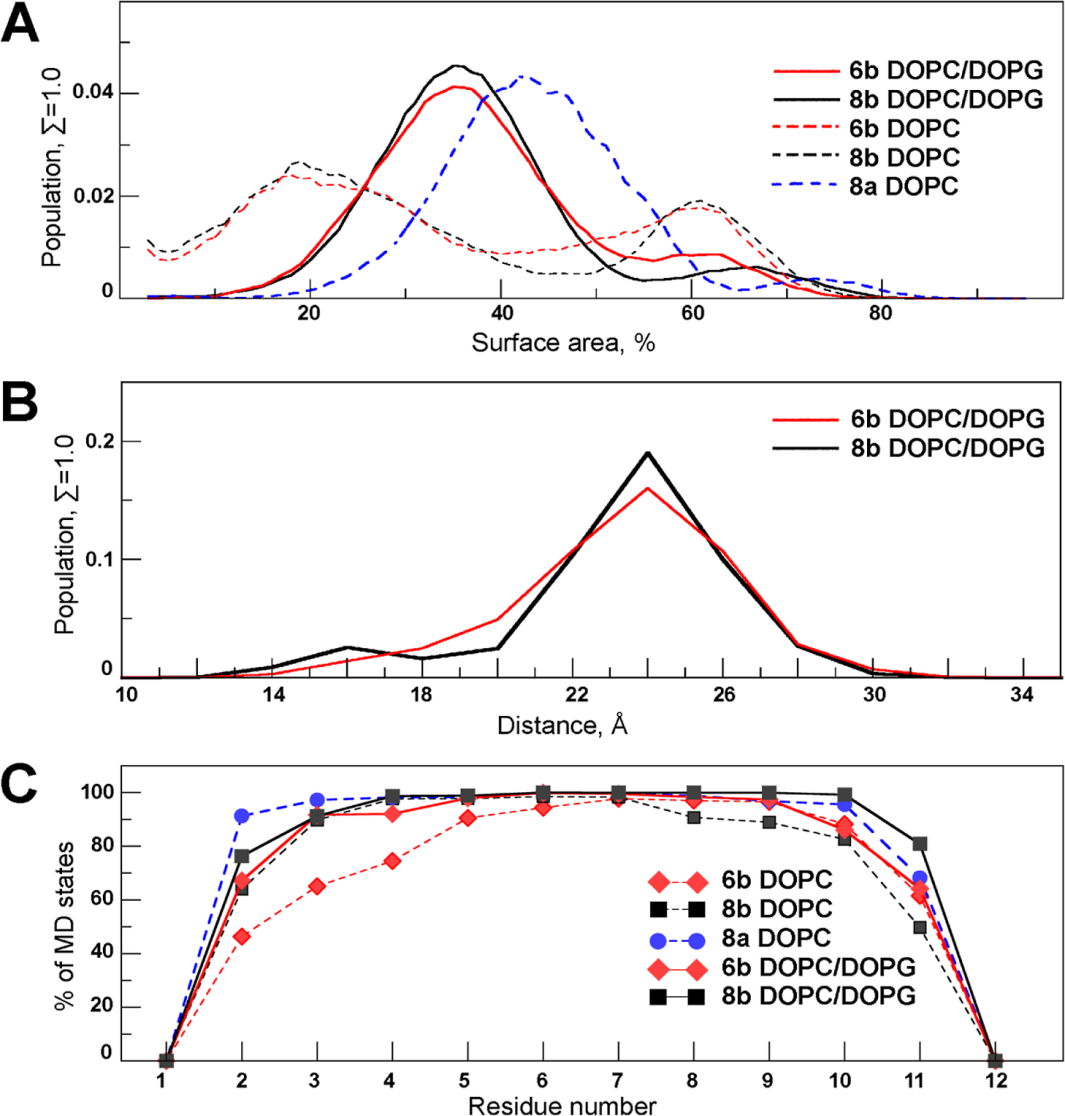
Summary of MD simulation results for peptides **6b**, **8a**, and **8b**. (A) Distributions of the fraction of peptide surface in contact with DOPC/DOPG (solid line) and DOPC (dashed line) membranes. (B) Distributions of the distances between the peptide center of mass and bilayer center calculated over all states of MD runs for peptides **6b** and **8b**. In panels (A,B), the data for peptides **8a**, **8b**, and **6b** are shown with blue, black, and red lines, respectively. (C) Fractions of MD states of the peptides in helical conformation at a given residue. Curves are drawn and colored according to the legend.

In all MD simulations, we observed the binding of the peptides to the model membranes; however, all tested peptides were found at multiple different stages of incorporation into the bilayers. Typically, in the most populated state in the course of 200 ns MD runs, peptide molecules interact with polar head groups of bilayer by their charged residues while keeping the hydrophobic surface away from the bilayer (“*polar*” mode in Figure 12B). The *polar* mode is characterized by relatively small contact areas and large distances between the peptide center of the mass and the center of a bilayer (main distribution peaks in Figure 13A,B, respectively). In the *apolar* mode, the hydrophobic surface of the peptide helix faces the bilayer and interacts with nonpolar or weakly polar regions of the membrane. In contrast to the peripheral interaction in the *polar* mode, in the “*apolar*” mode, the peptide molecules are deeply inserted into the membrane: more than 60% of the peptide molecular surface is in contact with membrane lipids (Figure 13A), and the center of mass of the peptides is closer to the membrane center by ca. 8 Å (Figure 13B). Moreover, the insertion depth into the DOPC/DOPG bilayer is notably smaller for the less-active peptide **6b** than for the active peptide **8b** (Figure 13B). Figure 13A,B shows that the *apolar* mode is poorly populated in MD simulations of all peptides in both model bilayers. Out of 30 MD starts performed for each peptide, a spontaneous insertion of all hydrophobic residues into the DOPC/DOPG membrane was observed in three MD runs for peptide **8b** and only in one MD trajectory for peptide **6b**. Both *polar* and *apolar* interaction modes are stabilized by multiple salt bridges and hydrogen bonds between all modeled peptides and lipids (Table 3).

**Table 3:** Structural Characteristics of Principal Membrane-Bound States of the Peptides^a^

peptide	bilayer	mode	diCM^b^ (Å)	*S*_bured_^c^ (%)	helical^d^	Phob^e^	Es^f^	HBond^g^
**6b**	DOPC/DOPG		16.7 ± *0.9*	67 ± *5*	8.7 ± *0.6*	74.3 ± *6.3*	5.5 ± *1.3*	8.6 ± *2.3*
**8b**			16.7 ± *1.2*	68 ± *5*	9.2 ± *0.8*	76.2 ± *6.4*	6.4 ± *1.9*	9.1 ± *2.4*
**6b**		*apolar*	17.6 ± *0.9*	65 ± *5*	8.4 ± *2.0*	70.3 ± *6.0*	5.2 ± *1.7*	6.9 ± *2.1*
**8a**	DOPC		17.2 ± *1.2*	74 ± *4*	9.9 ± *0.3*	77.1 ± *5.1*	9.6 ± *2.0*	13.4 ± *3.6*
**8b**			17.5 ± *1.2*	65 ± *5*	9.0 ± *1.1*	73.0 ± *5.9*	5.1 ± *1.7*	7.1 ± *1.9*
**6b**	DOPC/DOPG		24.9 ± *1.5*	38 ± *5*	9.3 ± *1.1*	46.4 ± *7.0*	7.7 ± *2.1*	7.9 ± *2.2*
**8b**			24.7 ± *1.5*	38 ± *5*	9.6 ± *0.8*	47.7 ± *6.8*	7.9 ± *2.1*	8.2 ± *2.3*
**6b**		*polar*	25.5 ± *1.5*	38 ± *6*	8.0*±1.7*	44.6 ± *7.1*	3.2 ± *1.8*	4.5 ± *2.2*
**8a**	DOPC		24.9 ± *1.7*	43 ± *7*	9.4 ± *1.0*	46.4 ± *7.1*	9.2 ± *2.4*	12.2 ± *3.6*
**8b**			25.7 ± *1.7*	37 ± *6*	8.5 ± *2.2*	43.8 ± *6.9*	3.7 ± *1.9*	4.7 ± *2.0*

a Data (average values ± standard deviations) were given by averaging over all MD states where the peptide inserts in “*apolar*” or “*polar*” modes.

b Distance between the center of mass of the peptide and the center of the bilayer.

c Percent of the molecular surface of the peptide that is in contact with lipid molecules.

d Number of residues in a helical conformation.

e Number of hydrophobic (Phob) contacts between the peptide and heavy lipid atoms at a distance of 7 Å or less.

f Number of electrostatic (Es) contacts between peptide and heavy lipid atoms at a distance of 6 Å or less.

g Number of peptide–lipid hydrogen bonds.

Interestingly, an association of Lys-containing peptides **6b** and **8b** with the zwitterionic DOPC bilayer is weaker than with the negatively charged bilayer. As can be seen in Figure 13A, a large cluster of weakly bound peptides makes the distribution of contact areas drastically different from that observed for the same peptides with the DOPC/DOPG bilayer. In turn, the Arg-containing peptide **8a** strongly binds to the DOPC membrane, and no dissociation events are observed (Figure 13A).

#### 2.3.9 Assessment of the Peptide Helicity by MD Simulations

The stable helical structure in the central part of the peptides (residues 3–10) was observed in more than 80% of the MD states in both model bilayers, with the only exception of the peptide **6b** in the presence of DOPC (Figure 13C and Table 3). As also shown by the CD and NMR data, these peptides adopt a helical conformation in the presence of DOPC/DOPG liposomes, while in a water environment, they form only a short turn and are primarily unstructured. In turn, the Lys-containing peptides **6b** and **8b** had less-stable helices (up to full destruction) upon interaction with the DOPC membrane as compared to the mixed DOPC/DOPG bilayer. Interestingly, the process of peptide incorporation into the membrane by the hydrophobic side chains was often accompanied by destabilization of the helix in peptides **6b** and **8b** and, in a number of MD simulations, resulted in limited incorporation of the hydrophobic motif (defined as “intermediate” states in Figure 12B). At the same time, Arg-containing peptide **8a** with high hemolytic activity shows a very stable helix during all MD simulations. The main factor of such conformational stability is multiple energetically favorable interactions between the cationic Arg side chains and phosphate groups of lipids. As a result, twice as many of the corresponding H-bonds and stable electrostatic interactions were found for the membrane-bound peptide **8a** compared to Lys-containing **6b** and **8b** (Table 3). Overall, for both *polar* and *apolar* principal membrane binding modes, the average helical content (the number of residues in helical conformation) for the most active peptides **8b** in DOPC/DOPG and **8a** in DOPC are higher than for the low-active analogue **6b** in both bilayers (Table 3).

## 3 Discussion

Studies on various naturally occurring AMPs such as melittin,^29^ magainins,^30^ and cecropin^31^ convincingly demonstrated that the peptides’ ability to adopt a helical conformation with well-defined amphipathicity is critical for their antimicrobial action. However, the comparatively large molecular size of AMPs leads to poor pharmacokinetic properties and hampers the clinical development of many promising candidates.^6,13^ In recent years, progress in our understanding of the structure–activity relationship of AMPs resulted in the development of many synthetic cationic AMPs.^23,32^

Still, the proposed molecular mechanisms of AMP’s action on cell membranes are mostly hypothetical, and the factors determining the antibacterial and hemolytic activity of the peptides are not fully identified. The experimental approaches provide information about the level of antimicrobial activity and cytotoxicity but only limited data about the structural characteristics of the peptides (e.g., their overall secondary structure and its localization, contacts with a membrane-like environment, the effect of amino acid substitutions on the structure and membrane contacts, etc.). These data are insufficient to answer the aforementioned question about the detailed mechanism of action of AMPs. The two key problems are the following: (1) the lack or very limited data on the dynamics (or time-wise behavior) of the systems under consideration—in the case of peptide–membrane interactions; this aspect is of high importance; (2) the complexity of the “membrane response”—reaction of the lipid bilayer to the peptide insertion—and inadequate experimental methods to analyze this effect. The molecular modeling methods, in particular, MD, could help in this situation—they allow deciphering the quantitative relationships of the “structure–dynamics–activity” of AMPs in the presence of membranes of various compositions. In turn, the principal disadvantages of the in silico methods are (1′) their purely empirical nature and, consequently, the need for careful calibration based on experimental data and (2′) very often, MD data are fragmentary and suffer the “sampling problem” due to the insufficient statistics of the analysis of the complex peptide–membrane systems. It should be noted that in order to achieve the goal of this work—to explain the differences in activity observed in the experiment for peptides very similar in physicochemical characteristics (substitutions with amino acids similar in properties)—atomistic MD modeling and accumulation of a representative ensemble of peptide–membrane states are required. The existing rapid simplified methods of screening peptides for their membrane activity (including coarse-grained techniques) are inappropriate for such a task.

Based on the above discussion, the most effective way to solve the key problems is the consistent combination of experimental and computational methods. This approach is well known,^33^ but the attempts to comprehensively analyze the behavior of AMP in membranes often still do not solve (or do not completely solve) the problems formulated above either due to gaps in the experimental evidence or due to insufficient sampling in the computational analysis. In our previous work,^18^ we proposed a comprehensive—experimental and theoretical—approach that was successfully applied to determine the membrane interaction of the lead cyclic peptides and their linear analogues and described correlations between the peptides’ spatial structures and their bactericidal abilities. In the current article, we have improved our set of technologies; in particular, we paid special attention to solving the problem with calibration of simulation parameters based on the experimental data and to the sampling problem (see 1′ and 2′ above). The most important results obtained with our improved tools for the detailed analysis of the antibacterial and hemolytic effects of the newly designed membrane-active peptides are described below:

(1) Using a rational structure-based design, we created AMPs with high bactericidal activity, along with activity against antibiotic-resistant strains and low hemolytic activity. We synthesized a large panel of peptides similar in amino acid composition but differing in length and sequence distribution of specific residues. We positioned cationic and hydrophobic residues in such a way that the peptides would have a well-defined amphipathic surface upon attaining the helical conformation within the proximity of the target membrane. After the initial test of the peptides’ antibacterial and hemolytic activity, we selected the most active peptides for additional evaluation of their bactericidal activity, cytotoxicity, and mechanistic studies of their mode of action.

Cationic AMPs are known to exert their antimicrobial action via physical disruption of bacterial membranes, which causes intracellular content release and eventually leads to cell death.^6^ The membranolytic action of the cationic AMPs is considered one of the primary reasons for their rapid killing action as compared to classical antibiotics.^34^ The ability of our lead peptides to rapidly neutralize the microbes via the membranolytic action potentially could leave bacterial pathogens with little scope for resistance development. The lead peptides **8a** and **8b** demonstrated strong membranolytic behavior on bacterial membranes mimicking liposomes (Figure 5) and live bacterial cells [Figures 6 and 7, S7 (Supporting Information)]. The evident morphological alterations induced by **8b** in the bacterial membrane led to the loss of membrane integrity and cell death (Figure 8).

We identified peptide **8b** as the most promising compound in terms of further optimization based on its rapid killing action against MRSA and *E. coli*, comparable to peptide-based antibiotics (Figure 4), and its good selectivity toward the bacterial membrane, evident from the weak toxicity against tested human cells (Figure 3).

(2) To identify the mechanism of antibacterial activity of peptide **8b**, as well as the major factors determining the activity, further study of the peptide was carried out using biophysical (NMR, CD) and computational (MD, mapping of hydrophobic properties) methods. The same approaches were applied in parallel to a close analogue of the peptide **8b**–peptide **6b**, which differs only in two substitutions (Leu residues in **8b** are replaced with Ala in **6b**), but at the same time demonstrates a very low activity. This direct comparison allowed us to evince a set of important features of the highly active peptide **8b**: upon binding and interaction with the membrane, **8b** obtains a more stable helical structure (Figure 13C), has a much more pronounced pattern of hydrophobicity on its surface (Figure 12A), interacts more strongly with the model bacterial membrane, and inserts deeper into it (Figure 13A), destabilizing the lipid bilayer. In summary, a combination of these features ensures fast and strong bactericidal activity of **8b**.
(3) A similar approach was applied to elucidate the factors determining the low-to-moderate hemolytic activity of **8b**. Peptides **6b** and hemolytically active **8a** (with all Lys replaced by Arg) from the synthesized panel were used as the “sparring partners” of peptide **8b**. The analysis shows that Arg residues, strongly interacting with the polar headgroups of zwitterionic lipids, stabilize the helical conformation of **8a** tightly bound to the model mammalian membrane and ensure deep immersion of the peptide into the membrane (Figure 13A). This explains a much higher cytotoxic effect of peptide **8a** compared to **6b** and **8b**, which require negatively charged lipids (much more abundant in the bacterial cell membrane) for efficient interaction with the membrane.

It should be noted that the results of biophysical experiments and modeling are consistent with each other in assessing the secondary structure of all studied peptides in the presence of a membrane, as well as in their ability to bind to the lipid bilayer. Such validation of MD results is very important because it significantly increases the reliability of the subsequent conclusions obtained via the calculations: the difference in binding to the membrane of active (**8b**) and inactive (**6b**) peptides and the atomistic structural and dynamic characteristics of peptides and membranes in an isolated state and in a complex. Collection of such information is an extremely time-consuming process, or it is still impossible to obtain it using modern experimental approaches. In addition, it should be noted that the conclusions based on modeling were made for a large ensemble of states obtained in a series of independent long-term MD runs. In the future, the proposed approach will be used both for constructing analogues based on **8b** with improved properties (increased therapeutic index, i.e. HC_50_/MIC) and for studying other AMP families.

## 4 Conclusions

By designing a series of amphiphilic peptides using a structure-based rational approach, we were able to identify short 12-mer AMPs with improved activity. Systematic analysis of lead peptides revealed the combined structural and functional role of particular amino acids in the antibacterial and hemolytic activity of the peptides. A structural transition from the extended structure to the amphiphilic helix in the presence of membrane-mimicking liposomes was evident for 12-mer peptides. The amino acids with long hydrophobic side chains (Leu and Ile) stabilize the amphipathic helix and allow deeper insertion into the cell membrane, resulting in rapid membranolytic action of the peptides against a broad range of multi-drug resistant strains. The data also revealed lower toxicity toward mammalian cells for the peptides with Lys as cationic amino acids compared with peptides that contain Arg. Overall, in addition to the identification of a small potent AMP with moderate hemolytic toxicity, the described key structural determinants important for the activity and selectivity of AMPs will be instrumental in the development of novel small peptide-based antibiotics. The described approach and obtained results will be used in the rational design of the next generation of broad-spectrum AMPs, limiting the resistance development ability of bacterial pathogens.

## 5 Experimental Section

### 5.1 Materials

Fmoc-Rink amide 4-methylbenzhydrilamine (MBHA) resin (loading 0.465 mmol/g, 100–200 mesh size) was purchased from Sigma-Aldrich (St. Louis, MO, USA). The coupling reagent 2-(1*H*-benzotriazole-1-yl)-1,1,3,3-tetramethylaminium hexafluorophosphate (HBTU) and Fmoc-amino acids, Fmoc-Ala-OH, Fmoc-Ile-OH, Fmoc-Leu-OH, Fmoc-Trp(Boc)-OH, Fmoc-Lys(Boc)-OH, and Fmoc-Arg(pbf)-OH, were purchased from AAPPTec LLC (Louisville, KY, USA). *N*,*N*-Dimethylformamide (DMF), *N,N*-diisopropylethylamine (DIPEA), trifluoroacetic acid (TFA), acetic acid, triisopropylsilane (TIS), piperidine, and all other reagents were bought from Sigma-Aldrich (St. Louis, MO, USA). 1-Hydroxybenzotriazole (HOBt) and 1,3-diisopropylcarbodiimide (DIC) were purchased from Sigma-Aldrich Chemical Co. (Milwaukee, WI, USA). Ultra-pure water was purchased from the Milli-Q system (Temecula, CA, USA). The MTS Assay Kit (98%) was purchased from Promega (Madison, WI, USA). All phospholipids and cholesterol were purchased from Avanti Polar Lipids (Alabaster, USA). Calcein dye was obtained from Sigma. Whole human blood was purchased from BioIVT, USA.

All mammalian and bacterial cell culture supplies were purchased from Corning (Christiansburg, VA, USA) and Fisher Scientific (Waltham, MA, USA). All the mammalian cell and bacterial experiments were carried out under a laminar flow hood Labconco (Kansas City, MO, USA). The cell culture was carried out at 37 °C with 5% CO_2_ in a Forma incubator using a T-75 flask. The human lung fibroblast cell (MRC-5, ATCC no. CCL-171), human embryonic kidney cells (HEK293, ATCC no. CRL 1573), human hepatoma HepaRG cells (Gibco, HPRGC10), and human epidermal keratinocytes (HEKa, ATCC PCS-200-011) were purchased from American Type Culture Collection (ATCC; USA). All cells were maintained in a 5% CO_2_ incubator (37 °C). Human serum was purchased from Sigma-Aldrich. All bacterial strains employed in this study are procured from VWR, USA, and propagated as per the recommendation of ATCC.

### 5.2 Solid-Phase Peptide Synthesis

The peptides were synthesized manually on Fmoc-Rink amide MBHA resin (loading 0.465 mmol/g) using the standard Fmoc/*t*Bu solid-phase peptide synthesis protocol. The resin was allowed to swell in dry DMF for 1 h and subjected to Fmoc deprotection using 20% piperidine in DMF (20%, v/v). The amino acid couplings were conducted by using Fmoc-l-amino acid (3 equiv), HOBt (3 equiv), and DIC (4 equiv) dissolved in dry DMF, and the reaction mixture was allowed to shake at room temperature for 2 h. The coupling of each amino acid was confirmed by a negative Kaiser test. After each successive coupling, the Fmoc protecting group was removed by treatment with piperidine in DMF (20%, v/v). After the completion of the desired peptide sequence, the N-terminal Fmoc protecting group was removed. For those peptides with N-acetylation, the resin was subjected to N-terminal acylation by treating the peptidyl resin with a mixture of pyridine and acetic anhydride (1:2; v/v) in DMF and allowing the reaction to progress at room temperature for 10 min. The peptidyl resin was dried under vacuum, and the peptide was cleaved from the resin with a mixture of TFA/water/TIS [95:2.5:2.5 (v/v/v)]. The resin was removed by filtration, and the peptide was precipitated with ice-cooled diethyl ether. After multiple ether washes, the crude mass was redissolved in acetonitrile/water (1:1 v/v with 0.5% TFA), and the solution was lyophilized to obtain the crude peptide.

### 5.3 Purification and Analytical Characterization of the Synthesized Peptides

The crude peptides were purified using a reversed-phase high-pressure liquid chromatography (RP-HPLC) system (Shimadzu LC-20AP). The peptides were dissolved in acetonitrile/water (2:1 v/v, with 0.5% TFA) to a final concentration of 20 mg/mL. Following filtration through a 0.45 μm Millipore filter, the peptide solutions were loaded onto a column via multiple 10 mL injections. A preparative C18 column (Gemini, 5 μm particle size, 100 Å pore size, 21.2 mm × 250 mm) from Phenomenex was used with a mixture of water and acetonitrile [both containing 0.1% (v/v) TFA] as an eluent at a flow rate of 8 mL/min. The detection wavelength was 220 nm. Fractions containing the desired peptides were lyophilized.

The purity analysis of the peptides was conducted on a RP-HPLC system (Shimadzu; LC-20ADXR) by using a Phenomenex (Luna) analytical C18 column (4 μm, 150 × 4.6 mm). The mass of the purified peptides was determined in the positive-ion mode using Q-TOF LC/MS (Compass Hystar 4.1, Bruker, USA). The purity (>95%) and mass data of all the synthesized peptides are provided in the Supporting Information. The relative hydrophobicity of the peptides (**6a–e**, **7a–d**, and **8a–e**) expressed as RP-HPLC elution time was determined using a Phenomenex (Luna) analytical C18 column (4 μm, 150 × 4.6 mm) with conditions, linear AB gradient (1% acetonitrile/min) at a flow rate of 0.3 mL/min, where eluant A was water with 0.1% TFA (v/v) and eluant B was acetonitrile with 0.1% TFA (v/v) and the temperature was 25 °C (chromatograms are provided in the Supporting Information).

NH_2_-R-W-R-R-W-W-R-CONH_2_ (**1a**): HR-MS (ESI-TOF) (*m*/*z*) C_57_H_81_N_23_O_7_ calcd, 1200.4290; found, 1201.6819 [M + H]^+^, 600.8397 [M + H]^2+^; NH_2_-R-R-W-W-R-R-W-CONH_2_ (**1b**): HR-MS (ESI-TOF) (*m*/*z*): C_57_H_81_N_23_O_7_ calcd, 1200.4290; found, 1201.3672 [M + H]^+^, 600.6390 [M + H]^2+^; NH_2_-W-R-R-W-W-R-R-CONH_2_ (**1c**): HR-MS (ESI-TOF) (*m*/*z*): C_57_H_81_N_23_O_7_ calcd, 1200.4290; found, 1201.3702 [M + H]^+^, 600.6396 [M + H]^2+^; NH_2_-R-R-W-R-R-W-W-CONH_2_ (**1d**): HR-MS (ESI-TOF) (*m*/*z*): C_57_H_81_N_23_O_7_ calcd, 1200.4290; found, 1201.3710 [M + H]^+^, 600.6402 [M + H]^2+^; NH_2_-R-W-W-R-R-W-R-CONH_2_ (**1e**): HR-MS (ESI-TOF) (*m*/*z*): C_57_H_81_N_23_O_7_ calcd, 1200.4290; found, 1202.6720 [M+2H]^+^, 601.3404 [M + H]^2+^; NH_2_-R-W-W-R-R-W-A-R-CONH_2_ (**2a**): HR-MS (ESI-TOF) (*m*/*z*): C_60_H_86_N_24_O_8_ calcd, 1271.5080; found, 1272.7128 [M + H]^+^, 636.3602 [M + H]^2+^; NH_2_-R-W-W-R-R-A-W-R-CONH_2_ (**2b**): HR-MS (ESI-TOF) (*m*/*z*): C_60_H_86_N_24_O_8_ calcd, 1271.5080; found, 1272.7125 [M + H]^+^, 636.8605 [M + H]^2+^; NH_2_-R-W-A-R-R-W-W-R-CONH_2_ (**2c**): HR-MS (ESI-TOF) (*m*/*z*): C_60_H_86_N_24_O_8_ calcd, 1271.5080; found, 1272.7140 [M + H]^+^, 636.8608 [M + H]^2+^; NH_2_-W-R-R-W-A-R-R-W-CONH_2_ (**2d**): HR-MS (ESI-TOF) (*m*/*z*): C_60_H_86_N_24_O_8_ calcd, 1271.5080; found, 1272.7138 [M + H]^+^, 636.8584 [M + H]^2+^; NH_2_-A-R-R-W-W-R-R-W-CONH_2_ (**2e**): HR-MS (ESI-TOF) (*m*/*z*): C_60_H_86_N_24_O_8_ calcd, 1271.5080; found, 1272.7108 [M + H]^+^, 636.8596 [M + H]^2+^; NH_2_-R-W-W-R-R-A-W-R-A-CONH_2_ (**3a**): HR-MS (ESI-TOF) (*m*/*z*): C_63_H_91_N_25_O_9_ calcd, 1342.5870; found, 1344.0491 [M + H]^+^, 672.0780 [M + H]^2+^; NH_2_-R-A-W-R-R-W-W-R-A-CONH_2_ (**3b**): HR-MS (ESI-TOF) (*m*/*z*): C_63_H_91_N_25_O_9_ calcd, 1342.5870; found, 1343.7501 [M + H]^+^, 671.8785 [M + H]^2+^; NH_2_-R-W-A-R-R-W-W-R-A-CONH_2_ (**3c**): HR-MS (ESI-TOF) (*m*/*z*): C_63_H_91_N_25_O_9_ calcd, 1342.5870; found, 1344.0474 [M + H]^+^, 672.0775 [M + H]^2+^; NH_2_-R-W-W-R-A-A-W-R-R-CONH_2_ (**3d**): HR-MS (ESI-TOF) (*m*/*z*): C_63_H_91_N_25_O_9_ calcd, 1342.5870; found, 1343.7476 [M + H]^+^, 671.8777 [M + H]^2+^; NH_2_-R-W-W-R-R-A-W-A-R-CONH_2_ (**3e**): HR-MS (ESI-TOF) (*m*/*z*): C_63_H_91_N_25_O_9_ calcd, 1342.5870; found, 1343.7474 [M + H]^+^, 671.8773 [M + H]^2+^; NH_2_-A-W-W-R-R-A-W-R-R-CONH_2_ (**3f**): HR-MS (ESI-TOF) (*m*/*z*): C_63_H_91_N_25_O_9_ calcd, 1342.5870; found, 1344.0518 [M + H]^+^, 672.0793 [M + H]^2+^; NH_2_-R-A-W-R-R-A-W-R-A-W-CONH_2_ (**4a**): HR-MS (ESI-TOF) (*m*/*z*): C_66_H_96_N_26_O_10_ calcd, 1413.6660; found, 1415.7931 [M+2H]^+^, 707.8991 [M + H]^2+^, 472.2691 [M + H]^3+^; NH_2_-R-A-W-R-R-W-W-R-A-A-CONH_2_ (**4b**): HR-MS (ESI-TOF) (*m*/*z*): C_66_H_96_N_26_O_10_ calcd, 1413.6660; found, 1415.0931 [M + H]^+^, 707.5003 [M + H]^2+^, 471.6700 [M + H]^3+^; NH_2_-R-W-W-R-R-A-W-R-A-A-CONH_2_ (**4c**): HR-MS (ESI-TOF) (*m*/*z*): C_66_H_96_N_26_O_10_ calcd, 1413.6660; found, 1415.0918 [M + H]^+^, 707.5999 [M + H]^2+^, 471.6696 [M]^3+^; NH_2_-R-A-W-R-R-W-A-R-A-W-CONH_2_ (**4d**): HR-MS (ESI-TOF) (*m*/*z*) C_66_H_96_N_26_O_10_ calcd, 1413.6660; found, 1415.7829 [M+2H]^+^, 707.8947 [M + H]^2+^, 472.2664 [M + H]^3+^; NH_2_-R-W-A-R-R-A-W-R-A-W-CONH_2_ (**4e**): HR-MS (ESI-TOF) (*m*/*z*): C_66_H_96_N_26_O_10_ calcd, 1413.6660; found, 1415.0838 [M + H]^+^, 707.5955 [M + H]^2+^, 471.6667 [M]^3+^; NH_2_-R-A-W-R-A-W-W-R-R-A-CONH_2_ (**4f**): HR-MS (ESI-TOF) (*m*/*z*): C_66_H_96_N_26_O_10_ calcd, 1413.6660; found, 1415.0826 [M + H]^+^, 707.5951 [M + H]^2+^, 471.6663 [M]^3+^; NH_2_-R-A-W-R-R-W-W-A-R-A-CONH_2_ (**4g**): HR-MS (ESI-TOF) (*m*/*z*): C_66_H_96_N_26_O_10_ calcd, 1413.6660; found, 1415.7913 [M+2H]^+^, 707.8982 [M + H]^2+^, 472.2685 [M + H]^3+^; NH_2_-A-A-W-R-R-W-W-R-R-A-CONH_2_ (**4h**): HR-MS (ESI-TOF) (*m*/*z*) C_66_H_96_N_26_O_10_ calcd, 1413.6660; found, 1415.7901 [M+2H]^+^, 707.8989 [M + H]^2+^, 472.2688 [M + H]^3+^; NH_2_-R-A-A-R-R-W-A-R-W-W-R-CONH_2_ (**5a**): HR-MS (ESI-TOF) (*m*/*z*): C_72_H_108_N_30_O_11_ calcd, 1569.8550; found, 1570.8114 [M + H]^+^, 842.4118 [M + TFA]^2+^, 785.4117 [M + H]^2+^, 523.6114 [M + H]^3+^; NH_2_-R-W-A-R-R-W-A-R-W-W-R-CONH_2_ (**5b**): HR-MS (ESI-TOF) (*m*/*z*): C_80_H_113_N_31_O_11_ calcd, 1684.9900; found, 1686.0234 [M + H]^+^, 900.0117 [M + TFA]^2+^, 843.0116 [M + H]^2+^, 562.0078 [M + H]^3+^; NH_2_-R-W-I-R-R-W-I-R-W-W-R-CONH_2_ (**5c**): HR-MS (ESI-TOF) (*m*/*z*): C_86_H_125_N_31_O_11_ calcd, 1769.1520; found, 1770.0895 [M + H]^+^, 942.0095 [M + TFA]^2+^, 885.0447 [M + H]^2+^, 591.0120 [M+2H]^3+^, 442.5044 [M + H]^4+^; NH_2_-R-W-L-R-R-W-L-R-W-W-R-CONH_2_ (**5d**): HR-MS (ESI-TOF) (*m*/*z*): C_86_H_125_N_31_O_11_ calcd, 1769.1520; found, 1770.0898 [M + H]^+^, 942.0108 [M + TFA]^2+^, 885.0411 [M + H]^2+^, 591.0129 [M + H]^3+^, 442.5221 [M + H]^4+^; Ac-R-W-I-R-R-W-I-R-W-W-R-CONH_2_ (**5e**): HR-MS (ESI-TOF) (*m*/*z*) C_88_H_127_N_31_O_12_ calcd, 1811.1890; found, 1812.0538 [M + H]^+^, 963.0233 [M + TFA]^2+^, 906.0202 [M + H]^2+^, 604.0171 [M + H]^3+,^ 453.0206 [M + H]^4+^; Ac-R-W-L-R-R-W-L-R-W-W-R-CONH_2_ (**5f**): HR-MS (ESI-TOF) (*m*/*z*): C_88_H_127_N_31_O_12_ calcd, 1811.1890; found, 1812.0548 [M + H]^+^, 906.0202 [M + H]^2+^, 604.0174 [M + H]^3+,^ 453.0048 [M + H]^4+^; NH_2_-R-W-A-R-R-W-A-R-W-W-R-R-CONH_2_ (**6a**): HR-MS (ESI-TOF) (*m*/*z*): C_86_H_125_N_35_O_12_ calcd, 1841.1790; found, 1842.0453 [M + H]^+^, 921.0344 [M + H]^2+^, 614.0935 [M + H]^3+,^ 461.0058 [M + H]^4+^; NH_2_-K-W-A-K-K-W-A-K-W-W-K-K-CONH_2_ (**6b**): HR-MS (ESI-TOF) (*m*/*z*): C_86_H_125_N_23_O_12_ calcd, 1673.0950; found, 1674.0214 [M + H]^+^, 837.5156 [M + H]^2+^, 558.6802 [M + H]^3+^; Ac-R-W-A-R-R-W-A-R-W-W-R-R-CONH_2_ (**6c**): HR-MS (ESI-TOF) (*m*/*z*): C_88_H_127_N_35_O_13_ calcd, 1883.2160; found, 999.0371 [M + TFA]^2+^, 942.0377 [M + H]^2+^, 628.0957 [M + H]^3+,^ 471.0739 [M + H]^4+^; Ac-K-W-A-K-K-W-A-K-W-W-K-K-CONH_2_ (**6d**): HR-MS (ESI-TOF) (*m*/*z*): C_88_H_127_N_23_O_13_ calcd, 1715.1320; found, 1716.0289 [M + H]^+^, 858.0181 [M + H]^2+^, 572.6828 [M + H]^3+^; NH_2_-K-W-A-K-K-W-W-K-W-W-K-K-CONH_2_ (**6e**): HR-MS (ESI-TOF) (*m*/*z*): C_94_H_130_N_24_O_12_ calcd, 1788.2300; found, 1789.0496 [M + H]^+^, 895.0266 [M + H]^2+^, 597.0237 [M]^3+^; NH_2_-R-W-I-R-R-W-I-R-W-W-R-R-CONH_2_ (**7a**): HR-MS (ESI-TOF) (*m*/*z*): C_92_H_137_N_35_O_12_ calcd, 1925.3410; found, 963.0784 [M + H]^2+^, 1020.0218 [M + TFA]^2+^, 642.0224 [M + H]^3+^, 481.2920 [M + H]^4+^; NH_2_-K-W-I-K-K-W-I-K-W-W-K-K-CONH_2_ (**7b**): HR-MS (ESI-TOF) (*m*/*z*): C_92_H_137_N_23_O_12_ calcd, 1757.2570; found, 1758.0148 [M + H]^+^, 879.0814 [M + H]^2+^, 586.0080 [M + H]^3+^, 440.2927 [M+2H]^4+^; Ac-R-W-I-R-R-W-I-R-W-W-R-R-CONH_2_ (**7c**): HR-MS (ESI-TOF) (*m*/*z*): C_94_H_139_N_35_O_13_ calcd, 1967.3780; found, 984.0841 [M + H]^2+^, 656.0261 [M + H]^3+^, 491.7949 [M + H]^4+^; Ac-K-W-I-K-K-W-I-K-W-W-K-K-CONH_2_ (**7d**): HR-MS (ESI-TOF) (*m*/*z*): C_94_H_139_N_23_O_13_ calcd, 1799.2940; found, 1800.1205 [M + H]^+^, 900.0644 [M + H]^2+^, 600.0128 [M + H]^3+^; NH_2_-R-W-L-R-R-W-L-R-W-W-R-R-CONH_2_ (**8a**): HR-MS (ESI-TOF) (*m*/*z*): C_92_H_137_N_35_O_12_ calcd, 1925.3410; found, 963.0777 [M + H]^2+^, 642.0221 [M + H]^3+^, 481.2917 [M + H]^4+^; NH_2_-K-W-L-K-K-W-L-K-W-W-K-K-CONH_2_ (**8b**): HR-MS (ESI-TOF) (*m*/*z*): C_92_H_137_N_23_O_12_ calcd, 1757.2570; found, 1758.1062 [M + H]^+^, 879.5566 [M + H]^2+^, 586.7080 [M + H]^3+^, 440.2934 [M + H]^4+^; Ac-R-W-L-R-R-W-L-R-W-W-R-R-CONH_2_ (**8c**): HR-MS (ESI-TOF) (*m*/*z*): C_94_H_139_N_35_O_13_ calcd, 1967.3780; found, 984.0858 [M + H]^2+^, 656.0277 [M + H]^3+^, 491.7963 [M + H]^4+^; Ac-K-W-L-K-K-W-L-K-W-W-K-K-CONH_2_ (**8d**): HR-MS (ESI-TOF) (*m*/*z*): C_94_H_139_N_23_O_13_ calcd, 1799.2940; found, 1800.1220 [M + H]^+^, 900.0640 [M + H]^2+^, 600.0127 [M + H]^3+^, 450.0938 [M + H]^4+^; NH_2_-K-W-L-K-K-W-W-K-W-W-K-K-CONH_2_ (**8e**): HR-MS (ESI-TOF) (*m*/*z*): C_97_H_136_N_24_O_12_ calcd, 1830.3110; found, 1831.0602 [M + H]^+^, 916.0329 [M + H]^2+^, 611.0279 [M+2H]^3+^.

### 5.4 Measurement of Antibacterial Activity

The antibacterial activity of all peptides (**1a–8d**) was determined by screening against a range of susceptible as well as drug-resistant bacterial strains. Description of the characteristics and growth conditions of various bacterial strains used in the study are provided in the Supporting Information (Table S3). Antibacterial susceptibility testing was carried out using a standard microtiter dilution method recommended by the clinical and laboratory standard institute (CLSI) and measured as a minimum inhibitory concentration (MIC), the lowest peptide concentration that inhibited bacterial growth. Briefly, the overnight grown cultures in the recommended broth for each bacterial strain were diluted in cation-adjusted Mueller Hinton Broth (CAMHB) to give an inoculum of 10^6^ colony-forming units (CFU)/mL. A 2-fold serially diluted test peptide solution (100 μL) was added to the microtiter plates. After adding bacterial suspension (100 μL), the plates were incubated at 37 °C for 24 h, and the MICs were determined. The same protocol was used to determine the MICs in the presence of salts and serum except using the media supplemented with various cationic salts (150 mM NaCl, 4.5 mM KCl, 6 mM NH_4_Cl, 1 mM MgCl_2_, and 2 mM CaCl_2_) or 25% FBS. The data was acquired from three independent assays performed in triplicate.

### 5.5 Measurement of Hemolytic Activity

The hemolytic activity of all peptides (**1a–8d**) was determined using hRBC. The assay was conducted by adding 75 μL of 2-fold serially diluted peptides to 75 μL of hRBC suspension (4% in PBS). The plates were incubated for 2 h at 37 °C without agitation. In order to determine the hemolysis at each tested concentration of peptides, the plate was centrifuged, and 100 μL of the supernatant was transferred to another 96-well plate to determine hemoglobin release spectrophotometrically at 567 nm. Percent hemolysis was calculated by the following formula
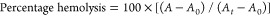
where *A* represents the absorbance of the peptide sample at 567 nm and *A*_0_ and *A*_*t*_ represent zero percent and 100% hemolysis determined in phosphate buffer saline and 1% Triton X-100, respectively.

### 5.6 Cytotoxicity

The in vitro cytotoxicity of **8a** and **8b** was evaluated using human lung fibroblast cells (MRC-5), human embryonic kidney cells (HEK-293), human hepatic cells (HepaRG), and human epidermal keratinocytes (HEKa). Cells were seeded at 10,000 per well in 0.1 mL of media in 96-well plates 24 h prior to the experiment. Lung and kidney cells were seeded in DMEM medium containing FBS (10%). Liver cells were seeded in William’s E medium with the GlutaMAX supplement. Epidermal keratinocytes were seeded in a Dermal Cell Basal medium supplemented with a keratinocyte growth kit. The peptides were added to each well in triplicates at a variable concentration of 10–250 μg/mL and incubated for 24h at 37 °C in a humidified atmosphere of 5% CO_2_. After the incubation period, the MTS solution (20 μL) was added to each well. Then, the cells were incubated for 2 h at 37 °C, and the cell viability was determined by measuring the absorbance at 490 nm using a SpectraMaxM2 microplate spectrophotometer. The percentage of cell survival was calculated as [(OD value of cells treated with the test mixture of compounds) – (OD value of culture medium)]/[(OD value of control cells) – (OD value of culture medium)] × 100%.

### 5.7 Bactericidal Kinetics

The time course of bacterial killing was studied by the exposure of overnight grown cultures of MRSA (ATCC BAA-1556) and *E. coli* (ATCC BAA-2452) to **8a** and **8b** at the MIC and 4× the MIC in Muller Hinton media. Bacterial cells (2 × 10^6^ CFU/mL) were treated with peptides and standard antibiotics and incubated at 37 °C. Aliquots were withdrawn at a 30 min time interval for 4 h and plated on the agar plate to determine the number of viable bacterial colonies. Data were obtained from two independent experiments performed in triplicate.

### 5.8 Calcein Dye Leakage Assay

The calcein dye leakage assay was conducted using the large unilamellar vesicles (LUVs) composed of 1,2-dioleoyl-*sn*-glycero-3-phosphocholine (DOPC) and 1,2-dioleoyl-*sn*-glycero-3-phosphoglycerol (DOPG) to mimic the bacterial membrane (DOPC/DOPG, 7:3, w/w) or mammalian membrane (DOPC/cholesterol, 10:1, w/w), as we described previously.^18^ At various concentrations (5, 10, 20, 30, and 50 μg/mL) test peptides **8a** and **8b** (50 μL) mixed with liposome suspension (50 μL), and fluorescence intensity was read every 10 min for 100 min at an excitation wavelength of 490 nm and an emission wavelength of 520 nm on a SpectraMax M5 multi-mode microplate reader. Considering calcein release from liposomes treated with a 10% solution (w/v) of Triton X-100 as 100%, the apparent percentage of dye leakage was calculated using the following formula
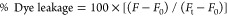
where *F* is the intensity measured at a given peptide concentration, *F*_0_ is the background intensity of the liposome sample, and *F*_t_ is the intensity after lysis induced by Triton X-100.

### 5.9 Fluorescence Microscopy

Fluorescence microscopy assay was performed by double-staining method using DAPI and PI as fluorophores. MRSA (ATCC BAA-1556) and *E. coli* (ATCC BAA-2452) cells in the mid-logarithmic phase were harvested by centrifugation and washed three times with PBS (10 mM, pH 7.3). Bacterial cells (10^7^ CFU/mL) were incubated with the test peptides (**8a** or **8b**) or with the standard antibiotics (daptomycin or polymyxin B) at the concentration of MIC and 4 × MIC for 1 h. Then, the cells were pelleted by centrifugation at 3000*g* for 15 min in a microcentrifuge. The supernatant was decanted, and the cells were washed with PBS several times and incubated with PI (10 mg/mL) in the dark for 15 min at 37 °C. The excess PI was removed by washing the cells with PBS several times. Then, the cells were incubated with DAPI (20 mg/mL) for 15 min in the dark at 37 °C. The DAPI solution was removed, and cells were washed with PBS several times. Controls were performed by following the same procedure without the treatment with test peptides or standard antibiotics. The bacterial cells were then examined under a Keyence fluorescence microscope (BZ-X710) with an oil-immersion objective (60×).

### 5.10 FACS Analysis

The flow cytometric analysis was performed by using MRSA (ATCC BAA-1556) and *E. coli* (ATCC BAA-2452) cultures grown to the mid log phase in Mueller Hinton broth (HIMEDIA). Before treatment, bacterial cells were washed thrice with buffer (10 mM Tris, pH 7.4) and resuspended in the same buffer to obtain 10^7^ CFU/mL bacterial suspensions. Test peptides (**8a** and **8b**) and the standard antibiotics (daptomycin and polymyxin B) at MIC and 4 × MIC were incubated with bacterial suspension for 1 h. Following the treatment with test peptides, the cells were pelleted by centrifugation at 3000*g* for 15 min in a microcentrifuge. The supernatant was decanted, and the cells were washed with PBS several times and then incubated with PI (10 mg/mL) in the dark for 15 min at 37 °C. FACS analysis of the stained bacterial cells was performed using a FACscan flow cytometer (BD Accuri C6, BD Biosciences, California, USA), and data were analyzed by using Cell Quest software.

### 5.11 SEM Analysis

SEM analysis of untreated and peptide (**8b**)-treated MRSA (ATCC BAA-1556) and *E. coli* (ATCC BAA-2452) cells was conducted. Bacterial cells were cultured to the exponential phase in MHB at 37 °C under constant shaking at 210 rpm. The overnight bacterial cultures were diluted to obtain an inoculum size of 2 × 10^6^ CFU/mL and treated with **8b** at 4 × MIC for 30 min. After treatment, the bacterial cell suspension was centrifuged at 2000 × *g* for 10 min, and the cell pellets were washed thrice with PBS and re-suspended in deionized water. Untreated bacterial cells are included as a control. For SEM analysis, the cells were fixed by treating them with 4% glutaraldehyde in 0.2 MNa-cacodylate buffer for 3 h. The samples were dehydrated with a graded series of ethanol and dried using HMDS (hexamethyl disilazane). Before analysis, samples were subjected to Au/Pd coating (approximately 20 nm thicknesses) and observed under a scanning electron microscope (Zeiss Sigma 300).

### 5.12 CD Measurements

The CD spectra of peptides were recorded using a Jasco J-1500 CD spectrophotometer (Jasco, Easton, MD) at 25 °C with a 1 mm path length cell. Wavelengths from 190 to 260 nm were scanned with 1.0 nm step resolution, 100 nm/min speed, 0.4 s response time, and 1 nm bandwidth. CD spectra of the peptides were collected and averaged over three scans in 10 mM sodium phosphate buffer (pH 7.2), helix-inducing solvent 50% TFE (v/v), and bacterial mimic liposomes (75 μM lipid). Spectra were corrected by subtracting a baseline spectrum containing only buffer or 50% TFE or liposomes. The mean residue molar ellipticity was calculated using the formula
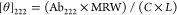
where Ab_222_ is the absorbance observed at 222 nm, MRW is the mean residue molecular weight, *C* is the concentration in mg/mL, and *L* is the path length.

### 5.13 NMR Spectroscopy

NMR spectra were recorded on a Bruker Ascend spectrometer (400 MHz) equipped with a Prodigy Broadband (BBI) Cryoprobe. All NMR data were acquired and processed using Topspin software (Bruker Biospin). All NMR spectra were recorded using standard Bruker pulse sequences with gradient water suppression. 1D and 2D ^1^H NMR spectra for lead peptide **8b** and its closest analogues **6b**, **7b**, and **8a** were recorded at a concentration of 1.5 mM in water and in the presence of liposomes at 25 °C, and changes in the linewidths, positions of the backbone amide and aromatic resonances, and NOE cross-peaks were analyzed. For the analysis of the interaction with liposomes, the peptides were mixed with different amounts of DOPC/DOPG liposomes (7:3, w/w; concentration 3.42 mM) or DOPC/cholesterol (10:1, w/w; concentration 3.40 mM) prepared as described above. To achieve the lipid/peptide molar ratio of approximately 1:1, the samples were prepared by mixing equal volumes of peptide stock solution (3 mM) and liposome stock solution. The NMR data were analyzed using Topspin (Bruker Biospin) and CARA software.^35^

### 5.14 Molecular Dynamics Simulations

To study the peptide–membrane interactions, a set of MD simulations of the functionally alternative pair of peptides **6b** and **8b** were carried out in two model lipid membranes: hydrated pre-equilibrated zwitterionic and net negatively charged bilayers composed of DOPC and DOPC/DOPG lipids, respectively. Both model membranes contained 128 lipid molecules. To reproduce the lipid ratio (7:3 w/w) used in experiments, the two-component bilayer consisted of 96 DOPC and 32 DOPG lipid molecules. To explain the high hemolytic activity of Arg-containing peptides, additional simulations of peptide **8a** were conducted in the DOPC bilayer mimicking a mammalian membrane.

#### 5.14.1 Preparation of Starting Configurations

Due to the high conformational plasticity of the peptides in water and mainly helical conformation in the presence of liposomes (according to NMR data). Only one type of starting structure–α-helix–was selected. The initial spatial structures of the peptides were constructed with Maestro, version 9.3.5 (Schrödinger, USA). At least 12 independent MD runs were performed for each peptide–membrane system, giving a total simulation time of 6.4 μs for peptides **6b** and **8b** in the DOPC/DOPG bilayer and 2.4 μs for peptides **6b**, **8a**, and **8b** in the DOPC membrane. The peptide molecule was fully exposed to water and located next to the membrane surface in all starting positions.

#### 5.14.2 MD Protocols

MD simulations were performed using the GROMACS^36^ package version 2020.4 and the all-atom CHARMM36 force field.^37^ In all calculations, the tip3p^38^ water model and 3D periodic boundary conditions were employed. To keep the system electrically neutral, Na^+^ counterions were added. A spherical cutoff function (12 Å) and the particle mesh Ewald (PME) algorithm^39^ (with a 12 Å cutoff) were used to treat van der Waals and electrostatic interactions, respectively. The preparation and production of the MD stages were the same for all peptides and described elsewhere.^18^ Finally, MD production runs (duration at least 200 ns each) were conducted in an *NPT* ensemble at a semi-isotropic pressure and a constant temperature of 310 K with an integration step of 2 fs.

#### 5.14.3 Data Analysis

MD data were analyzed and averaged over sets of MD trajectories (depending on the peptide and its mode of membrane binding, see Table 3). MD trajectories were sampled for analysis at time intervals of 100–1000 ps. The conformational mobility of the peptides and their secondary structure were evaluated using standard GROMACS utilities (*gmx rms*, *gmx dssp*). Intermolecular contacts (including hydrophobic and electrostatic interactions and hydrogen bonds), as well as the depth of peptide insertion into the membrane, were delineated using GROMACS tools (*gmx hbond*, *gmx distance*) and IMPULSE software.^40^ The accessible surface area (“contact area”) of the peptide that is in contact with membrane lipids was estimated by *naccess* software.^41^ The distribution of hydrophobic/hydrophilic properties on the molecular surfaces of peptides was calculated using the molecular hydrophobicity potential (MHP) approach^28^ implemented in the PLATINUM software.^42^ For the analysis, the MHP values were calculated in log *P* units, where *P* is the octanol–water distribution coefficient. Molecular graphics were rendered using PyMOL v. 2.5 (http://pymol.org).

## Supporting Information Available

Helical wheel projection, CD analysis, antibacterial activity in the presence of salts and serum, cytotoxicity of daptomycin, description of the growth conditions of bacterial strains, flow cytometry analysis, analytical HPLC data of the synthesized compounds, comparative hydrophobicity analysis, mass spectra, and NMR analysis (PDF)
Molecular formula strings (CSV)

## Abbreviations

AMP: antimicrobial peptide
CFU: colony-forming units
DAPI: 4′,6-diamidino-2-phenylindole
DIC: *N*,*N*-diisopropylcarbodiimide
DOPC: diolylphosphatidylcholine
DOPG: diolylphosphatidylglycerol
HOBt: 1-hydroxybenzotriazole
LUVs: large unilamellar vesicles
MeCN: acetonitrile
MD: molecular dynamics
MHB: Müller-Hinton broth
MHP: molecular hydrophobicity potential
PI: propidium iodide
RMSD: root-mean-square deviation
TFE: 2,2,2-trifluoroethanol
TIS: triisopropylsilane
