# Supplementary material for: Structure-Based Rational Design of Small α-Helical Peptides with Broad-Spectrum Activity against Multidrug-Resistant Pathogens
Source: J Med Chem. 2022 Dec 27;66(1):855–74. doi: 10.1021/acs.jmedchem.2c01708 (PMC9841524; doi:10.1021/acs.jmedchem.2c01708)
Supplement: Supplementary file 1 — jm2c01708_si_001.pdf [file jm2c01708_si_001.pdf]

Supporting Information

Structure-Based Rational Design of Small α-Helical Peptides with Broad-Spectrum Activity Against Multidrug-Resistant Pathogens

Sandeep Lohan,[§,$] Anastasia G. Konshina,[‡] Roman G. Efremov,[‡,§,ψ] Innokentiy Maslennikov,[†] Keykavous Parang[§]

[§]Center for Targeted Drug Delivery, Department of Biomedical and Pharmaceutical Sciences, Chapman University School of Pharmacy, Harry and Diane Rinker Health Science Campus, 94 01 Jeronimo Rd, Irvine, California 92618, United States

[$]AJK Biopharmaceutical, 5270 California Ave, Irvine, CA 92617

[†]Structural Biology Research Center, Department of Biomedical and Pharmaceutical Sciences, Chapman University School of Pharmacy, Harry and Diane Rinker Health Science Campus, 9401 Jeronimo Rd., Irvine, California 92618, United States

[‡]M.M. Shemyakin & Yu.A. Ovchinnikov Institute of Bioorganic Chemistry, Russian Academy of Sciences, Miklukho-Maklaya Street, 16/10, Moscow, 117997, Russia

[§]National Research University Higher School of Economics, Myasnitskaya ul. 20, Moscow, 101000, Russia

[ψ]Moscow Institute of Physics and Technology (State University), Dolgoprudny, 141701 Moscow Oblast, Russia

Table of Contents **Page**

1. **Helical wheel projection diagrams……………………………………………………S3**
   Figure S1. Helical wheel projection diagram of synthesized peptides
2. **Circular Dichroism (CD) analysis……………………………………….….………..S4-S8**
   Figure S2. CD spectra of **1c**, **2a**, **3e**, **4f**, and **5d** recorded in PBS, TFE, and Liposomes.
   Figure S3. CD spectra of **6a-6e** recorded in PBS, TFE, and Liposomes.
   Figure S4. CD spectra of **7a-7d** recorded in PBS, TFE, and Liposomes.
   Figure S5. CD spectra of **8a-8e** recorded in PBS, TFE, and Liposomes.
   Table S1. RP-HPLC retention times and CD spectroscopy analysis of selected peptides.
3. **Antibacterial activity in the presence of salts and serum………………………………S9**
   Table S2. Antibacterial activities of lead peptides (**7a**, **7b**, **8a**, and **8b**) and antibiotics in the presence of FBS and various cationic salts.
4. **Cytotoxicity assay of daptomycin**…………………………………………………**S10**
   Figure S6. Cytotoxicity of daptomycin against human embryonic kidney (HEK293) and human liver cells (HPRGC10).
5. **Bacterial propagation………………………………………………………………S11**
   Table S3. Description of the characteristics and growth conditions of various bacterial strains used in the study.
6. **Flow cytometry (FACS) analysis.…………………………………………..........S12**
   Figure S7. Flow cytometric analysis of MRSA (A1-A8) and *E. coli* (B1-B8) treated with peptides **8a** and **8b** or standard antibiotics daptomycin and polymyxin B at MIC and 4×MIC.
7. **Purity Analysis………………………………………………………..…………..S13-S35**
   HPLC Chromatograms of all synthesized peptides (Figures S8-S51)
8. **Comparative hydrophobicity analysis………………………………………………S36-S43**
   HPLC Chromatograms (Figures S52-S65)
9. **High Resolution Mass Spectrometry (HR-MS) data………………………...……S44-S65**
   HR-MS data of all synthesized peptides (Figures S66-S109)
10. **High Resolution NMR data…………………………………………………….S66-S67**
   NMR chemical shift assignment for peptides (Tables S4-S7)

1. Helical wheel projection diagrams

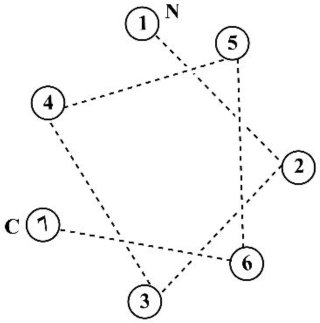

Code	1	2	3	4	5	6	7
1a	R	W	R	R	W	W	R
1b	R	R	W	W	R	R	W
1c	W	R	R	W	W	R	R
1d	R	R	W	R	R	W	W
1e	R	W	W	R	R	W	R

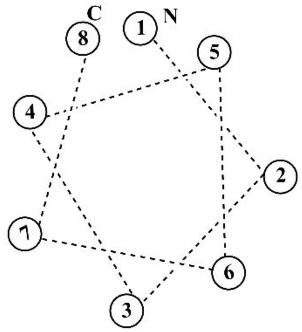

Code	1	2	3	4	5	6	7	8
2a	R	W	W	R	R	W	A	R
2b	R	W	W	R	R	A	W	R
2c	R	W	A	R	R	W	W	R
2d	W	R	R	W	A	R	R	W
2e	A	R	R	W	W	R	R	W

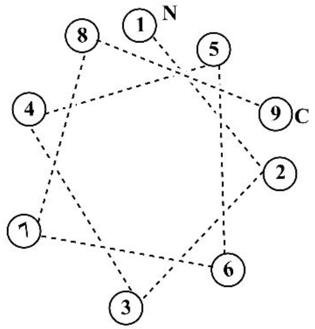

Code	1	2	3	4	5	6	7	8	9
3a	R	W	W	R	R	A	W	R	A
3b	R	A	W	R	R	W	W	R	A
3c	R	W	A	R	R	W	W	R	A
3d	R	W	W	R	A	A	W	R	R
3e	R	W	W	R	R	A	W	A	R
3f	A	W	W	R	R	A	W	R	R

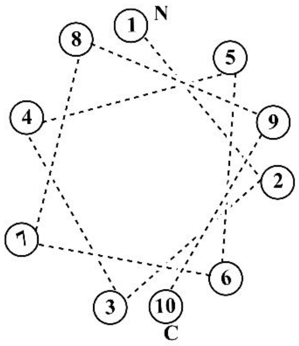

Code	1	2	3	4	5	6	7	8	9	10
4a	R	A	W	R	R	A	W	R	A	W
4b	R	A	W	R	R	W	W	R	A	A
4c	R	W	W	R	R	A	W	R	A	A
4d	R	A	W	R	R	W	A	R	A	W
4e	R	W	A	R	R	A	W	R	A	W
4f	R	A	W	R	A	W	W	R	R	A
4g	R	A	W	R	R	W	W	A	R	A
4h	A	A	W	R	R	W	W	R	R	A

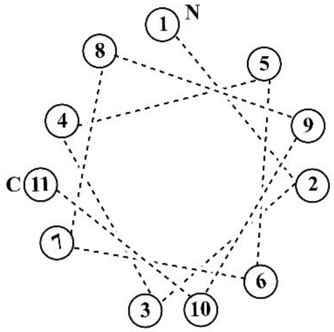

Code	1	2	3	4	5	6	7	8	9	10	11
5a	R	A	A	R	R	W	A	R	W	W	R
5b	R	W	A	R	R	W	A	R	W	W	R
5c	R	W	I	R	R	W	I	R	W	W	R
5d	R	W	L	R	R	W	L	R	W	W	R

Figure S1. Helical wheel projection diagram of synthesized peptides.

2. Circular dichroism (CD) analysis

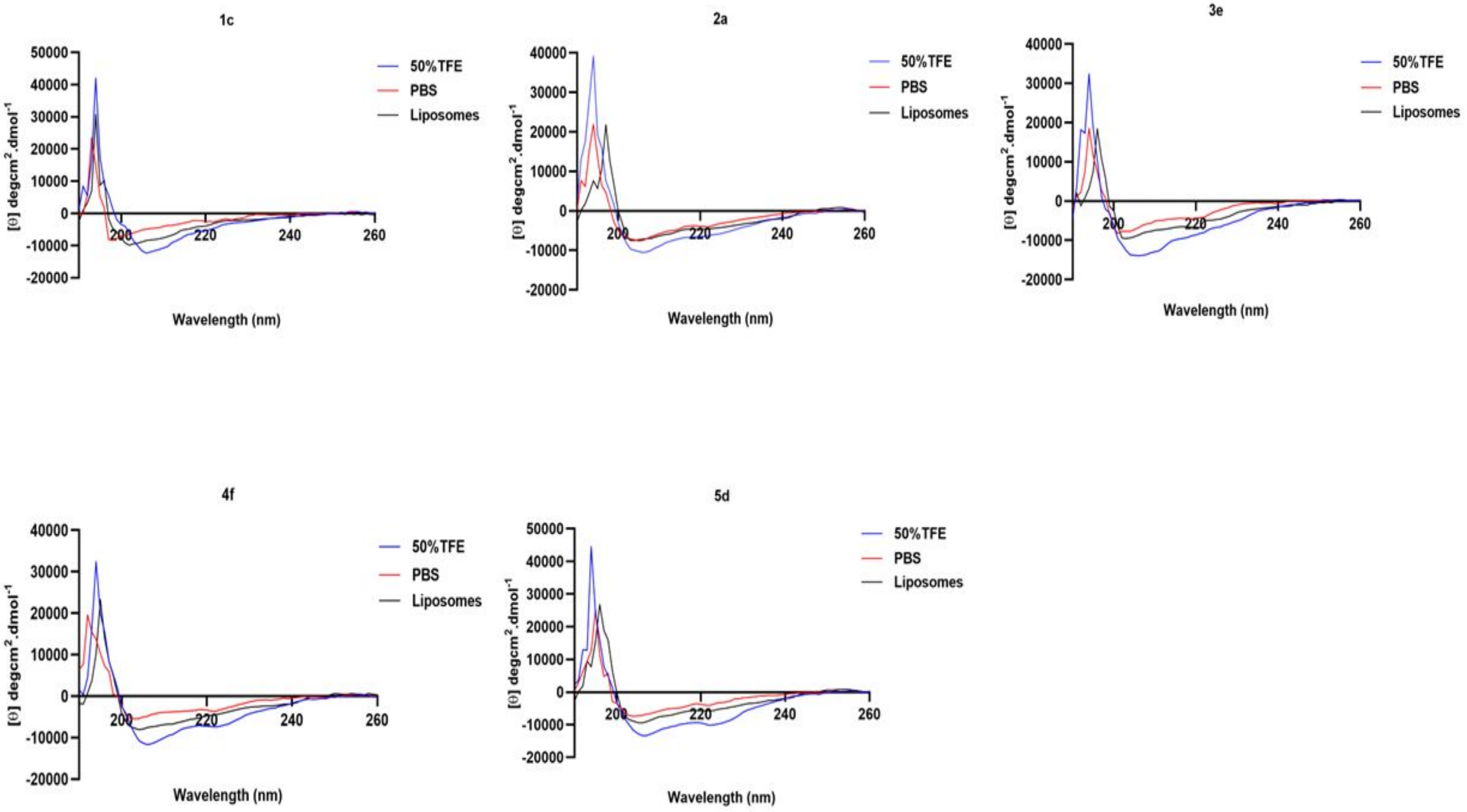

Figure S2. CD spectra of **1c**, **2a**, **3e**, **4f**, and **5d** recorded in PBS, TFE, and liposomes.

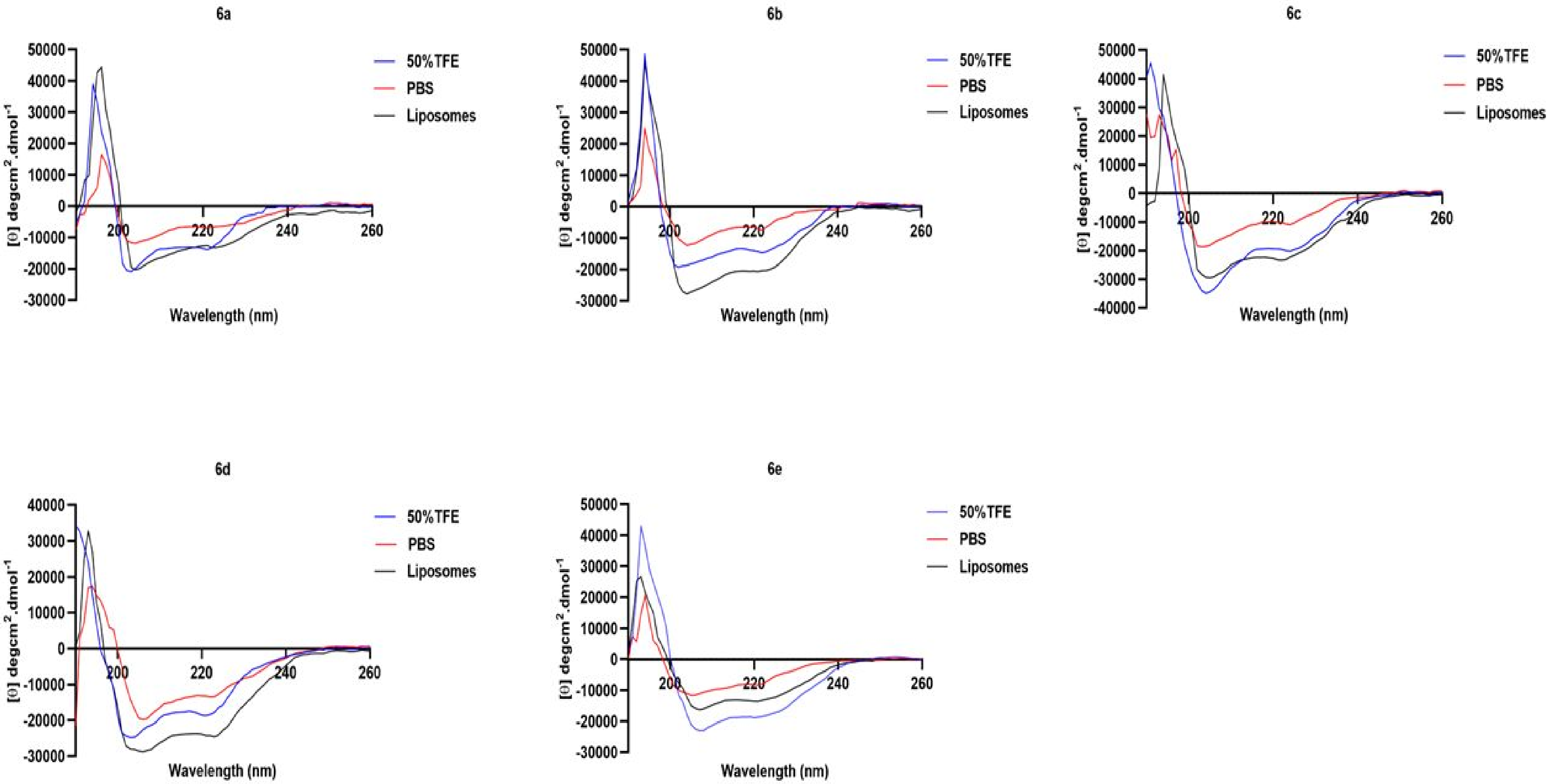

Figure S3. CD spectra of **6a**-**6e** recorded in PBS, TFE, and liposomes.

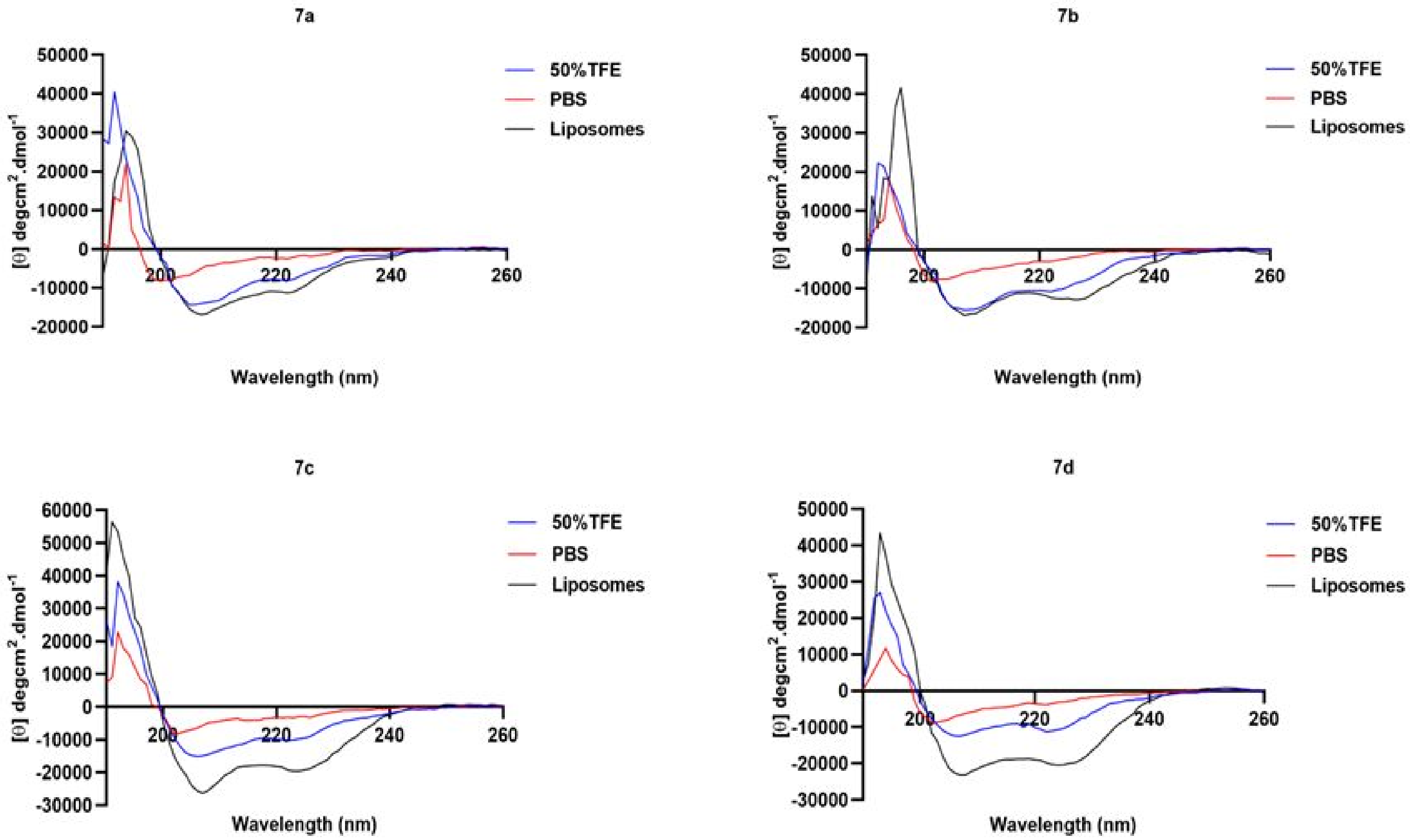

Figure S4. CD spectra of **7a-7d** recorded in PBS, TFE, and liposomes.

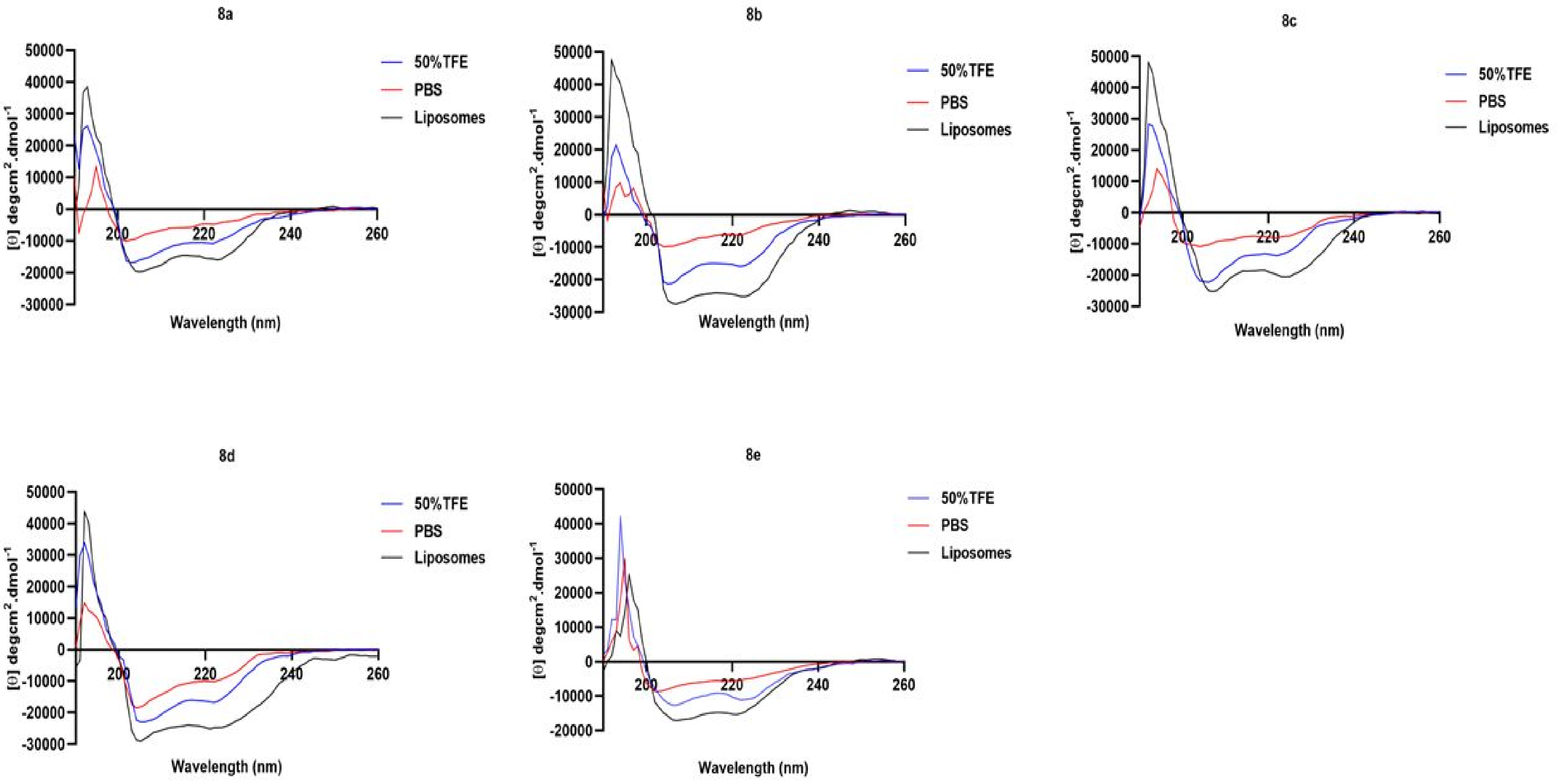

Figure S5. CD spectra of **8a**-**8e** recorded in PBS, TFE, and liposomes.

Table S1. RP-HPLC retention times and CD spectroscopy analysis of the selected peptides

Peptide	t_R (min)[a]	$[\theta]_{222}$[b]			$\Delta[\theta]_{222}$ induced by		% of helicity induced[c] by	
		PBS	TFE	Liposomes	TFE	Liposomes	TFE	Liposomes
**1c**	-	-2638	-4646	-3092	-2008	-454	43.22	14.68
**2a**	-	-4051	-6217	-4556	-2165	-505	34.83	11.08
**3e**	-	-4113	-8008	-4987	-3895	-874	48.63	17.53
**4f**	-	-3665	-7441	-4470	-3775	-805	50.73	18.01
**5d**	-	-4099	-10148	-5800	-6048	-1700	59.60	29.31
**6a**	30.42	-6737	-13346	-13245	-6608	-6508	49.51	49.13
**6b**	28.98	-7476	-14689	-20391	-7212	-12915	49.10	63.33
**6c**	34.61	-10947	-18280	-20250	-7332	-9303	40.11	45.93
**6d**	32.81	-13464	-18432	-24274	-4968	-10810	26.95	44.53
**6e**	29.87	-7968	-18419	-13181	-10451	-5213	56.74	39.55
**7a**	33.93	-2471	-8205	-11307	-5734	-8836	69.88	78.14
**7b**	32.23	-3016	-10800	-12345	-7784	-9328	72.06	75.56
**7c**	40.63	-3192	-10279	-19297	-7087	-16105	68.94	83.45
**7d**	38.75	-3826	-11272	-19755	-7446	-15929	66.05	80.63
**8a**	34.99	-4794	-10811	-15616	-6017	-10822	55.65	69.30
**8b**	33.41	-6387	-15887	-19313	-9499	-12926	59.79	66.92
**8c**	41.20	-8173	-13735	-19930	-5562	-11757	40.49	58.99
**8d**	39.25	-10222	-16759	-24786	-6537	-14564	39.00	58.75
**8e**	34.10	-5411	-11148	-15042	-5736	-9630	51.45	64.02

[a]t_R (min) denotes the retention time at 25°C by RP-HPLC.

[b]The mean residue molar ellipticity $[\theta]_{222}$ (mdeg cm^2/(dmol × res)) at 222 nm was measured at 25 °C in phosphate buffer saline (PBS, pH 7.0), in aqueous buffer containing 50% TFE (TFE), or in presence of bacterial membrane mimicking liposomes (peptide:lipid molar ratio 1:1.05).

[c]% helix induced is the increase in the molar ellipticity at 222 nm (in percentage) of a peptide in the presence of TFE or liposomes.

3. Antibacterial activity in the presence of salts and serum

Table S2. Antibacterial activities of lead peptides (**7a**, **7b**, **8a**, and **8b**) and antibiotics in the presence of FBS and various cationic salts[a]

Peptide	MIC[b] (µg/mL)						
	Control[c]	NaCl[d]	KCl[d]	NH_4Cl[d]	$MgCl_2$[d]	$CaCl_2$[d]	FBS[e] (25%)
MRSA (ATCC BAA-1556)							
**7a**	6.2	12.5	12.5	6.2	6.2	6.2	25
**7b**	6.2	25	12.5	12.5	6.2	12.5	25
**8a**	6.2	12.5	12.5	6.2	6.2	6.2	50
**8b**	3.1	12.5	6.2	6.2	6.2	6.2	12.5
Daptomycin	1.5	1.5	1.5	1.5	3.1	3.1	3.1
Ciprofloxacin	3.1	1.5	3.1	1.5	3.1	3.1	3.1
E. coli (ATCC 25922)							
**7a**	6.2	25	12.5	12.5	12.5	12.5	50
**7b**	6.2	25	25	25	12.5	12.5	50
**8a**	12.5	>50	25	25	50	25	>50
**8b**	6.2	25	12.5	12.5	25	25	25
Polymyxin B	0.7	0.7	0.7	0.7	1.5	0.7	3.1
Ciprofloxacin	0.7	0.7	0.7	0.7	0.7	0.7	0.7

[a]Results represent the MIC observed from three independent experiments performed in triplicate. [b]Minimum inhibitory concentrations (MIC) were determined as the lowest concentration of the peptides that inhibited bacteria growth. [c]Control represents the assay conducted in MH media without salt ions or serum. [d]The final concentrations of NaCl, KCl, NH_4Cl, $MgCl_2$, and $CaCl_2$ were 150 mM, 4.5 mM, 6 mM, 1 mM, and 2 mM, respectively. [e]Antibacterial activity determined in the presence of fetal bovine serum (FBS).

4. Cytotoxicity assay of daptomycin

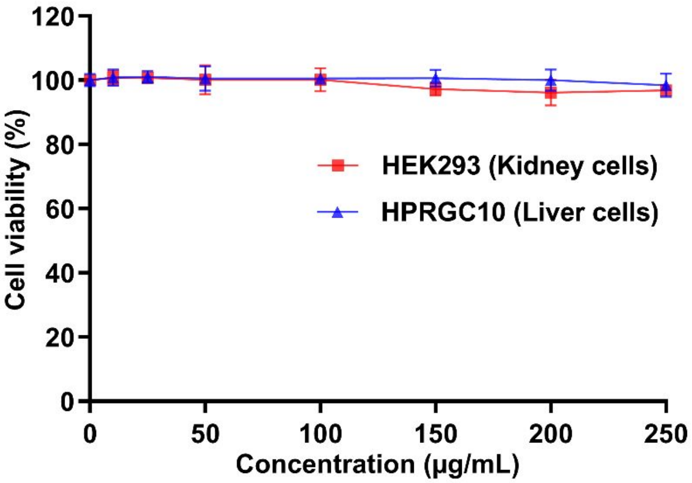

Figure S6. Cytotoxicity of daptomycin against human embryonic kidney (HEK293, red) and human liver cells (HPRGC10, blue). The results represent the data obtained from the experiments performed in triplicate (incubation for 24 h).

5. Bacterial Propagation

The bacterial strains employed in this study were obtained from the American Type Culture Collection (ATCC). Each strain was propagated as recommended by the ATCC, and each strain was stored as a frozen glycerol stock at -80°C. A bacterial colony was grown on the appropriate agar as indicated in Table S1 and was used to inoculate the appropriate broth. The culture was incubated at the appropriate conditions, as mentioned in Table S1. Following the incubation, the culture was diluted to an optical density 625 nm (OD_{625}) of 0.1 in cation adjusted Mueller Hinton Broth (CAMHB), which is equivalent to 1 × 10^8 CFU/mL. The culture was further diluted to 1 × 10^6 CFU/mL, which was used to conduct the MIC determination assay. For the *S. pneumoniae* strains, CAMHB + 2.5% lysed horse blood was required for the assay. The strains with their classification and properties are listed in Table S1.

Table S3. Description of the characteristics and growth conditions of various bacterial strains used in the study

Bacteria Strain	ATCC #	Classification	Properties	Growth Media/Agar	Incubation
Staphylococcus aureus	29213	Gram-positive Cocci	QC Strain	Trypticase Soy (TSB) broth	37 °C Aerobic
	33592		Methicillin resistant		
Enterococcus faecium	27270	Gram-positive Cocci	QC Strain	Brain Heart Infusion Broth (BHIB)	37 °C Aerobic
	700221		Vancomycin resistant		
Enterococcus faecalis	29212	Gram-positive Rod	QC Strain	Brain Heart Infusion Broth (BHIB)	37 °C Aerobic
	51575		Vancomycin resistant		
Streptococcus pneumoniae	49619	Gram-positive Diplococci	QC Strain	Trypticase Soy (TSB) broth	37 °C Aerobic
	700677		MDR		
Bacillus subtilis	6633	Gram-positive Rod	QC Strain	Luria-Bertani (LB) broth	37 °C Aerobic
Bacillus cereus	13061	Gram-positive Rod	QC Strain	Luria-Bertani (LB) broth	37 °C Aerobic
Escherichia coli	25922	Gram-negative Rod	QC Strain	Nutrient Broth (NB)	+37 °C Aerobic
	BAA-2452		NDM-1 and Carbapenem resistant		
Klebsiella pneumonia	13883	Gram-negative Rod	QC Strain	Nutrient Broth (NB)	+37 °C Aerobic
	BAA-2470		Carbapenem resistant		
Acinetobacter baumannii	BAA1605	Gram-negative Rod	MDR	Nutrient Broth (NB)	+37 °C Aerobic
Pseudomonas aeruginosa	10145	Gram-negative Rod	QC Strain	Luria-Bertani (LB) broth	+37°C Aerobic
	BAA-1744		Imipenem resistant		

6. Flow Cytometry (FACS) Analysis

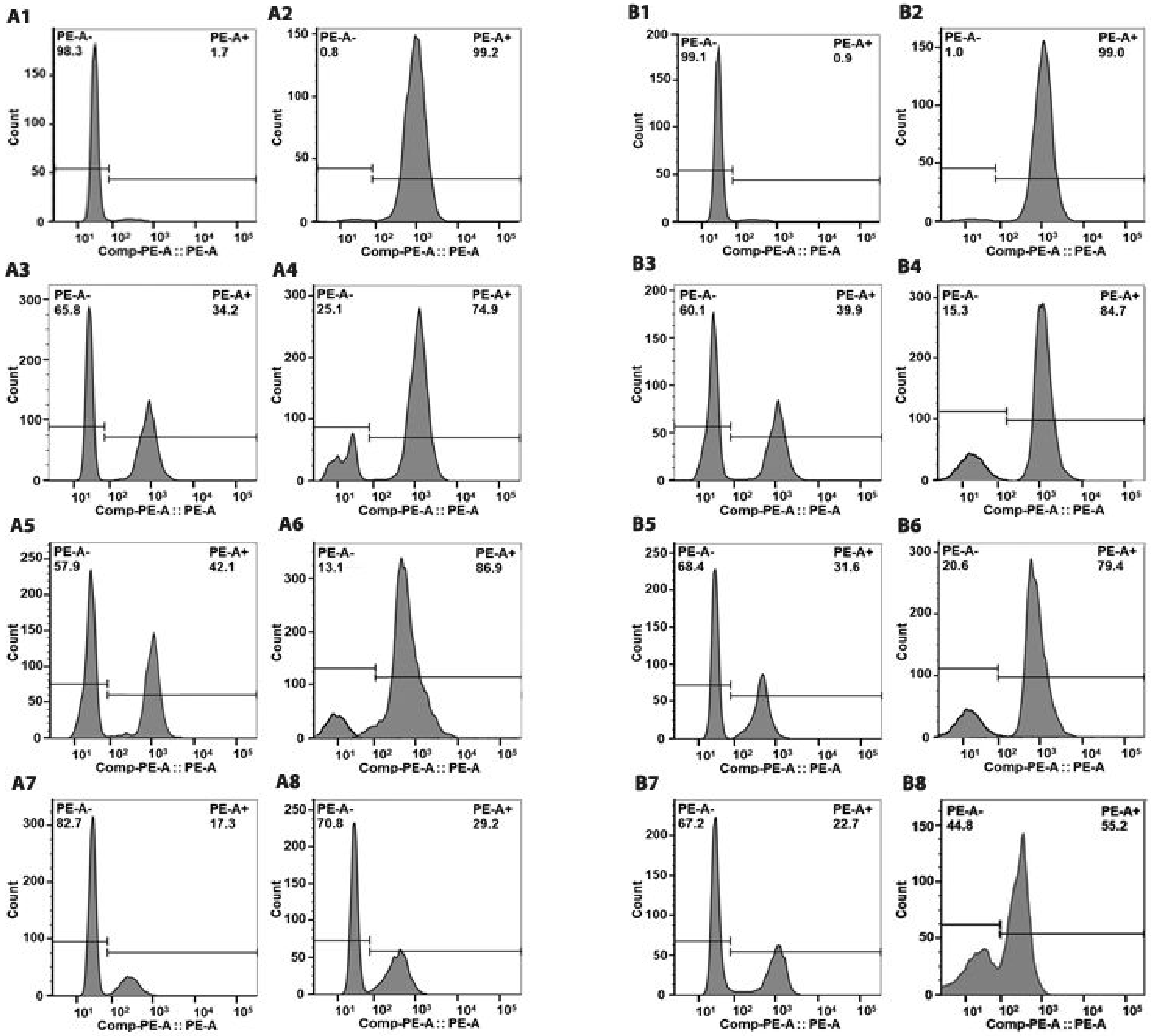

Figure S7. Flow cytometric analysis of MRSA (A1-A8) and *E. coli* (B1-B8) treated with peptides **8a** and **8b** and standard antibiotics (daptomycin and polymyxin B) at MIC and 4×MIC. The increments of the fluorescence signal represent PI uptake resulting from test peptide and antibiotic treatment. (A1 and B1) No treatment (negative control); (A2 and B2) 10% Triton X-100 (positive control); (A3 and B3) **8a** (MIC); (A4 and B4) **8a** (4×MIC); (A5 and B5) **8b** (MIC); (A6 and B6) **8b** (4×MIC); (A7) daptomycin (MIC); (A8) daptomycin (4×MIC); (B7) polymyxin B (MIC); (B8) polymyxin B (4×MIC).

7. Purity Analysis

The purity of all synthesized peptides was determined using reverse phase analytical HPLC (Shimadzu; LC-20ADXR). Below mentioned are the conditions used to conduct the purity analysis.

Method 1

Column: Phenomenex (Luna), 4 um, C18, 150 × 4.6 mm

Flow rate: 0.5 ml/min

Mobile Phase: Buffer A-Water containing 0.1% TFA,
Buffer B-Acetonitrile containing 0.1% TFA

Gradient

Time (min)	Buffer B (%)
0.01	5
2	5
25	70
27	95
32	95
32.01	5
38	5

HPLC Chromatograms of all the synthesized peptides (1a-8e)

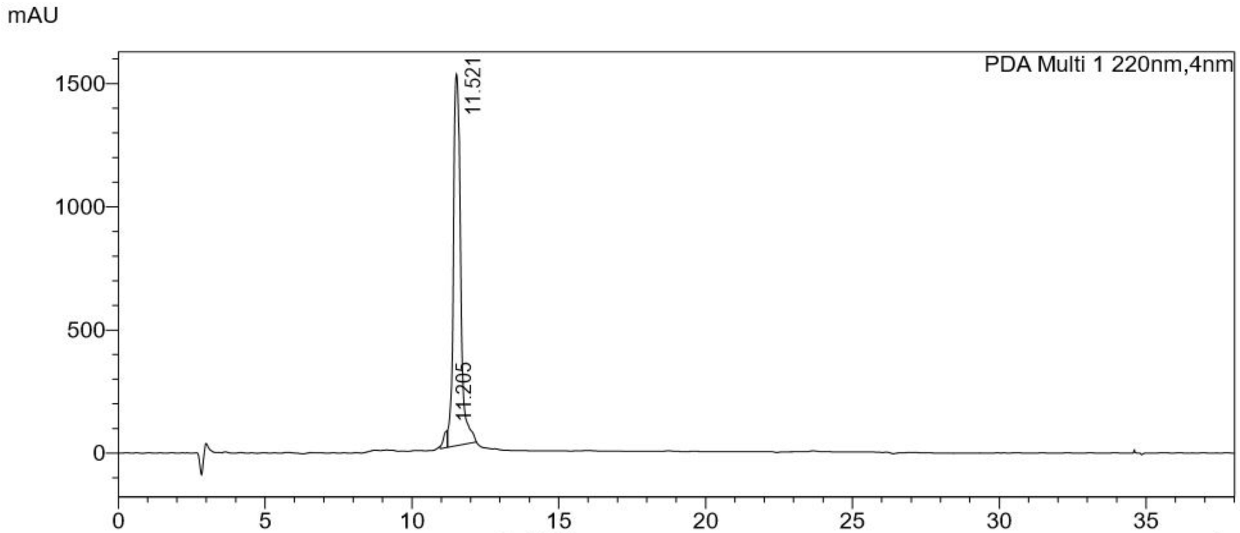

Peak Table

PDA Ch1 220nm

Peak#	Ret. Time	Area	Height	Area%
1	11.205	667229	67268	2.558
2	11.521	25417984	1507351	97.442
Total		26085213	1574619	100.000

Figure S8. HPLC chromatogram of **1a**.

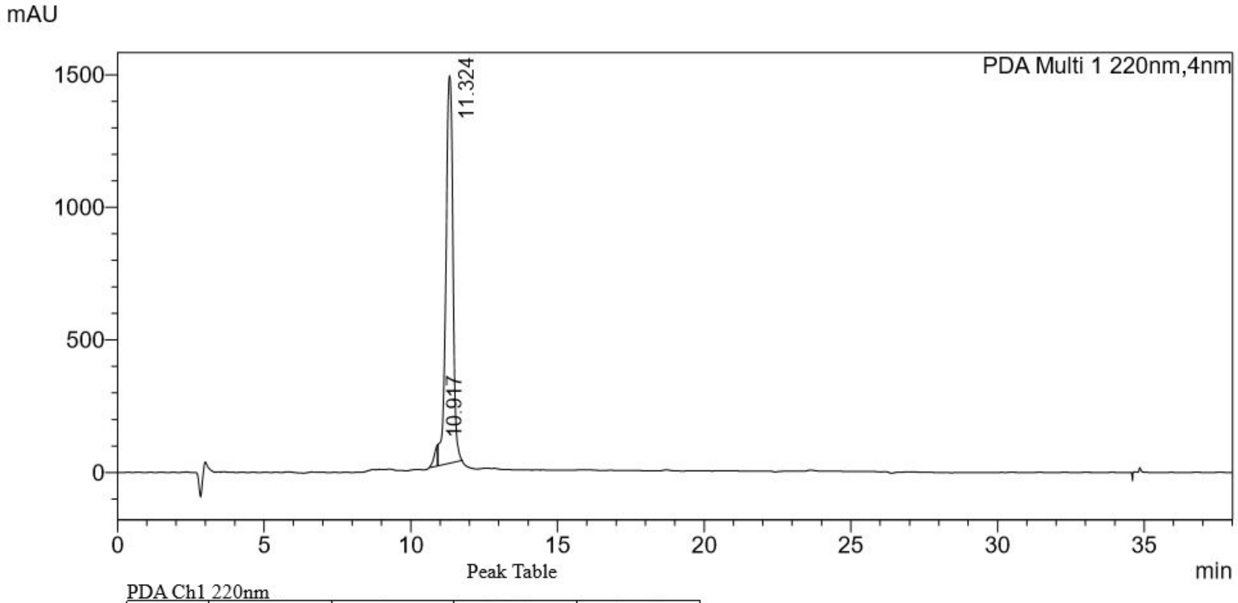

Peak Table

PDA Ch1 220nm

Peak#	Ret. Time	Area	Height	Area%
1	10.917	648633	78308	2.567
2	11.324	24624201	1458855	97.433
Total		25272833	1537163	100.000

Figure S9. HPLC chromatogram of **1b**.

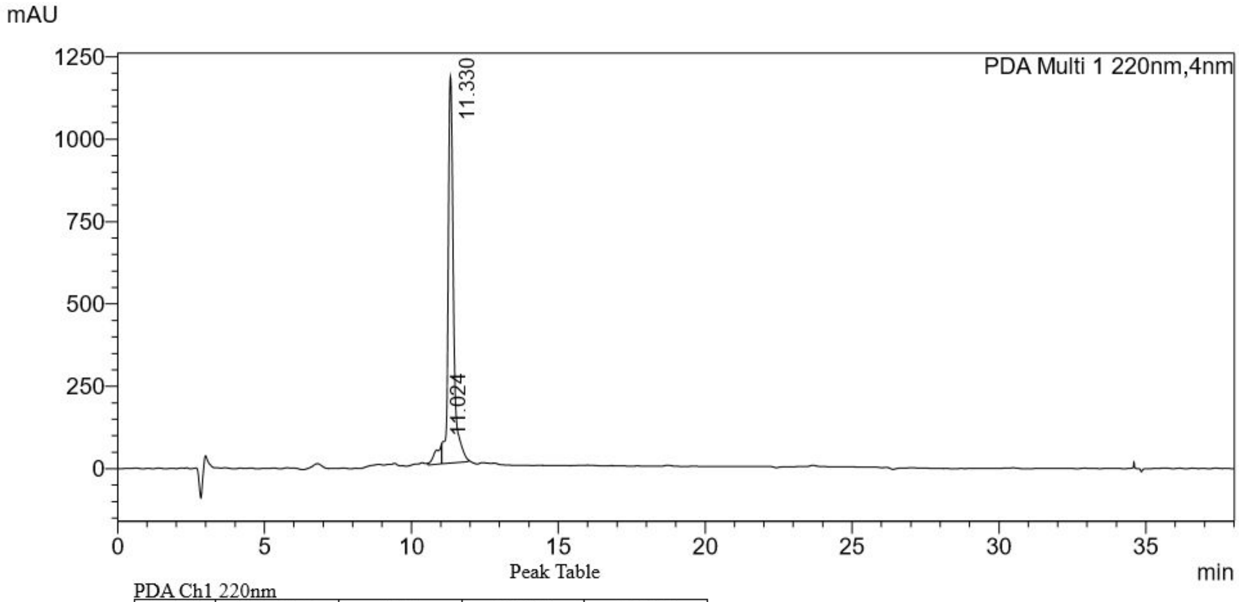

Peak Table

PDA Ch1 220nm

Peak#	Ret. Time	Area	Height	Area%
1	11.024	868777	59320	5.472
2	11.330	15008950	1172681	94.528
Total		15877727	1232001	100.000

Figure S10. HPLC chromatogram of **1c**.

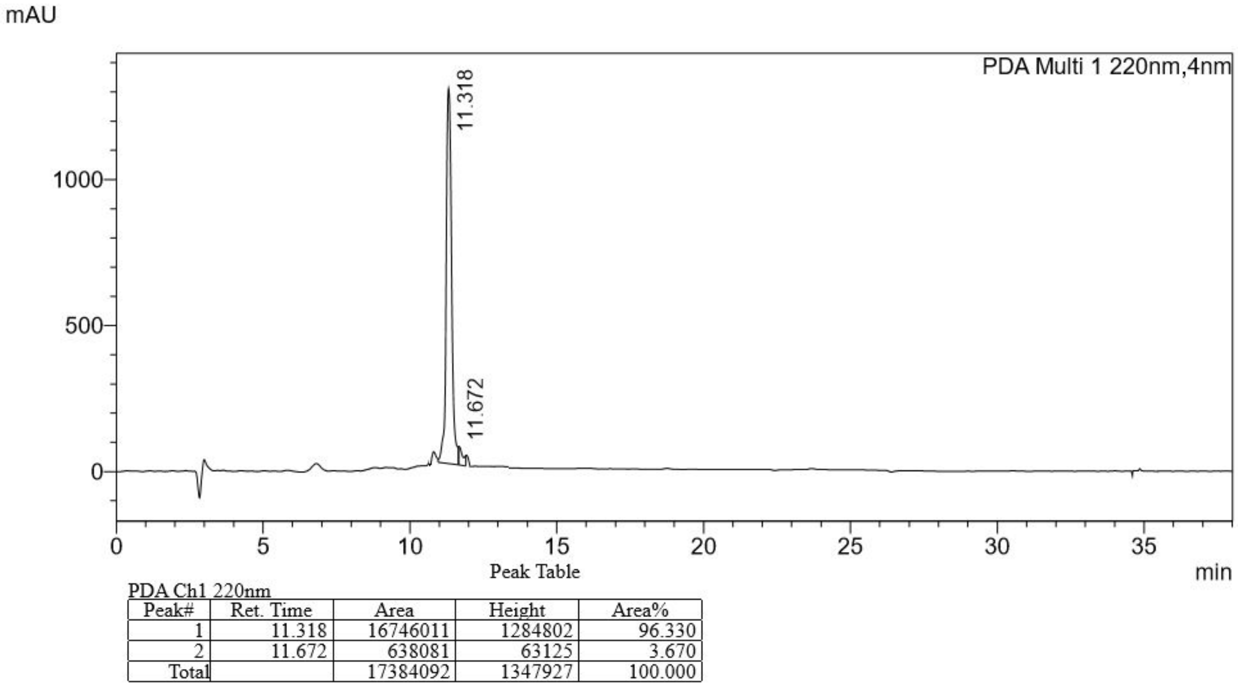

Peak Table

PDA Ch1 220nm

Peak#	Ret. Time	Area	Height	Area%
1	11.318	16746011	1284802	96.330
2	11.672	638081	63125	3.670
Total		17384092	1347927	100.000

Figure S11. HPLC chromatogram of **1d**.

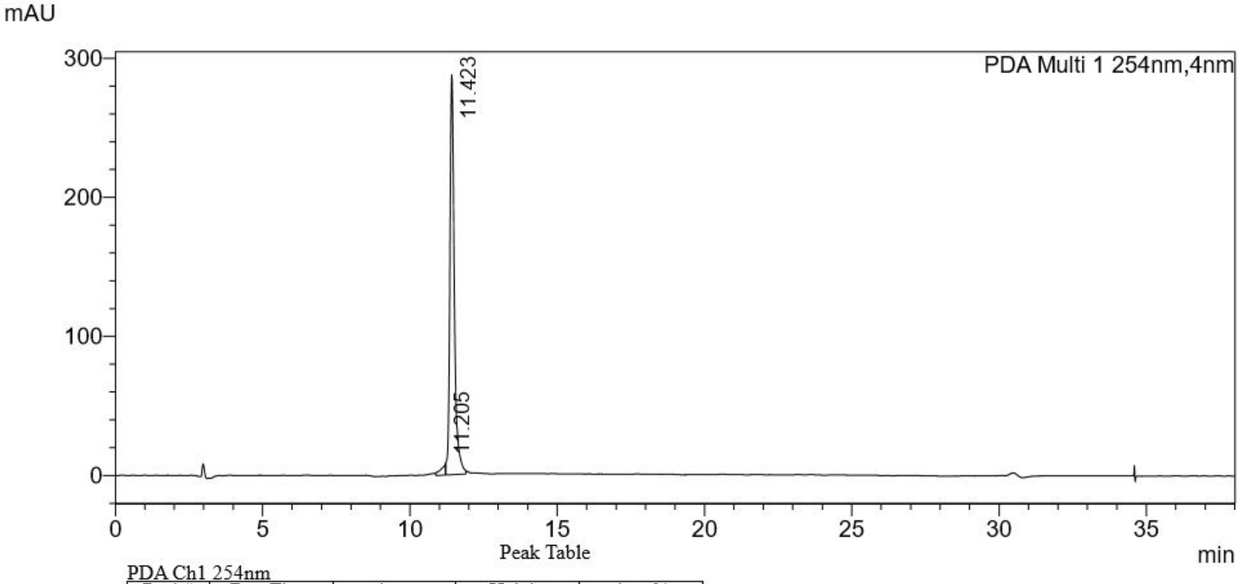

Peak Table

PDA Ch1 254nm

Peak#	Ret. Time	Area	Height	Area%
1	11.205	90017	8058	2.883
2	11.423	3032760	287760	97.117
Total		3122777	295818	100.000

Figure S12. HPLC chromatogram of **1e**.

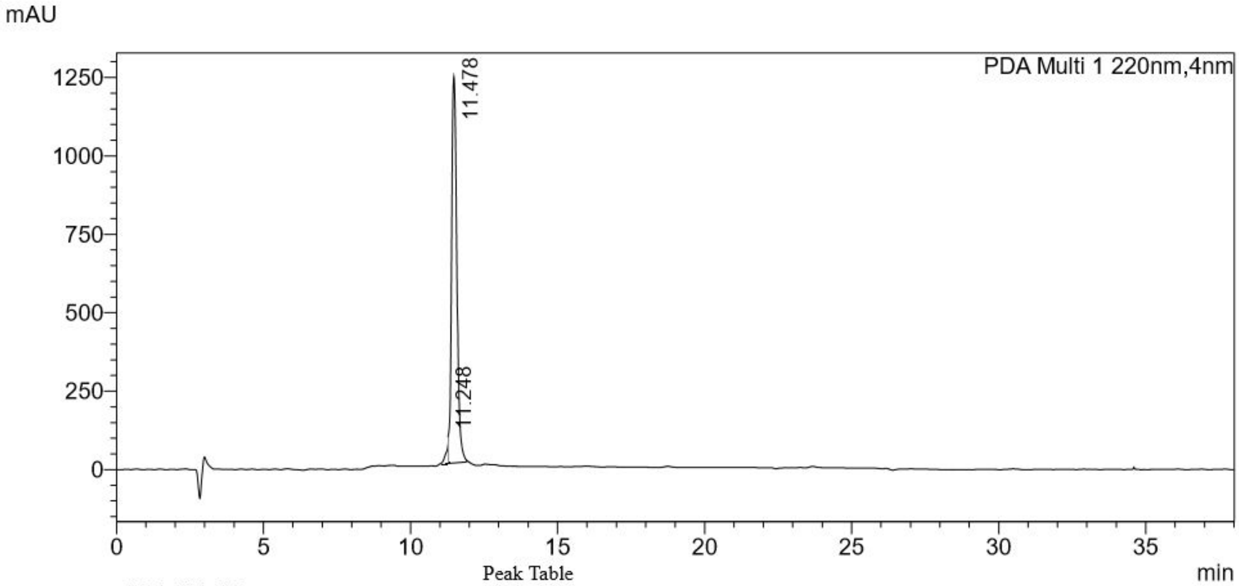

Peak Table

PDA Ch1 220nm

Peak#	Ret. Time	Area	Height	Area%
1	11.248	477598	74020	3.001
2	11.478	15435295	1231182	96.999
Total		15912893	1305202	100.000

Figure S13. HPLC chromatogram of **2a**.

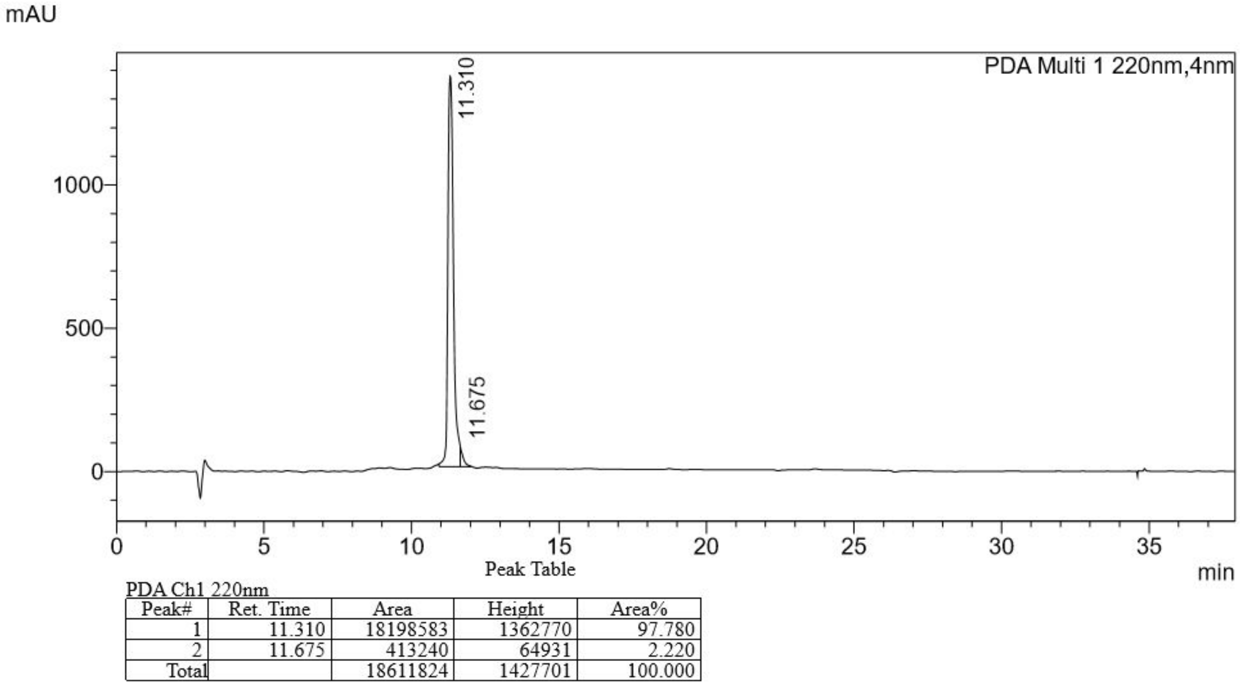

Peak Table

PDA Ch1 220nm

Peak#	Ret. Time	Area	Height	Area%
1	11.310	18198583	1362770	97.780
2	11.675	413240	64931	2.220
Total		18611824	1427701	100.000

Figure S14. HPLC chromatogram of **2b**.

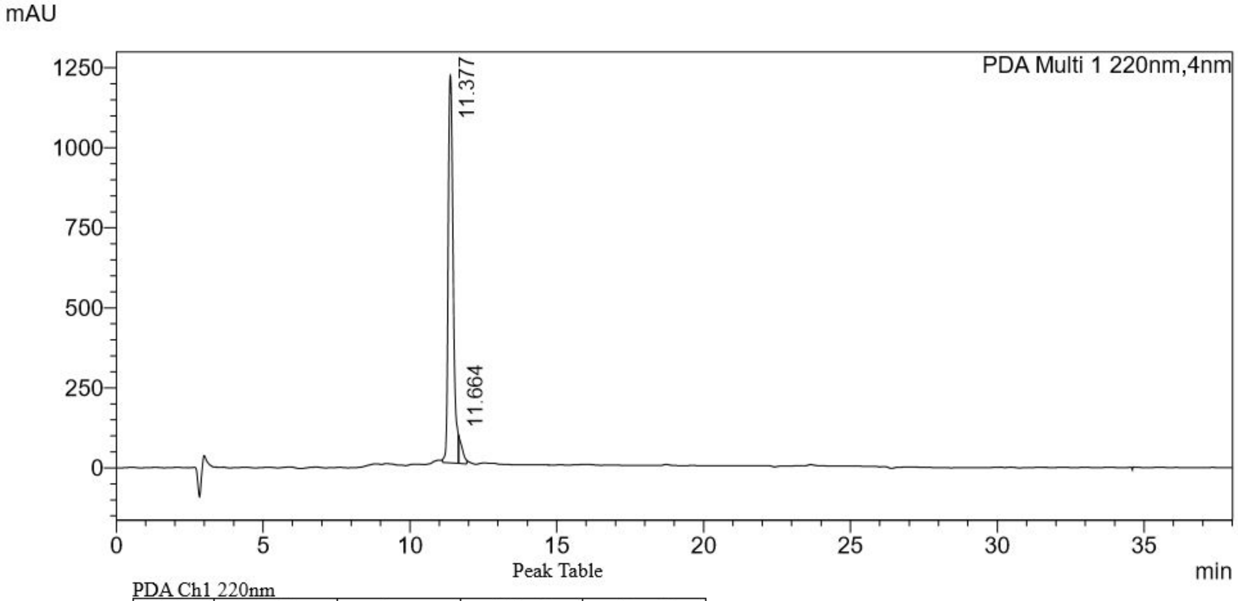

Peak Table

PDA Ch1 220nm

Peak#	Ret. Time	Area	Height	Area%
1	11.377	14480420	1209891	95.094
2	11.664	747071	85242	4.906
Total		15227491	1295133	100.000

Figure S15. HPLC chromatogram of **2c**.

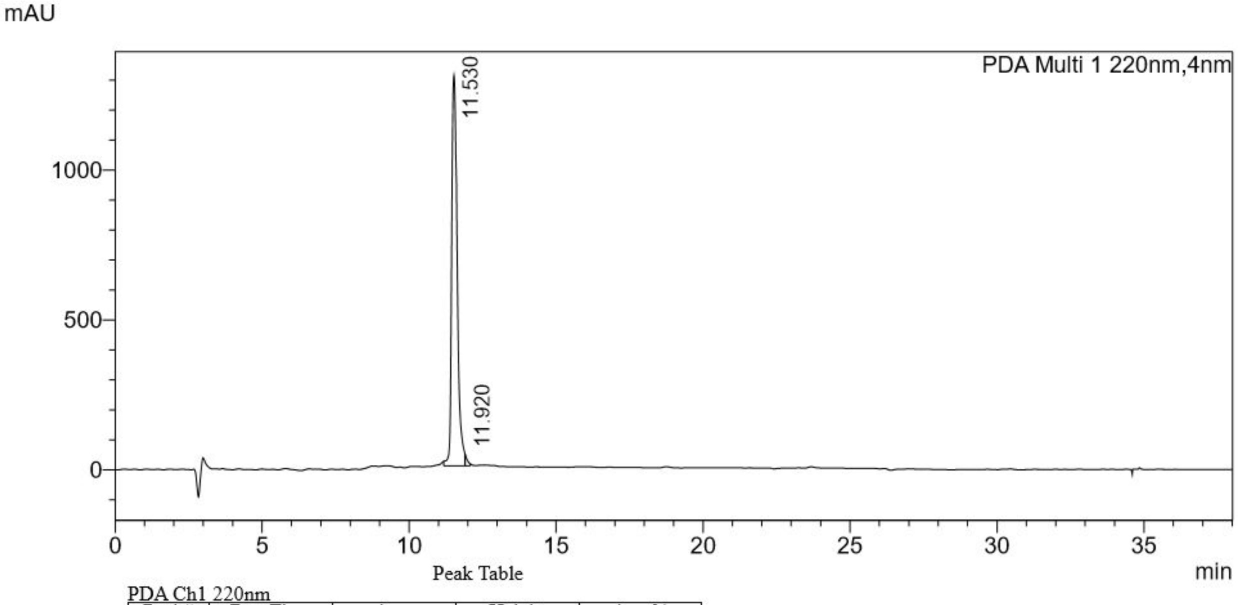

Peak Table

PDA Ch1 220nm

Peak#	Ret. Time	Area	Height	Area%
1	11.530	16835769	1303226	98.819
2	11.920	201141	36201	1.181
Total		17036909	1339426	100.000

Figure S16. HPLC chromatogram of **2d**.

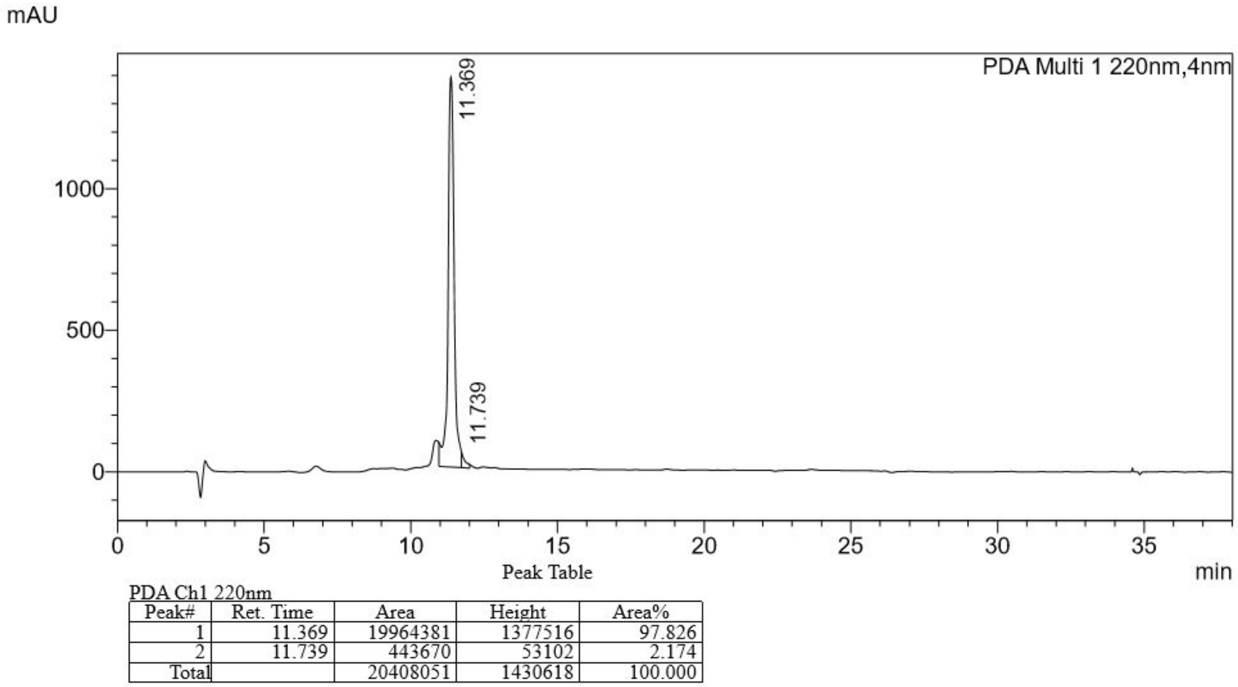

Peak Table

PDA Ch1 220nm

Peak#	Ret. Time	Area	Height	Area%
1	11.369	19964381	1377516	97.826
2	11.739	443670	53102	2.174
Total		20408051	1430618	100.000

Figure S17. HPLC chromatogram of **2e**.

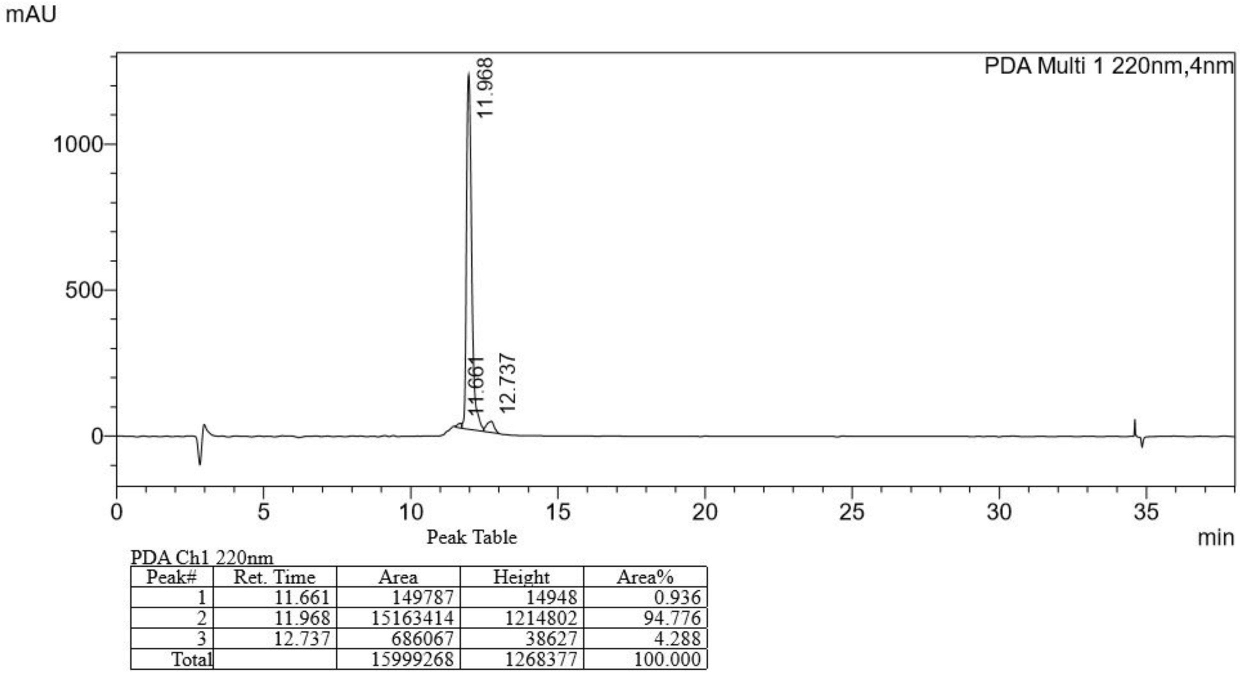

Peak Table

PDA Ch1 220nm

Peak#	Ret. Time	Area	Height	Area%
1	11.661	149787	14948	0.936
2	11.968	15163414	1214802	94.776
3	12.737	686067	38627	4.288
Total		15999268	1268377	100.000

Figure S18. HPLC chromatogram of **3a**.

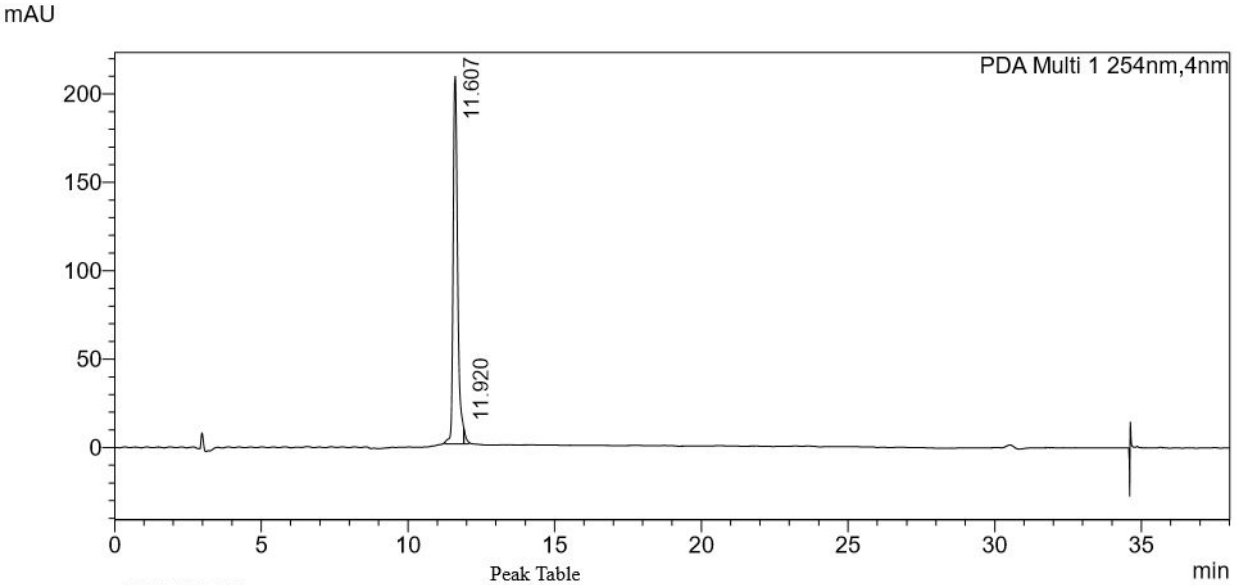

Peak Table

PDA Ch1 254nm

Peak#	Ret. Time	Area	Height	Area%
1	11.607	2123835	208100	98.262
2	11.920	37566	7929	1.738
Total		2161401	216029	100.000

Figure S19. HPLC chromatogram of **3b**.

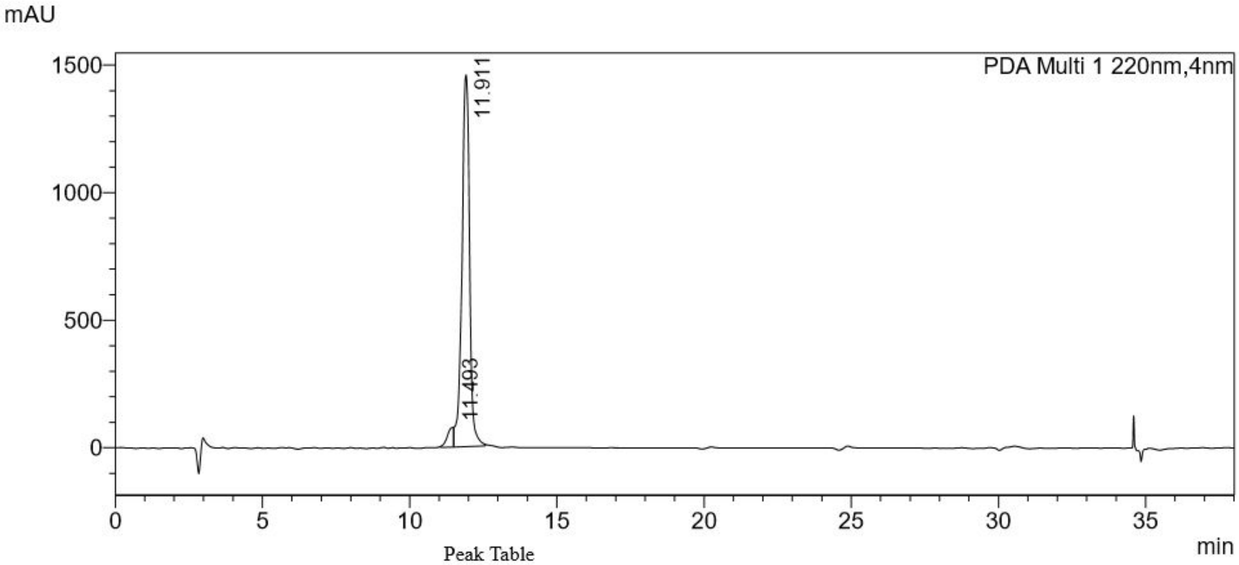

Peak Table

PDA Ch1 220nm

Peak#	Ret. Time	Area	Height	Area%
1	11.493	1158872	77241	4.180
2	11.911	26564577	1455287	95.820
Total		27723448	1532528	100.000

Figure S20. HPLC chromatogram of **3c**.

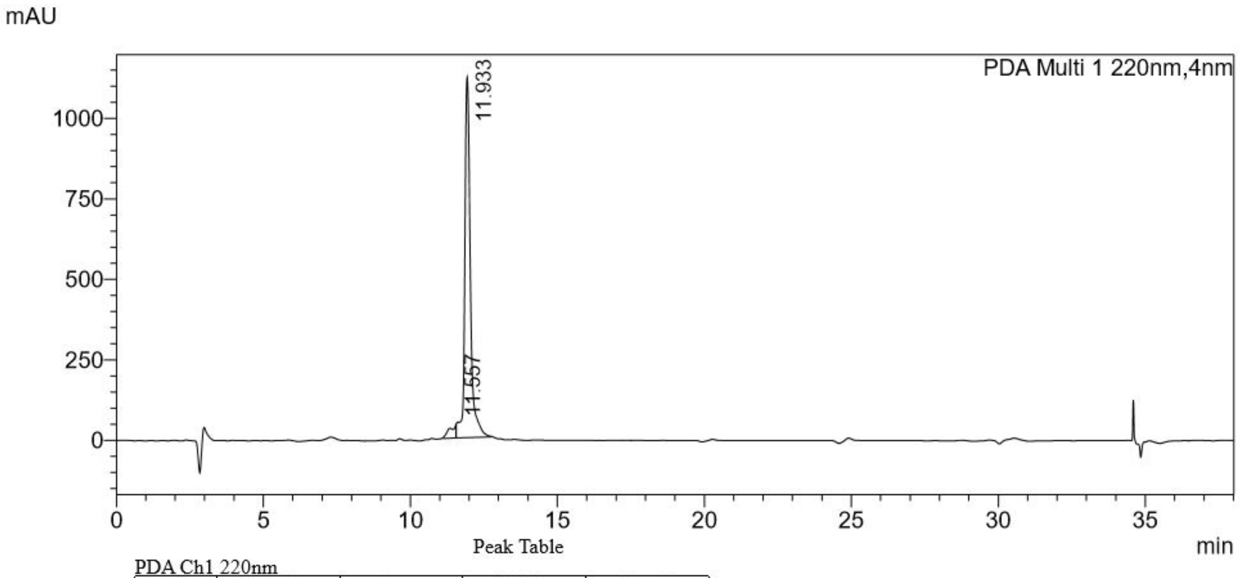

PDA Ch1 220nm

Peak#	Ret. Time	Area	Height	Area%
1	11.557	649507	41078	4.160
2	11.933	14963439	1120366	95.840
Total		15612946	1161444	100.000

Figure S21. HPLC chromatogram of **3d**.

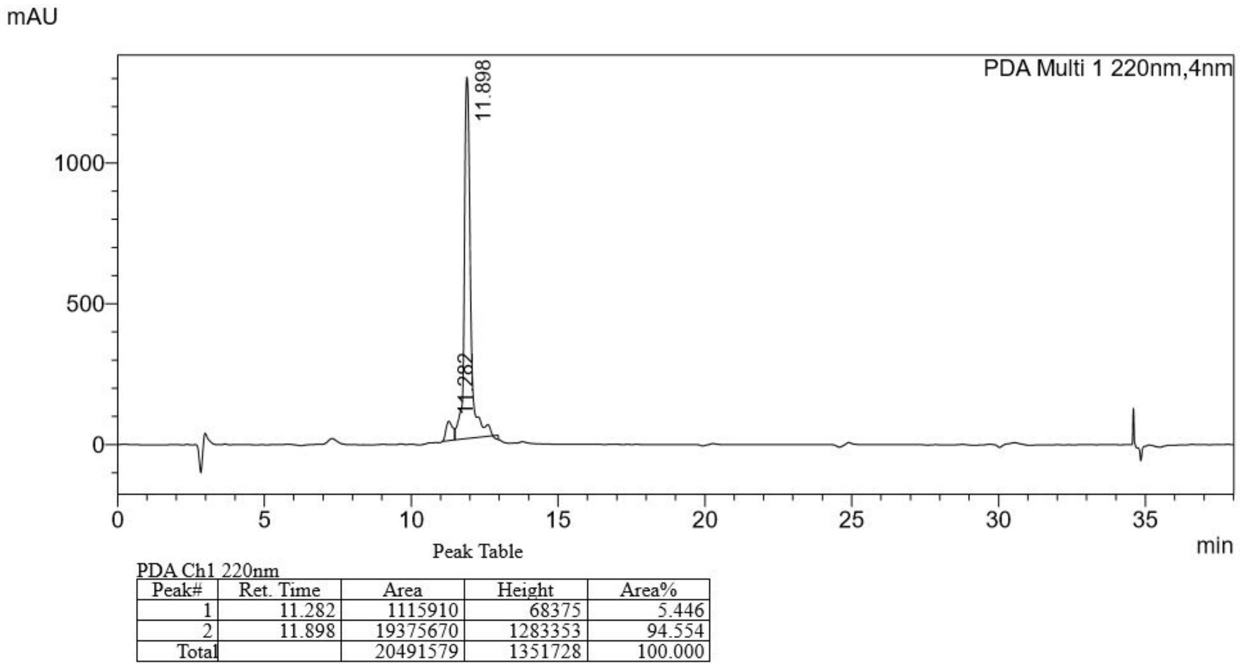

PDA Ch1 220nm

Peak#	Ret. Time	Area	Height	Area%
1	11.282	1115910	68375	5.446
2	11.898	19375670	1283353	94.554
Total		20491579	1351728	100.000

Figure S22. HPLC chromatogram of **3e**.

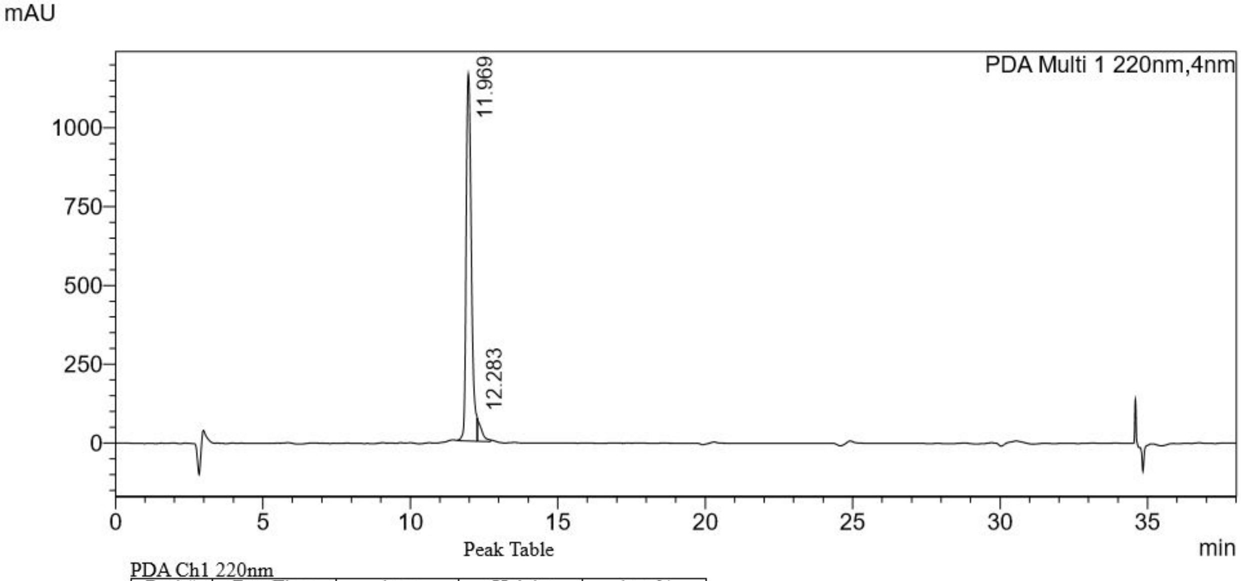

Peak Table

PDA Ch1 220nm

Peak#	Ret. Time	Area	Height	Area%
1	11.969	14567389	1163890	95.466
2	12.283	691832	69010	4.534
Total		15259221	1232900	100.000

Figure S23. HPLC chromatogram of **3f**.

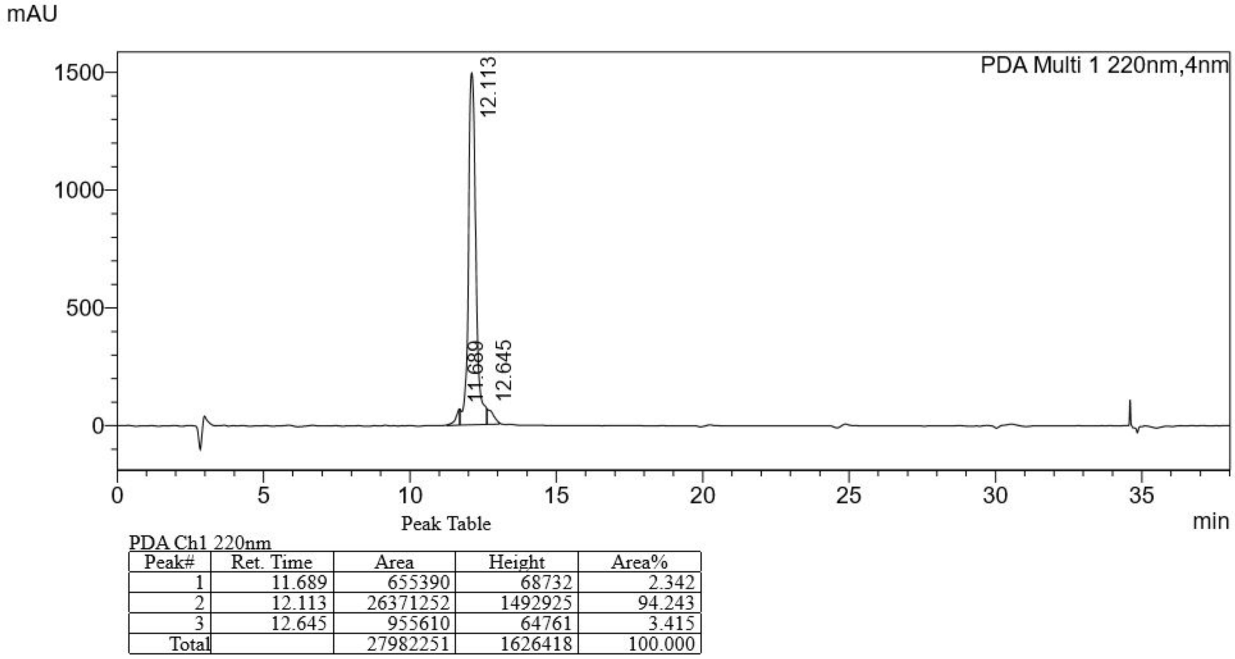

Peak Table

PDA Ch1 220nm

Peak#	Ret. Time	Area	Height	Area%
1	11.689	655390	68732	2.342
2	12.113	26371252	1492925	94.243
3	12.645	955610	64761	3.415
Total		27982251	1626418	100.000

Figure S24. HPLC chromatogram of **4a**.

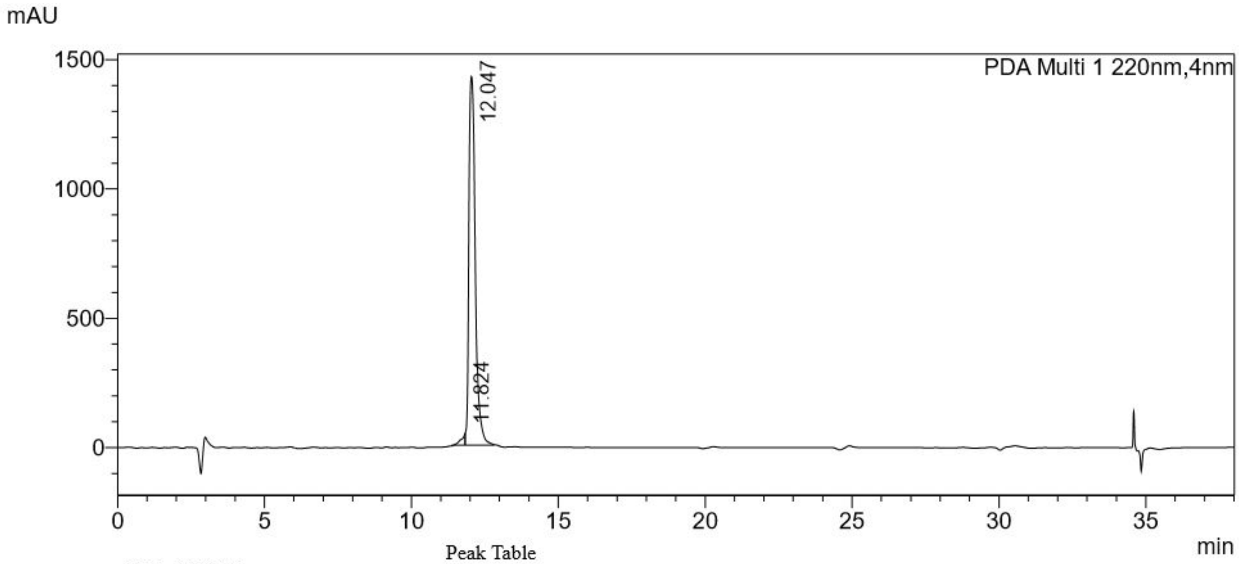

Peak Table

PDA Ch1 220nm

Peak#	Ret. Time	Area	Height	Area%
1	11.824	419076	43158	1.885
2	12.047	21809995	1426752	98.115
Total		22229071	1469910	100.000

Figure S25. HPLC chromatogram of **4b**.

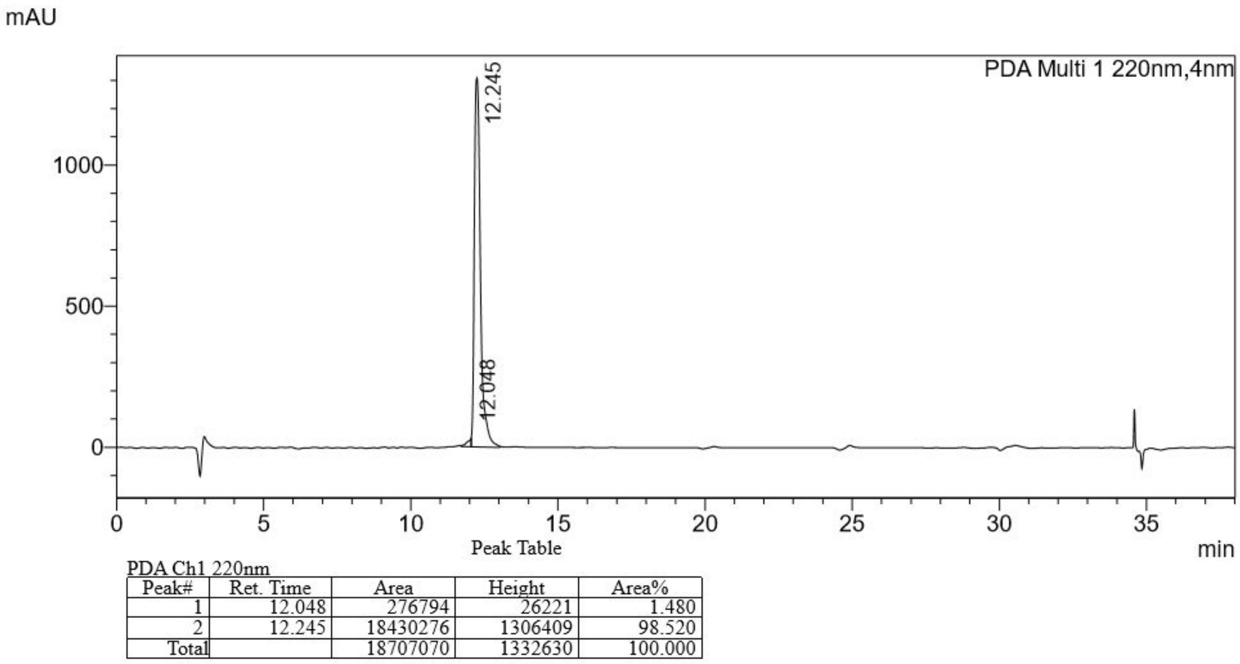

Peak Table

PDA Ch1 220nm

Peak#	Ret. Time	Area	Height	Area%
1	12.048	276794	26221	1.480
2	12.245	18430276	1306409	98.520
Total		18707070	1332630	100.000

Figure S26. HPLC chromatogram of **4c**.

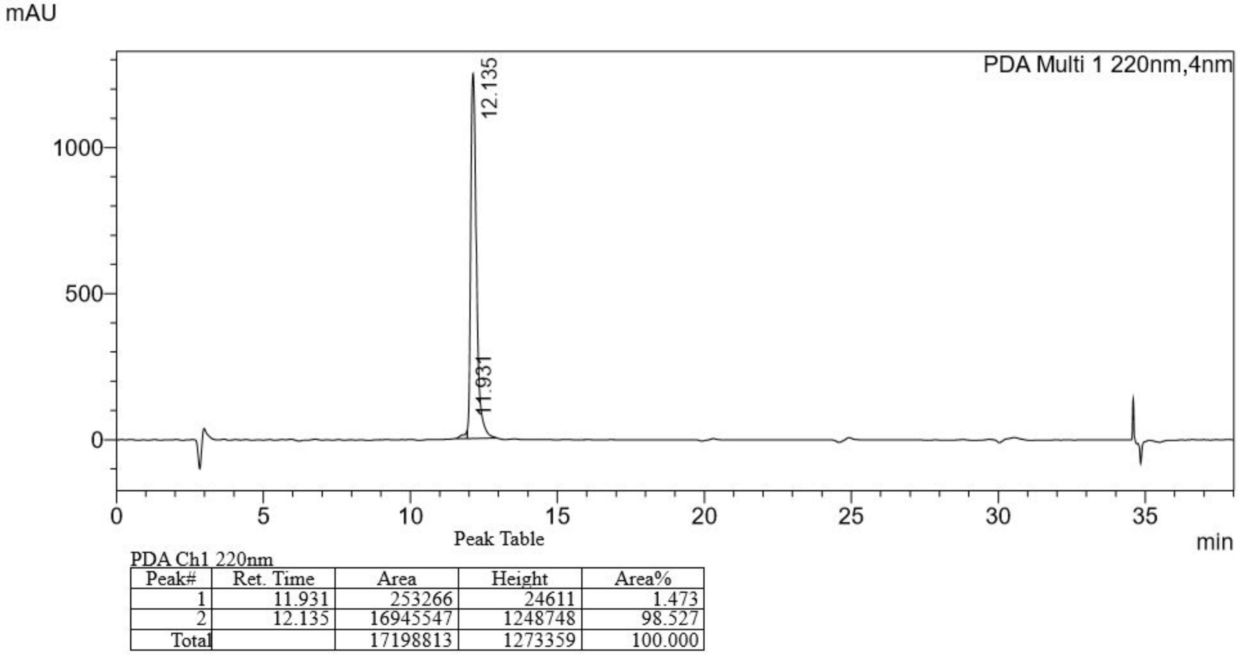

Peak Table

PDA Ch1 220nm

Peak#	Ret. Time	Area	Height	Area%
1	11.931	253266	24611	1.473
2	12.135	16945547	1248748	98.527
Total		17198813	1273359	100.000

Figure S27. HPLC chromatogram of **4d**.

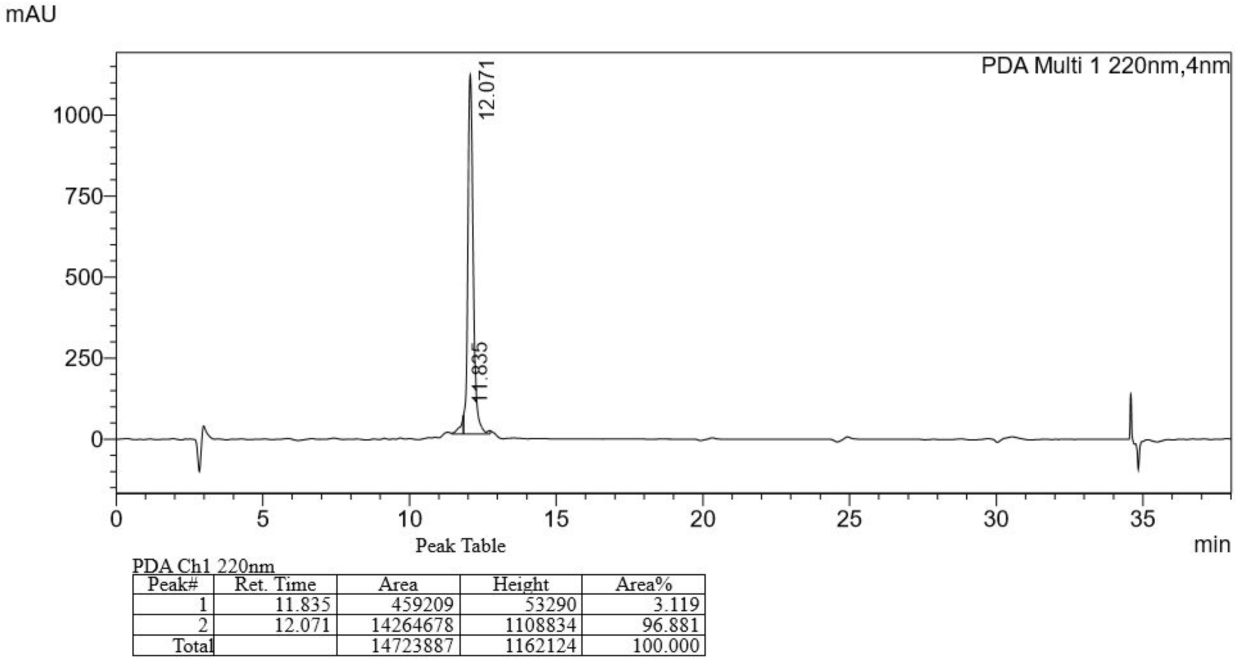

Peak Table

PDA Ch1 220nm

Peak#	Ret. Time	Area	Height	Area%
1	11.835	459209	53290	3.119
2	12.071	14264678	1108834	96.881
Total		14723887	1162124	100.000

Figure S28. HPLC chromatogram of **4e**.

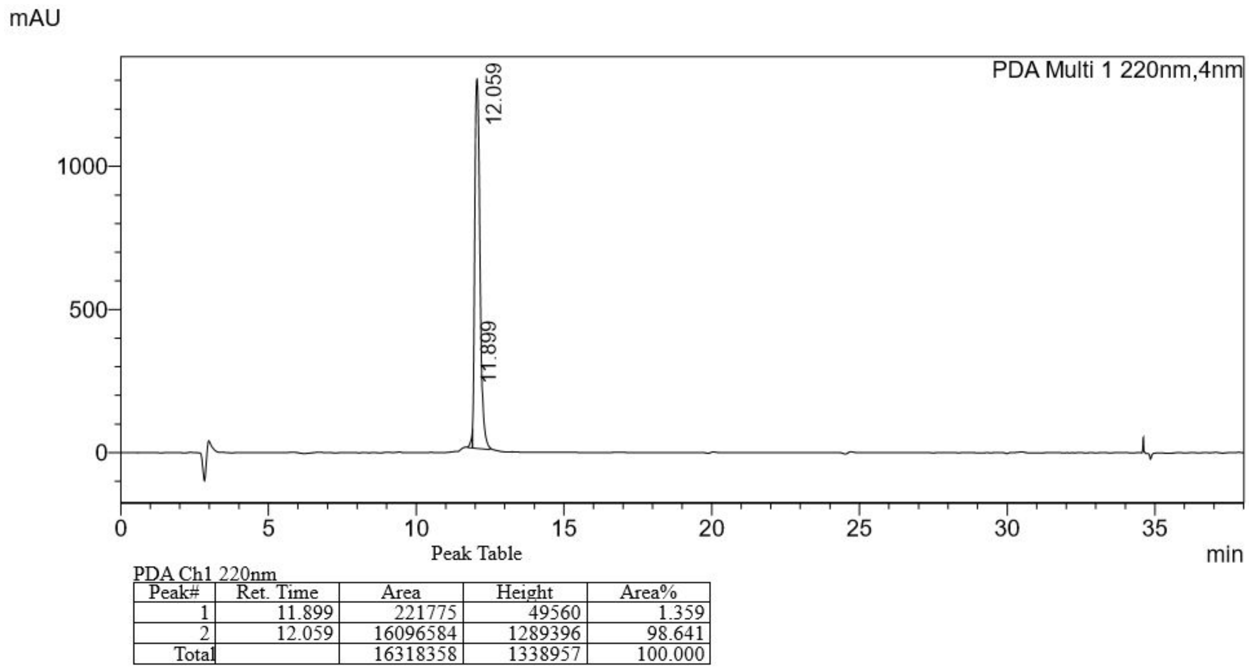

PDA Ch1 220nm

Peak#	Ret. Time	Area	Height	Area%
1	11.899	221775	49560	1.359
2	12.059	16096584	1289396	98.641
Total		16318358	1338957	100.000

Figure S29. HPLC chromatogram of **4f**.

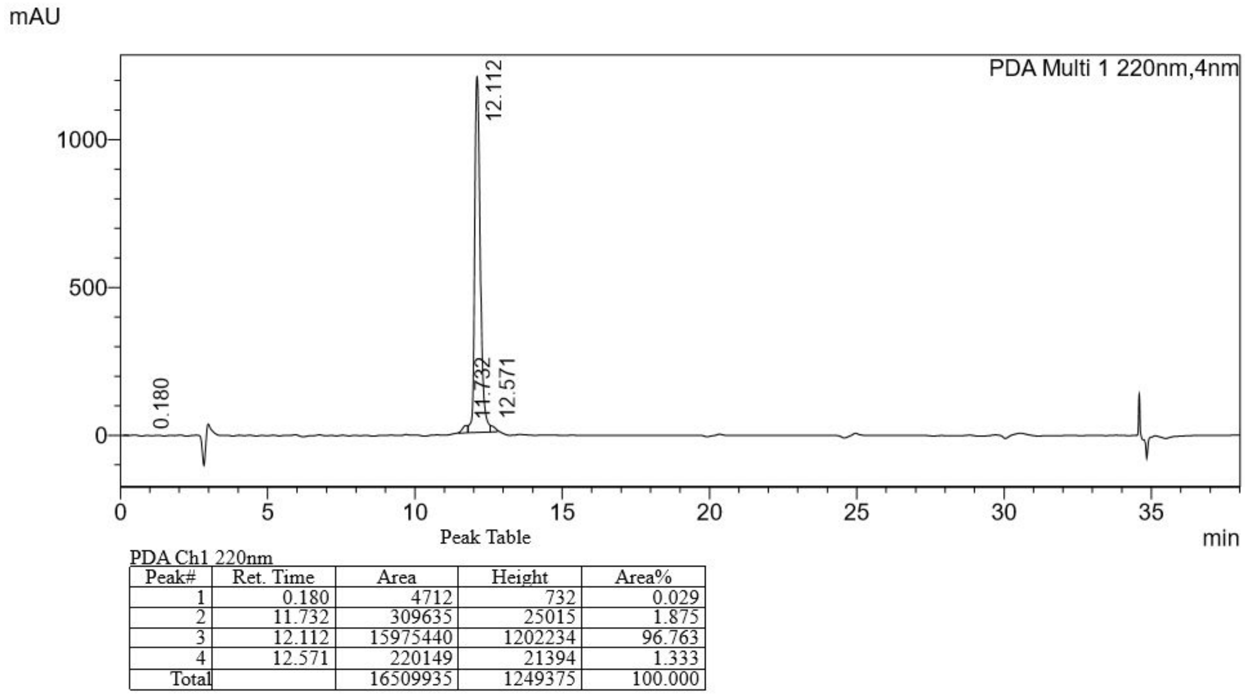

PDA Ch1 220nm

Peak#	Ret. Time	Area	Height	Area%
1	0.180	4712	732	0.029
2	11.732	309635	25015	1.875
3	12.112	15975440	1202234	96.763
4	12.571	220149	21394	1.333
Total		16509935	1249375	100.000

Figure S30. HPLC chromatogram of **4g**.

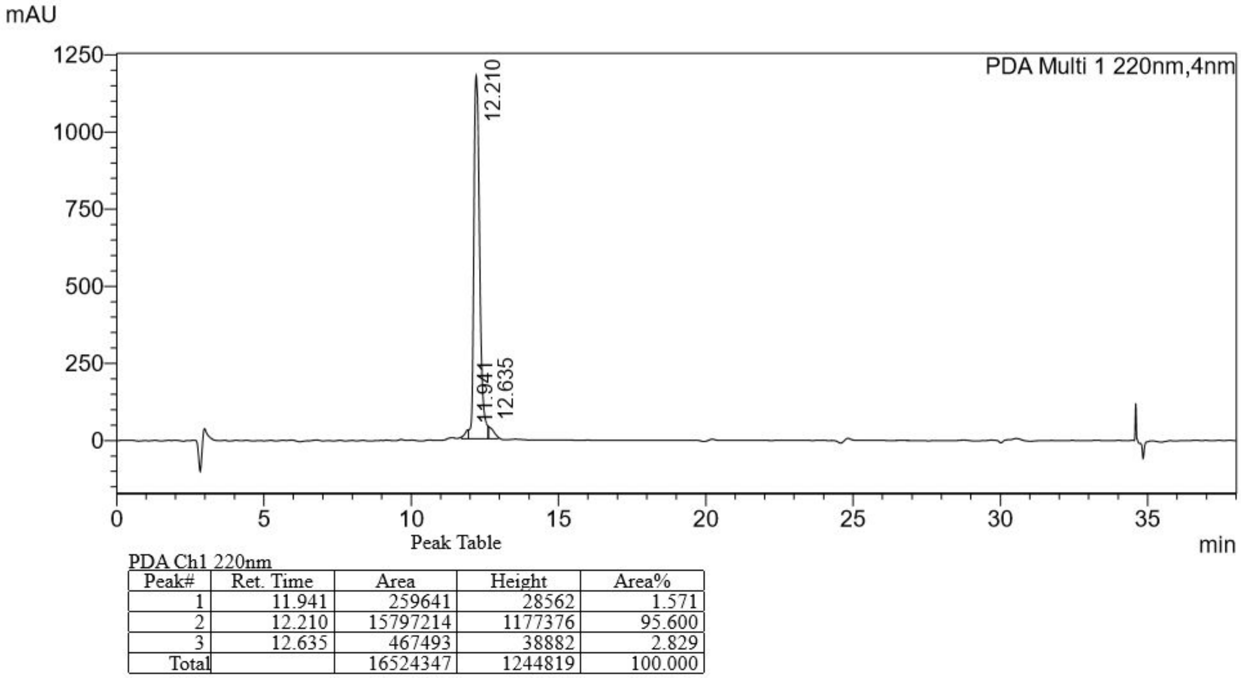

Peak Table

PDA Ch1 220nm

Peak#	Ret. Time	Area	Height	Area%
1	11.941	259641	28562	1.571
2	12.210	15797214	1177376	95.600
3	12.635	467493	38882	2.829
Total		16524347	1244819	100.000

Figure S31. HPLC chromatogram of **4h**.

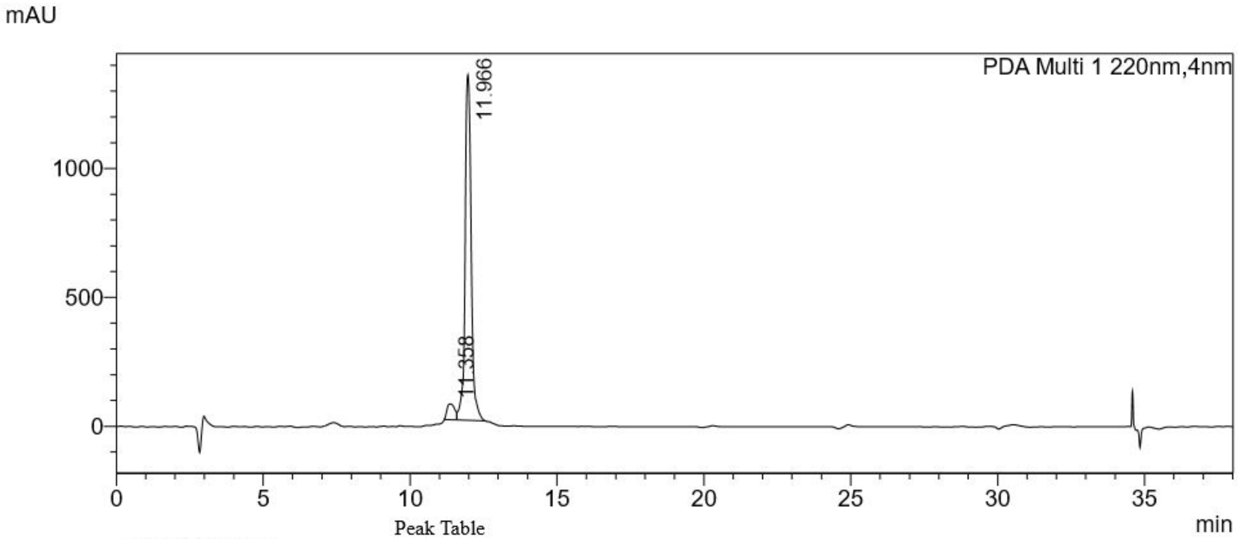

Peak Table

PDA Ch1 220nm

Peak#	Ret. Time	Area	Height	Area%
1	11.358	1148436	61001	5.536
2	11.966	19597208	1340564	94.464
Total		20745644	1401565	100.000

Figure S32. HPLC chromatogram of **5a**.

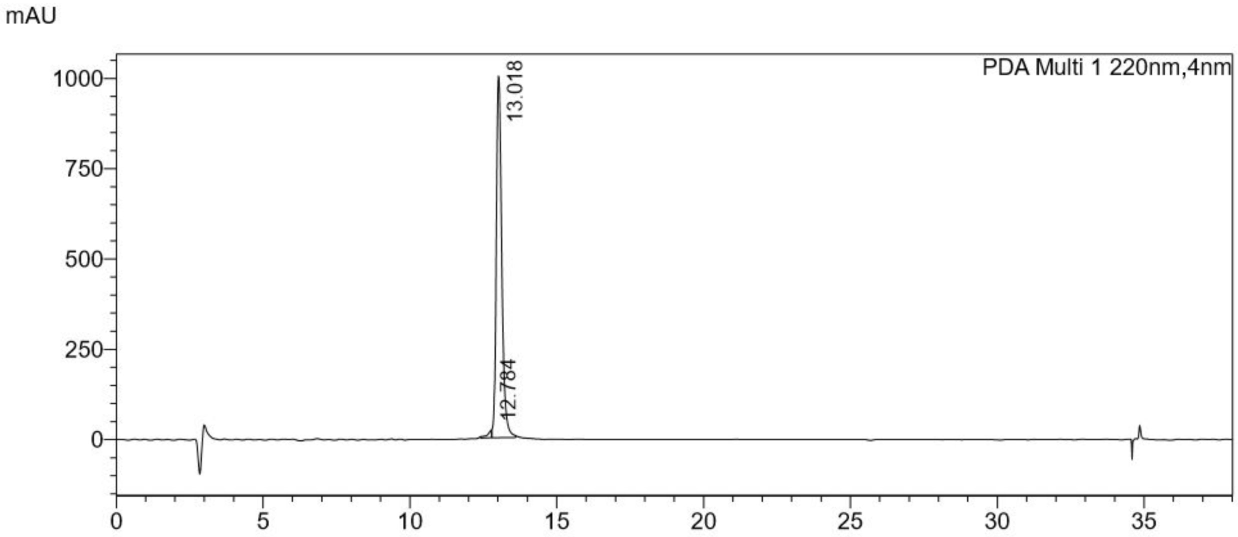

Peak Table

PDA Ch1 220nm

Peak#	Ret. Time	Area	Height	Area%
1	12.784	218978	21403	1.642
2	13.018	13118390	1000241	98.358
Total		13337367	1021644	100.000

Figure S33. HPLC chromatogram of **5b**.

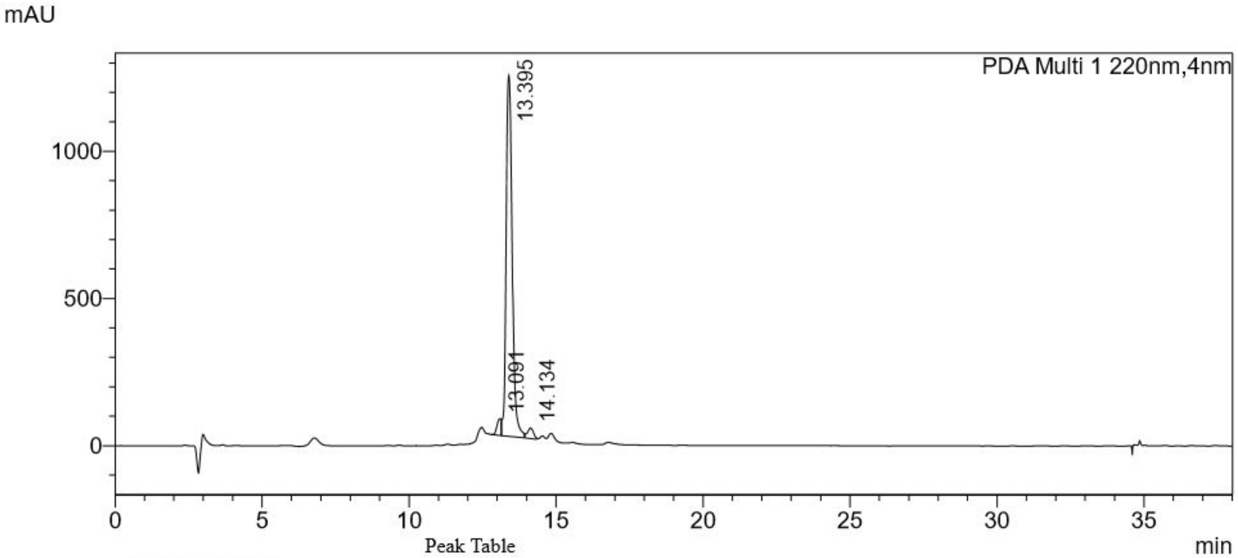

Peak Table

PDA Ch1 220nm

Peak#	Ret. Time	Area	Height	Area%
1	13.091	575443	56339	3.092
2	13.395	17526543	1225696	94.183
3	14.134	507056	35699	2.725
Total		18609042	1317734	100.000

Figure S34. HPLC chromatogram of **5c**.

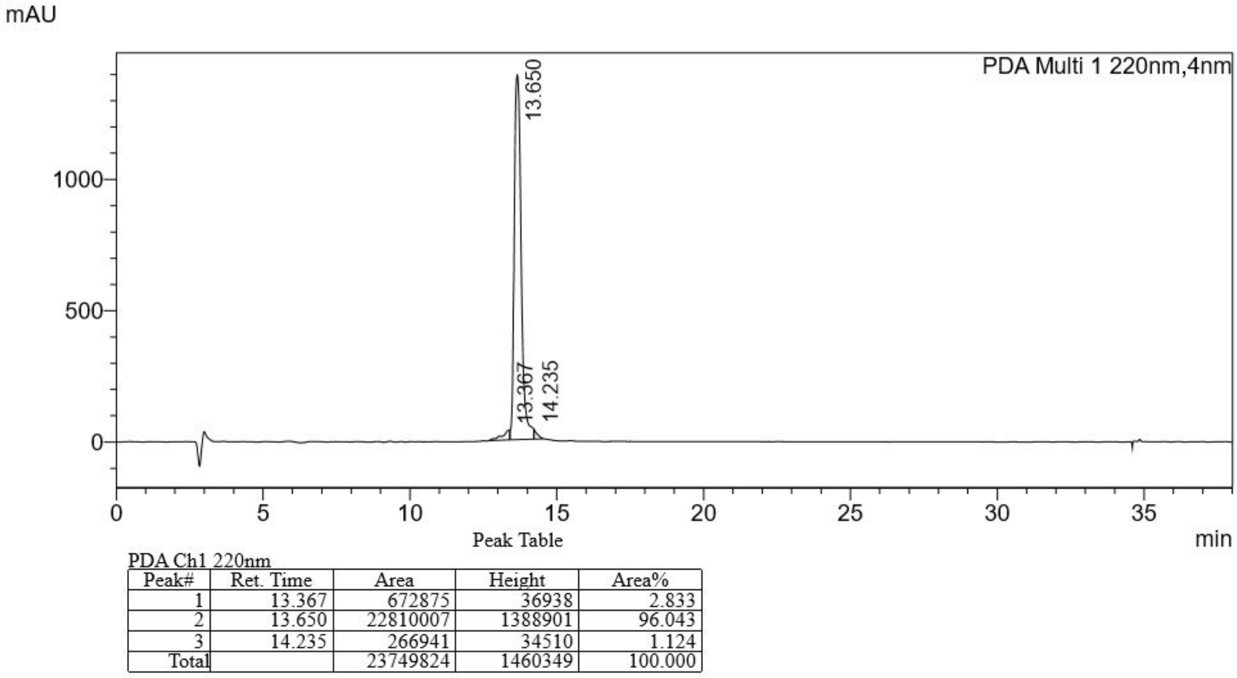

Peak Table

PDA Ch1 220nm

Peak#	Ret. Time	Area	Height	Area%
1	13.367	672875	36938	2.833
2	13.650	22810007	1388901	96.043
3	14.235	266941	34510	1.124
Total		23749824	1460349	100.000

Figure S35. HPLC chromatogram of **5d**.

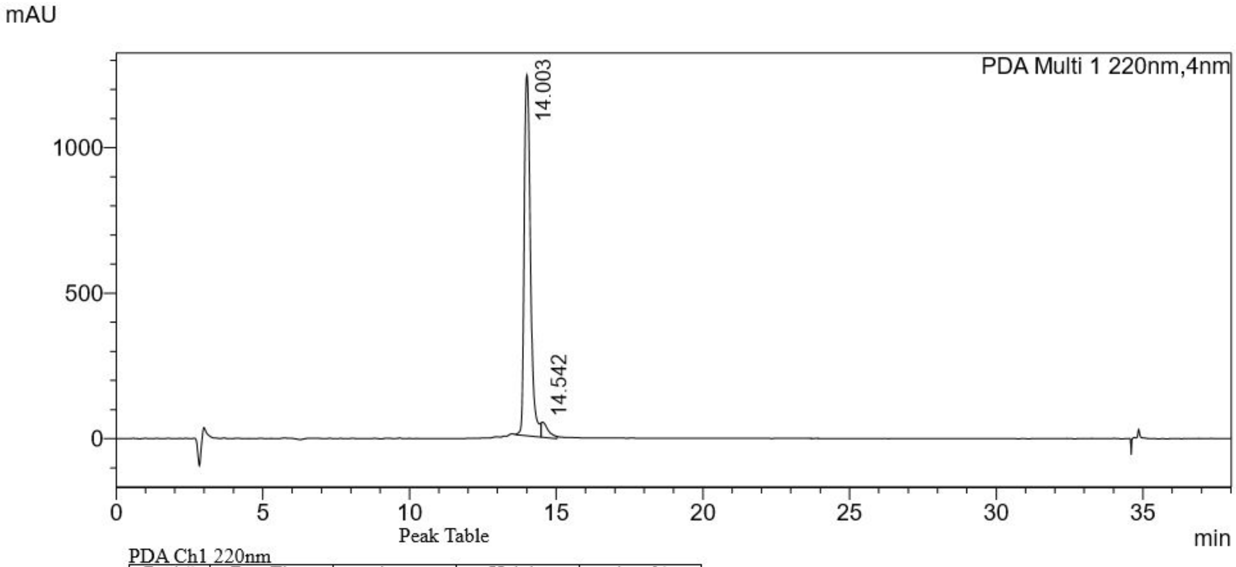

Peak Table

PDA Ch1 220nm

Peak#	Ret. Time	Area	Height	Area%
1	14.003	18631124	1239836	95.578
2	14.542	861921	51720	4.422
Total		19493045	1291556	100.000

Figure S36. HPLC chromatogram of **5e**.

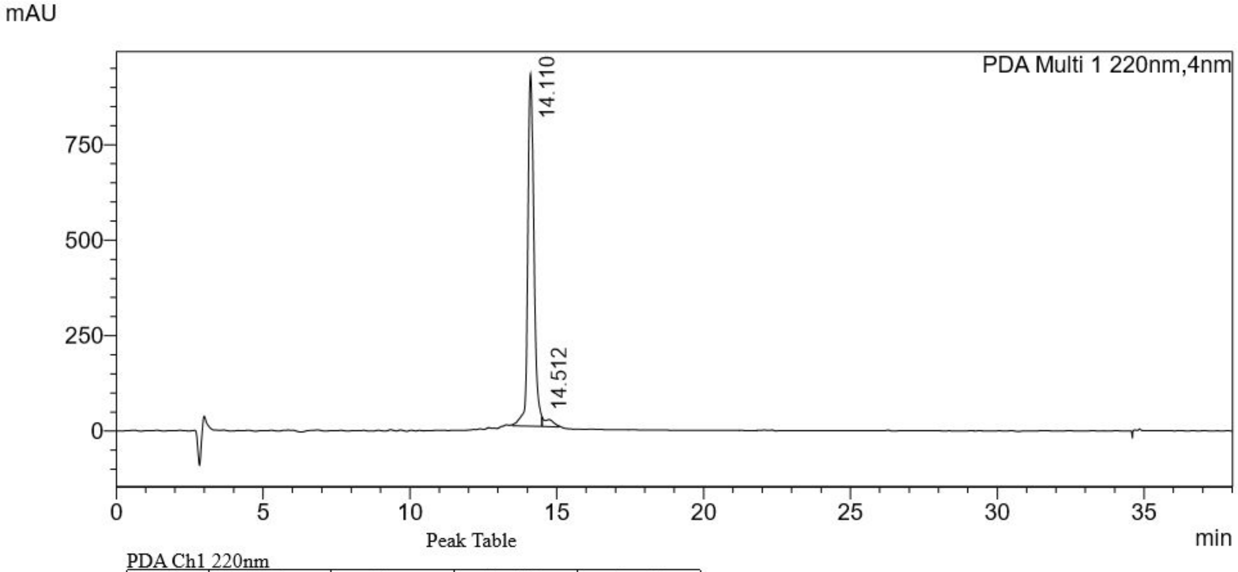

Peak Table

PDA Ch1 220nm

Peak#	Ret. Time	Area	Height	Area%
1	14.110	13263202	923083	96.667
2	14.512	457249	23299	3.333
Total		13720451	946382	100.000

Figure S37. HPLC chromatogram of **5f**.

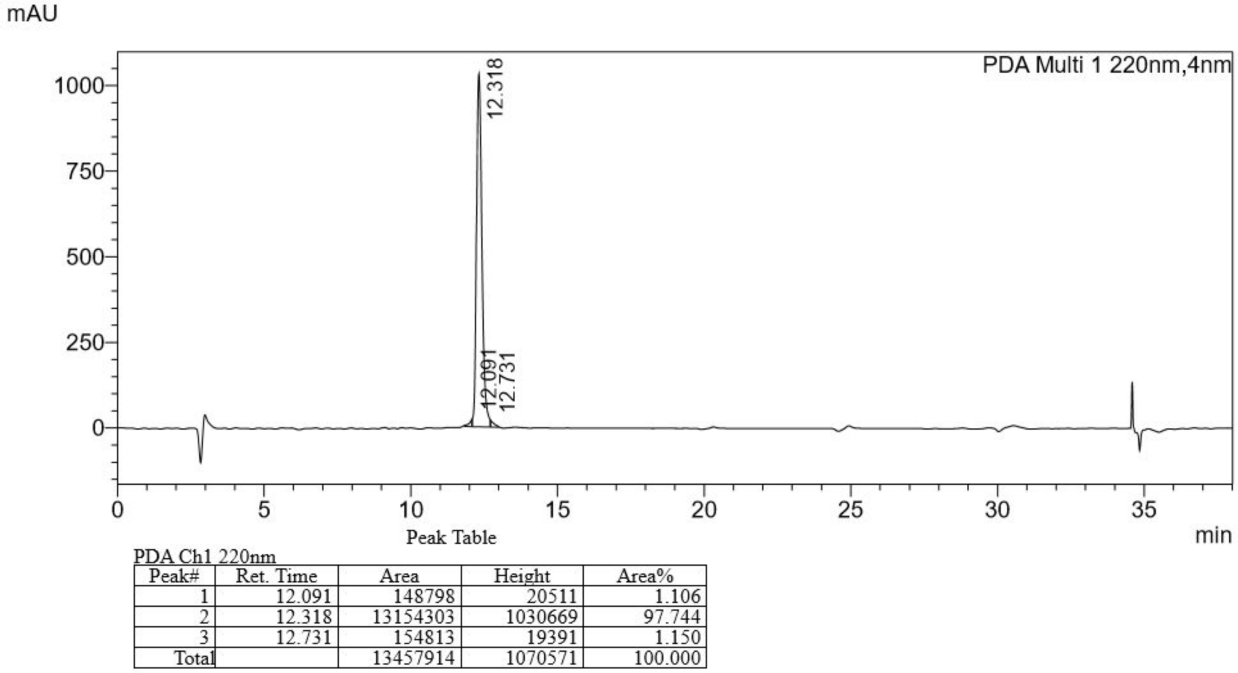

Peak Table

PDA Ch1 220nm

Peak#	Ret. Time	Area	Height	Area%
1	12.091	148798	20511	1.106
2	12.318	13154303	1030669	97.744
3	12.731	154813	19391	1.150
Total		13457914	1070571	100.000

Figure S38. HPLC chromatogram of **6a**.

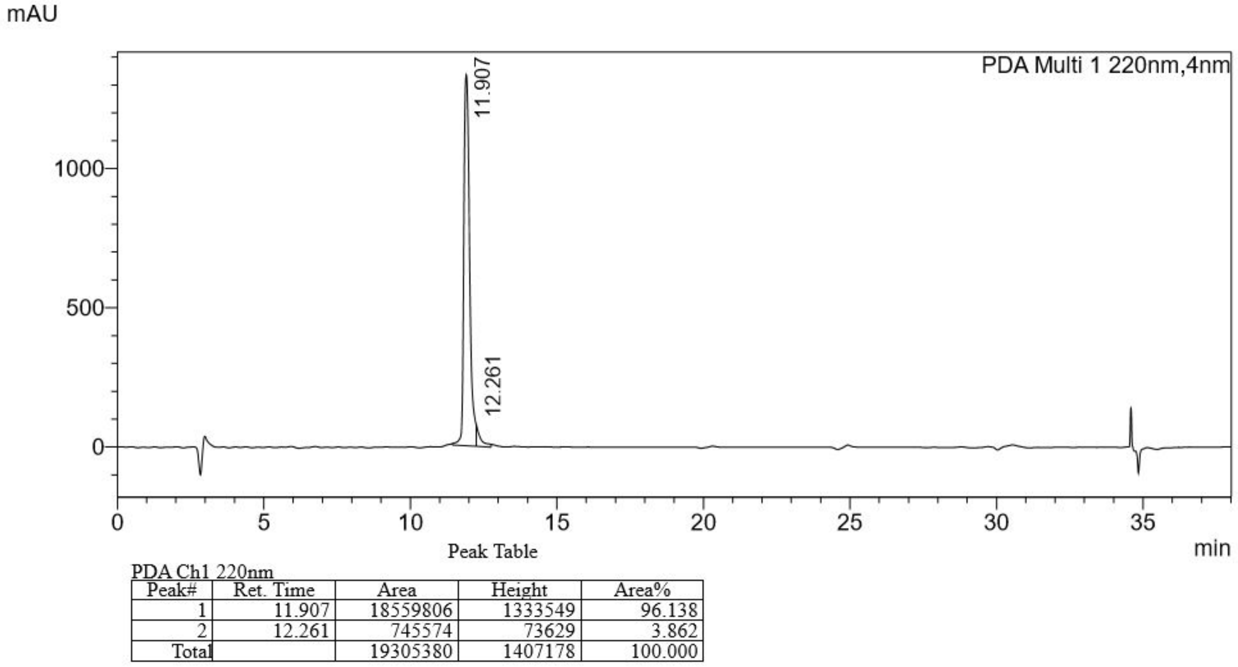

Peak Table

PDA Ch1 220nm

Peak#	Ret. Time	Area	Height	Area%
1	11.907	18559806	1333549	96.138
2	12.261	745574	73629	3.862
Total		19305380	1407178	100.000

Figure S39. HPLC chromatogram of **6b**.

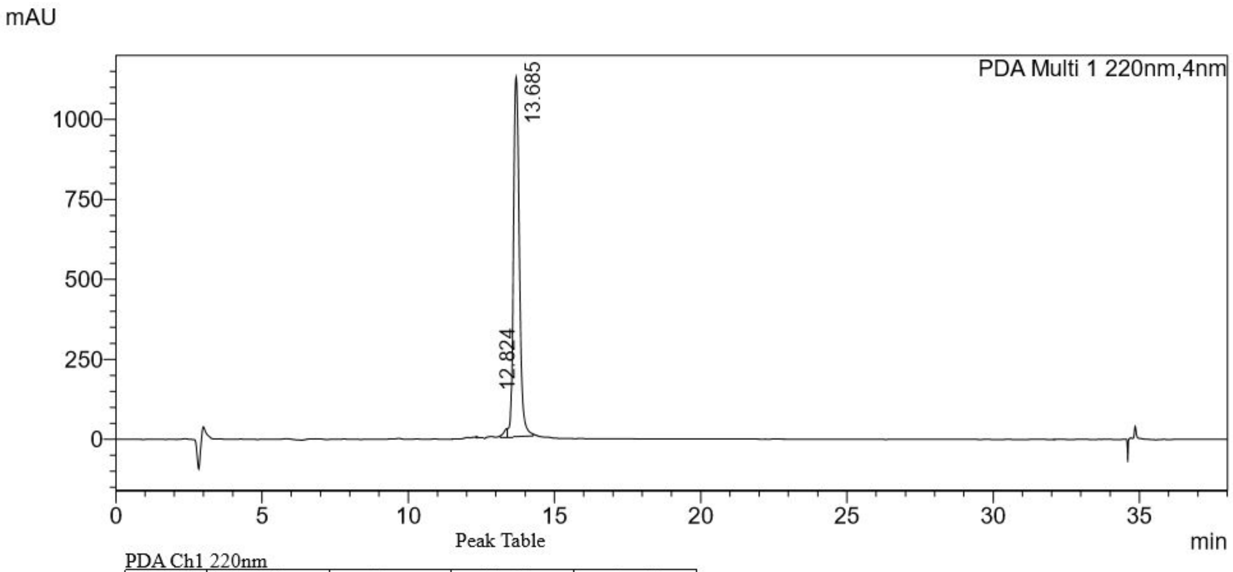

Peak Table

PDA Ch1 220nm

Peak#	Ret. Time	Area	Height	Area%
1	12.824	2193350	128188	11.482
2	13.685	16909435	1122500	88.518
Total		19102785	1250688	100.000

Figure S40. HPLC chromatogram of **6c**.

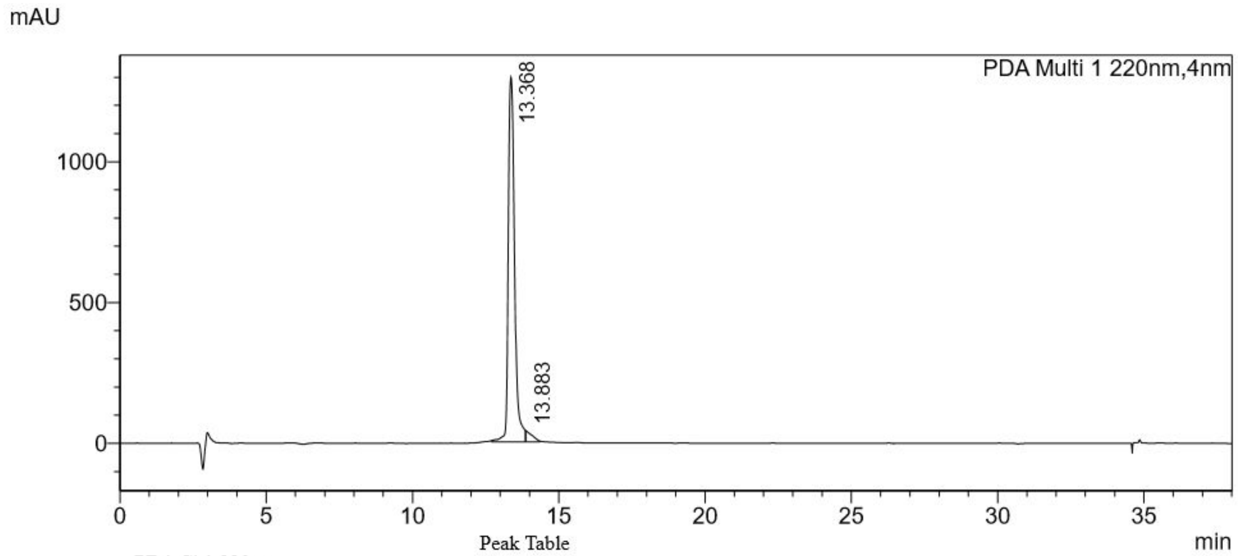

Peak Table

PDA Ch1 220nm

Peak#	Ret. Time	Area	Height	Area%
1	13.368	19471263	1295013	97.006
2	13.883	601008	39379	2.994
Total		20072271	1334393	100.000

Figure S41. HPLC chromatogram of **6d**.

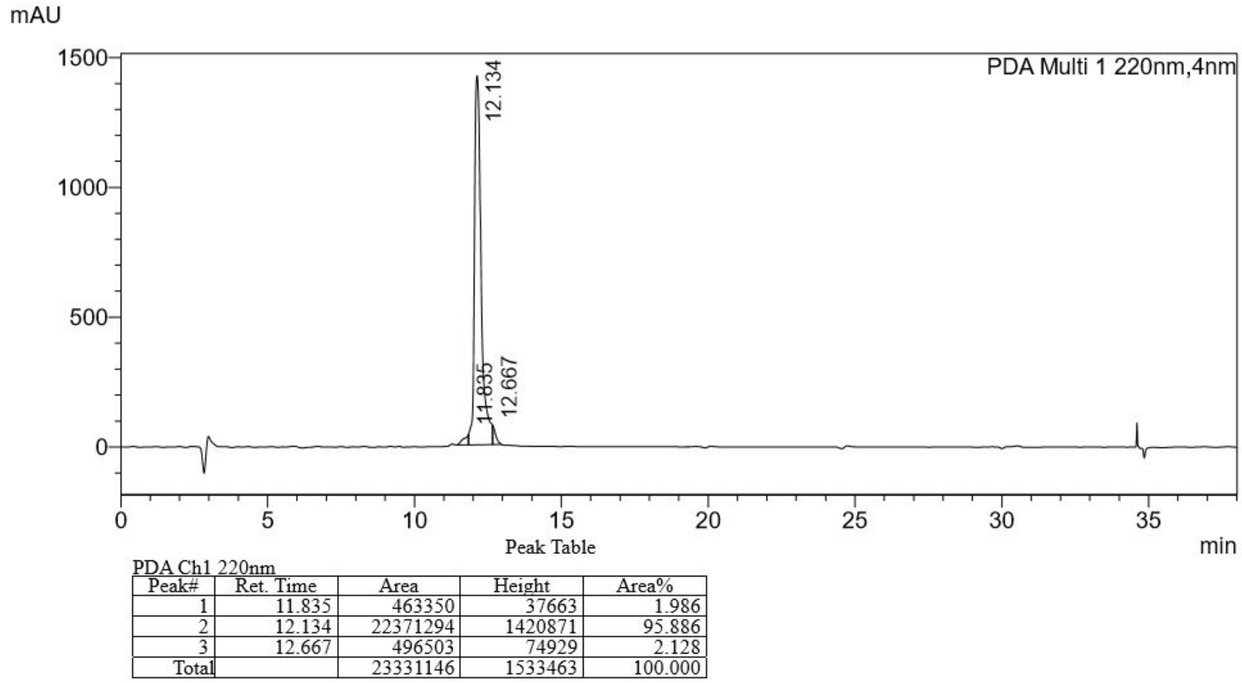

Peak Table

PDA Ch1 220nm

Peak#	Ret. Time	Area	Height	Area%
1	11.835	463350	37663	1.986
2	12.134	22371294	1420871	95.886
3	12.667	496503	74929	2.128
Total		23331146	1533463	100.000

Figure S42. HPLC chromatogram of **6e**.

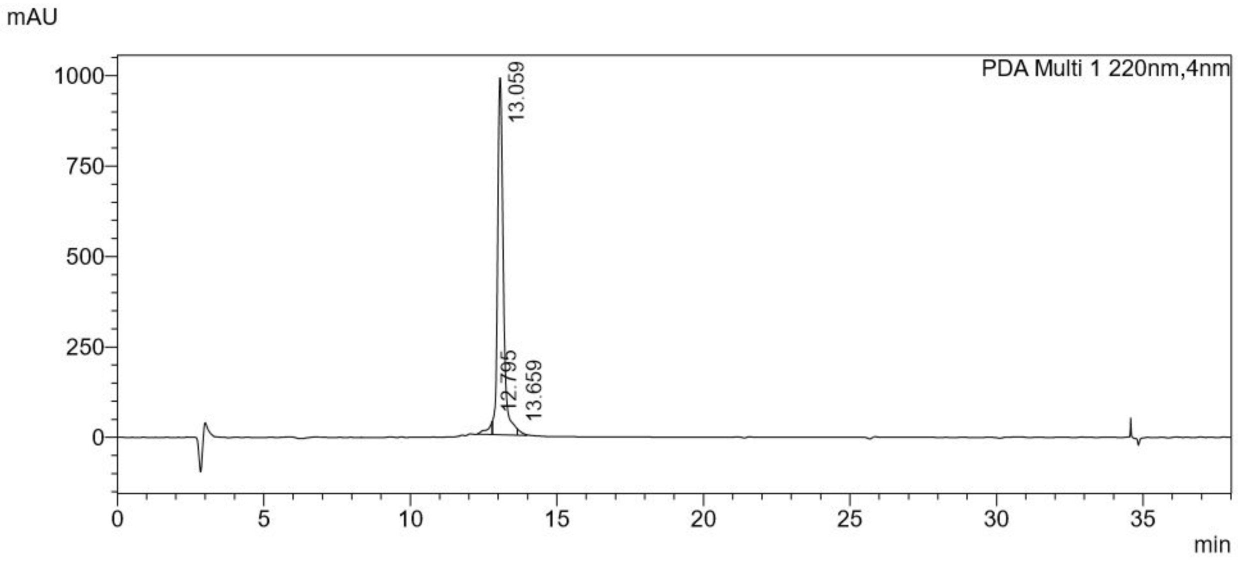

Peak Table

PDA Ch1 220nm

Peak#	Ret. Time	Area	Height	Area%
1	12.795	413971	34883	2.936
2	13.059	13529063	988201	95.941
3	13.659	158434	16945	1.124
Total		14101468	1040028	100.000

Figure S43. HPLC chromatogram of **7a**.

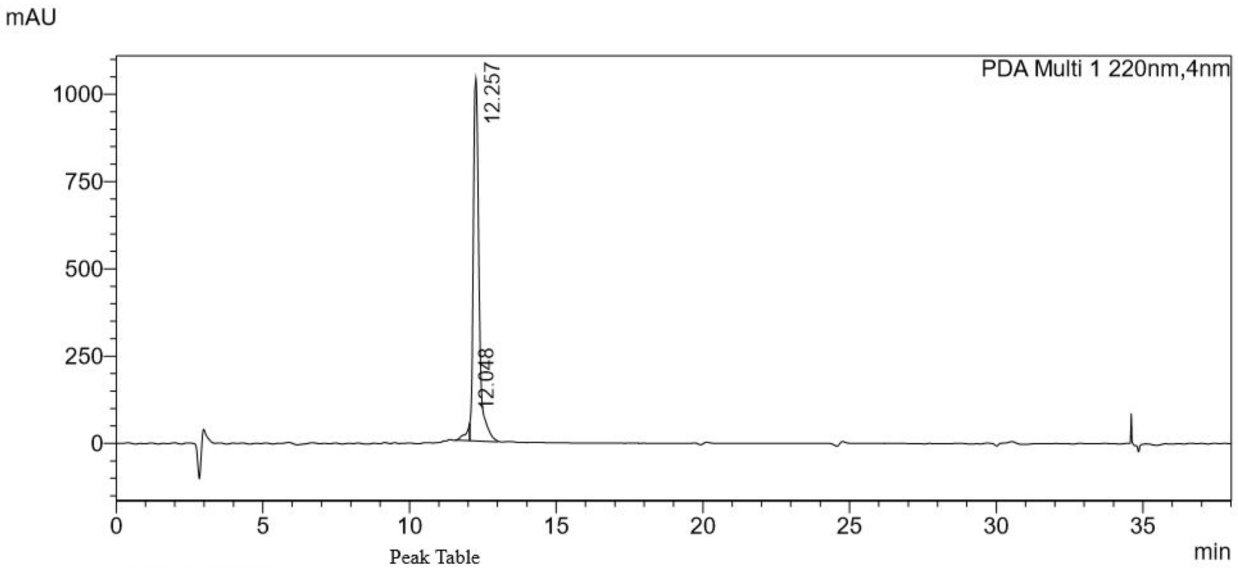

Peak Table

PDA Ch1 220nm

Peak#	Ret. Time	Area	Height	Area%
1	12.048	459309	45838	3.248
2	12.257	13683518	1039478	96.752
Total		14142827	1085315	100.000

Figure S44. HPLC chromatogram of **7b**.

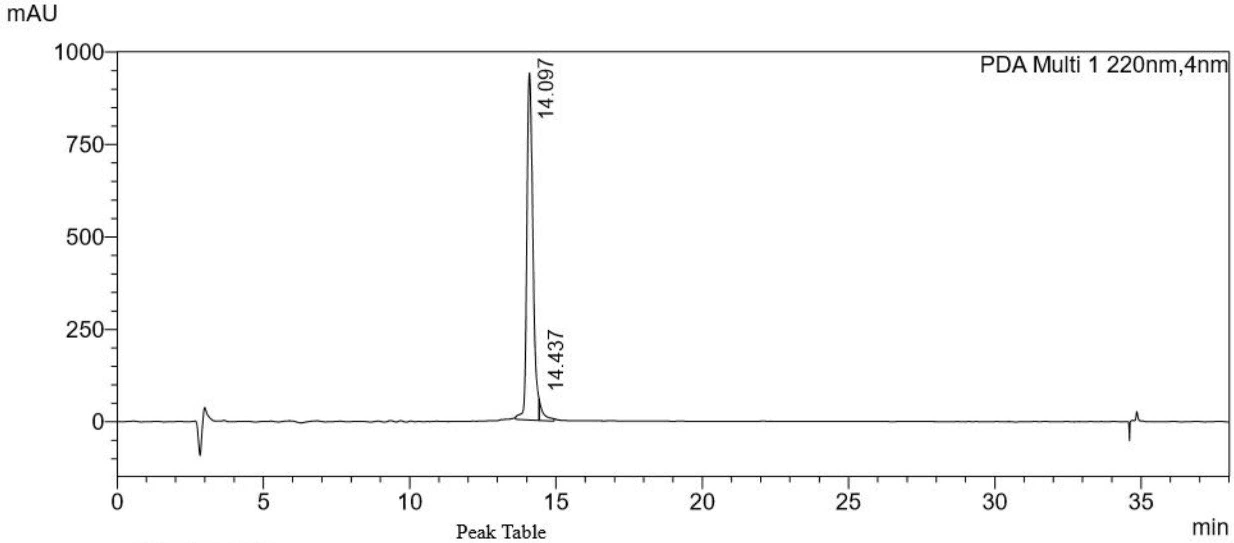

Peak Table

PDA Ch1 220nm

Peak#	Ret. Time	Area	Height	Area%
1	14.097	13301668	938329	96.216
2	14.437	523115	53578	3.784
Total		13824783	991907	100.000

Figure S45. HPLC chromatogram of **7c**.

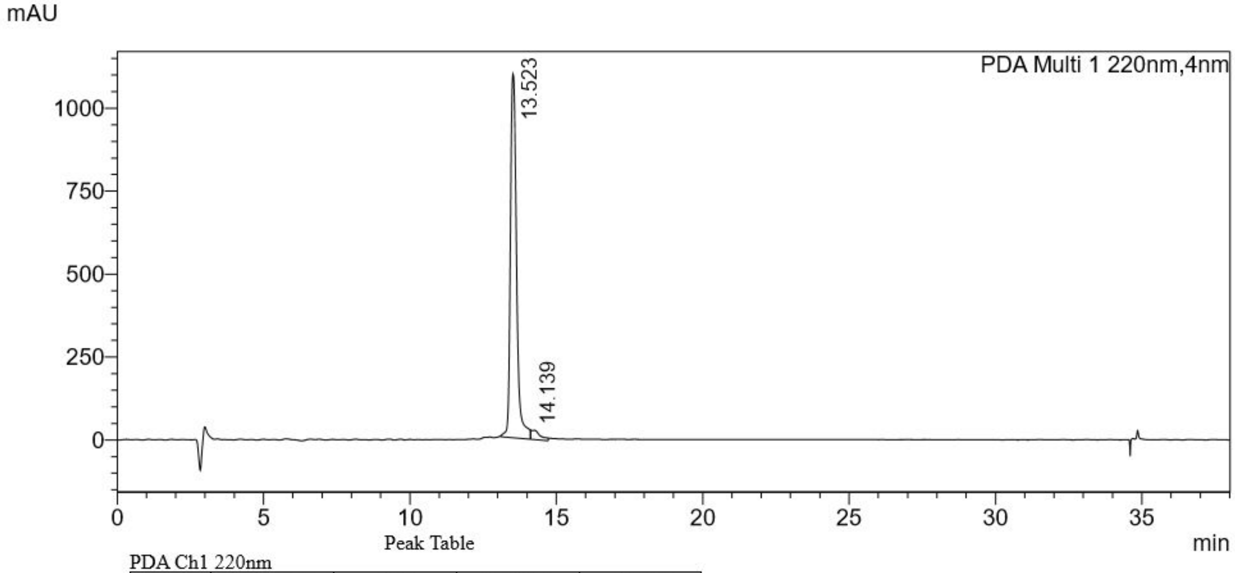

Peak Table

PDA Ch1 220nm

Peak#	Ret. Time	Area	Height	Area%
1	13.523	15591998	1097215	96.200
2	14.139	615980	27393	3.800
Total		16207978	1124608	100.000

Figure S46. HPLC chromatogram of **7d**.

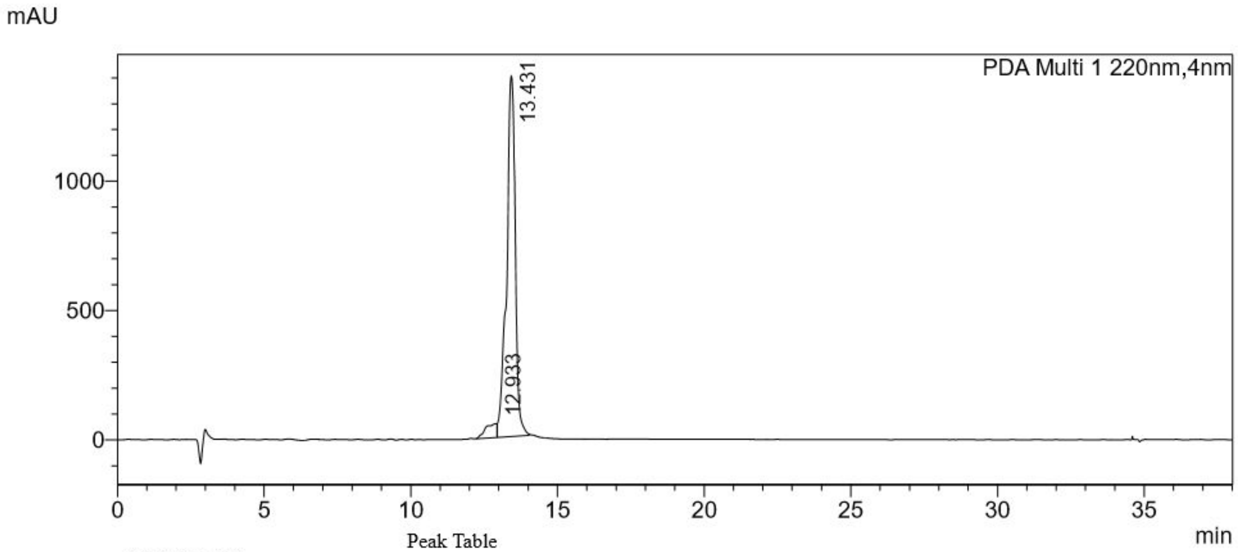

Peak Table

PDA Ch1 220nm

Peak#	Ret. Time	Area	Height	Area%
1	12.933	1458986	52584	4.884
2	13.431	28415226	1392883	95.116
Total		29874212	1445467	100.000

Figure S47. HPLC chromatogram of **8a**.

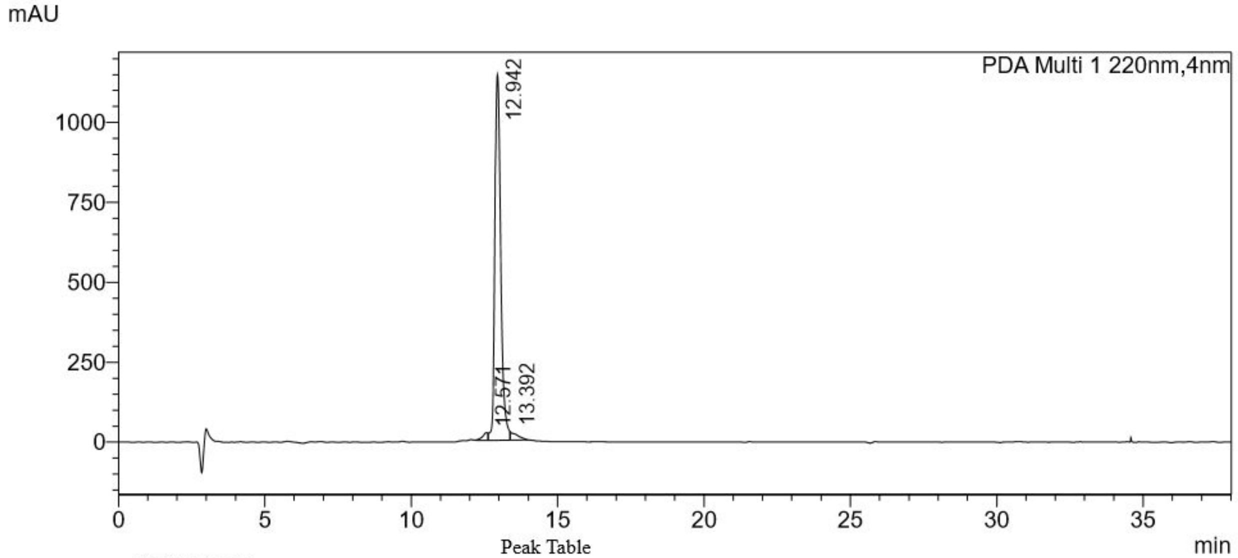

Peak Table

PDA Ch1 220nm

Peak#	Ret. Time	Area	Height	Area%
1	12.571	319084	23852	1.920
2	12.942	15874288	1144141	95.495
3	13.392	429726	25534	2.585
Total		16623097	1193527	100.000

Figure S48. HPLC chromatogram of **8b**.

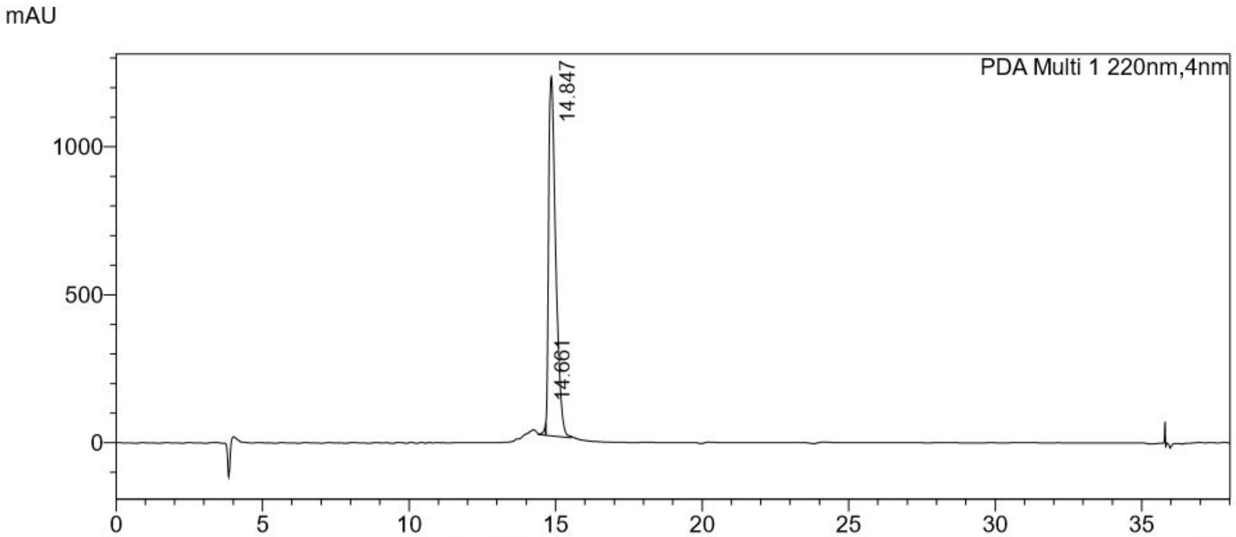

Peak Table

PDA Ch1 220nm

Peak#	Ret. Time	Area	Height	Area%
1	14.661	174562	35559	0.863
2	14.847	20045395	1214209	99.137
Total		20219957	1249768	100.000

Figure S49. HPLC chromatogram of **8c**.

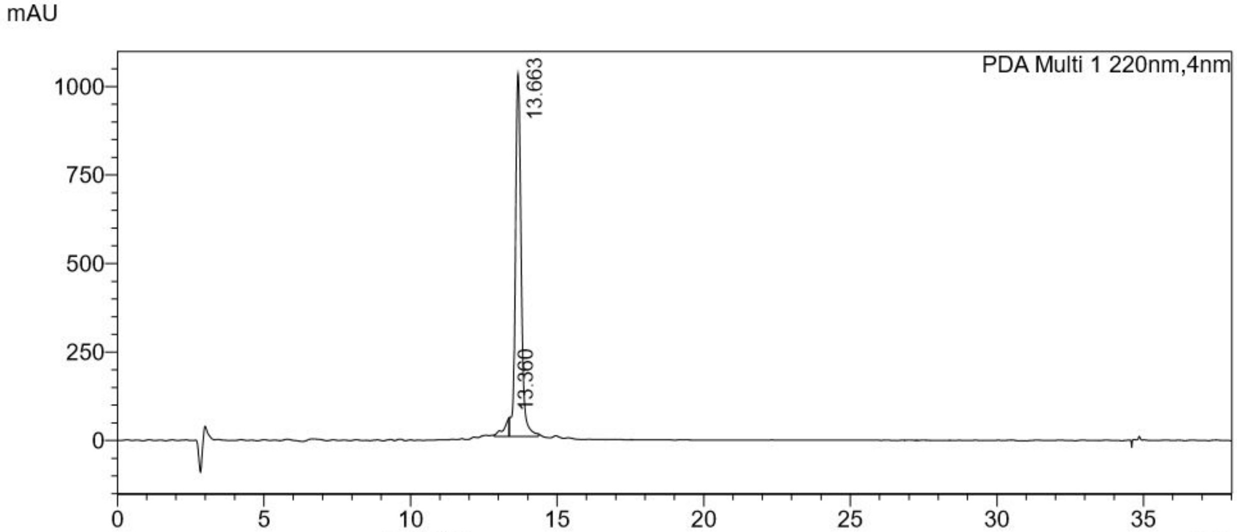

Peak Table

PDA Ch1 220nm

Peak#	Ret. Time	Area	Height	Area%
1	13.360	670269	52155	4.518
2	13.663	14166888	1024169	95.482
Total		14837157	1076323	100.000

Figure S50. HPLC chromatogram of **8d**.

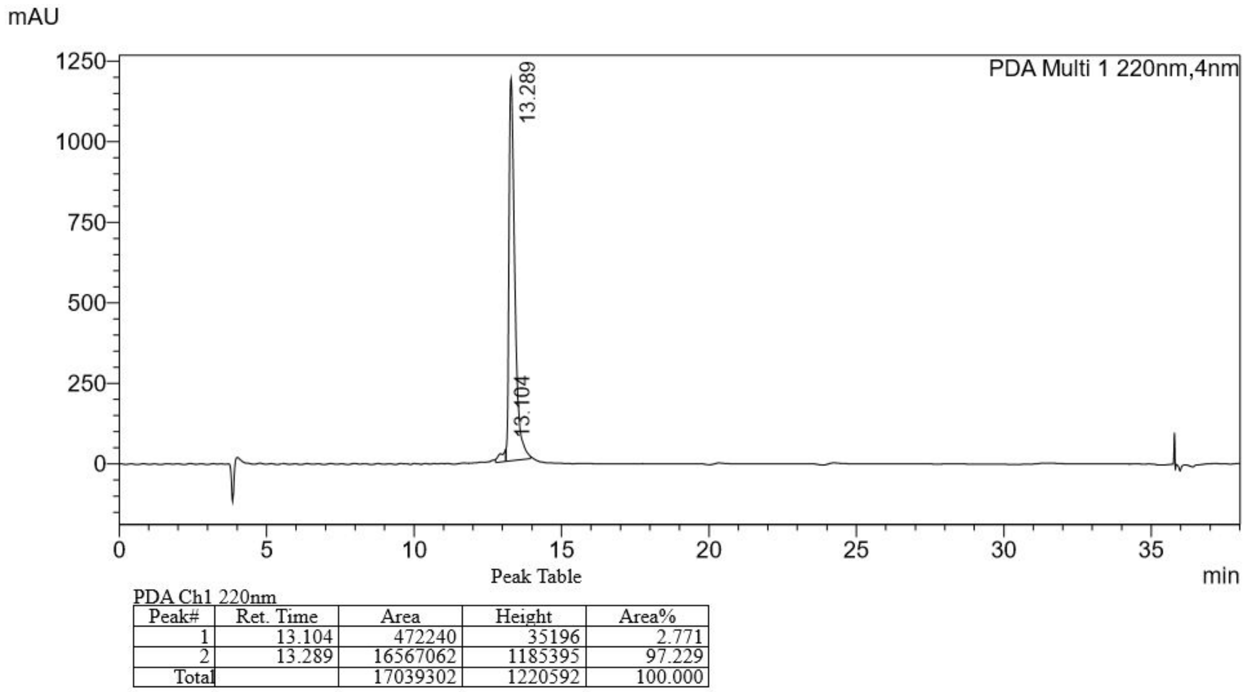

Peak Table

PDA Ch1 220nm

Peak#	Ret. Time	Area	Height	Area%
1	13.104	472240	35196	2.771
2	13.289	16567062	1185395	97.229
Total		17039302	1220592	100.000

Figure S51. HPLC chromatogram of **8e**.

8. Comparative Hydrophobicity analysis

The purity of all synthesized peptides was determined using reverse phase analytical HPLC (Shimadzu; LC-20ADXR). Below mentioned are the conditions used to conduct the purity analysis.

Method 1

Column: Phenomenex (Luna), 4 um, C18, 150 × 4.6 mm

Flow rate: 0.5 ml/min

Mobile Phase: Buffer A-Water containing 0.1% TFA,
Buffer B-Acetonitrile containing 0.1% TFA

Gradient

Time (min)	Buffer B (%)
0.01	2
2	2
2.01	2
65.01	65
65.02	100
72.00	100
72.01	2
80	2

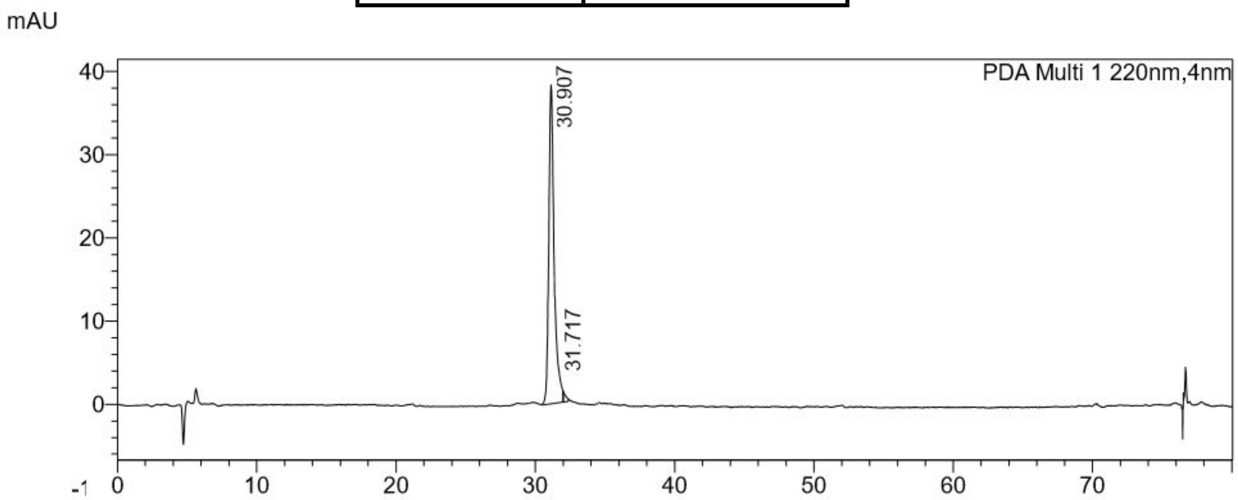

Peak Table

PDA Ch1 220nm

Peak#	Ret. Time	Area	Height	Area%
1	30.907	1697199	38004	96.745
2	31.717	57095	7705	3.255
Total		1754294	45708	100.000

Figure S52. HPLC chromatogram of **6a**.

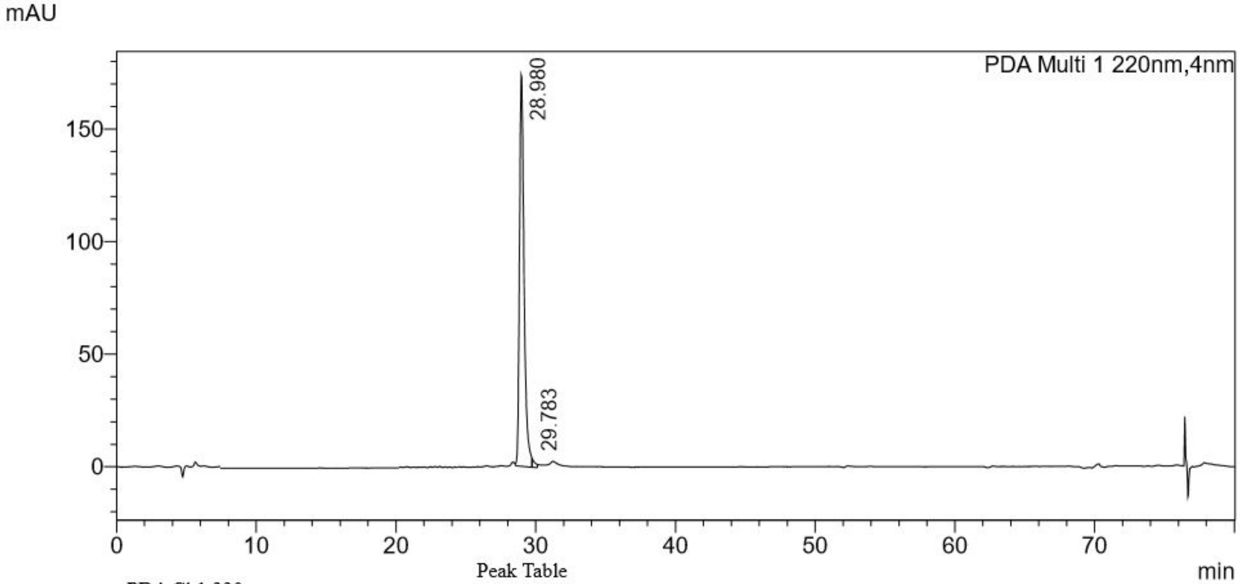

Peak Table

PDA Ch1 220nm

Peak#	Ret. Time	Area	Height	Area%
1	28.980	3962719	173701	98.732
2	29.783	50901	3512	1.268
Total		4013620	177213	100.000

Figure S53. HPLC chromatogram of **6b**.

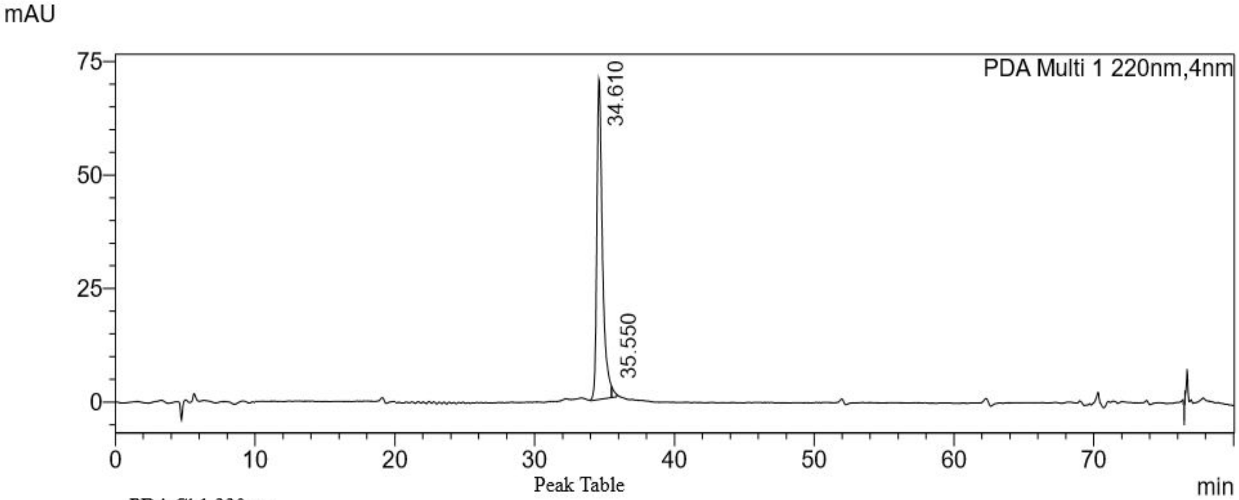

Peak Table

PDA Ch1 220nm

Peak#	Ret. Time	Area	Height	Area%
1	34.610	1981006	70551	98.569
2	35.550	28759	2209	1.431
Total		2009766	72760	100.000

Figure S54. HPLC chromatogram of **6c**.

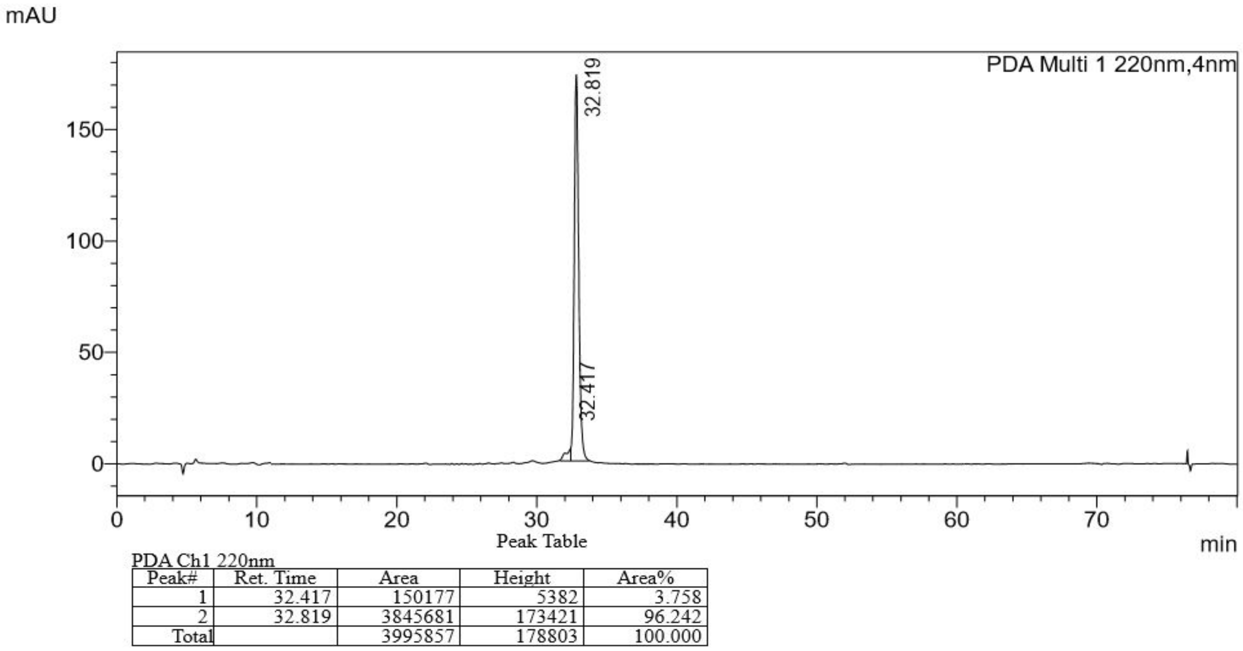

PDA Ch1 220nm

Peak#	Ret. Time	Area	Height	Area%
1	32.417	150177	5382	3.758
2	32.819	3845681	173421	96.242
Total		3995857	178803	100.000

Figure S55. HPLC chromatogram of **6d**.

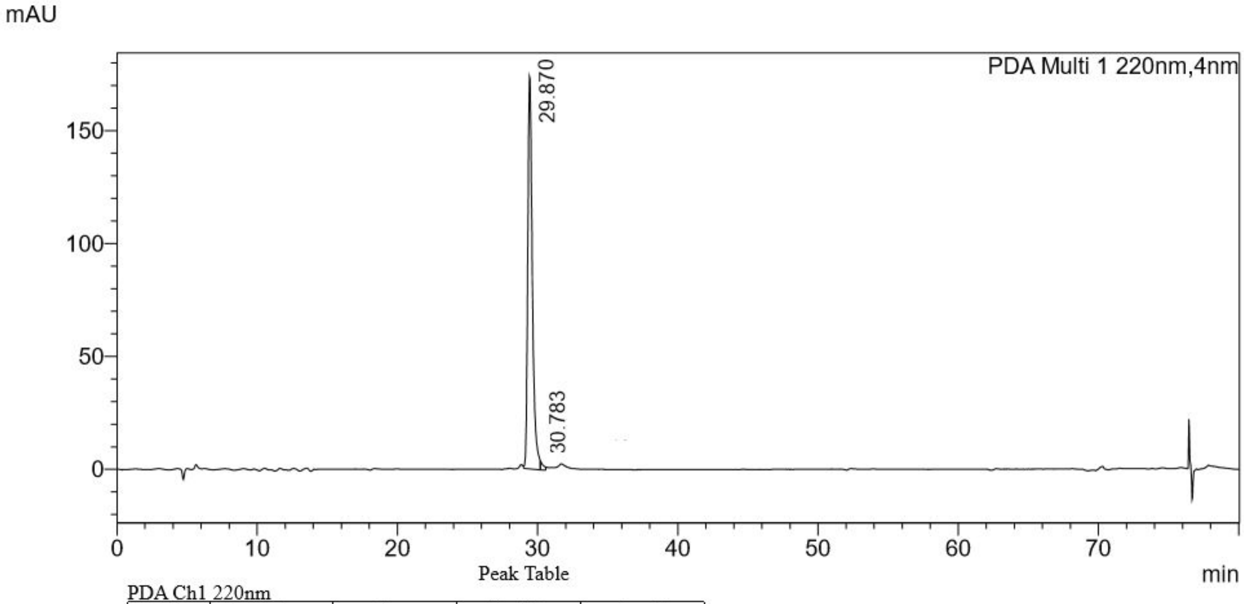

PDA Ch1 220nm

Peak#	Ret. Time	Area	Height	Area%
1	29.870	3962719	173701	98.732
2	30.783	50901	3512	1.268
Total		4013620	177213	100.000

Figure S56. HPLC chromatogram of **6e**.

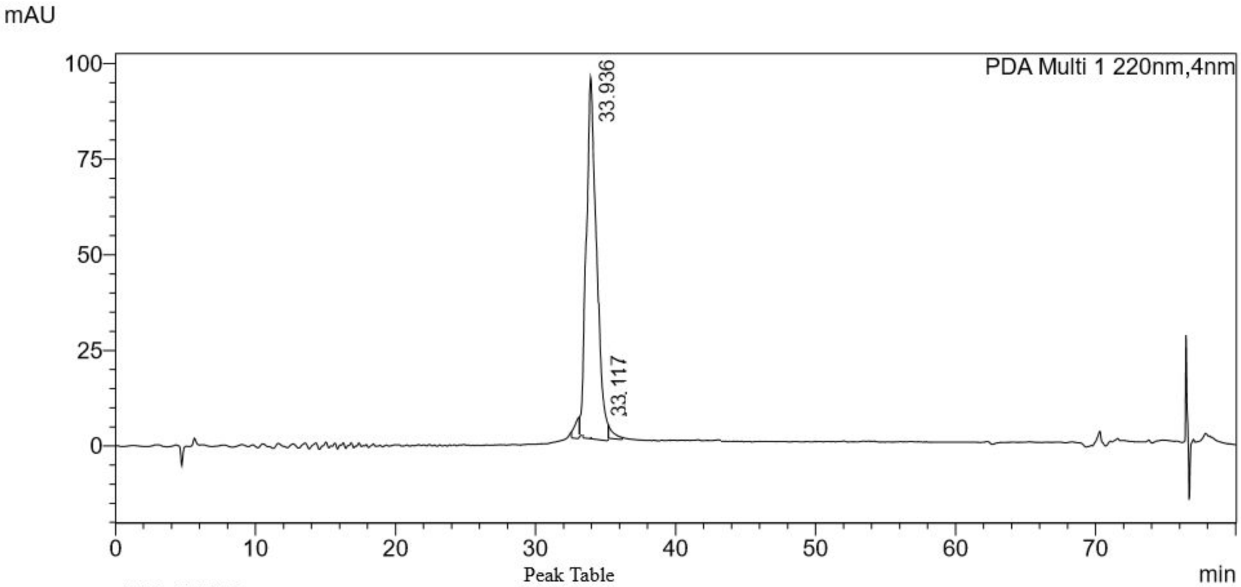

Peak Table

PDA Ch1 220nm

Peak#	Ret. Time	Area	Height	Area%
1	33.117	130618	5580	2.688
2	33.936	4729458	94895	97.312
Total		4860077	100475	100.000

Figure S57. HPLC chromatogram of **7a**.

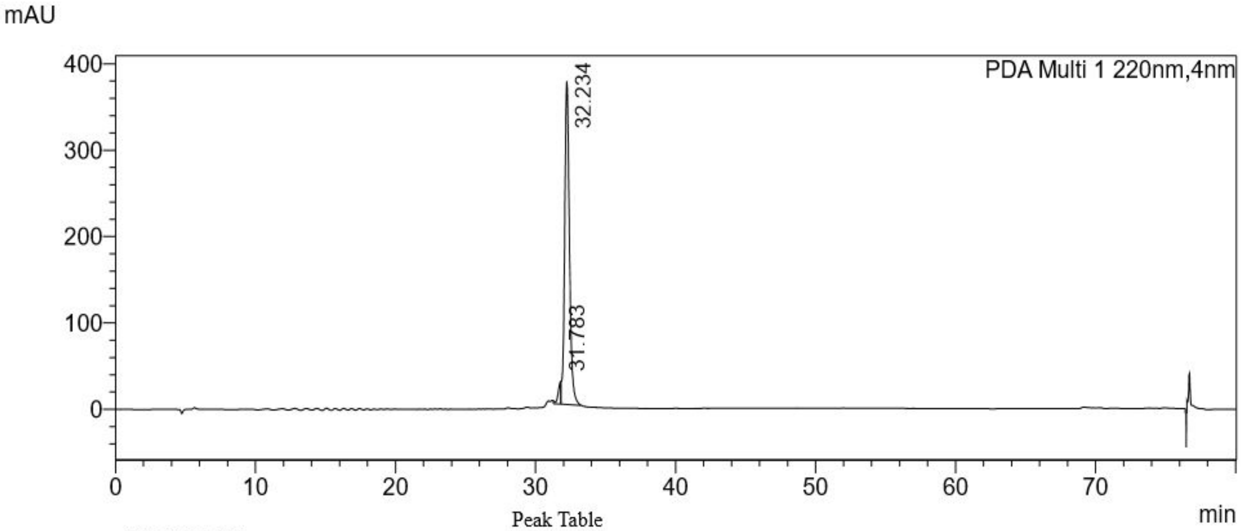

Peak Table

PDA Ch1 220nm

Peak#	Ret. Time	Area	Height	Area%
1	31.783	352157	25695	3.785
2	32.234	8952154	374464	96.215
Total		9304311	400159	100.000

Figure S58. HPLC chromatogram of **7b**.

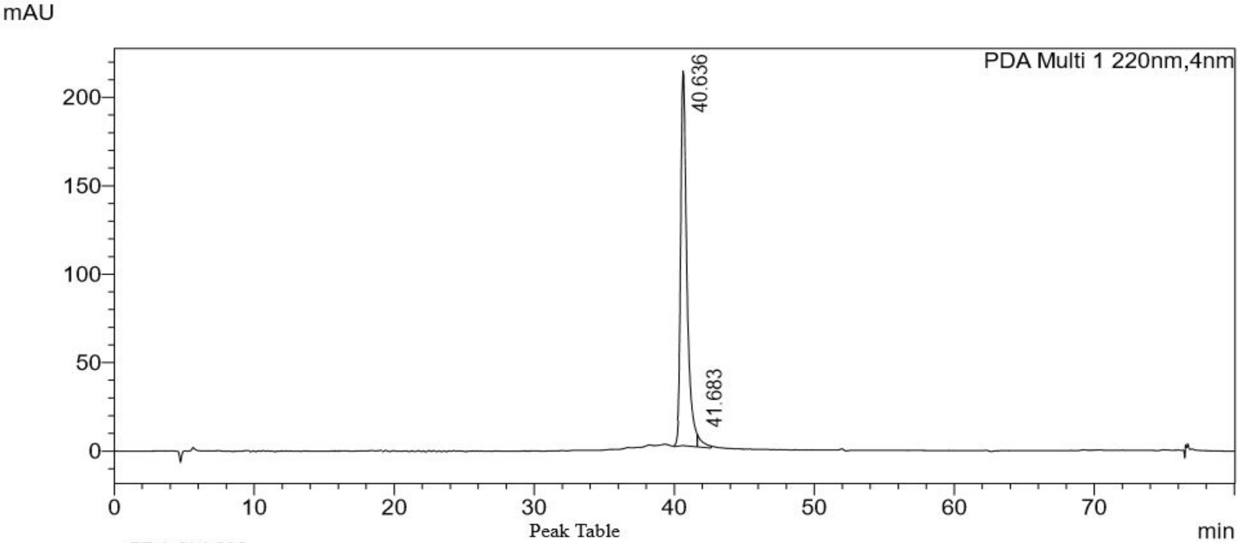

Peak Table

PDA Ch1 220nm

Peak#	Ret. Time	Area	Height	Area%
1	40.636	6658599	212120	97.505
2	41.683	170353	6607	2.495
Total		6828952	218727	100.000

Figure S59. HPLC chromatogram of **7c**.

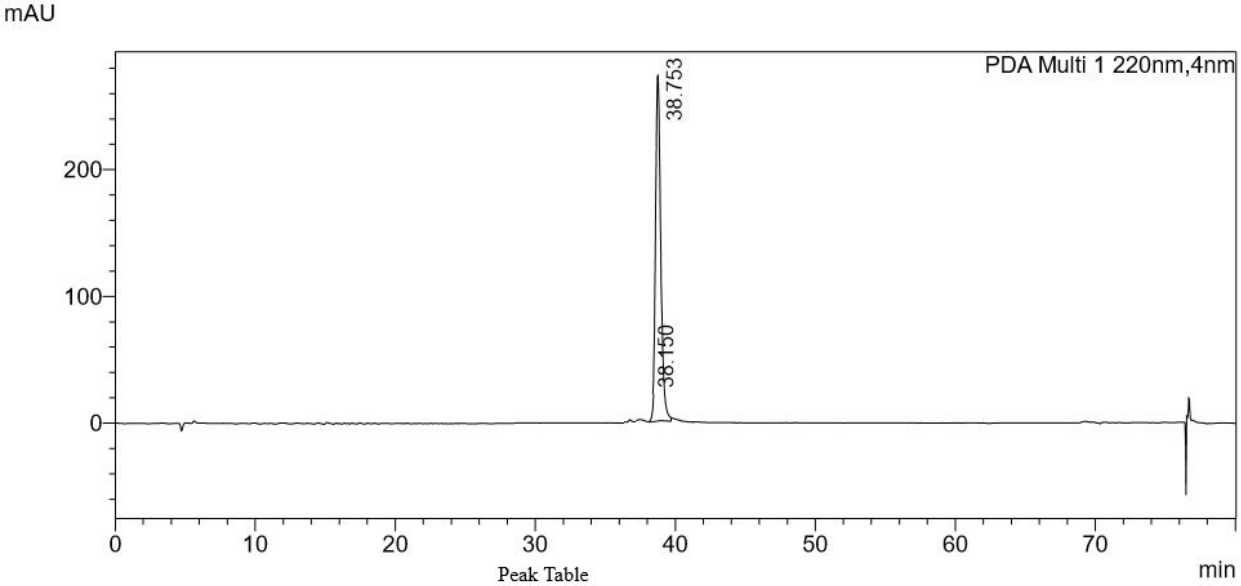

Peak Table

PDA Ch1 220nm

Peak#	Ret. Time	Area	Height	Area%
1	38.150	183909	16575	2.507
2	38.753	7152803	272148	97.493
Total		7336712	288723	100.000

Figure S60. HPLC chromatogram of **7d**.

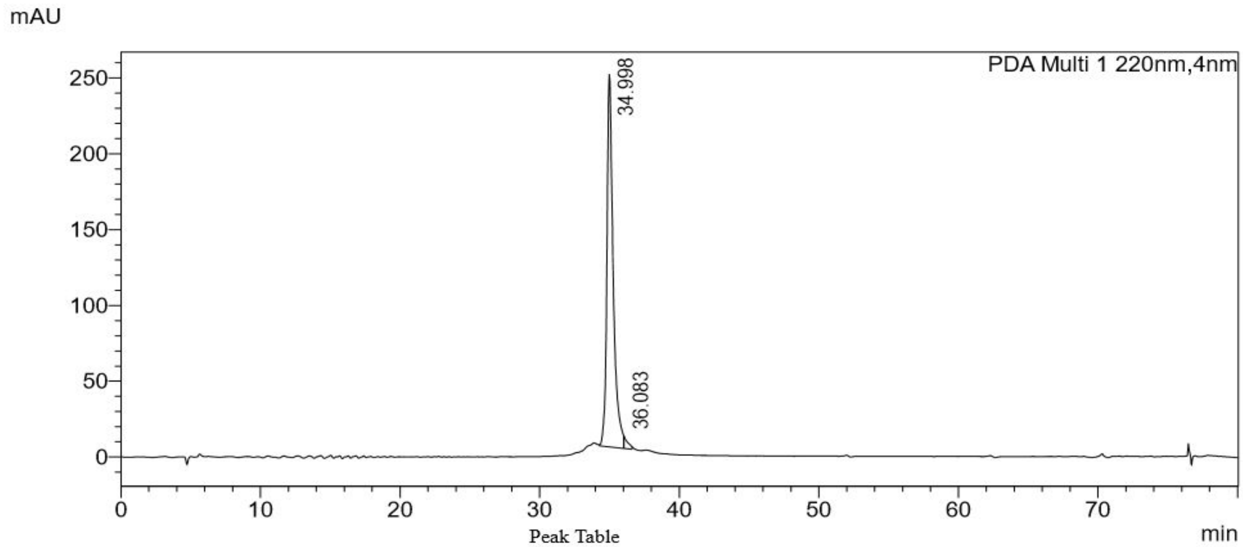

Peak Table

PDA Ch1 220nm

Peak#	Ret. Time	Area	Height	Area%
1	34.998	7585853	245805	98.149
2	36.083	143034	7373	1.851
Total		7728887	253177	100.000

Figure S61. HPLC chromatogram of **8a**.

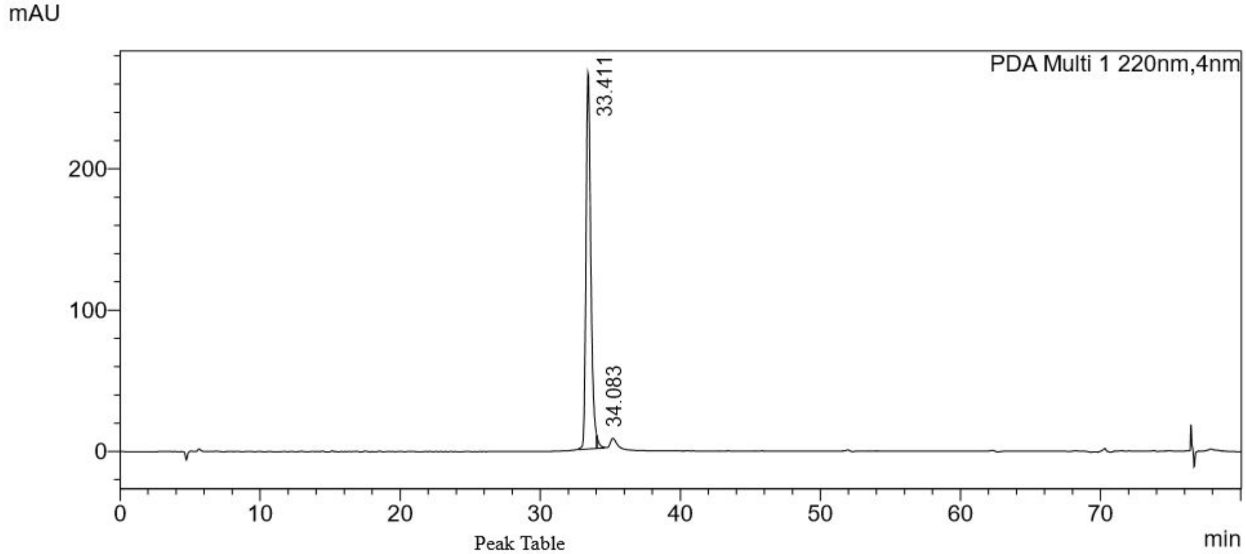

Peak Table

PDA Ch1 220nm

Peak#	Ret. Time	Area	Height	Area%
1	33.411	6351513	266161	98.679
2	34.083	85057	7611	1.321
Total		6436571	273772	100.000

Figure S62. HPLC chromatogram of **8b**.

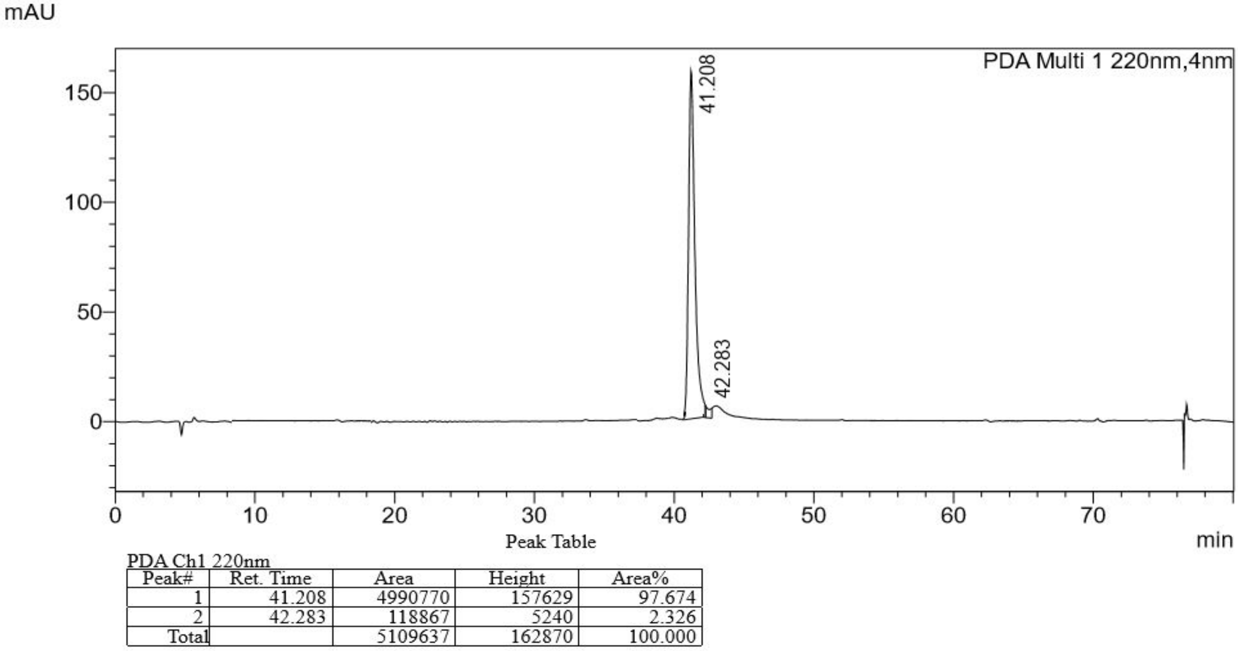

Peak Table

PDA Ch1 220nm

Peak#	Ret. Time	Area	Height	Area%
1	41.208	4990770	157629	97.674
2	42.283	118867	5240	2.326
Total		5109637	162870	100.000

Figure S63. HPLC chromatogram of **8c**.

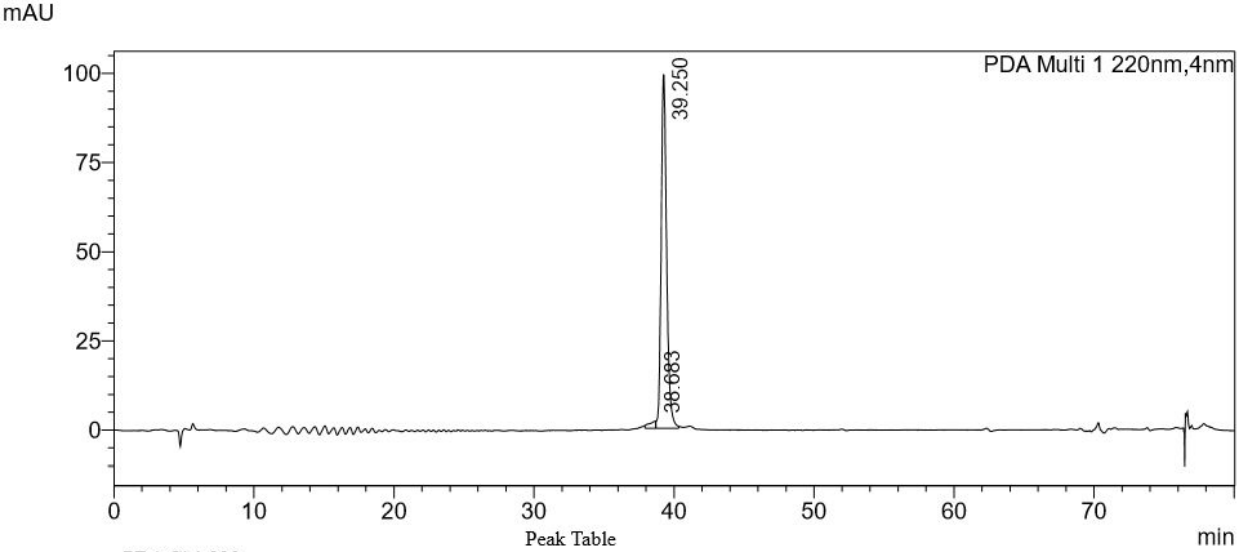

Peak Table

PDA Ch1 220nm

Peak#	Ret. Time	Area	Height	Area%
1	38.683	67785	2008	2.466
2	39.250	2681525	99077	97.534
Total		2749310	101084	100.000

Figure S64. HPLC chromatogram of **8d**.

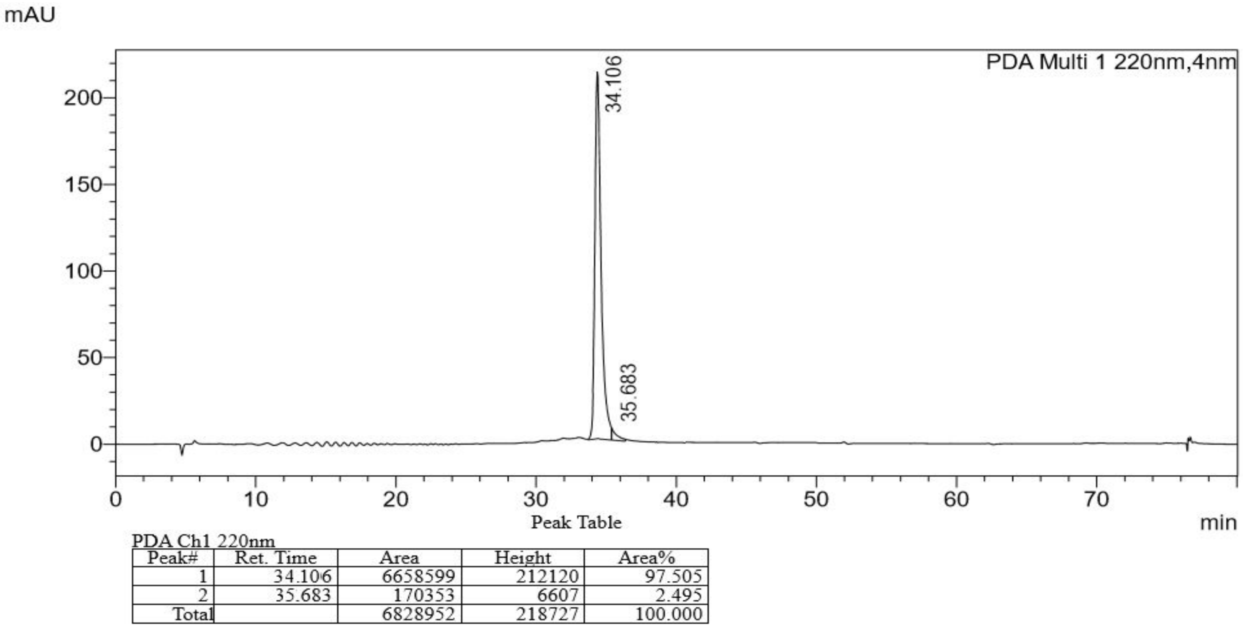

Peak Table

PDA Ch1 220nm

Peak#	Ret. Time	Area	Height	Area%
1	34.106	6658599	212120	97.505
2	35.683	170353	6607	2.495
Total		6828952	218727	100.000

Figure S65. HPLC chromatogram of **8e**.

9. High-resolution mass spectrometry (HRMS) data of all the synthesized peptides (1a-8e)

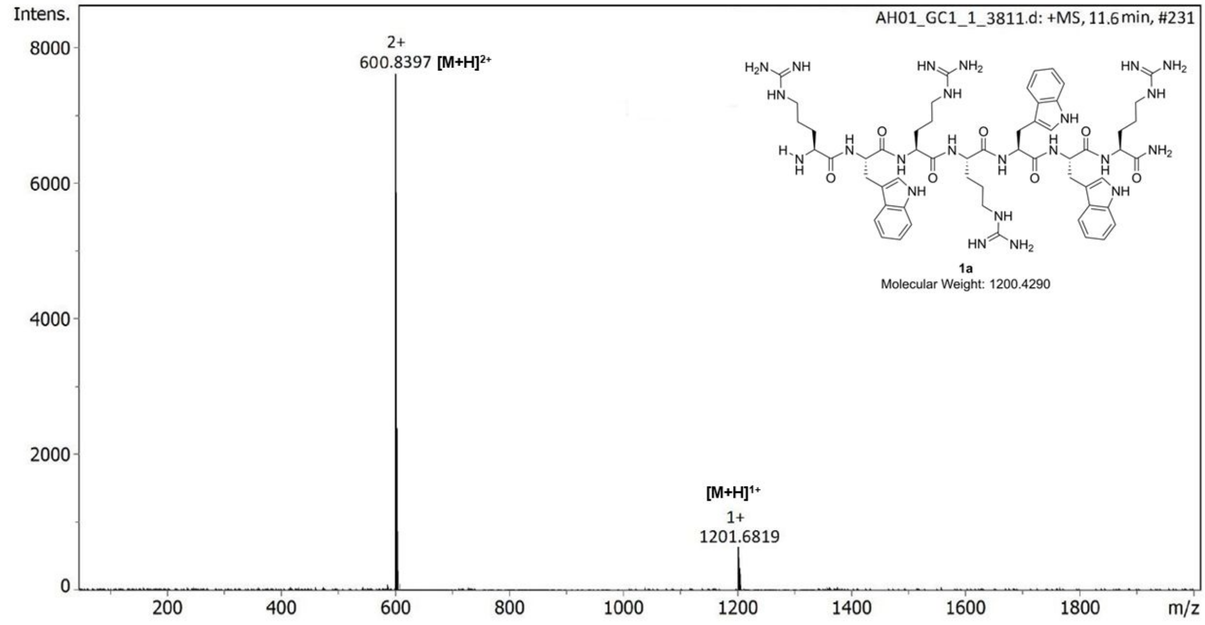

Figure S66. HRMS data of **1a**.

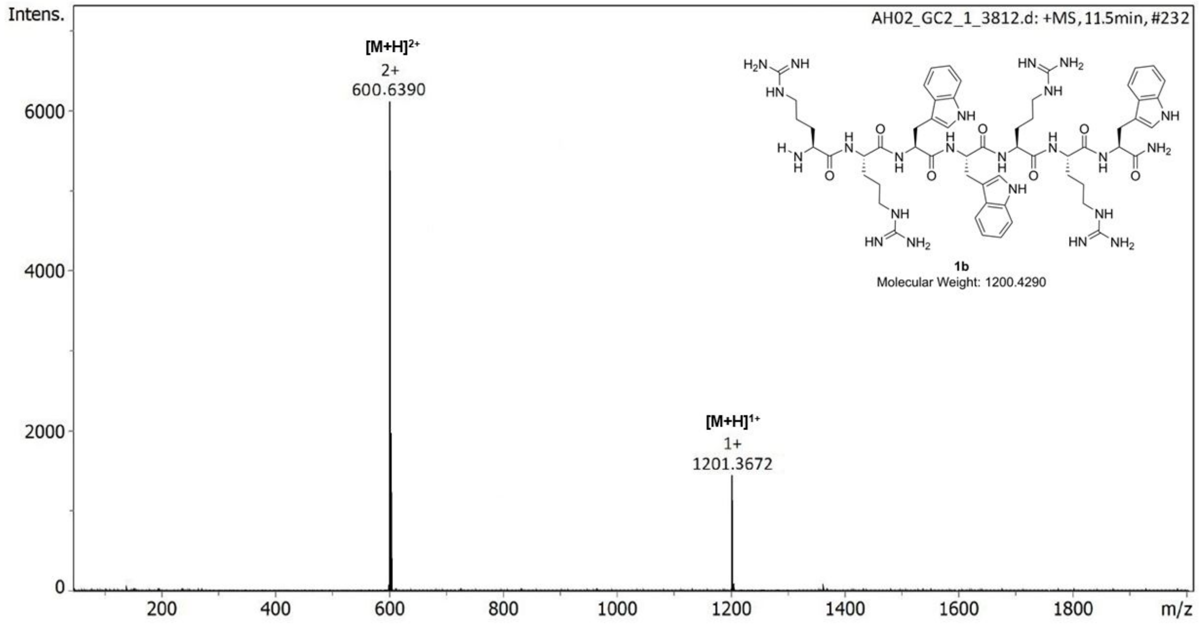

Figure S67. HRMS data of **1b**.

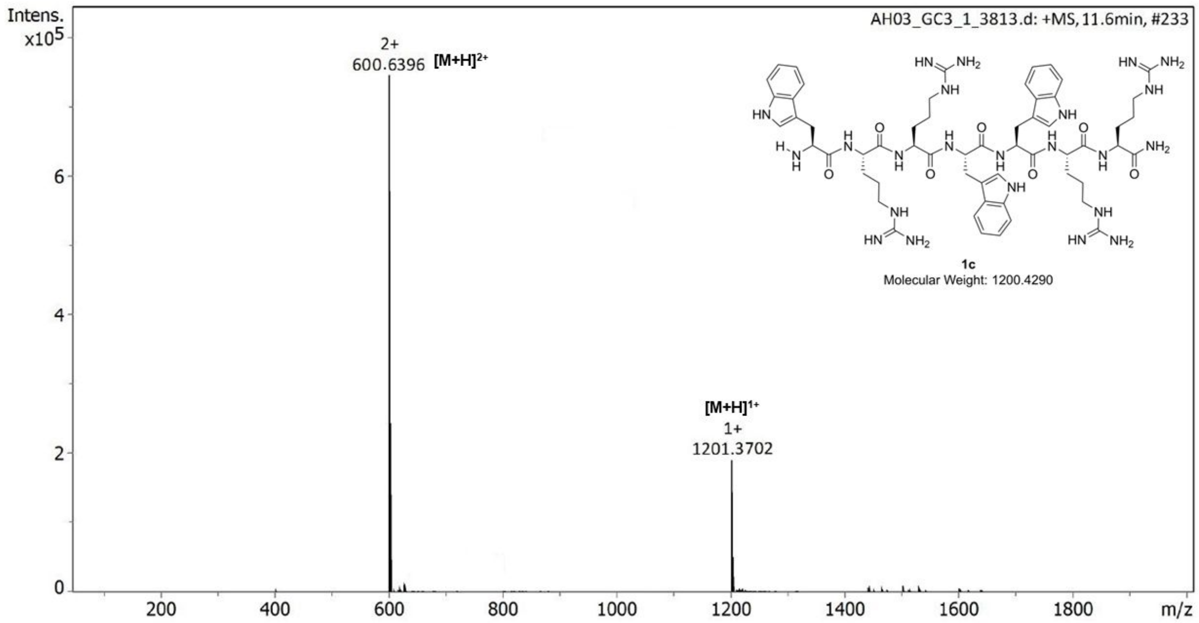

Figure S68. HRMS data of **1c**.

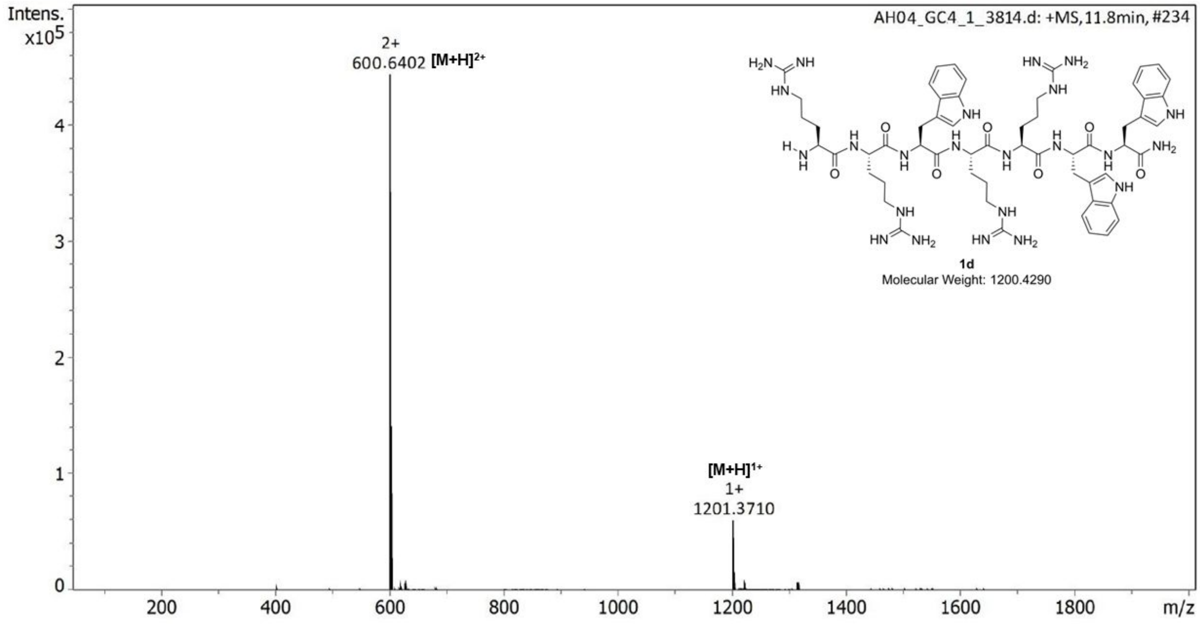

Figure S69. HRMS data of **1d**.

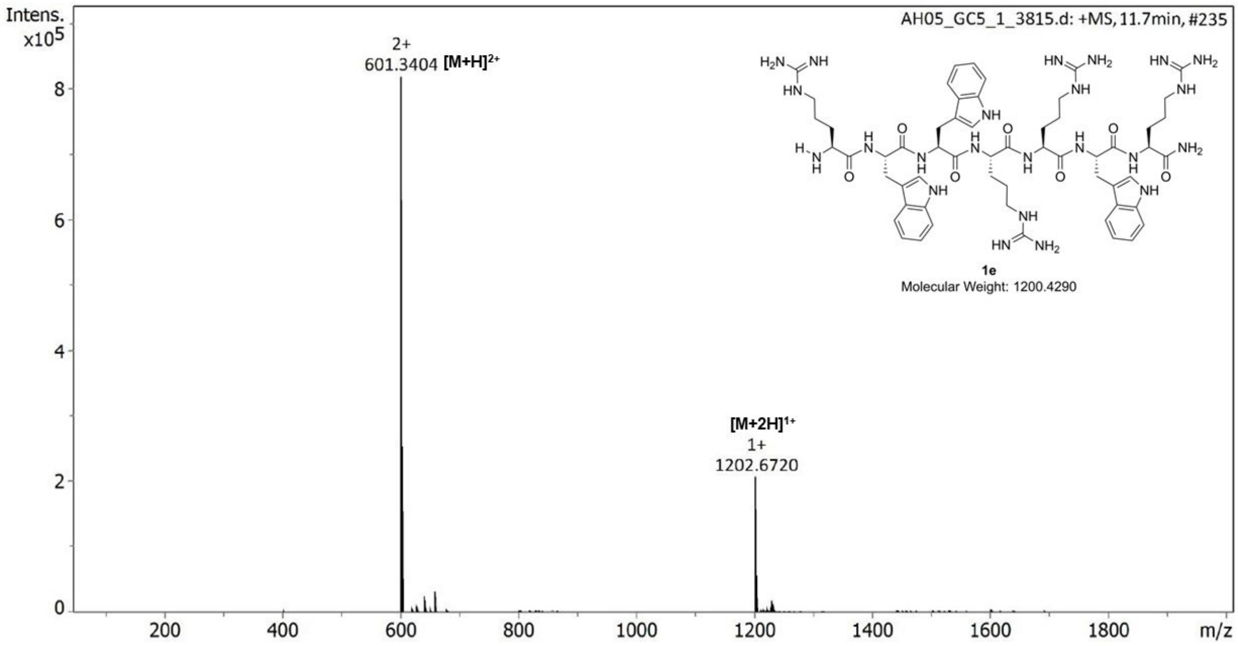

Figure S70. HRMS data of **1e**.

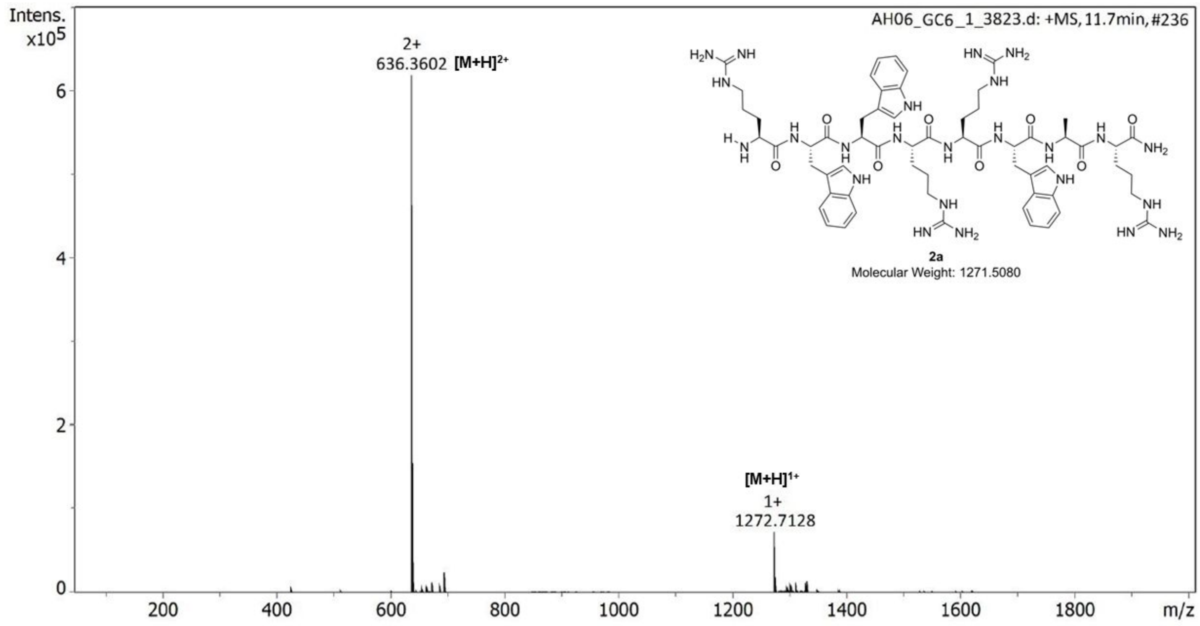

Figure S71. HRMS data of **2a**.

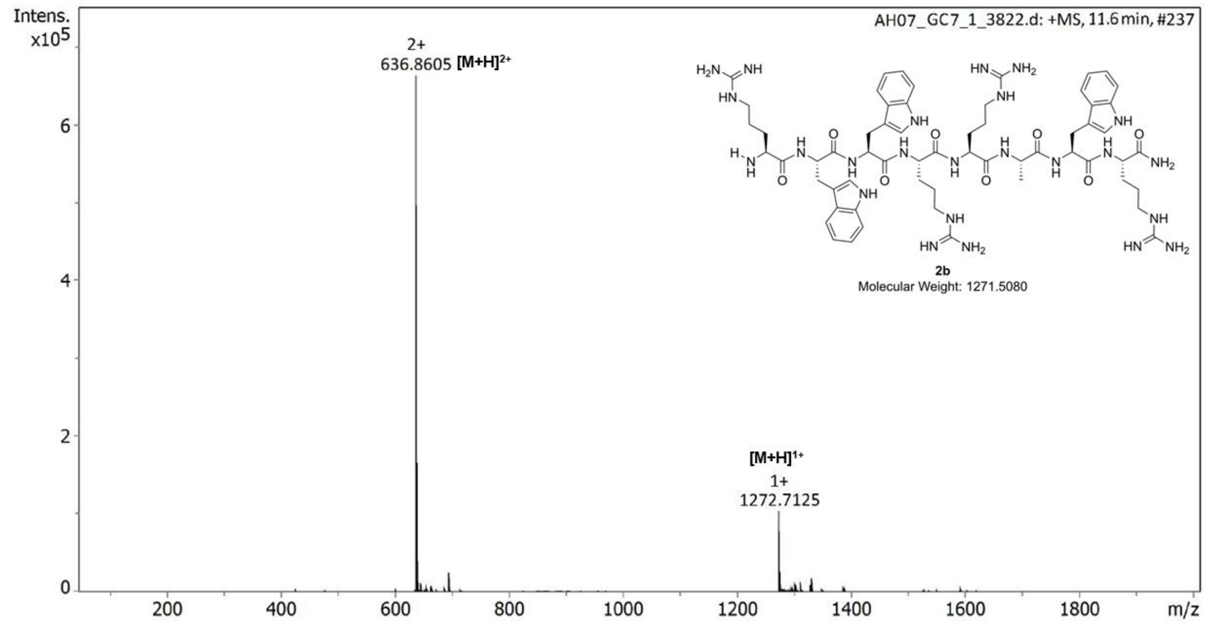

Figure S72. HRMS data of **2b**.

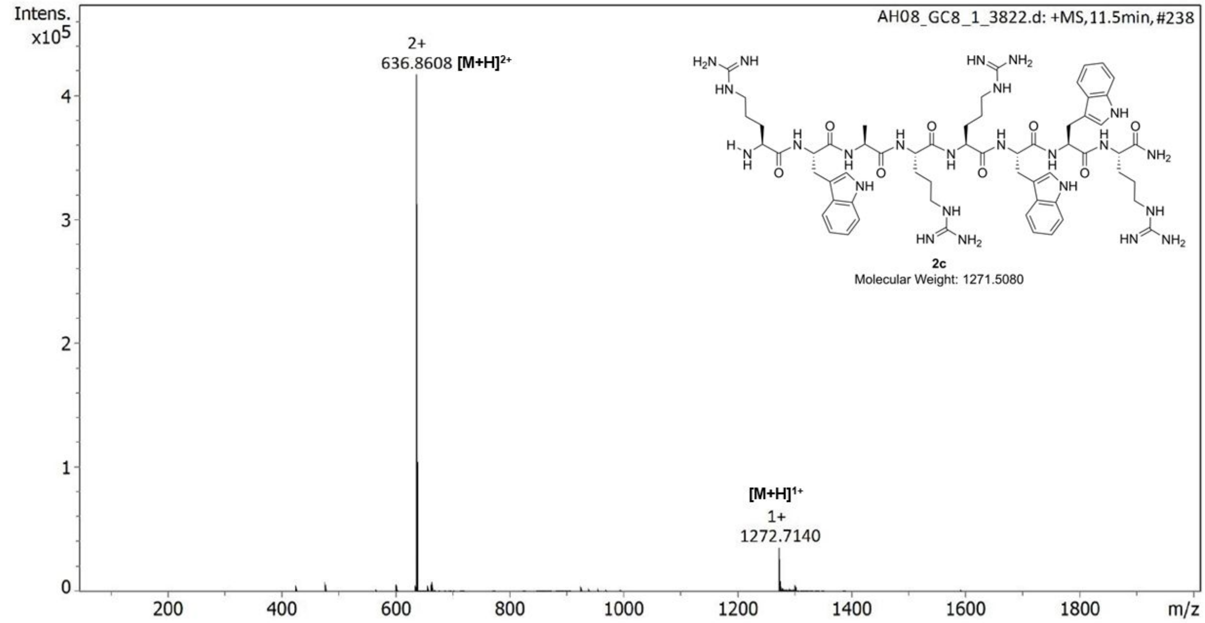

Figure S73. HRMS data of **2c**.

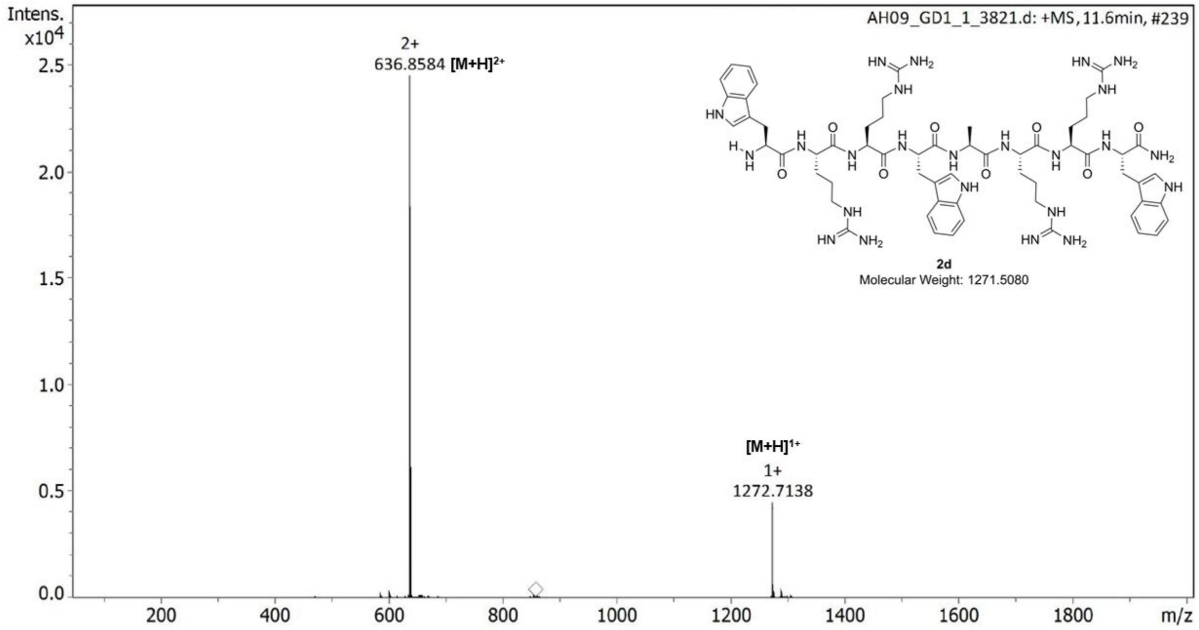

Figure S74. HRMS data of **2d**.

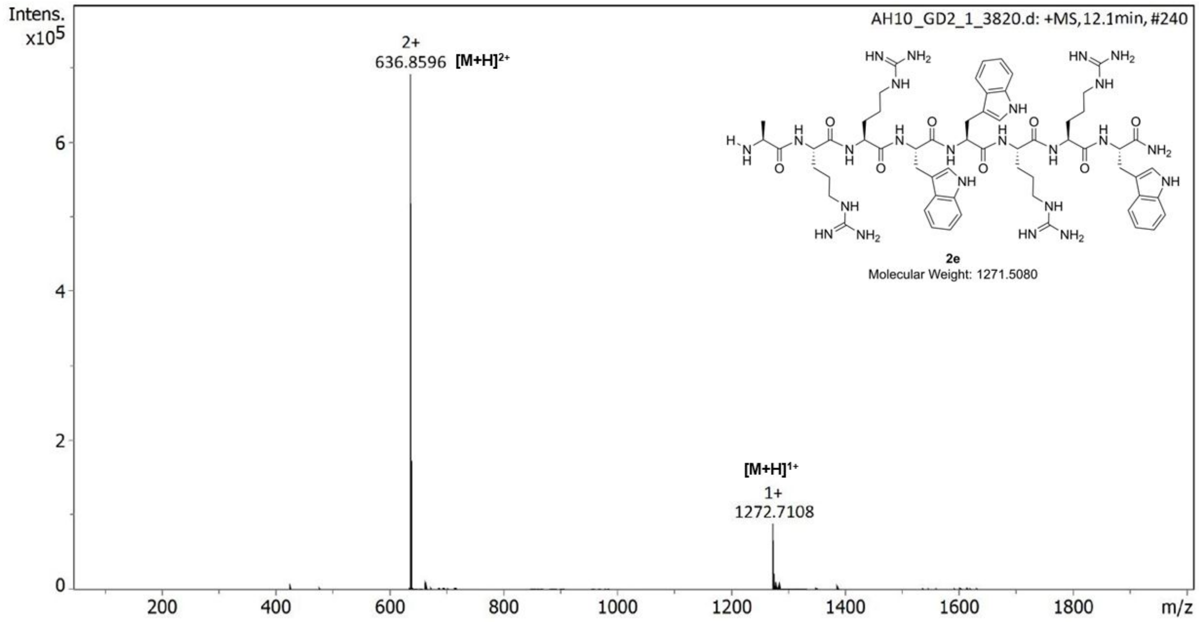

Figure S75. HRMS data of **2e**.

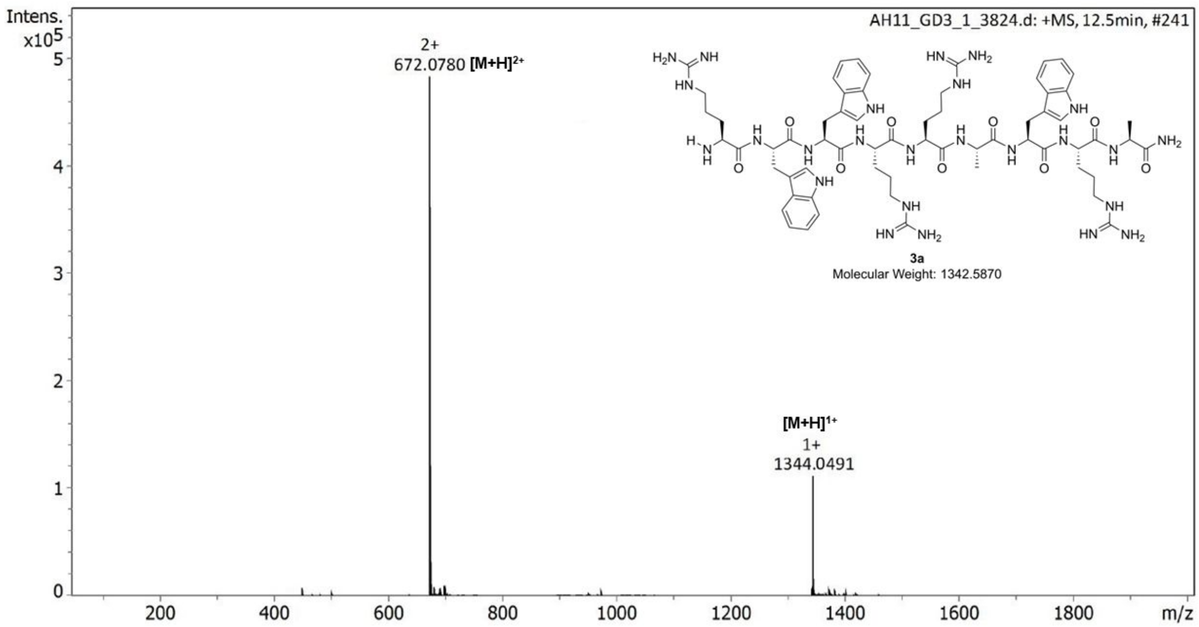

Figure S76. HRMS data of **3a**.

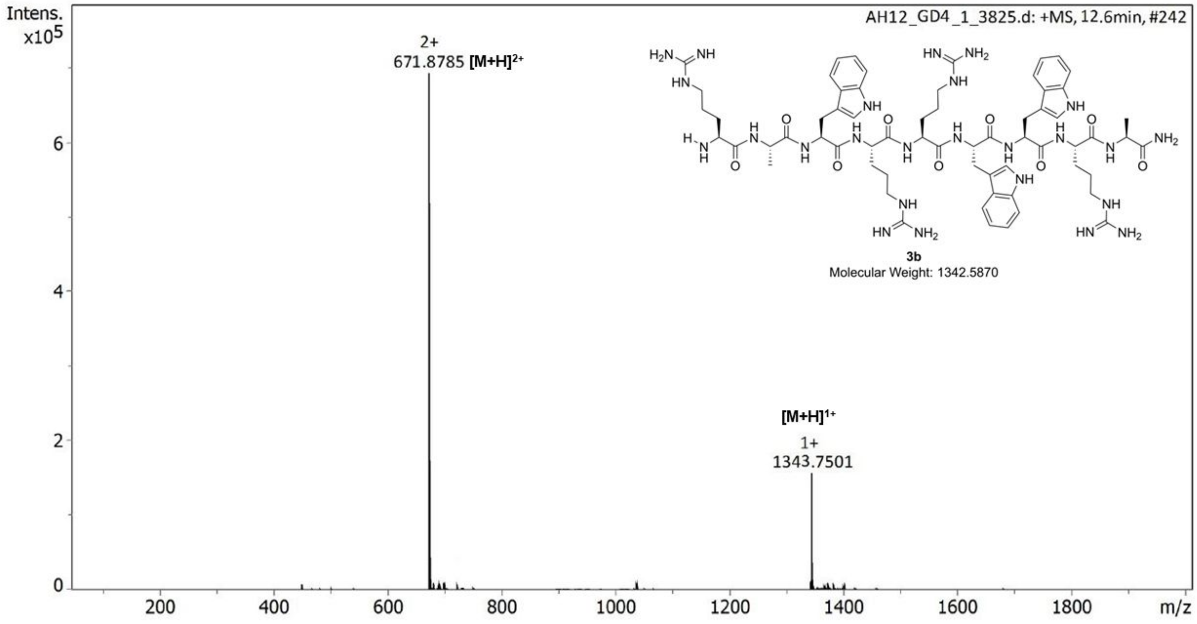

Figure S77. HRMS data of **3b**.

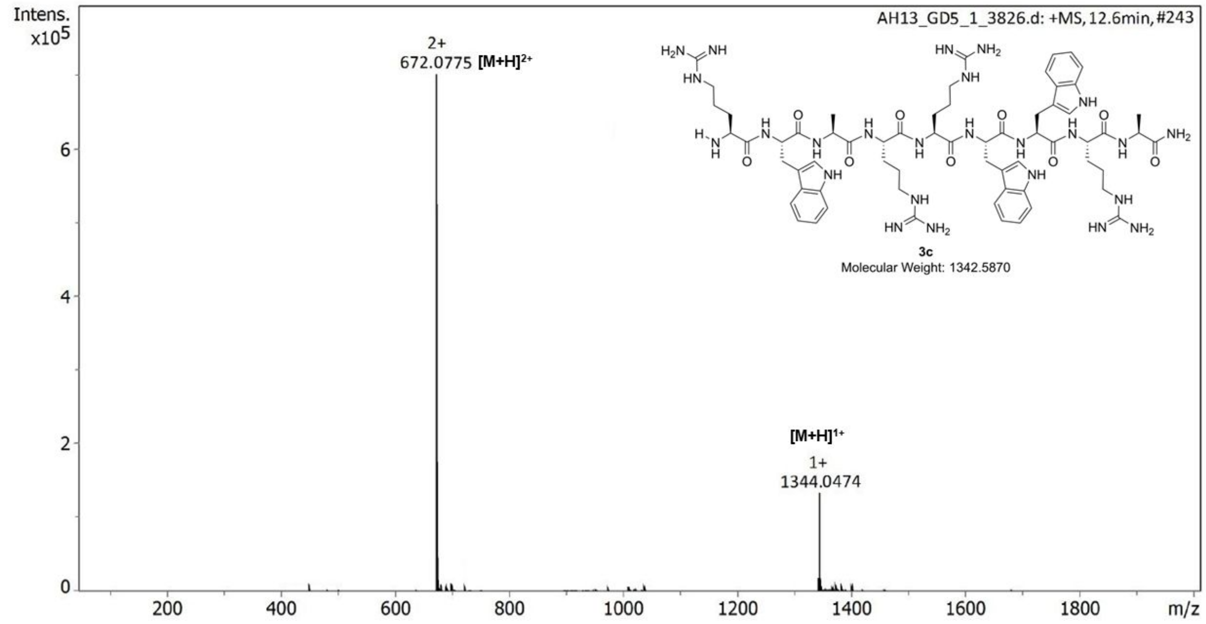

Figure S78. HRMS data of **3c**.

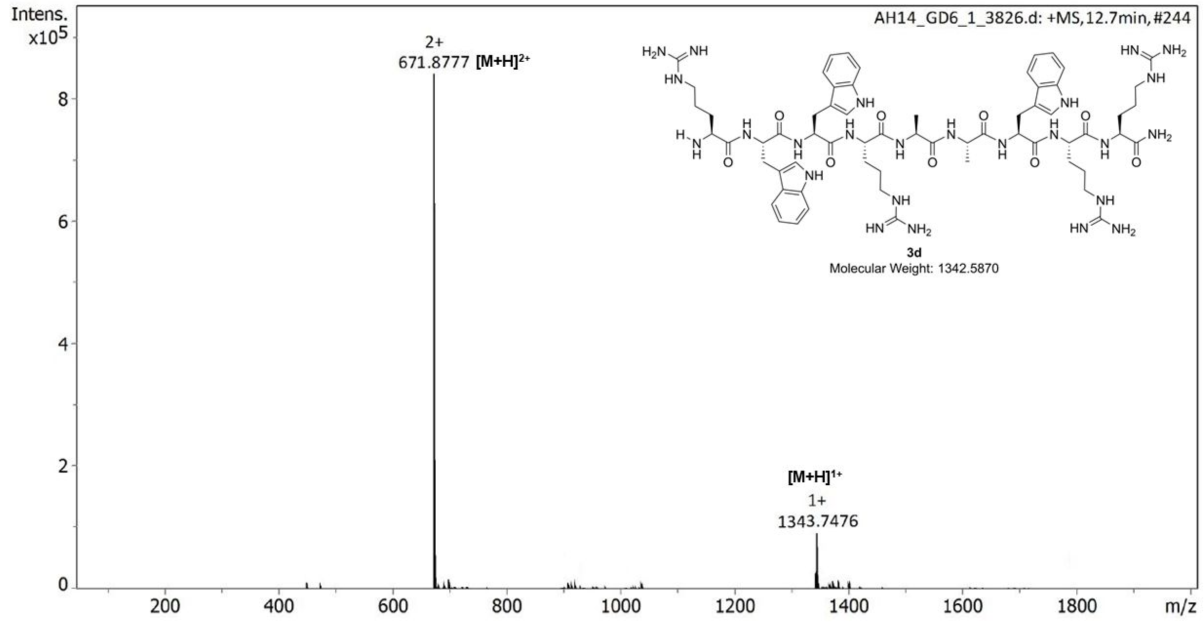

Figure S79. HRMS data of **3d**.

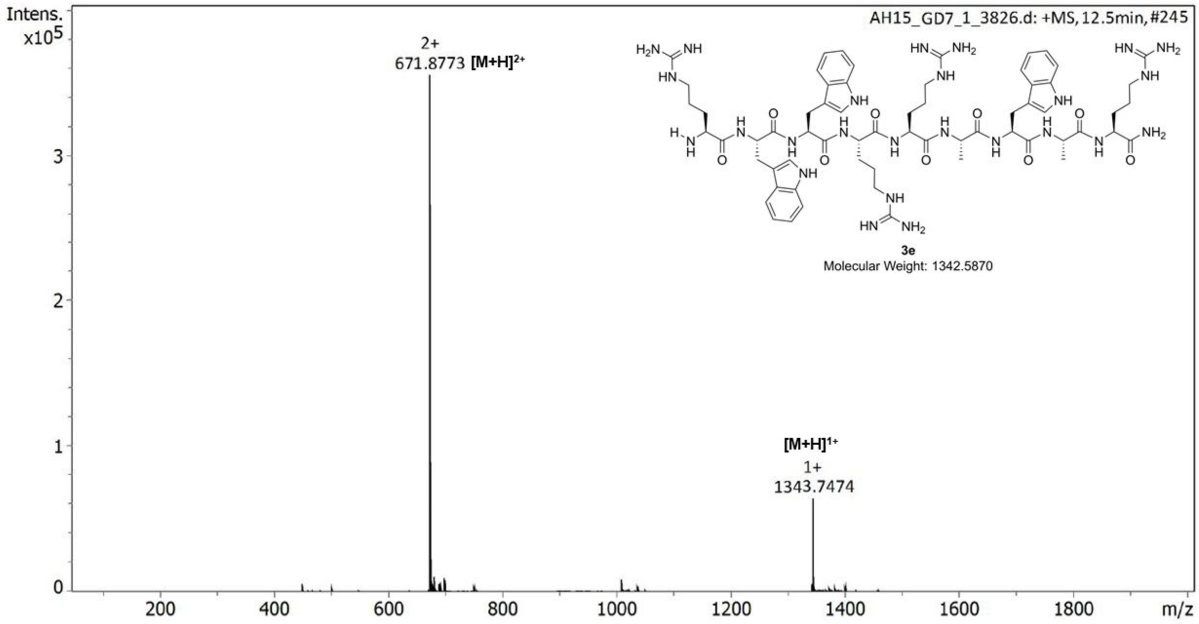

Figure S80. HRMS data of **3e**.

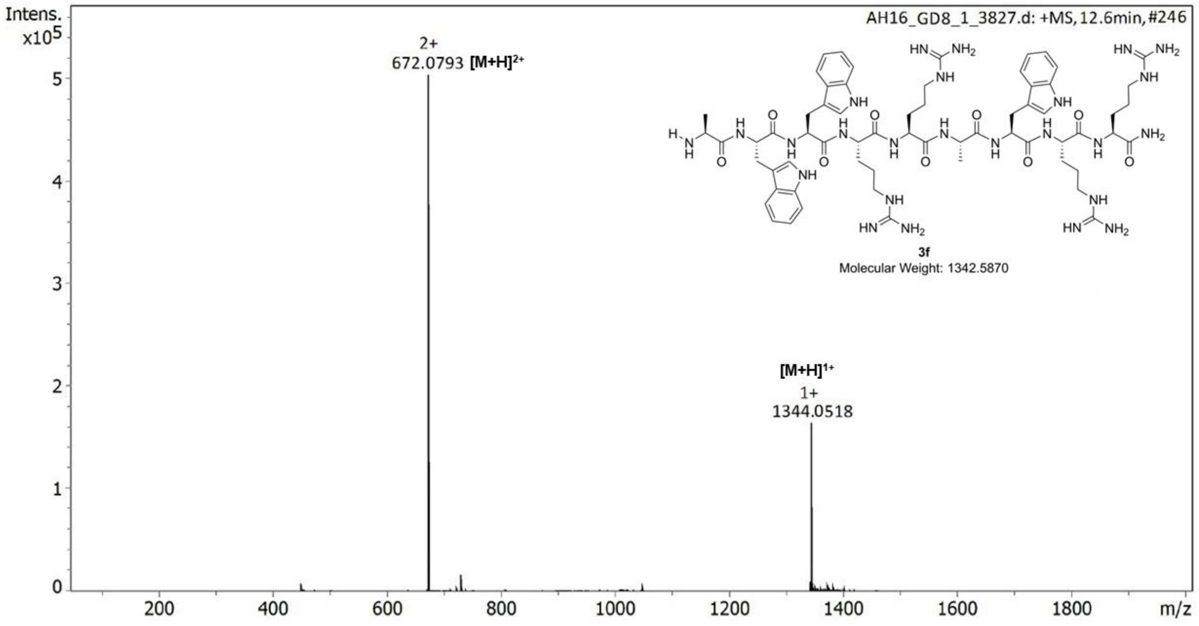

Figure S81. HRMS data of **3f**.

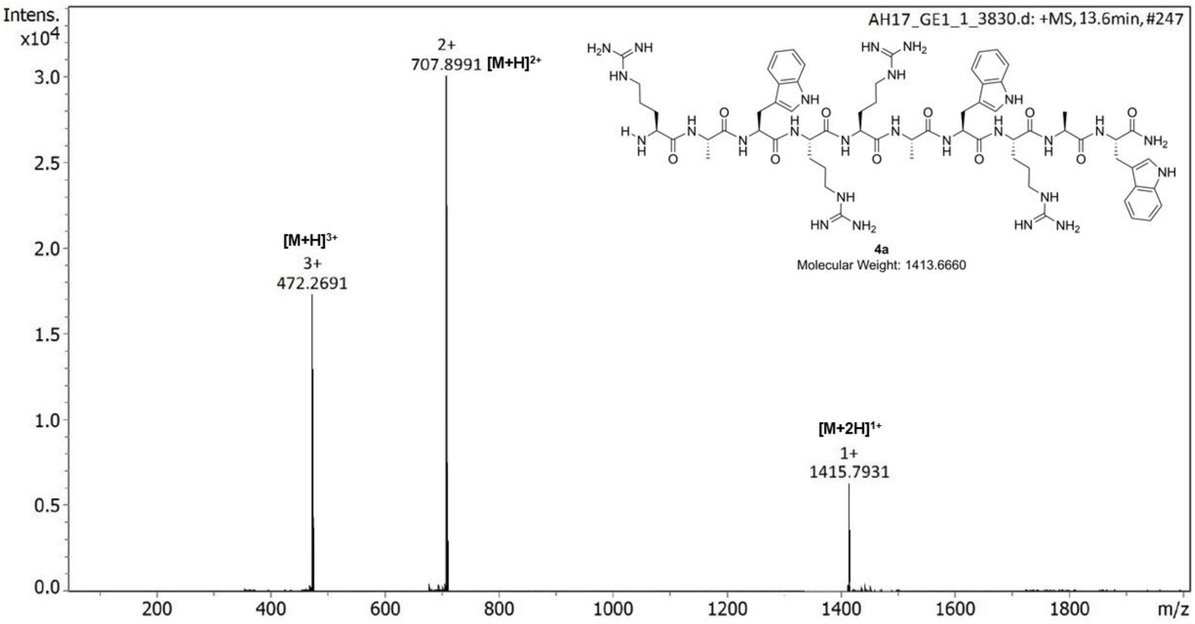

Figure S82. HRMS data of **4a**.

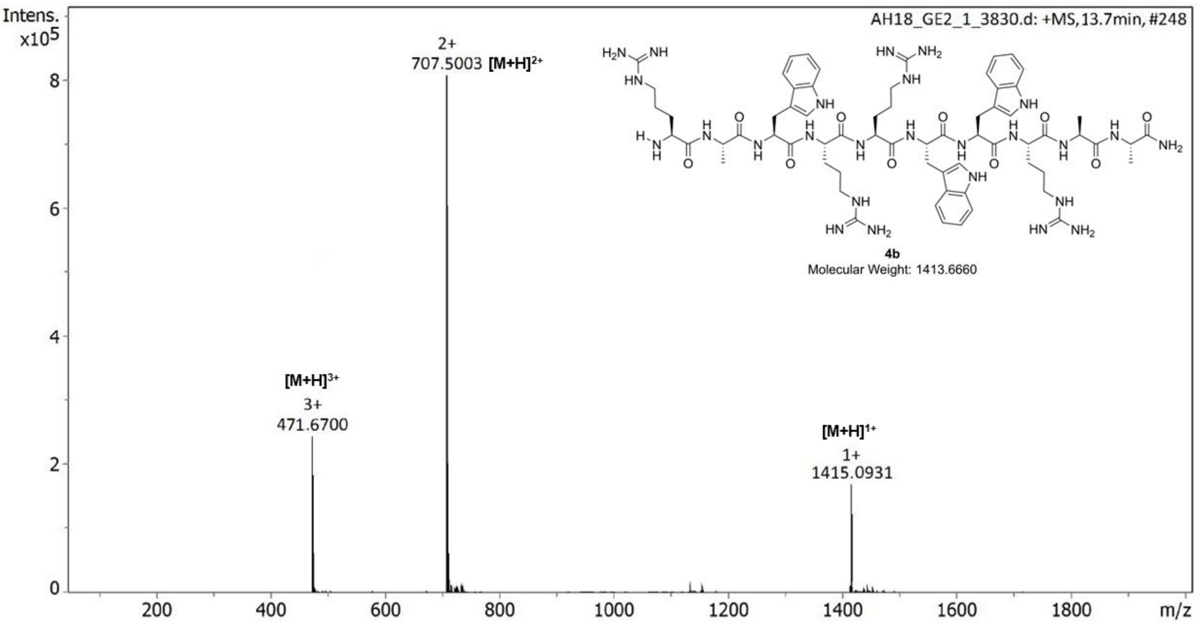

Figure S83. HRMS data of **4b**.

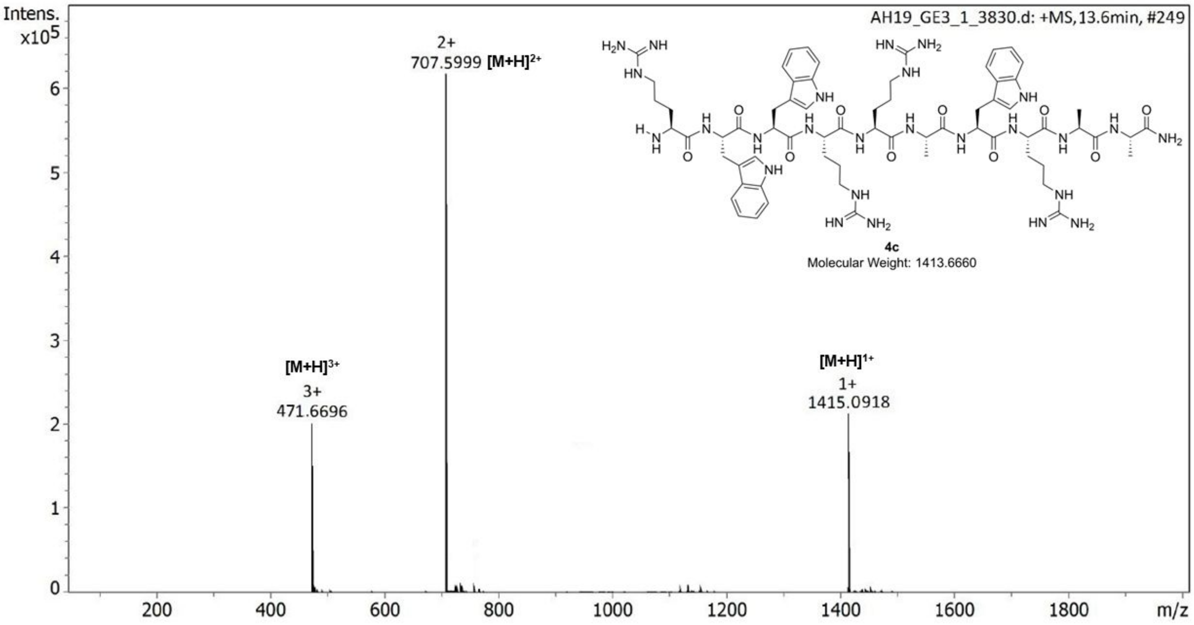

Figure S84. HRMS data of **4c**.

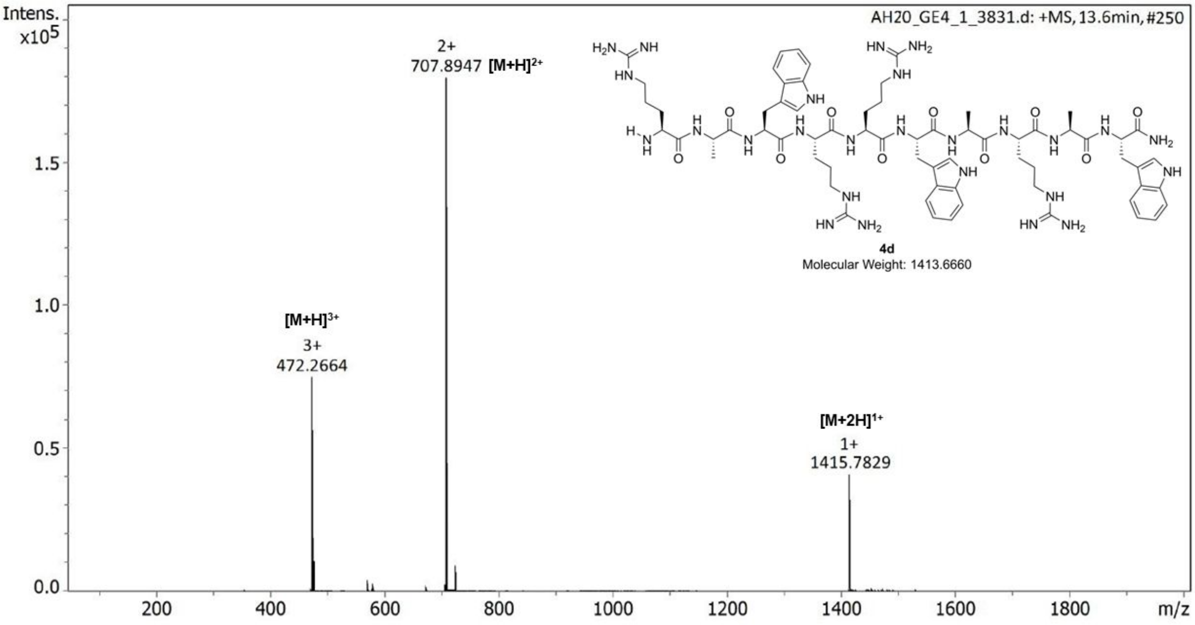

Figure S85. HRMS data of **4d**.

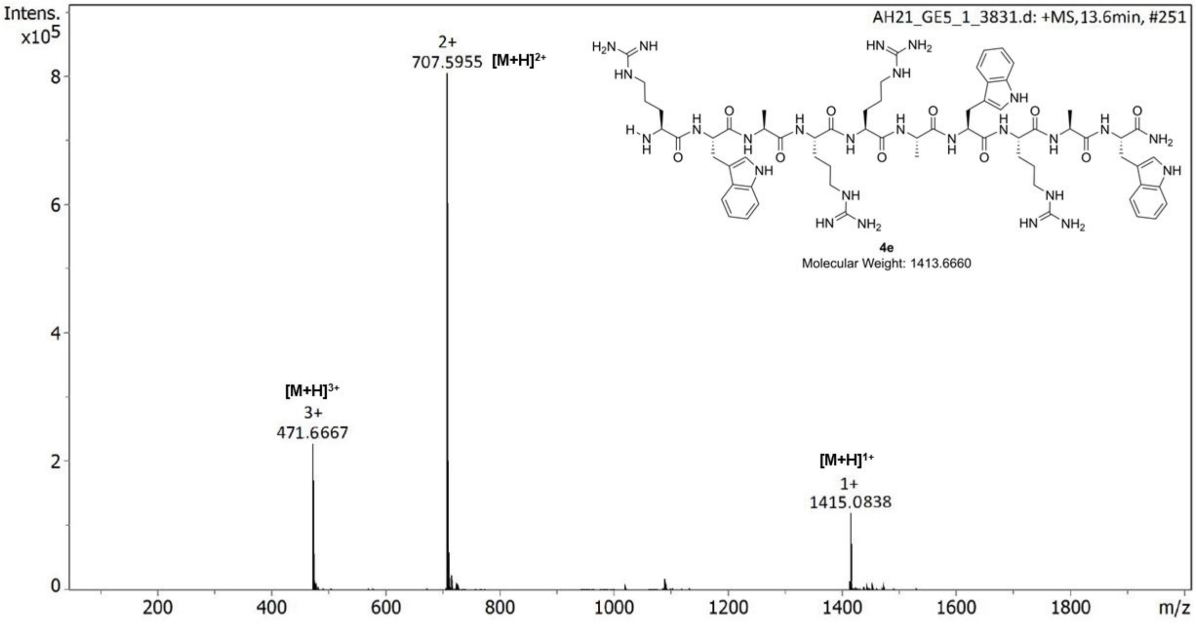

Figure S86. HRMS data of **4e**.

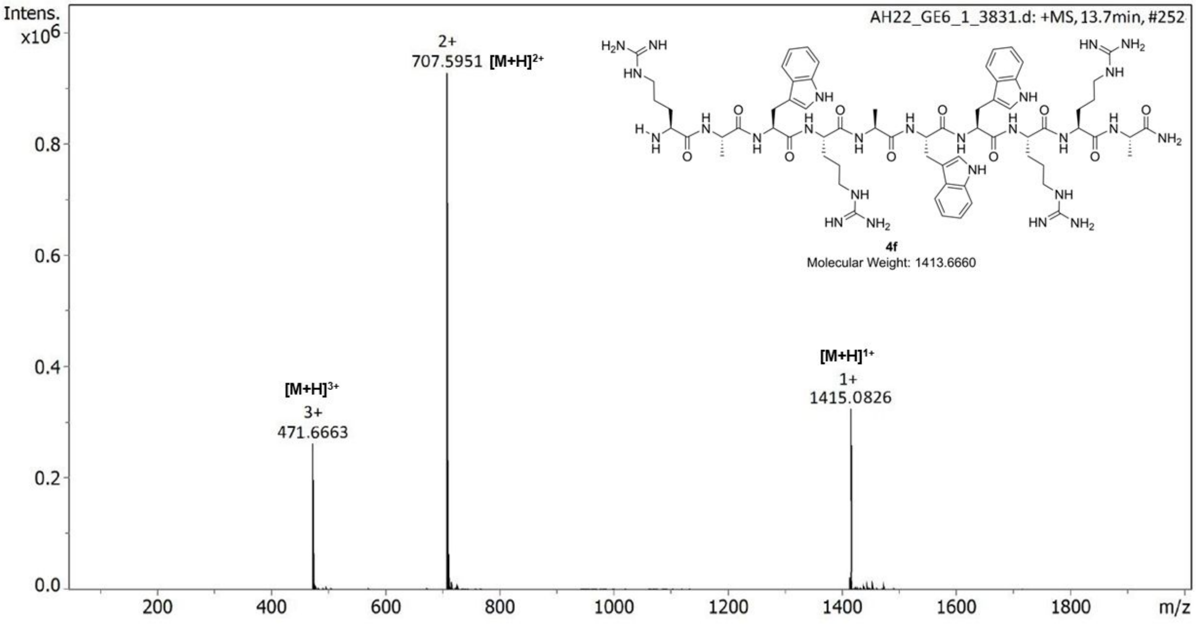

Figure S87. HRMS data of **4f**.

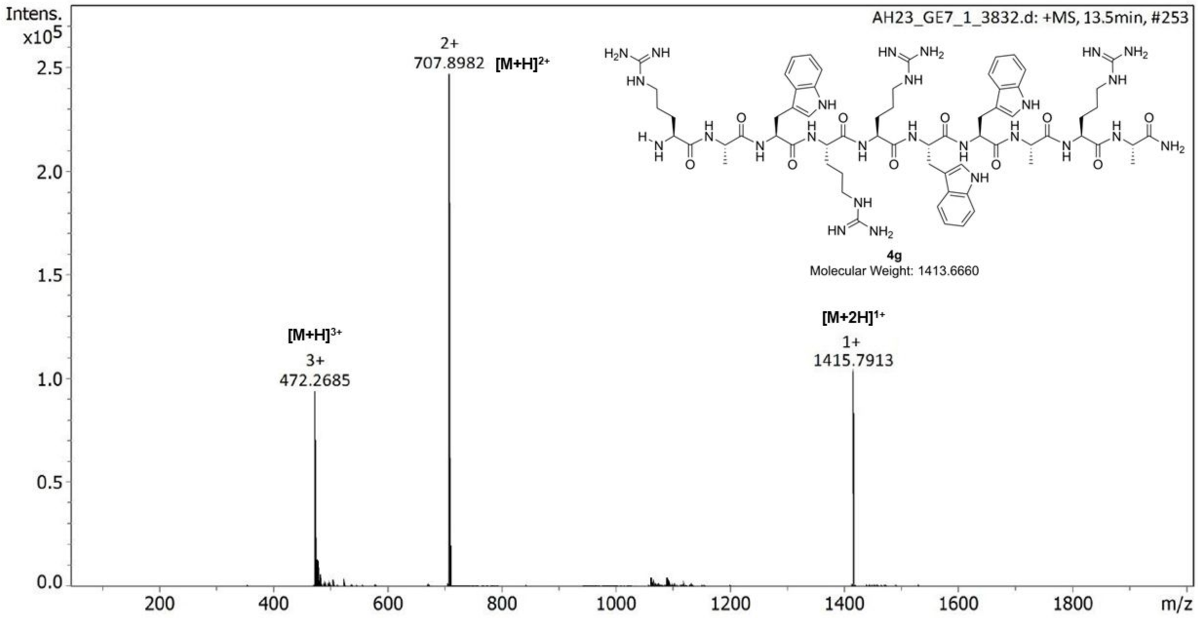

Figure S88. HRMS data of **4g**.

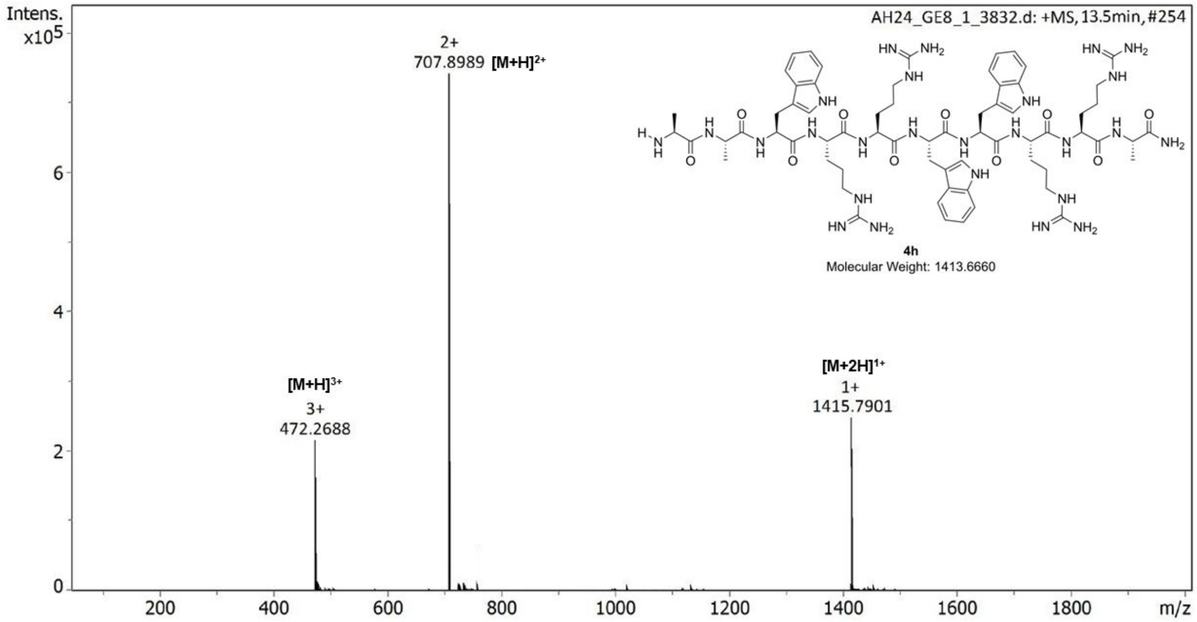

Figure S89. HRMS data of **4h**.

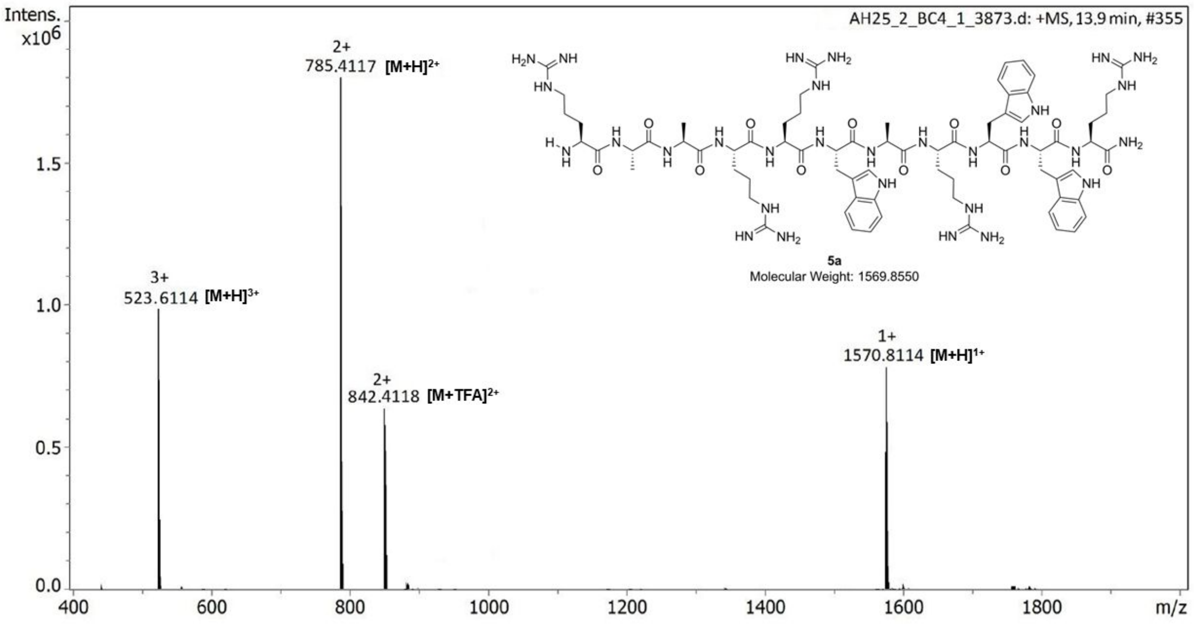

Figure S90. HRMS data of **5a**.

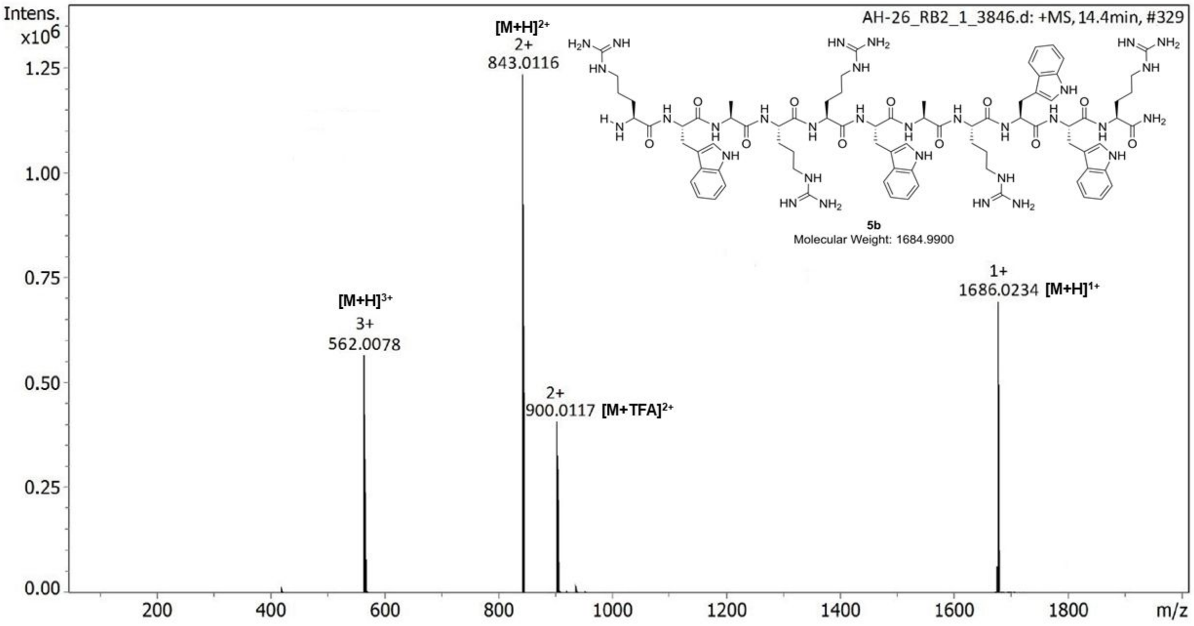

Figure S91. HRMS data of **5b**.

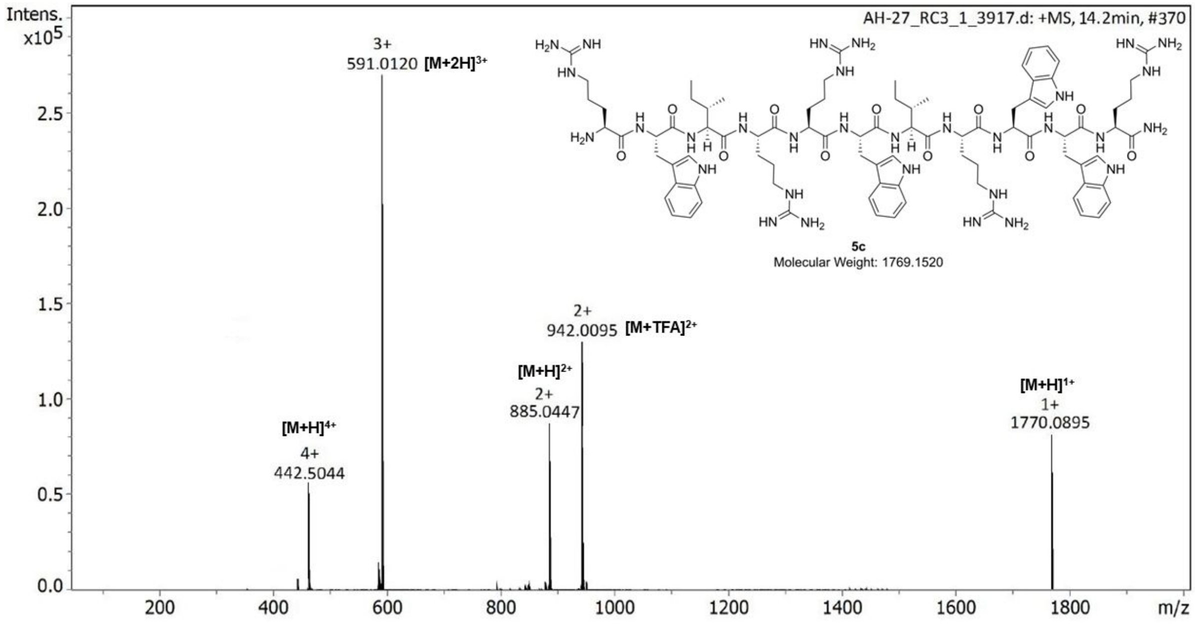

Figure S92. HRMS data of **5c**.

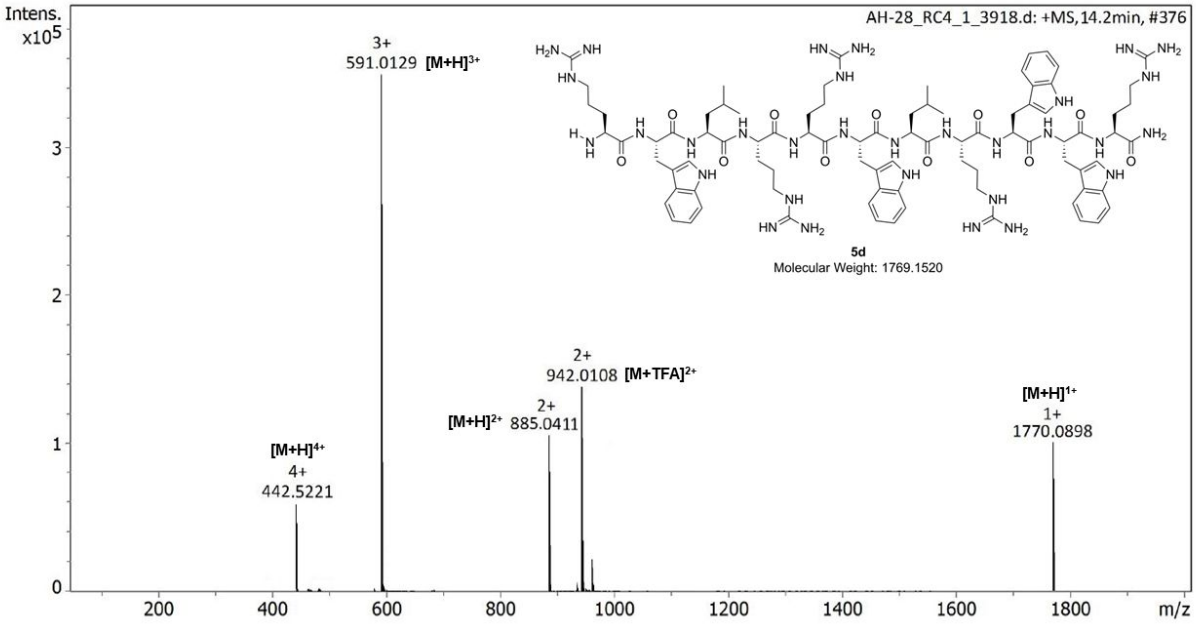

Figure S93. HRMS data of **5d**.

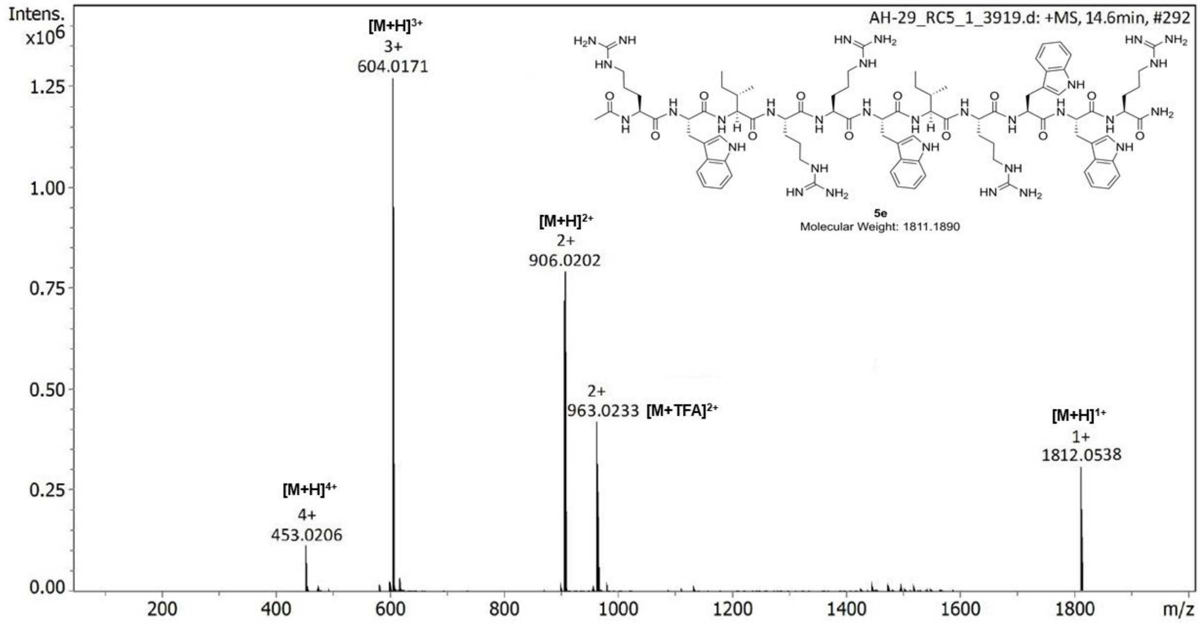

Figure S94. HRMS data of **5e**.

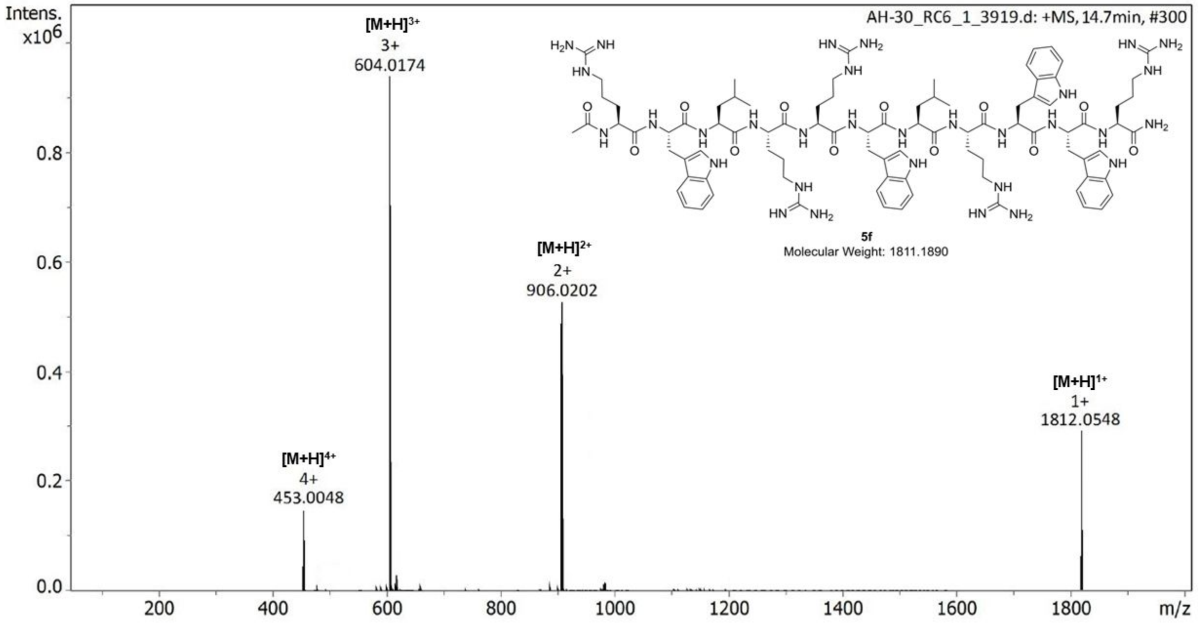

Figure S95. HRMS data of **5f**.

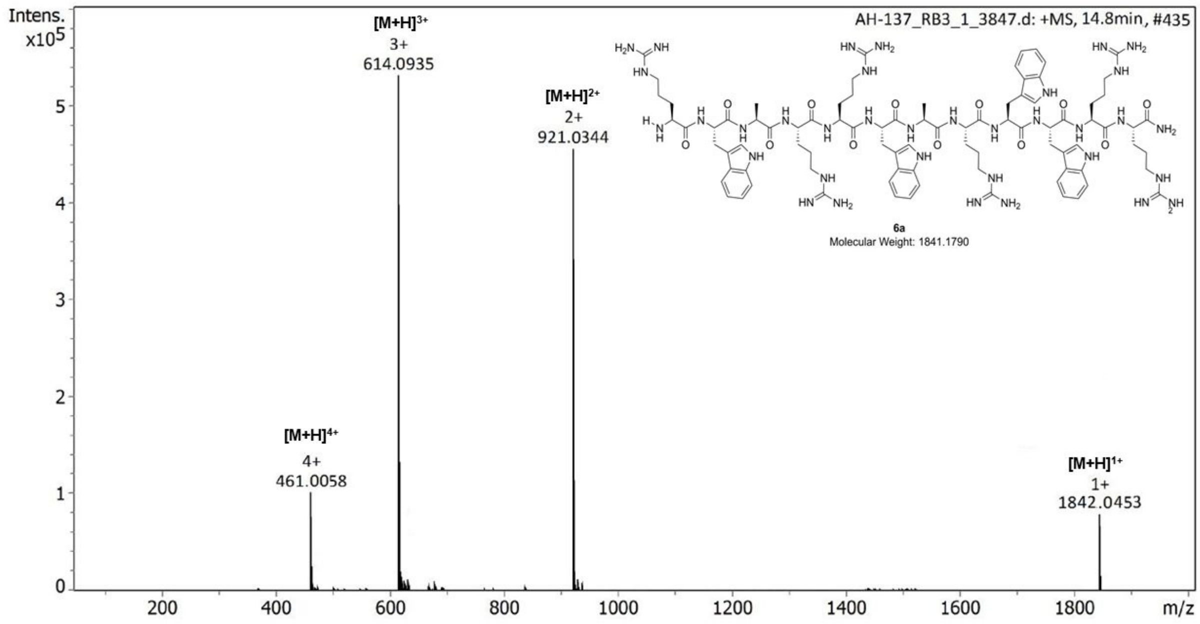

Figure S96. HRMS data of **6a**.

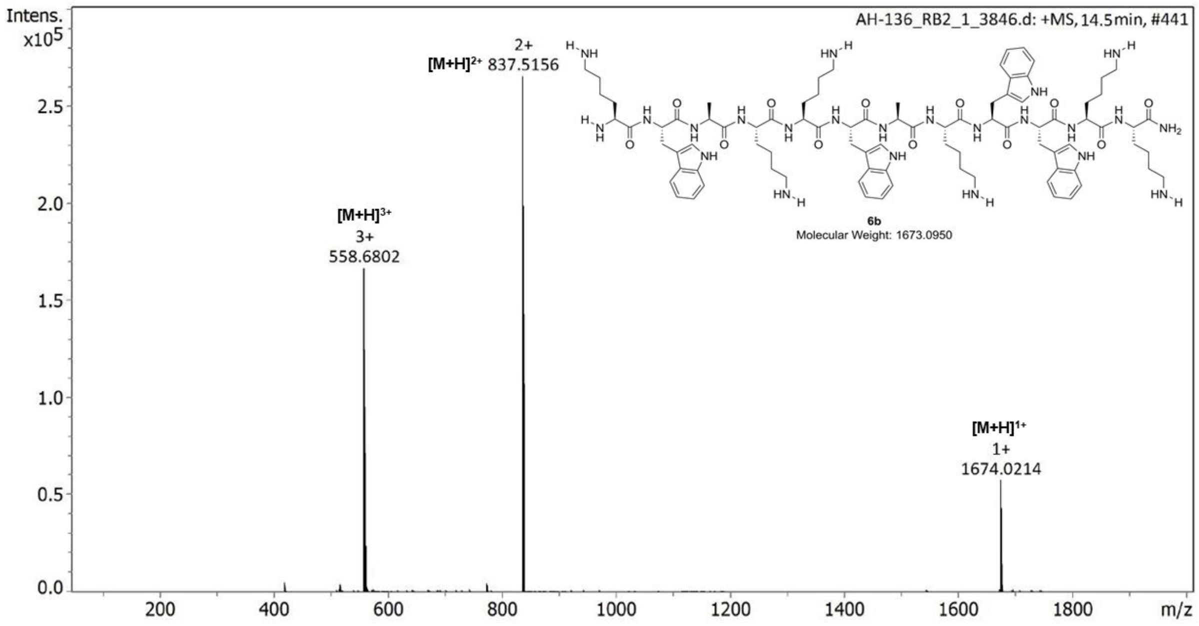

Figure S97. HRMS data of **6b**.

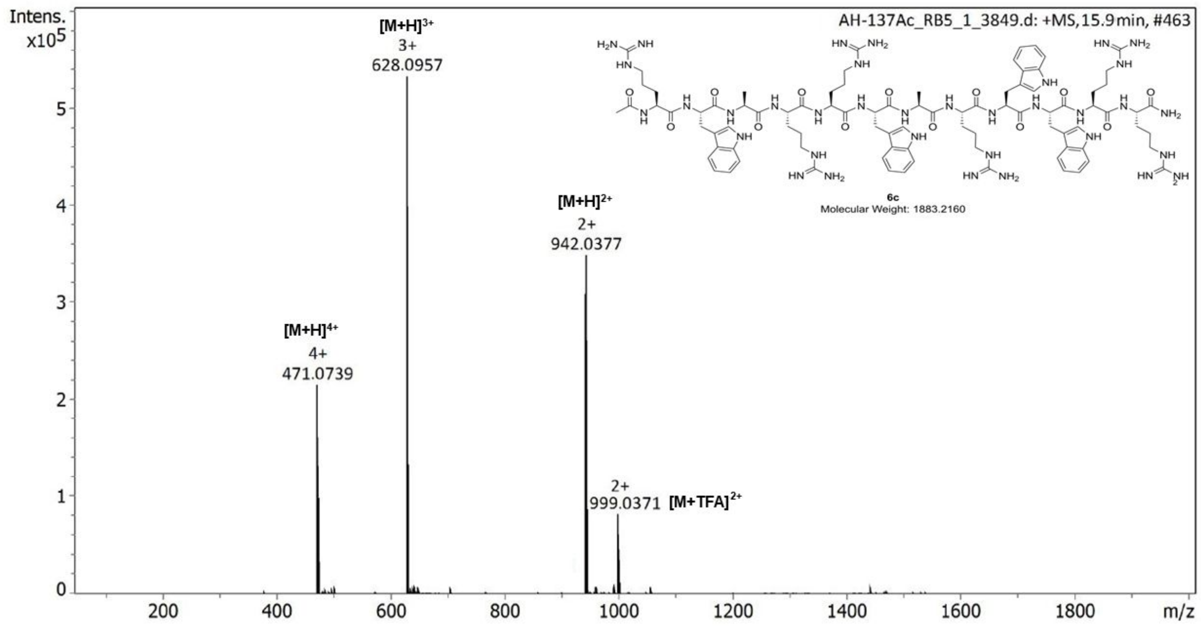

Figure S98. HRMS data of **6c**.

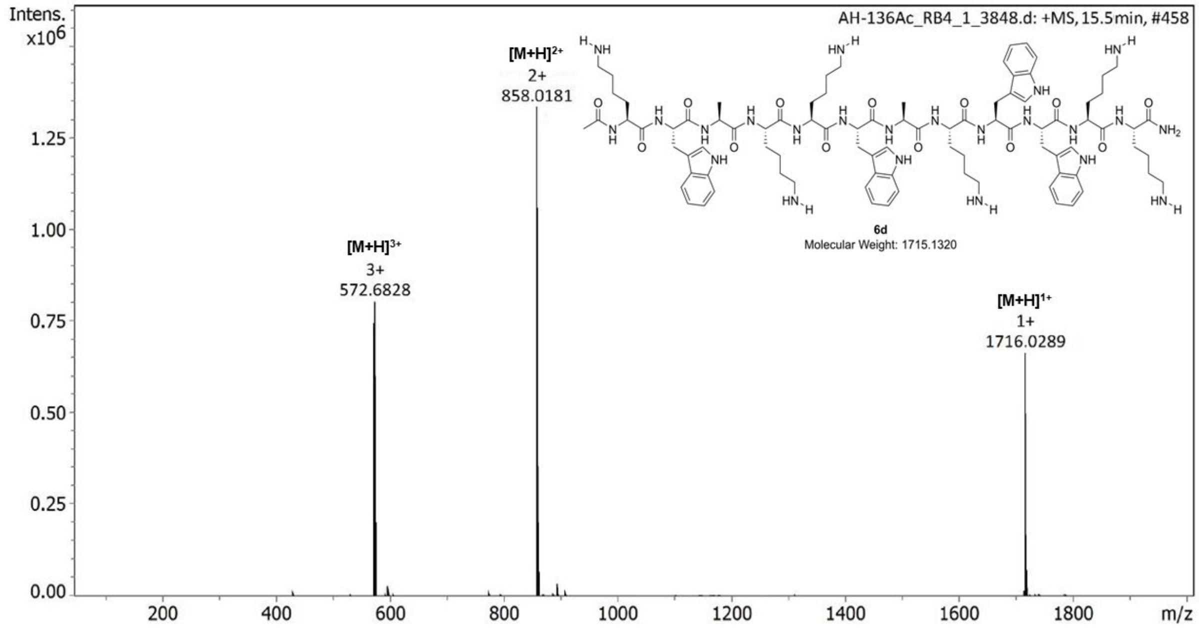

Figure S99. HRMS data of **6d**.

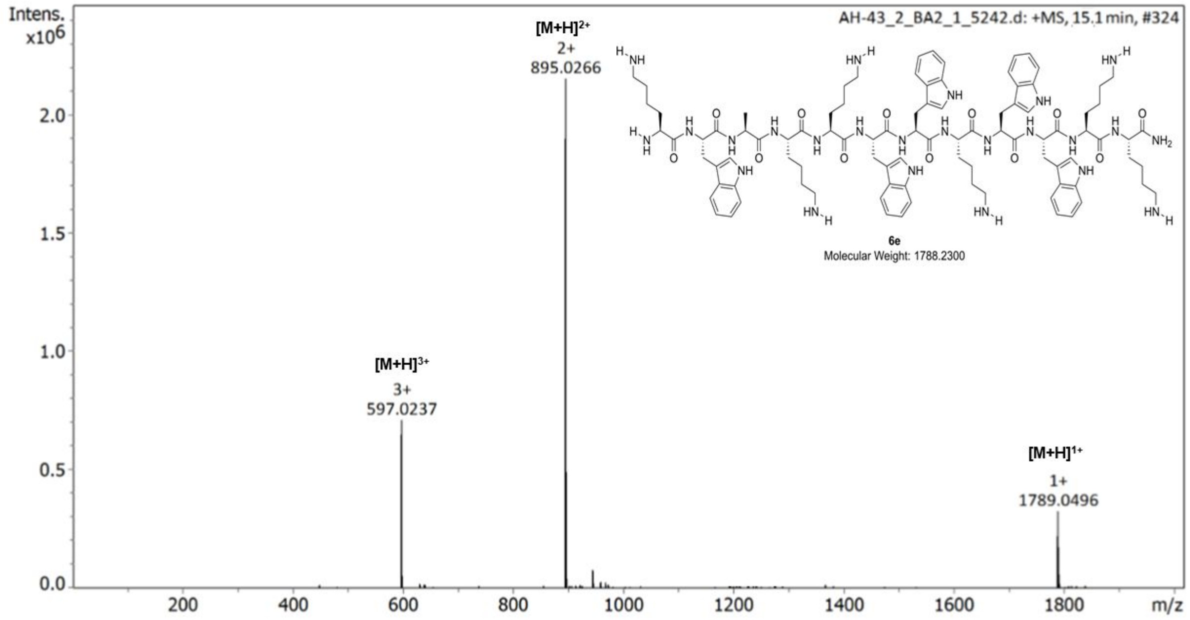

Figure S100. HRMS data of **6e**.

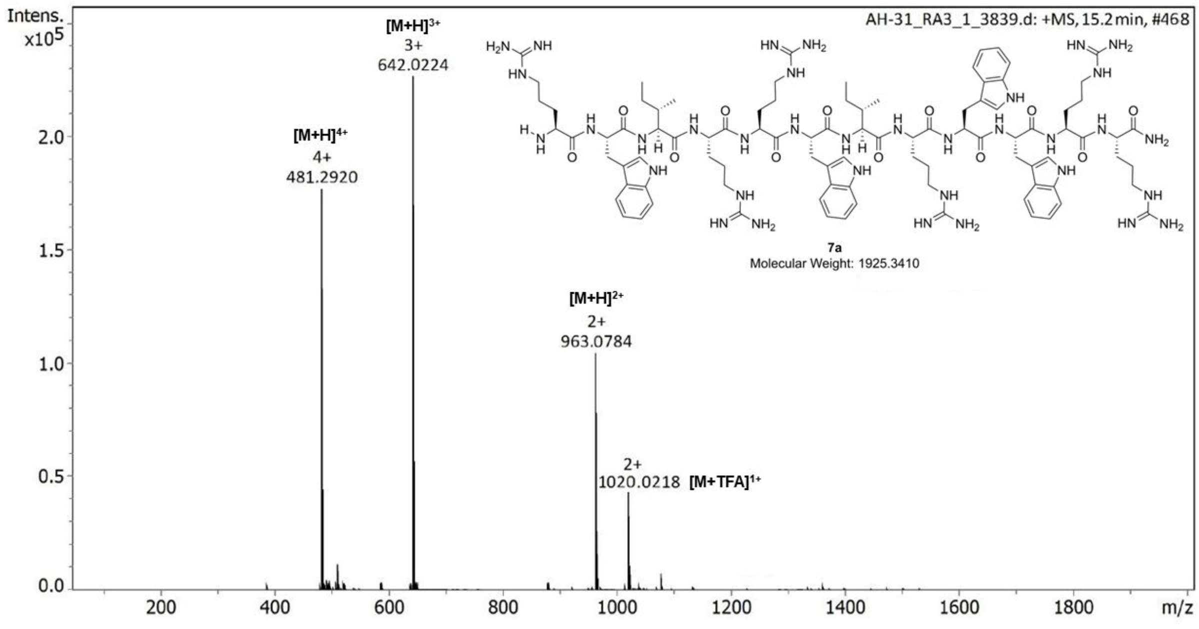

Figure S101. HRMS data of **7a**.

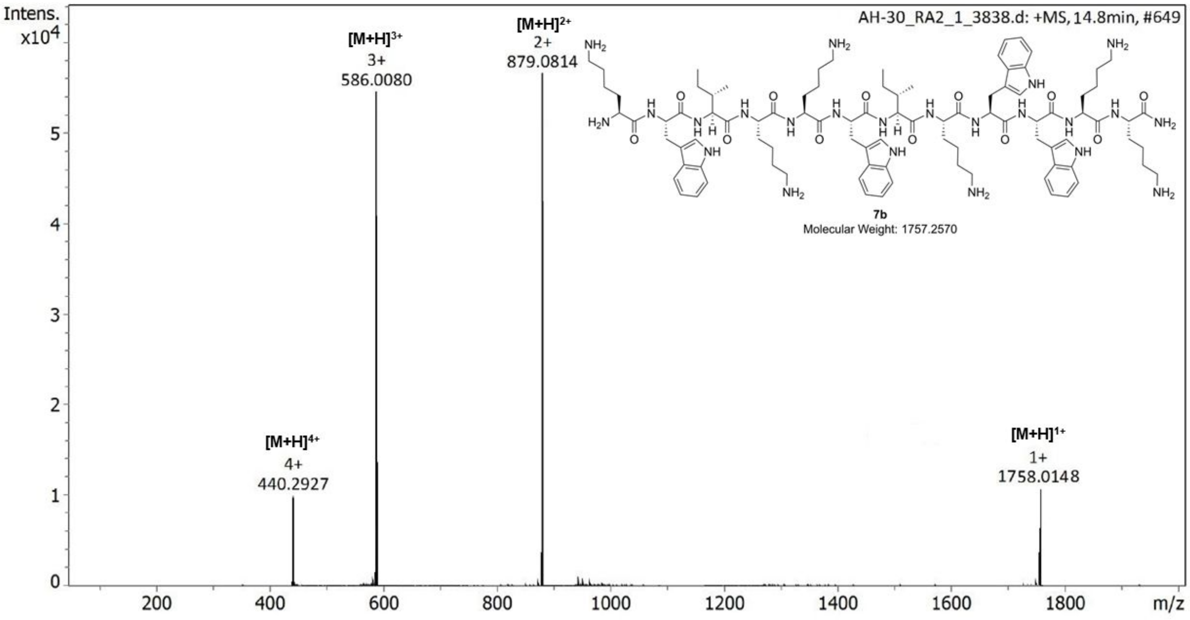

Figure S102. HRMS data of **7b**.

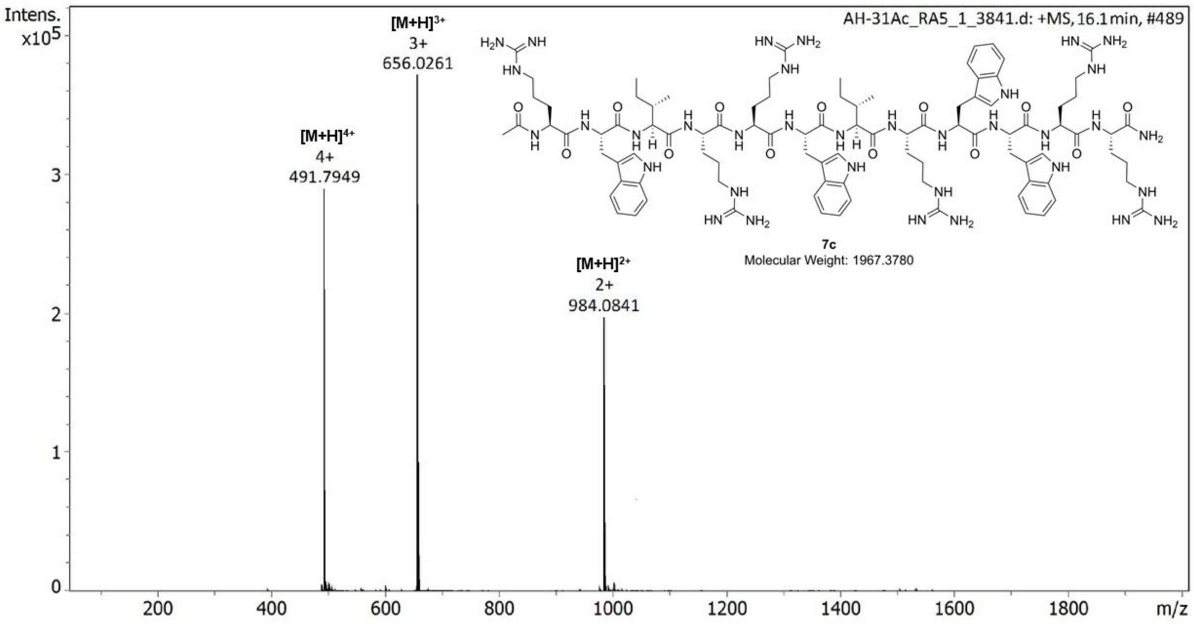

Figure S103. HRMS data of **7c**.

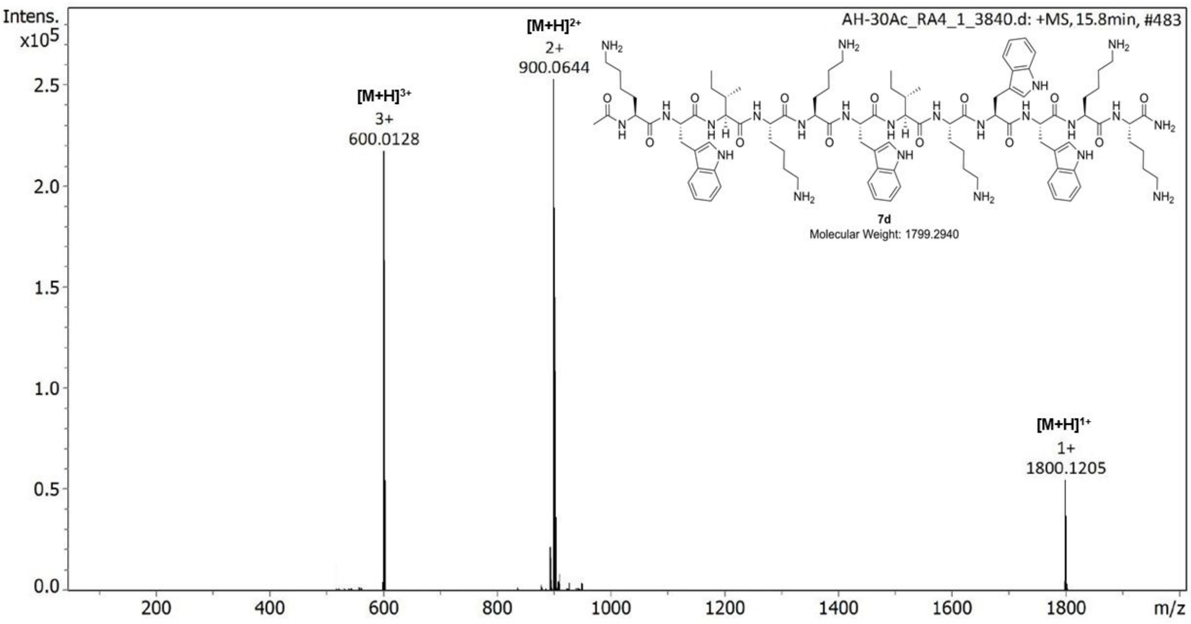

Figure S104. HRMS data of **7d**.

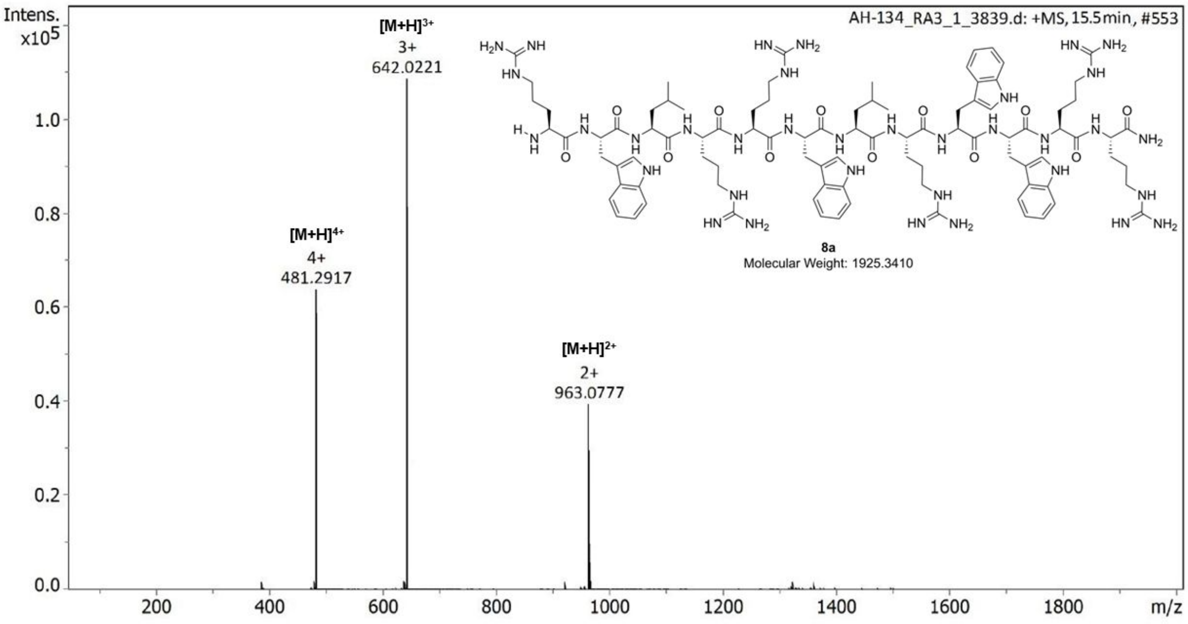

Figure S105. HRMS data of **8a**.

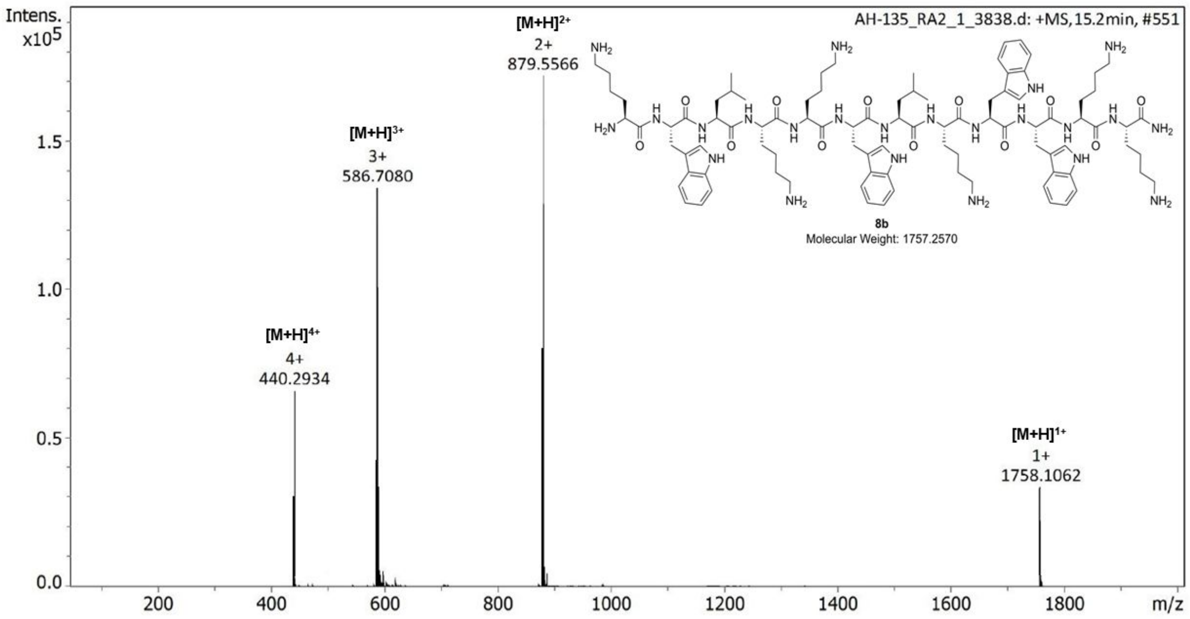

Figure S106. HRMS data of **8b**.

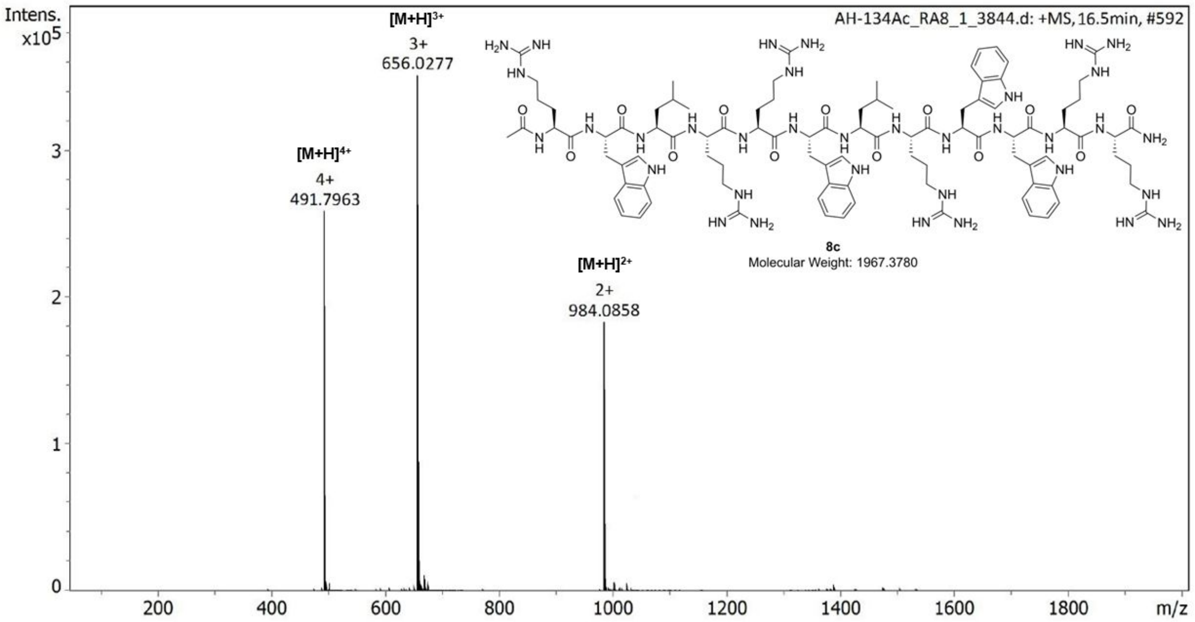

Figure S107. HRMS data of **8c**.

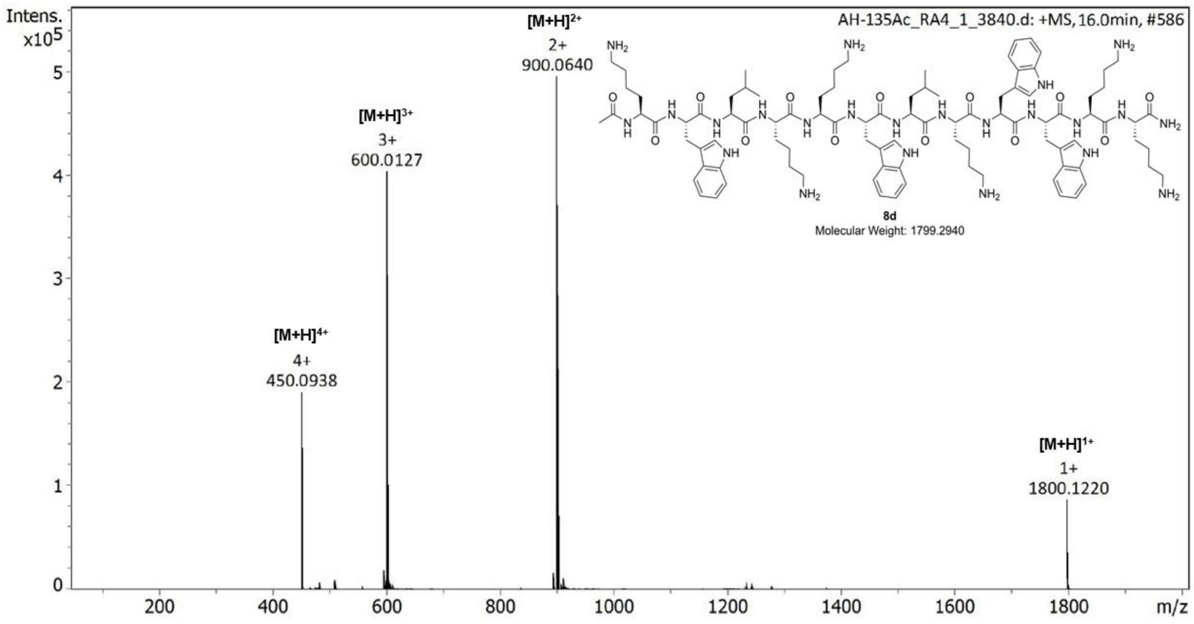

Figure S108. HRMS data of **8d**.

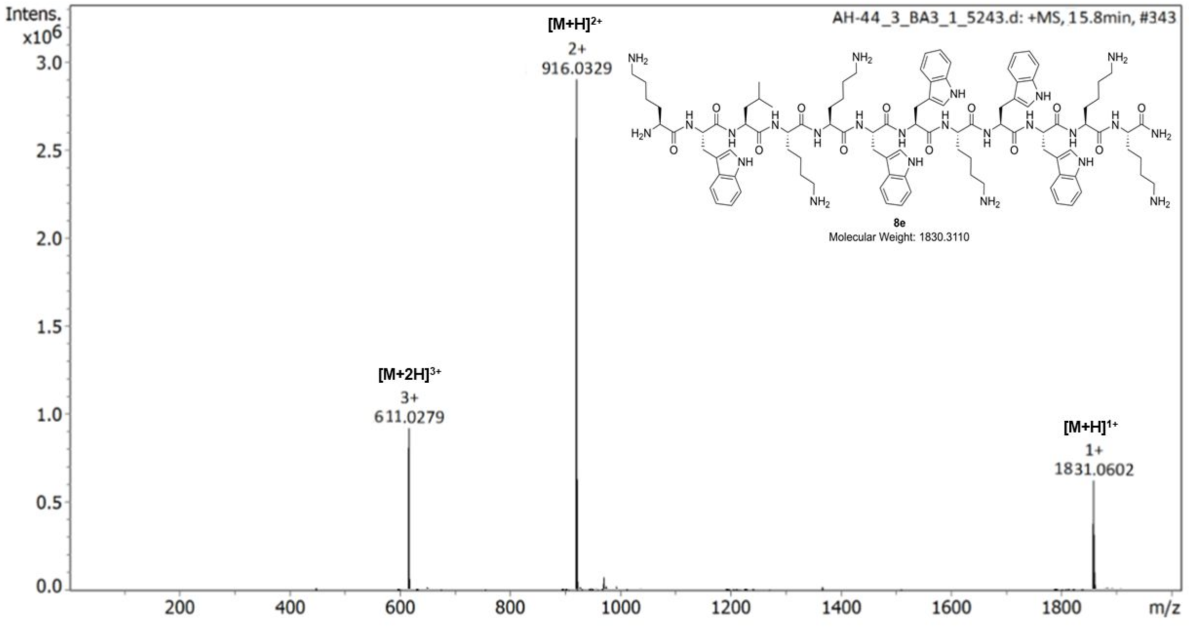

Figure S109. HRMS data of **8e**.

10. High Resolution NMR data

Table S4. Chemical shifts of peptide 6b.

Residue		H^N	$H\alpha$	$H\beta$	$H\beta^2$	$H\beta^3$	$H\gamma^2$	$H\gamma^3$	$H\delta^1$	$H\delta^2$	$H\delta^3$	$H\varepsilon^1$	$H\varepsilon^2$	$H\varepsilon^3$	$H\zeta^2$	$H\zeta^3$	$H\eta^2$
1	Lys		3.893		1.774	1.774	1.315	1.315		1.595	1.595		2.886	2.886			
2	Trp	8.620	4.602		3.189	3.189			7.187			10.048		7.544	7.411	7.075	7.184
3	Ala	8.033	4.175	1.207													
4	Lys	7.916	3.923		1.506	1.506	1.158	1.158		1.509	1.509		2.750	2.750			
5	Lys	8.121	4.125		1.563	1.563	1.140	1.175		1.493	1.493		2.786	2.786			
6	Trp	8.005	4.479		3.071	3.158			7.100			10.014		7.469	7.384	7.010	7.140
7	Ala	7.753	4.069	1.054													
8	Lys	7.951	3.898		1.441	1.441	1.109	1.109		1.497	1.497		2.772	2.772			
9	Trp	7.665	4.556		3.174	3.174			7.146			10.116		7.494	7.434	7.075	7.173
10	Trp	7.532	4.41		2.963	2.963			6.919			9.959		7.277	7.377	7.014	7.138
11	Lys	7.705	4.014		1.448	1.448	1.131	1.131		1.596	1.596		2.853	2.853			
12	Lys	8.007	4.031		1.595	1.595	1.313	1.313		1.672	1.672		2.875	2.875			

Table S5. Chemical shifts of peptide 7b.

Residue		H^N	$H\alpha$	$H\beta$	$H\beta^2$	$H\beta^3$	$H\gamma$	$H\gamma^2$	$H\gamma^3$	$H\delta$	$H\delta^1$	$H\delta^2$	$H\delta^3$	$H\varepsilon^1$	$H\varepsilon^2$	$H\varepsilon^3$	$H\zeta^2$	$H\zeta^3$	$H\eta^2$
1	Lys		3.936		1.816	1.816		1.330	1.330			1.621	1.621		2.907	2.907			
2	Trp	8.686	4.677		3.180	3.180					7.163			10.111		7.546	7.429	7.055	7.090
3	Ile	7.937	3.988	1.620			1.009	1.309	1.254	0.732									
4	Lys	7.991	3.881		1.511	1.511		1.137	1.137			1.511	1.511		2.745	2.745			
5	Lys	8.091	4.173		1.594	1.594		1.190	1.252			1.544	1.544		2.835	2.835			
6	Trp	8.164	4.589		3.105	3.175					7.127			10.061		7.519	7.419	7.035	7.163
7	Ile	7.778	3.941	1.553			0.714	1.253	0.962	0.598									
8	Lys	8.016	3.914		1.441	1.441		1.037	1.080			1.414	1.414		2.734	2.734			
9	Trp	7.797	4.556		3.158	3.158					7.169			10.062		7.488	7.398	7.085	7.183
10	Trp	7.617	4.437		2.987	2.987					7.006			10.041		7.539	7.410	7.049	7.286
11	Lys	7.703	4.008		1.580	1.580		1.138	1.138			1.448	1.448		2.853	2.853			
12	Lys	8.032	4.037		1.672	1.672		1.313	1.313			1.595	1.595		2.875	2.875			

Table S6. Chemical shifts of peptide 8a.

Residue		H^N	$H\alpha$	$H\beta^2$	$H\beta^3$	$H\gamma$	$H\gamma^2$	$H\gamma^3$	$H\delta^1$	$H\delta^2$	$H\delta^3$	$H\varepsilon$	$H\varepsilon^1$	$H\varepsilon^3$	$H\zeta^2$	$H\zeta^3$	$H\eta^2$
1	Arg		3.961	1.834	1.834		1.538	1.538		3.130	3.130	7.127					
2	Trp	8.717	4.632	3.173	3.249				7.151				10.112	7.571	7.428	7.090	7.184
3	Leu	8.082	4.258	1.416	1.416	1.254				0.820	0.775						
4	Arg	8.001	3.954	1.493	1.493		1.284	1.284		2.968	2.968	6.984					
5	Arg	8.149	4.148	1.591	1.591		1.349	1.409		2.973	2.973	6.998					
6	Trp	8.075	4.610	3.121	3.203				7.190				10.064	7.502	7.447	7.096	7.182
7	Leu	7.762	4.192	1.317	1.317	1.309				0.816	0.759						
8	Arg	7.995	3.952	1.500	1.500		1.364	1.364		2.960	2.960	6.984					
9	Trp	7.776	4.601	3.182	3.182				7.116				10.057	7.516	7.403	7.051	7.159
10	Trp	7.646	4.464	3.008	3.008				6.988				10.003	7.270	7.401	7.041	7.155
11	Arg	7.826	4.066	1.485	1.651		1.345	1.345		3.037	3.037	7.049					
12	Arg	8.07	4.091	1.624	1.733		1.523	1.523		3.059	3.059	7.059					

Table S7. Chemical shifts of peptide 8b.

Residue		H^N	$H\alpha$	$H\beta^2$	$H\beta^3$	$H\gamma$	$H\gamma^2$	$H\gamma^3$	$H\delta^1$	$H\delta^2$	$H\delta^3$	$H\varepsilon^1$	$H\varepsilon^2$	$H\varepsilon^3$	$H\zeta^2$	$H\zeta^3$	$H\eta^2$
1	Lys		3.961	1.792	1.792		1.315	1.315		1.615	1.615		2.898	2.898			
2	Trp	8.653	4.670	3.153	3.218				7.146			10.113		7.441	7.497	7.085	7.182
3	Leu	7.980	4.248	1.381	1.381	1.381			0.801	0.761							
4	Lys	7.897	3.918	1.487	1.487		1.158	1.158		1.487	1.487		2.779	2.764			
5	Lys	8.046	4.109	1.489	1.576		1.151	1.151		1.506	1.506		2.749	2.749			
6	Trp	7.993	4.562	3.105	3.175				7.177			10.043		7.571	7.416	7.063	7.161
7	Leu	7.617	4.162	1.262	1.348	1.253			0.772	0.772							
8	Lys	7.939	3.920	1.441	1.441		1.089	1.089		1.424	1.424		2.778	2.762			
9	Trp	7.665	4.556	3.193	3.144				7.088			10.064		7.508	7.401	7.041	7.146
10	Trp	7.548	4.420	2.987	2.934				6.922			9.981		7.212	7.393	7.024	7.142
11	Lys	7.725	4.032	1.454	1.454		1.138	1.138		1.612	1.612		2.870	2.849			
12	Lys	8.020	4.041	1.595	1.595		1.313	1.313		1.665	1.665		2.885	2.885			
